# Supplementary figures and images for: Generation of adult hippocampal neural stem cells occurs in the early postnatal dentate gyrus and depends on cyclin D2 (part 1 of 2)
Source: EMBO J. 2023 Dec 20;43(3):1. doi: 10.1038/s44318-023-00011-2 (PMC10897295; doi:10.1038/s44318-023-00011-2)

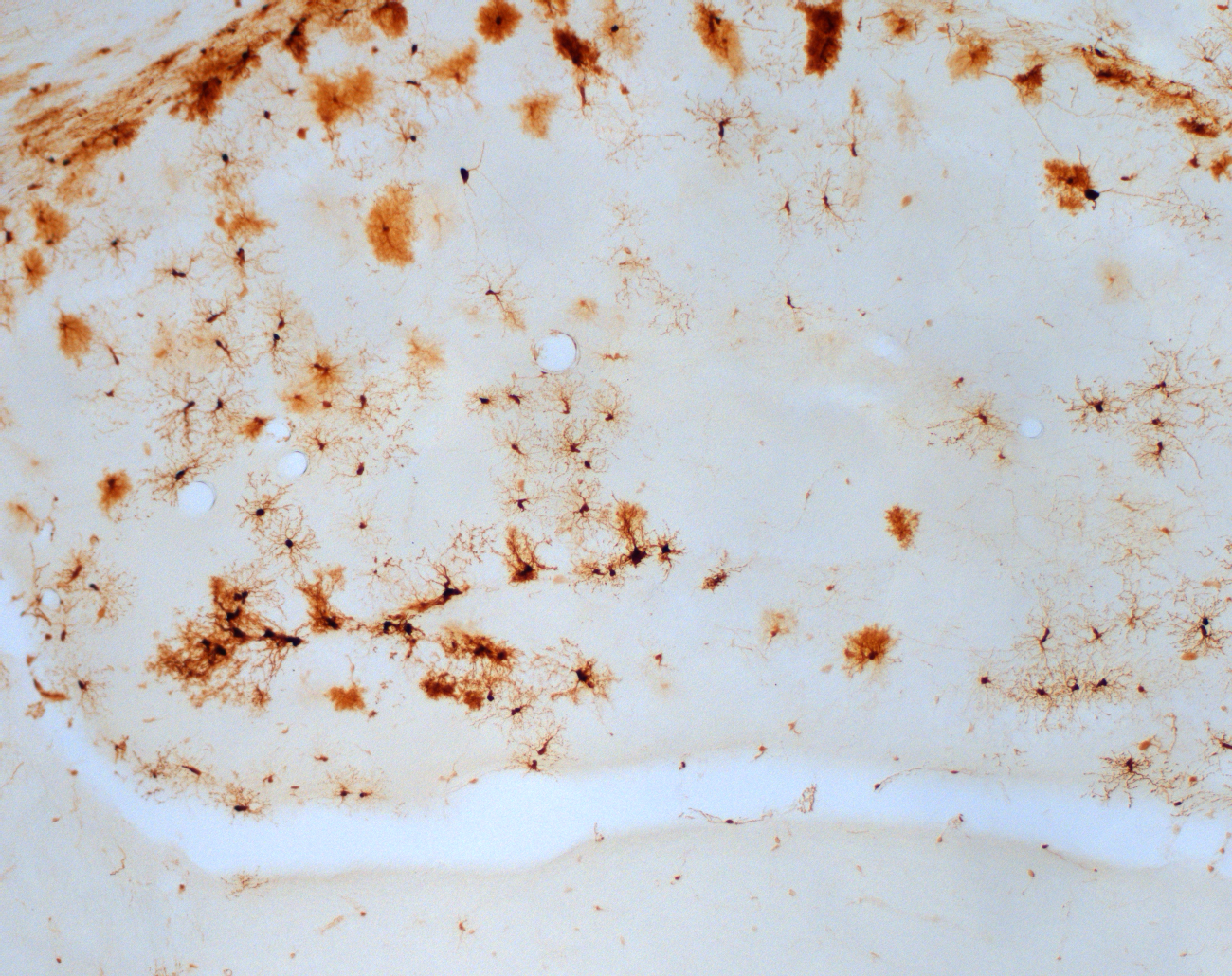

Supplement: Supplementary file 2 — Source Data Fig. 1 [file 44318_2023_11_MOESM2_ESM.zip › EMBOJ-2023-113564_SourceDataForFigure1/1A/KO_36872_S3_rechts.tif]

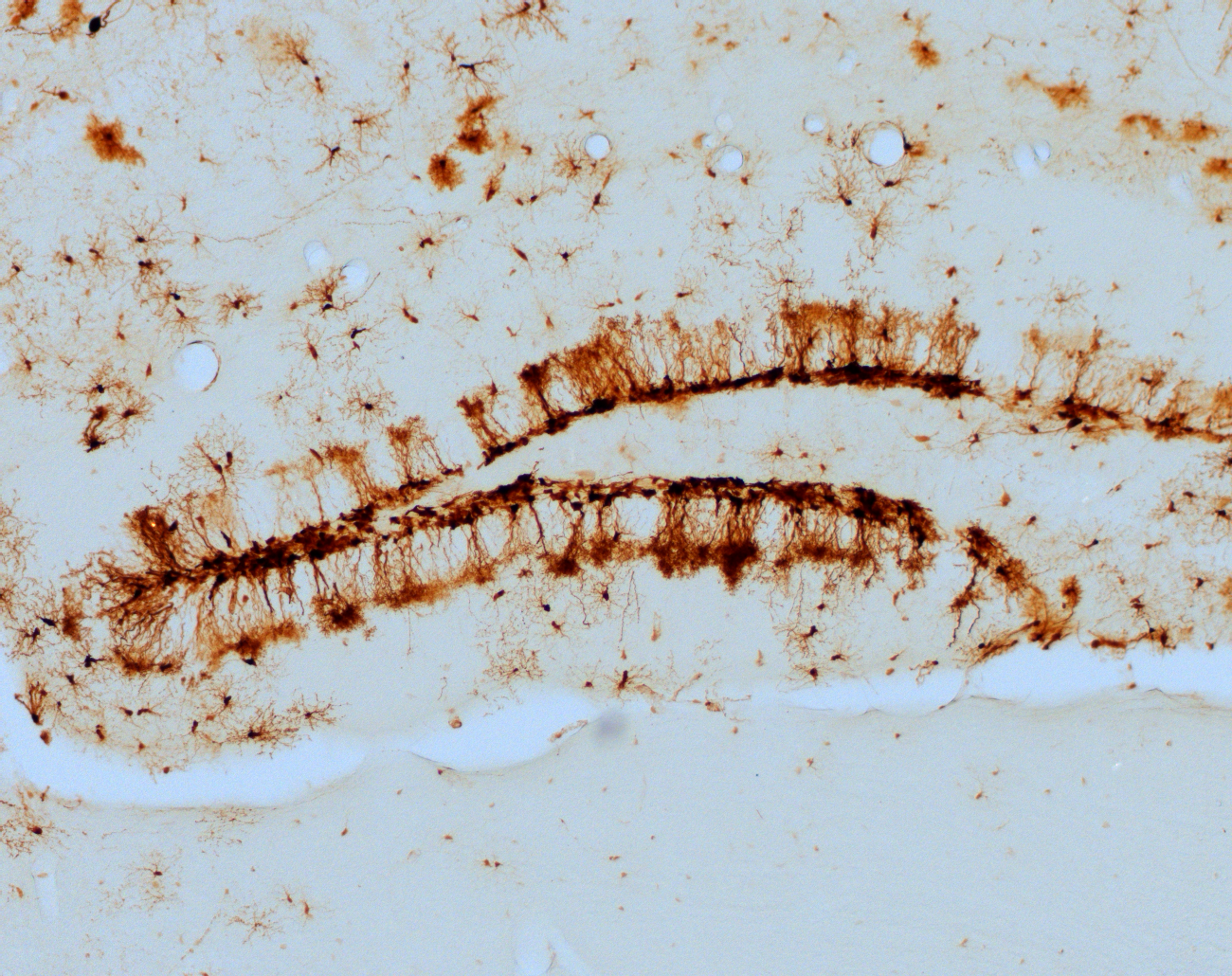

Supplement: Supplementary file 2 — Source Data Fig. 1 [file 44318_2023_11_MOESM2_ESM.zip › EMBOJ-2023-113564_SourceDataForFigure1/1A/WT_36884_S4_rechts.tif]

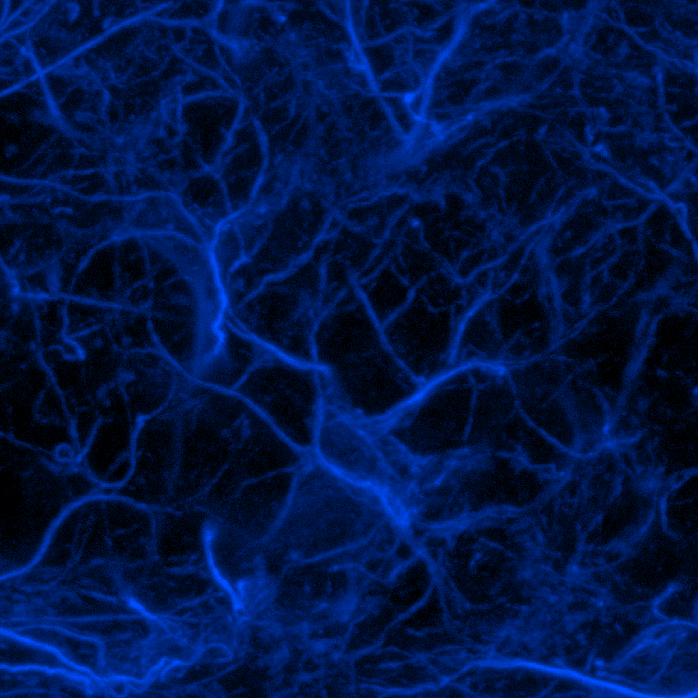

Supplement: Supplementary file 2 — Source Data Fig. 1 [file 44318_2023_11_MOESM2_ESM.zip › EMBOJ-2023-113564_SourceDataForFigure1/1D/KO_NSC_GFAP.tif]

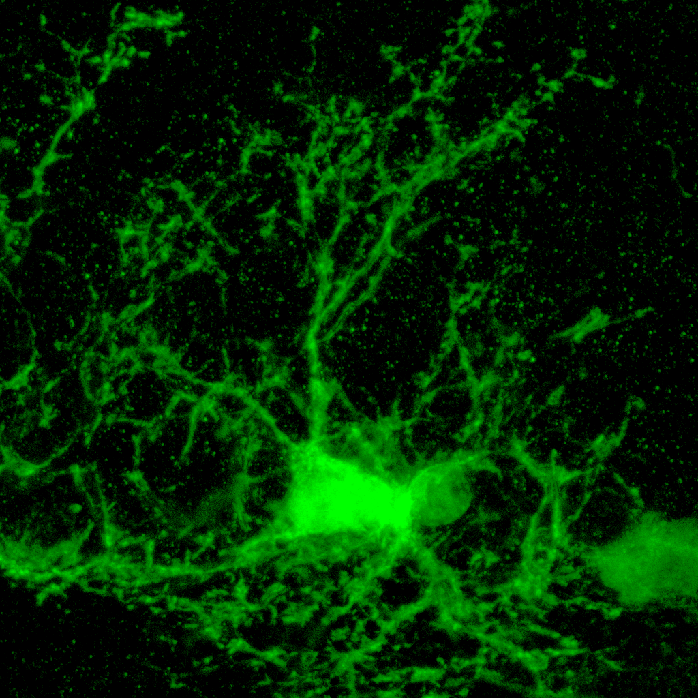

Supplement: Supplementary file 2 — Source Data Fig. 1 [file 44318_2023_11_MOESM2_ESM.zip › EMBOJ-2023-113564_SourceDataForFigure1/1D/KO_NSC_GFP.tif]

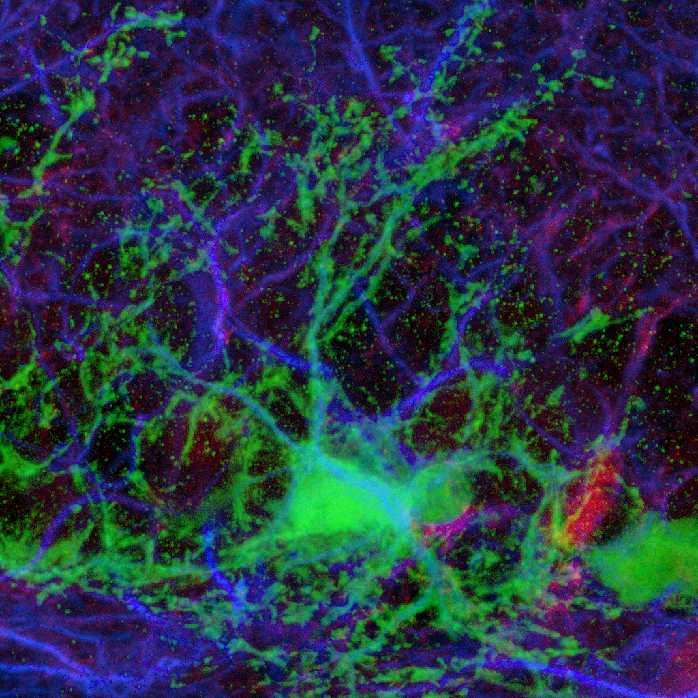

Supplement: Supplementary file 2 — Source Data Fig. 1 [file 44318_2023_11_MOESM2_ESM.zip › EMBOJ-2023-113564_SourceDataForFigure1/1D/KO_NSC_merged.tif]

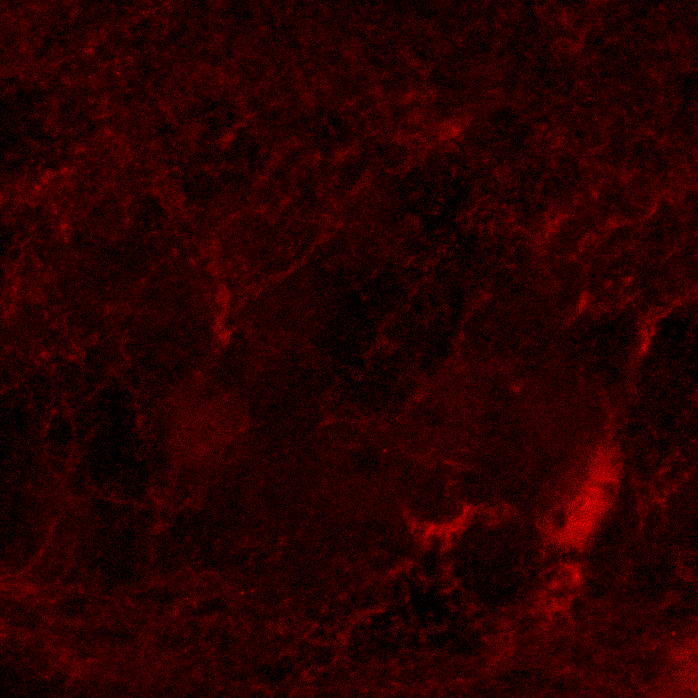

Supplement: Supplementary file 2 — Source Data Fig. 1 [file 44318_2023_11_MOESM2_ESM.zip › EMBOJ-2023-113564_SourceDataForFigure1/1D/KO_NSC_S100ß.tif]

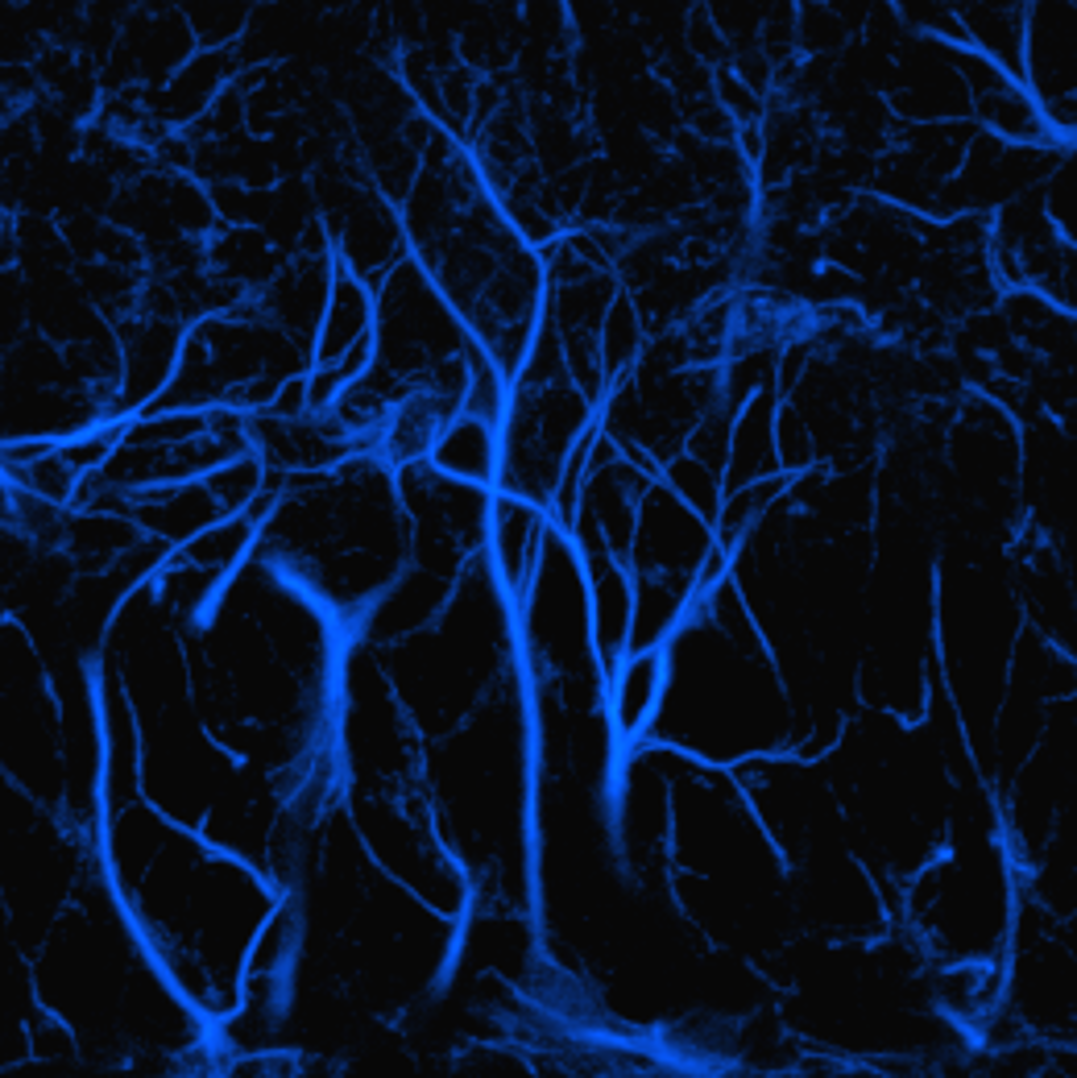

Supplement: Supplementary file 2 — Source Data Fig. 1 [file 44318_2023_11_MOESM2_ESM.zip › EMBOJ-2023-113564_SourceDataForFigure1/1D/WT_NSC_GFAP.png]

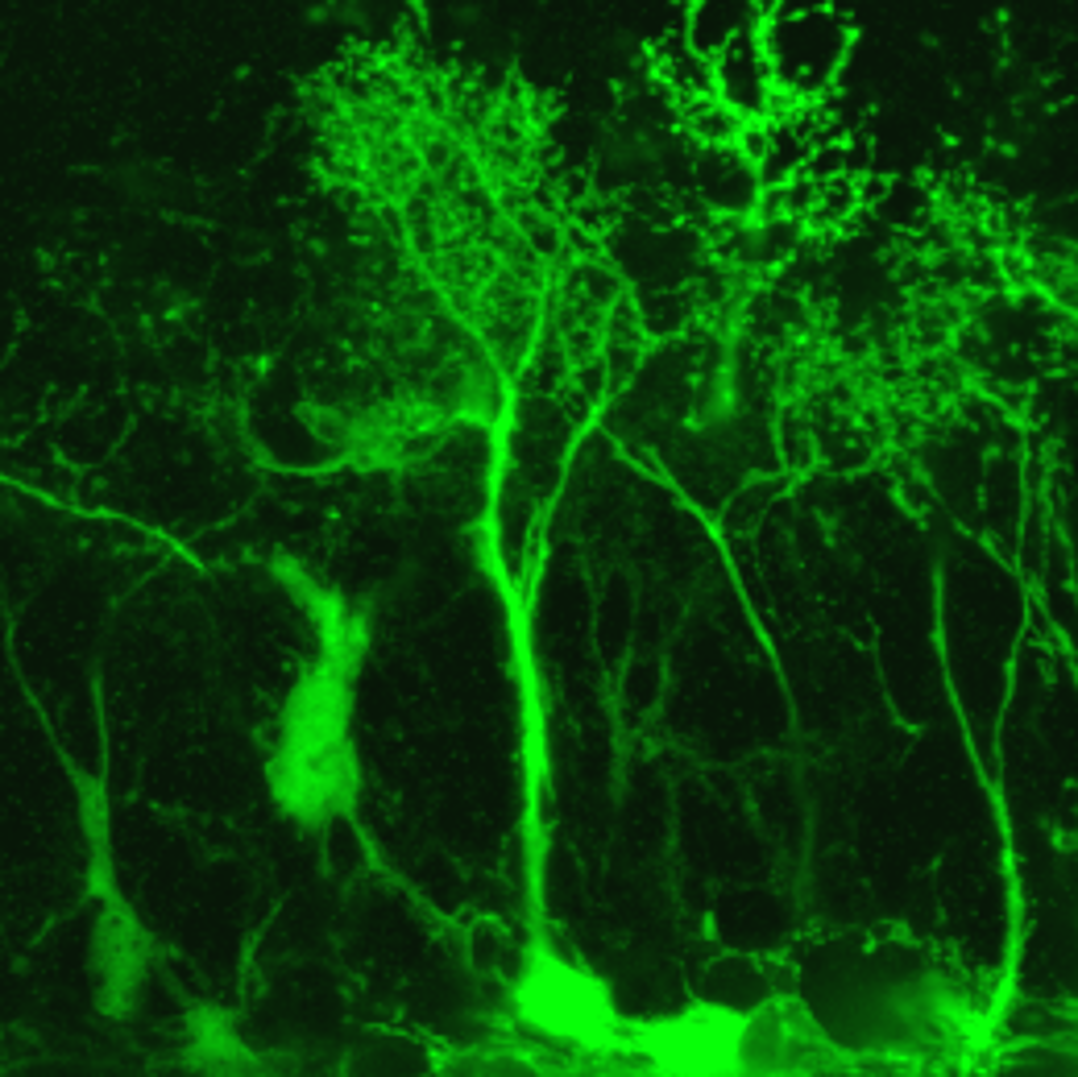

Supplement: Supplementary file 2 — Source Data Fig. 1 [file 44318_2023_11_MOESM2_ESM.zip › EMBOJ-2023-113564_SourceDataForFigure1/1D/WT_NSC_GFP.png]

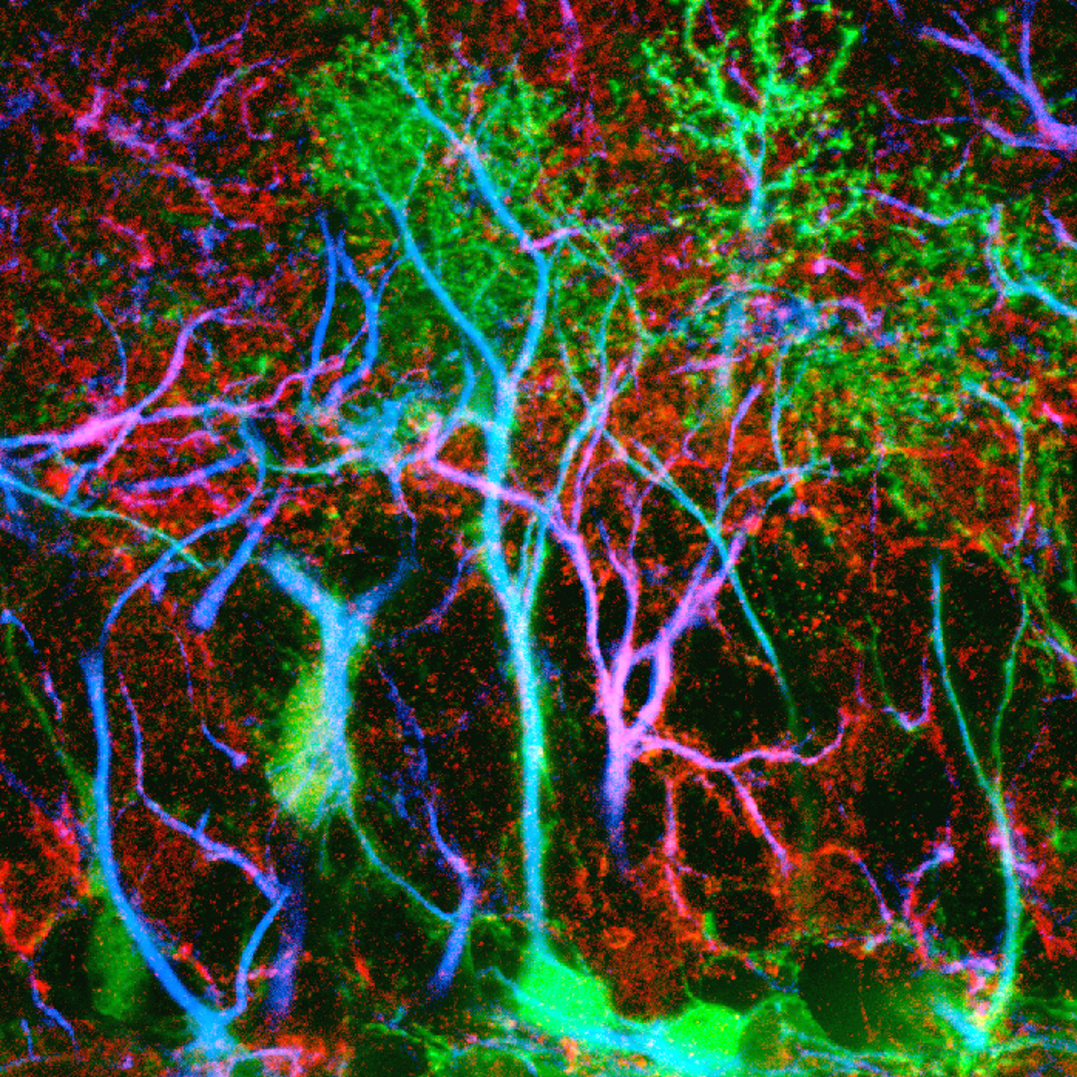

Supplement: Supplementary file 2 — Source Data Fig. 1 [file 44318_2023_11_MOESM2_ESM.zip › EMBOJ-2023-113564_SourceDataForFigure1/1D/WT_NSC_merged.png]

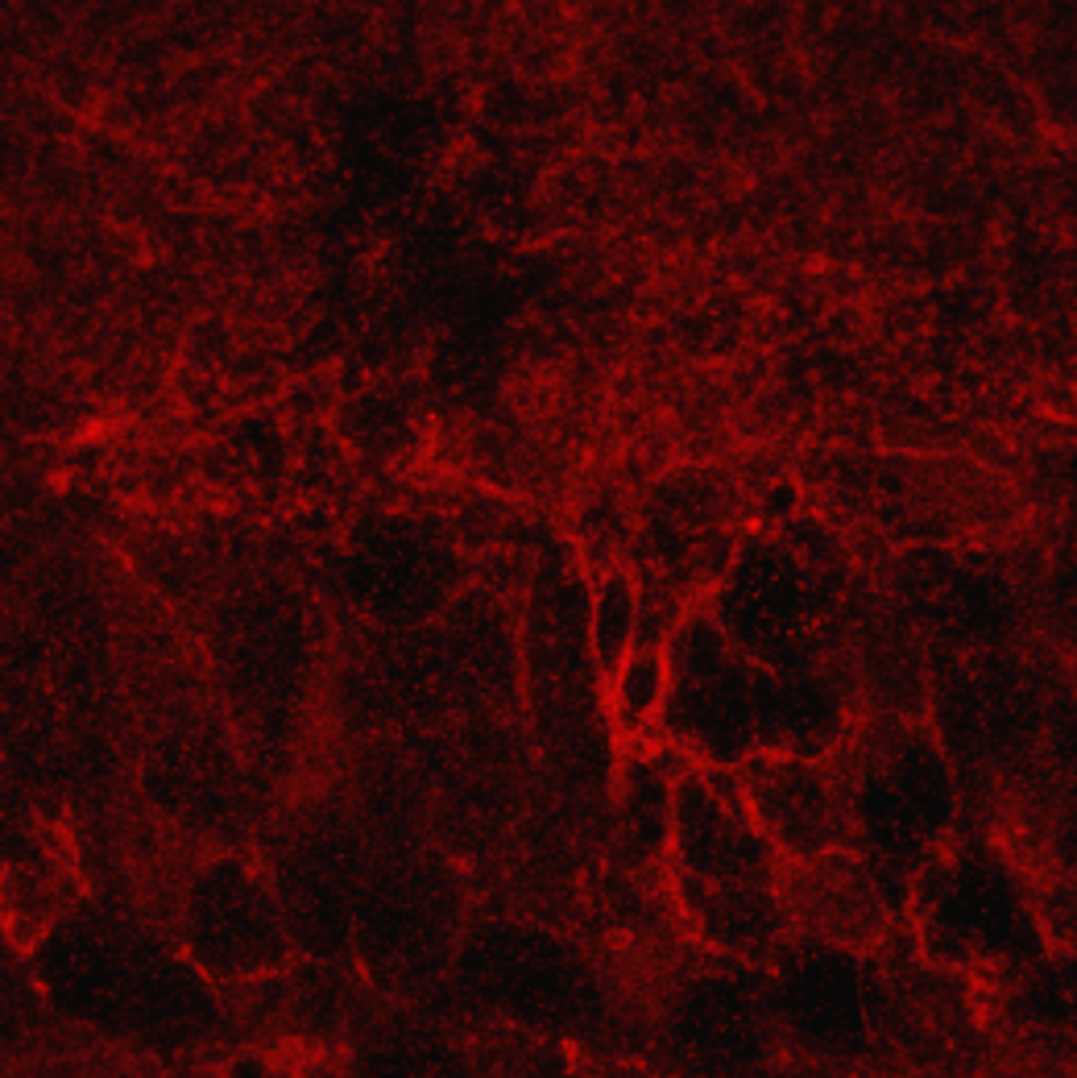

Supplement: Supplementary file 2 — Source Data Fig. 1 [file 44318_2023_11_MOESM2_ESM.zip › EMBOJ-2023-113564_SourceDataForFigure1/1D/WT_NSC_S100ß.png]

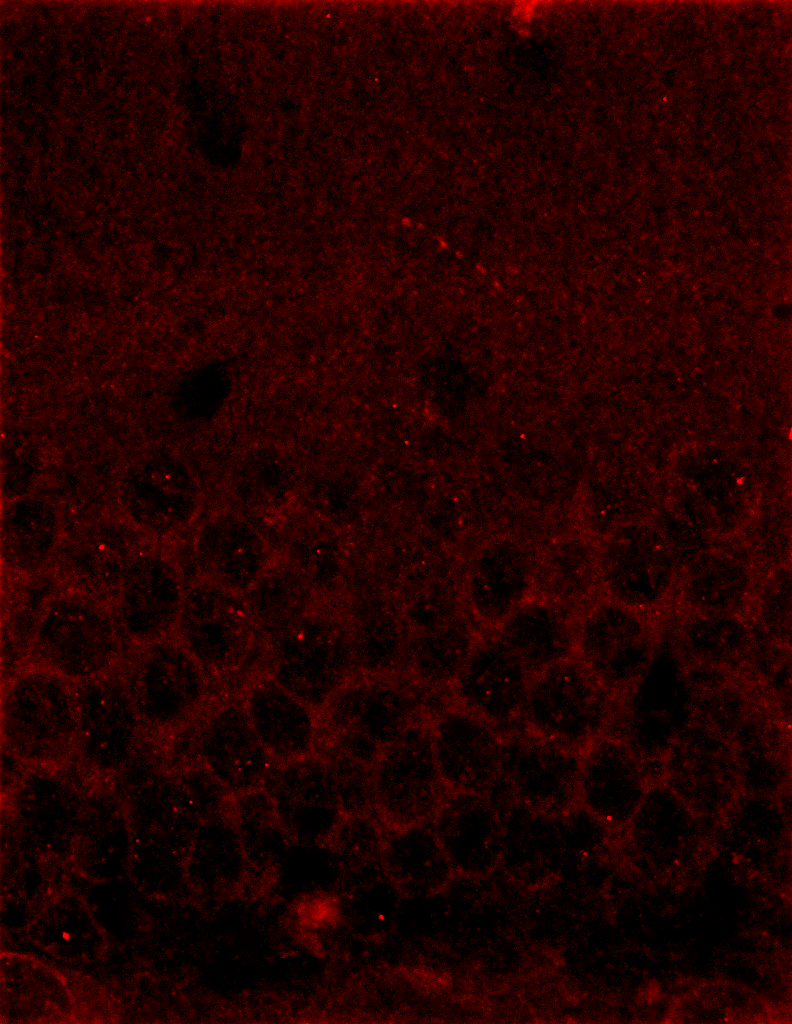

Supplement: Supplementary file 2 — Source Data Fig. 1 [file 44318_2023_11_MOESM2_ESM.zip › EMBOJ-2023-113564_SourceDataForFigure1/1L/WT_NSC_D2.tif]

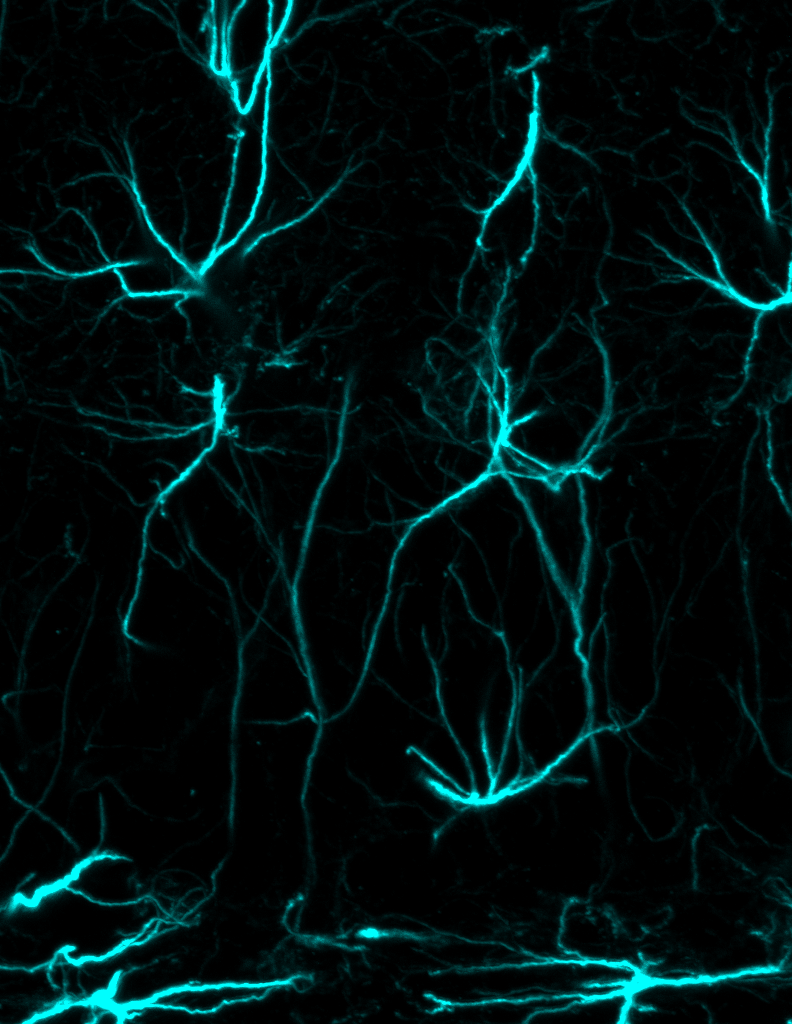

Supplement: Supplementary file 2 — Source Data Fig. 1 [file 44318_2023_11_MOESM2_ESM.zip › EMBOJ-2023-113564_SourceDataForFigure1/1L/WT_NSC_GFAP.tif]

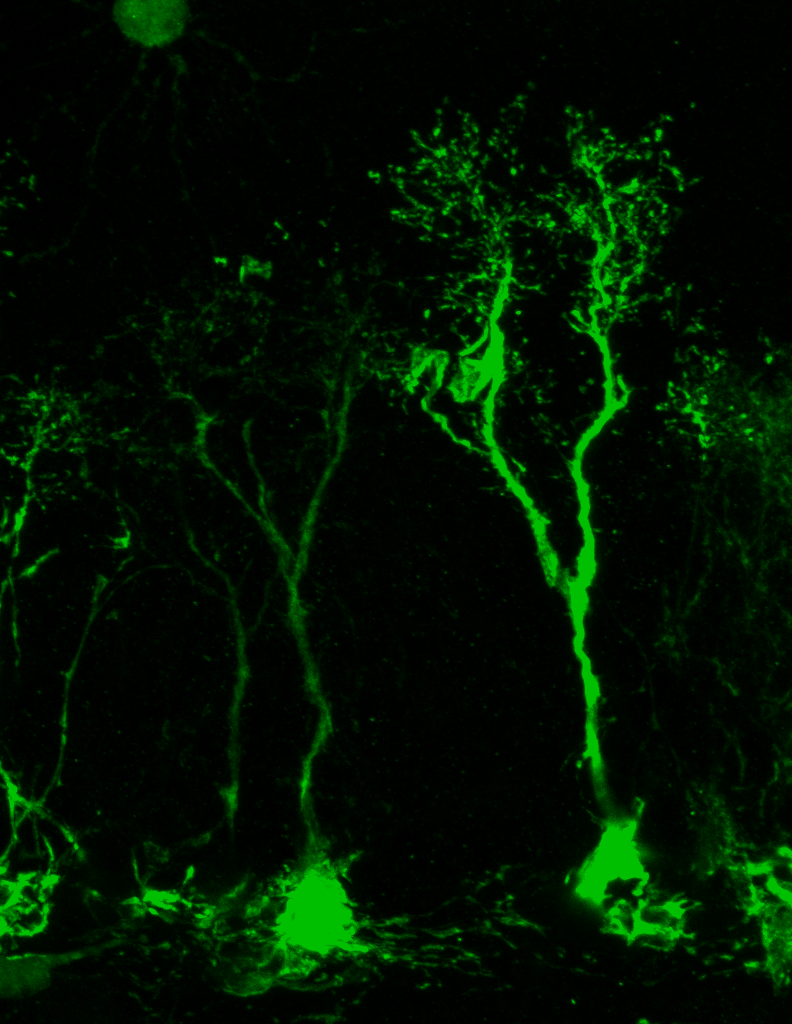

Supplement: Supplementary file 2 — Source Data Fig. 1 [file 44318_2023_11_MOESM2_ESM.zip › EMBOJ-2023-113564_SourceDataForFigure1/1L/WT_NSC_GFP.tif]

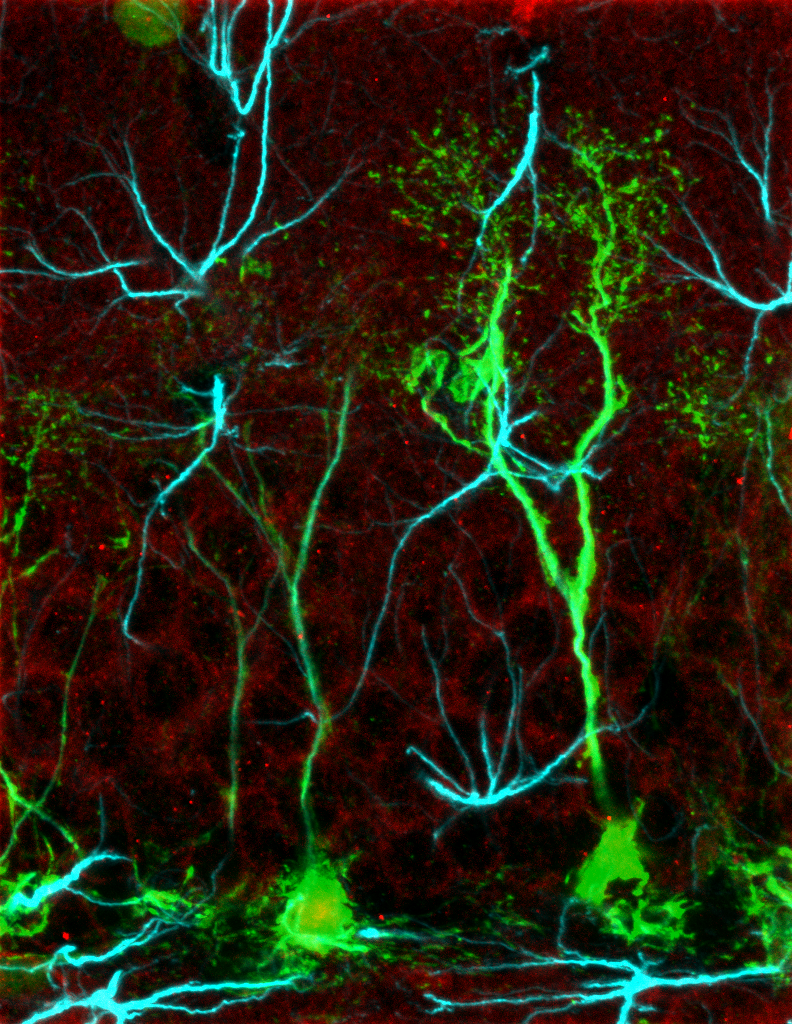

Supplement: Supplementary file 2 — Source Data Fig. 1 [file 44318_2023_11_MOESM2_ESM.zip › EMBOJ-2023-113564_SourceDataForFigure1/1L/WT_NSC_merge.tif]

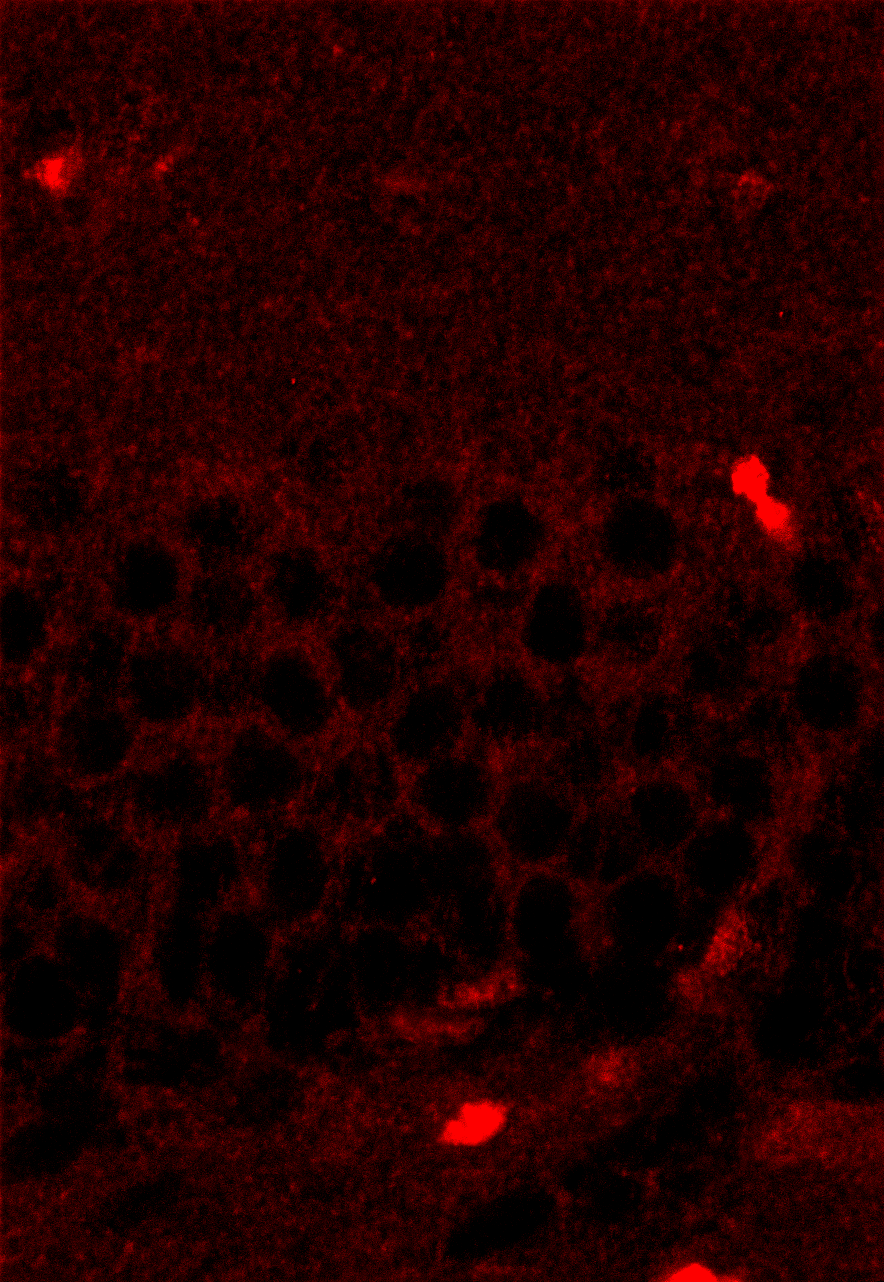

Supplement: Supplementary file 2 — Source Data Fig. 1 [file 44318_2023_11_MOESM2_ESM.zip › EMBOJ-2023-113564_SourceDataForFigure1/1M/WT_NSC_D1.tif]

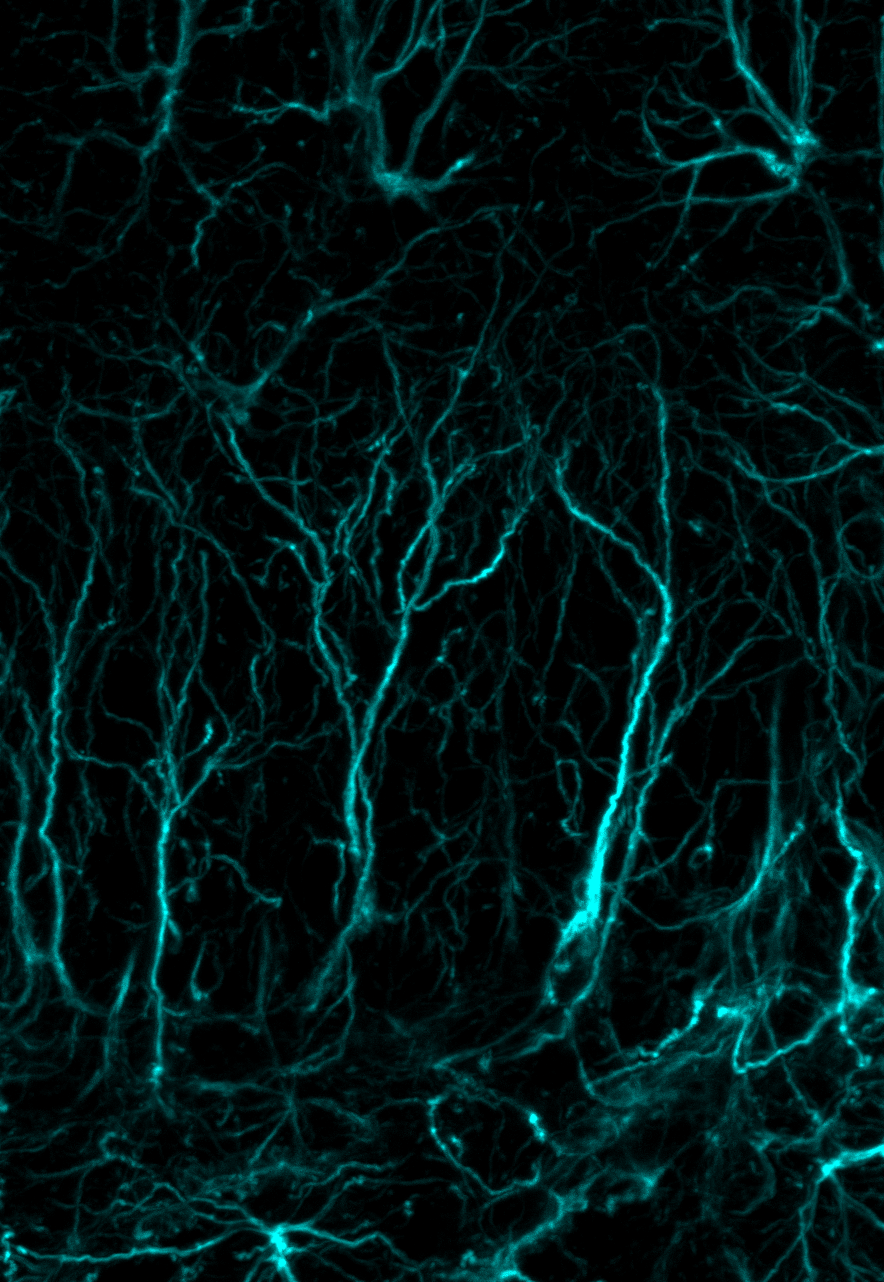

Supplement: Supplementary file 2 — Source Data Fig. 1 [file 44318_2023_11_MOESM2_ESM.zip › EMBOJ-2023-113564_SourceDataForFigure1/1M/WT_NSC_GFAP.tif]

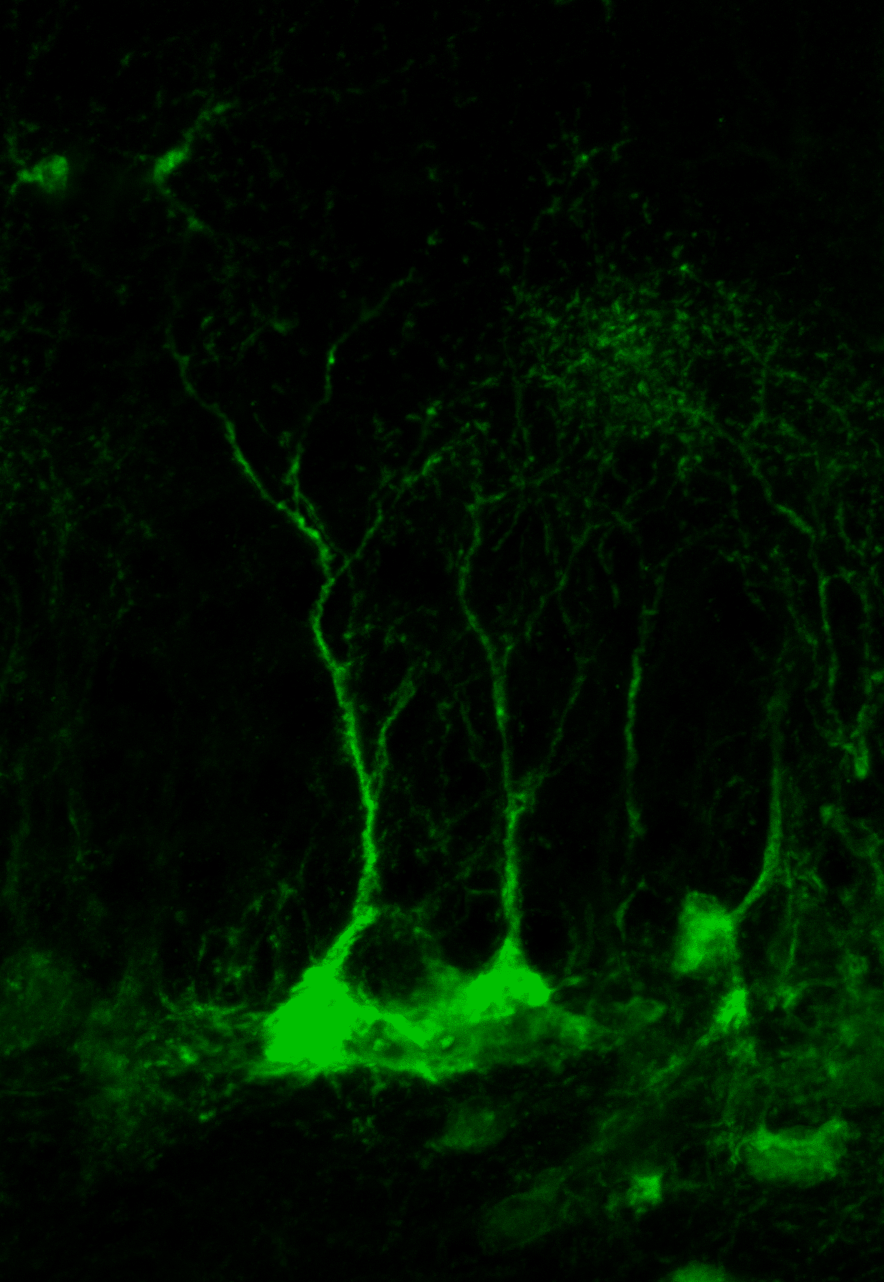

Supplement: Supplementary file 2 — Source Data Fig. 1 [file 44318_2023_11_MOESM2_ESM.zip › EMBOJ-2023-113564_SourceDataForFigure1/1M/WT_NSC_GFP.tif]

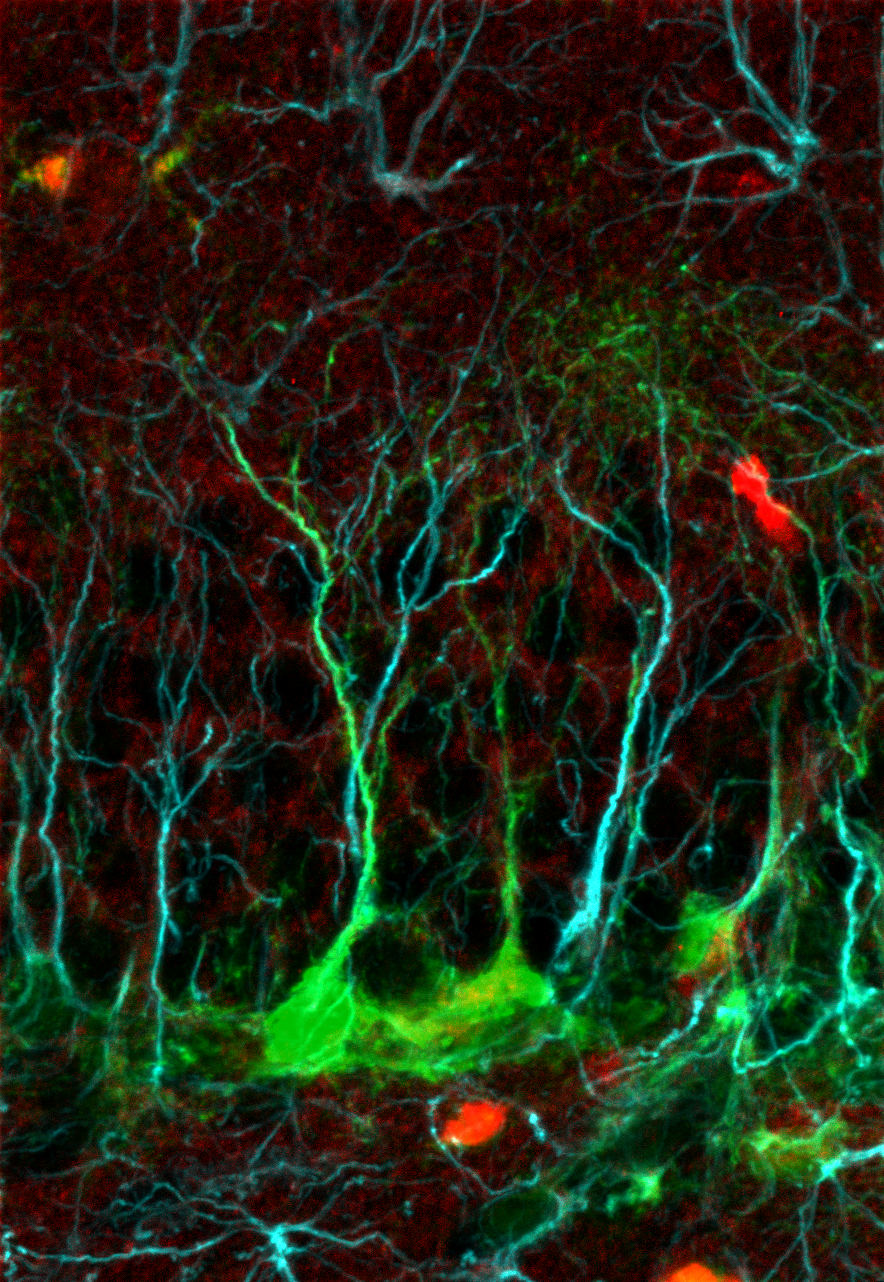

Supplement: Supplementary file 2 — Source Data Fig. 1 [file 44318_2023_11_MOESM2_ESM.zip › EMBOJ-2023-113564_SourceDataForFigure1/1M/WT_NSC_merge.tif]

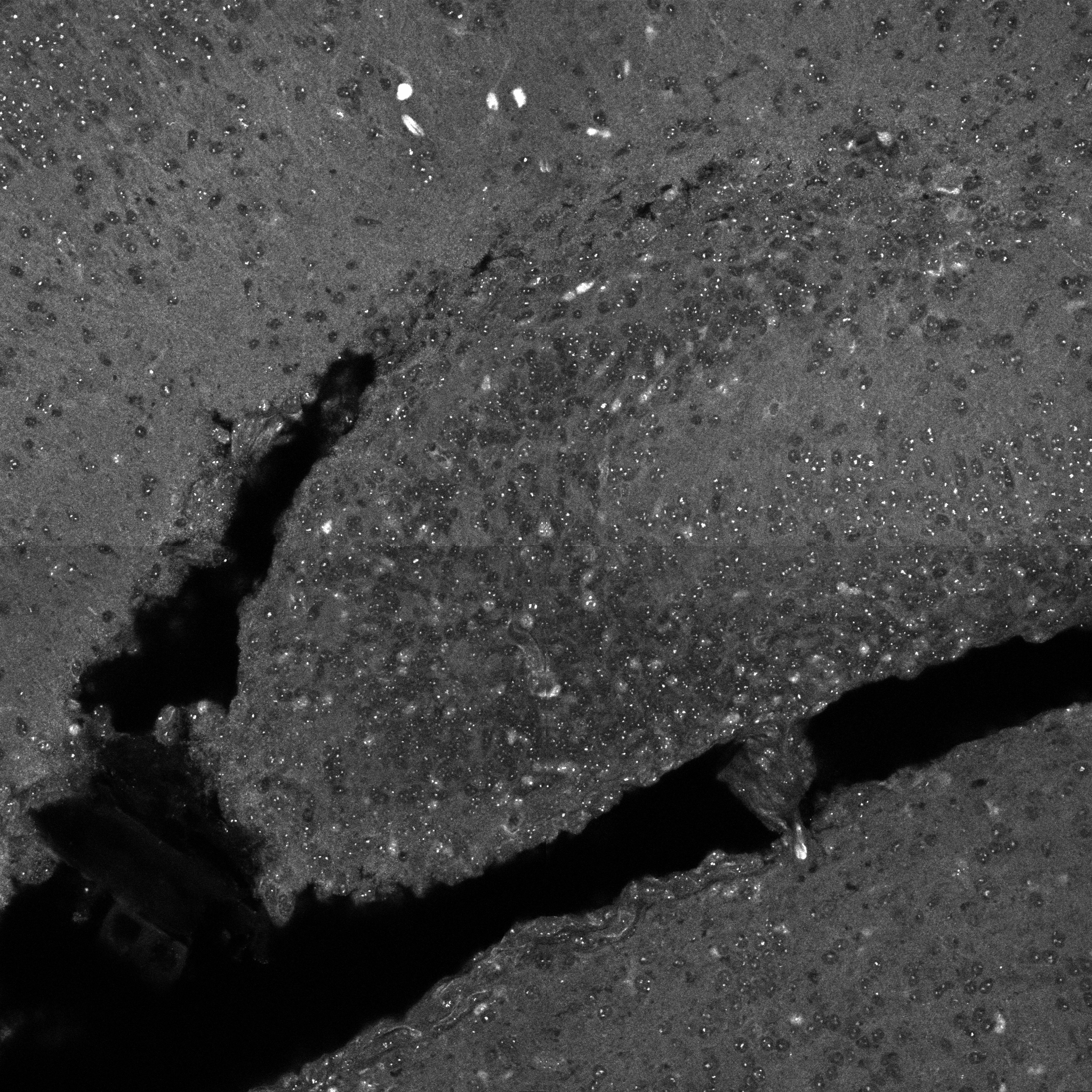

Supplement: Supplementary file 3 — Source Data Fig. 2 [file 44318_2023_11_MOESM3_ESM.zip › EMBOJ-2023-113564_SourceDataForFigure2/2B/P0_D2.png]

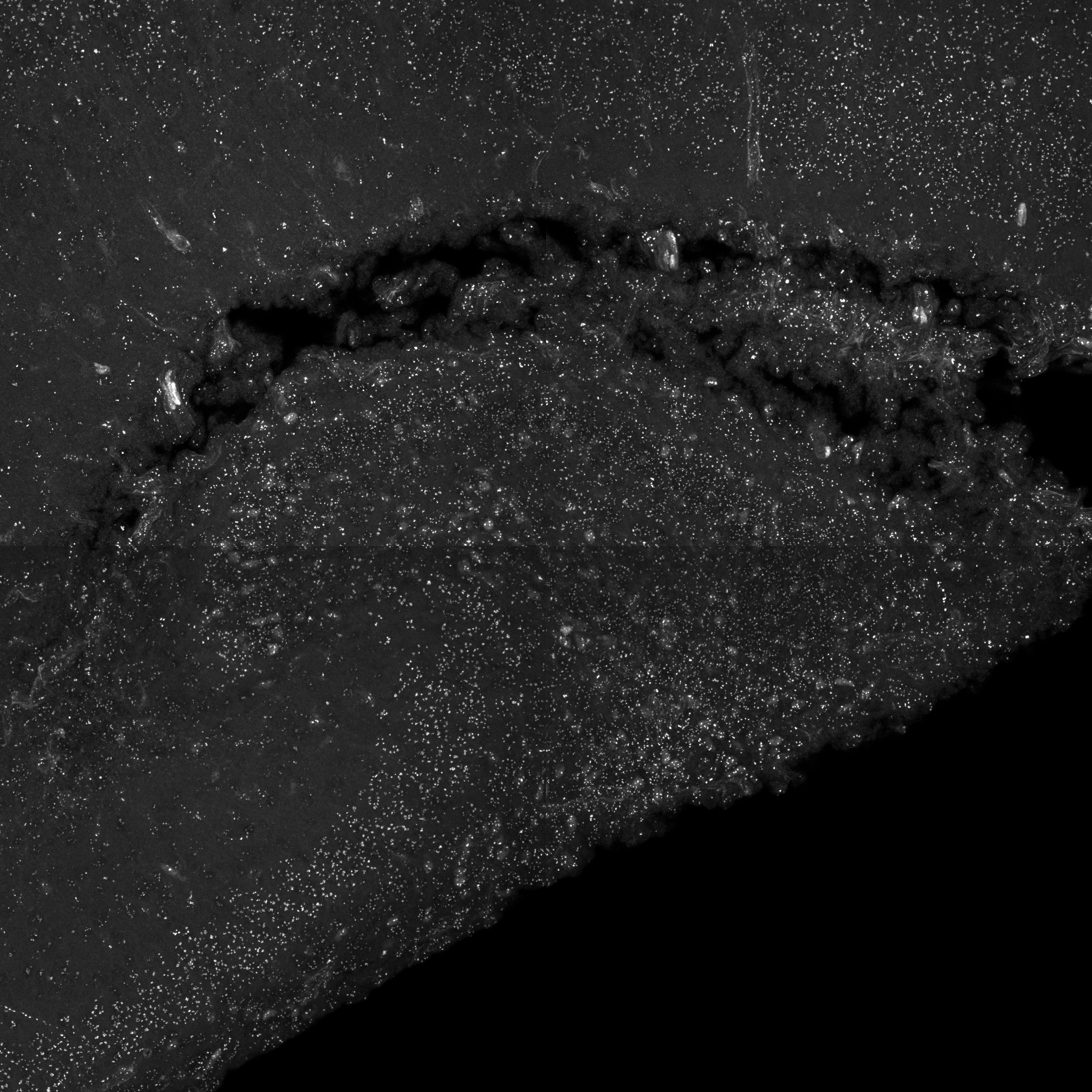

Supplement: Supplementary file 3 — Source Data Fig. 2 [file 44318_2023_11_MOESM3_ESM.zip › EMBOJ-2023-113564_SourceDataForFigure2/2B/P0_D2_replicate.png]

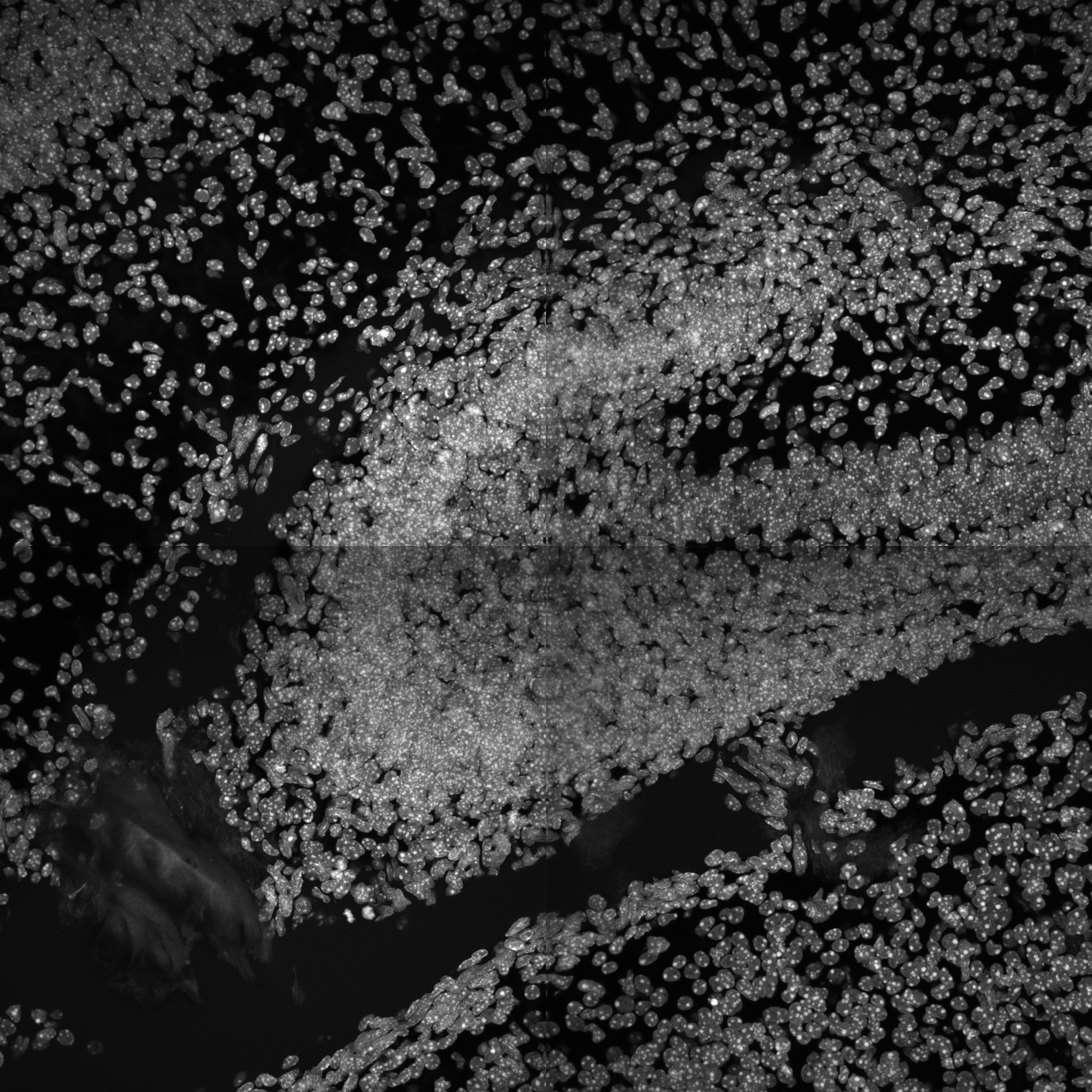

Supplement: Supplementary file 3 — Source Data Fig. 2 [file 44318_2023_11_MOESM3_ESM.zip › EMBOJ-2023-113564_SourceDataForFigure2/2B/P0_DAPI.png]

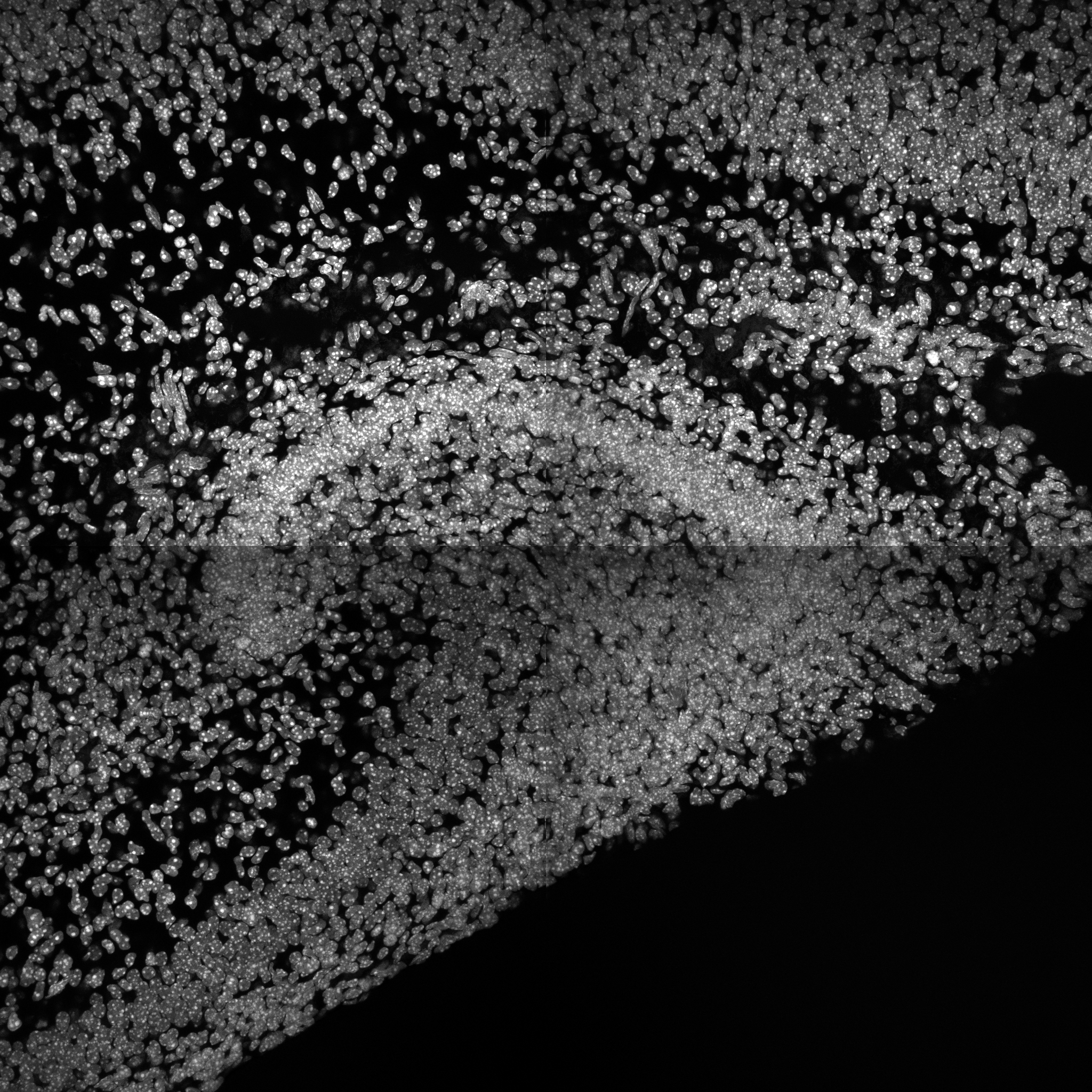

Supplement: Supplementary file 3 — Source Data Fig. 2 [file 44318_2023_11_MOESM3_ESM.zip › EMBOJ-2023-113564_SourceDataForFigure2/2B/P0_DAPI_replicate.png]

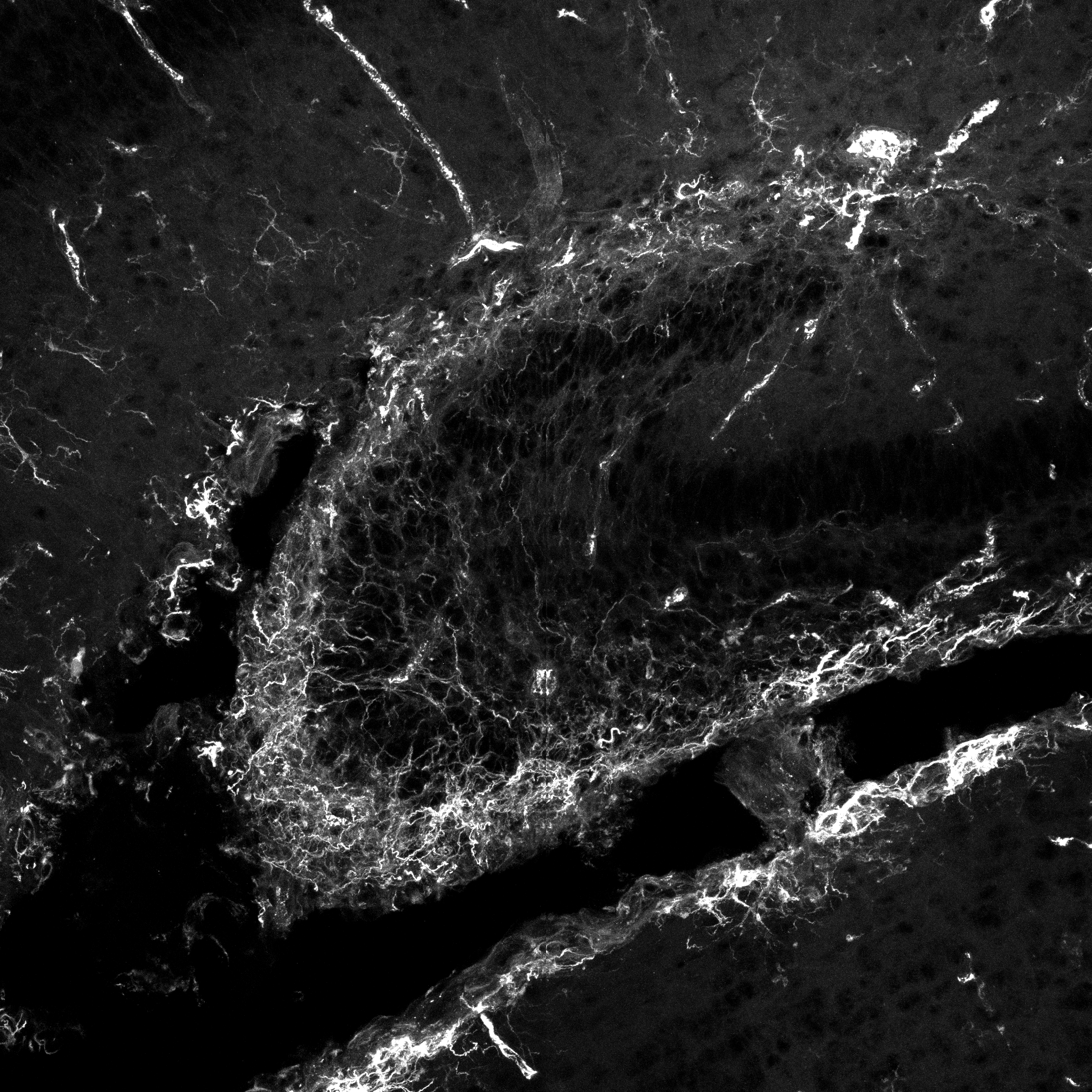

Supplement: Supplementary file 3 — Source Data Fig. 2 [file 44318_2023_11_MOESM3_ESM.zip › EMBOJ-2023-113564_SourceDataForFigure2/2B/P0_GFAP.png]

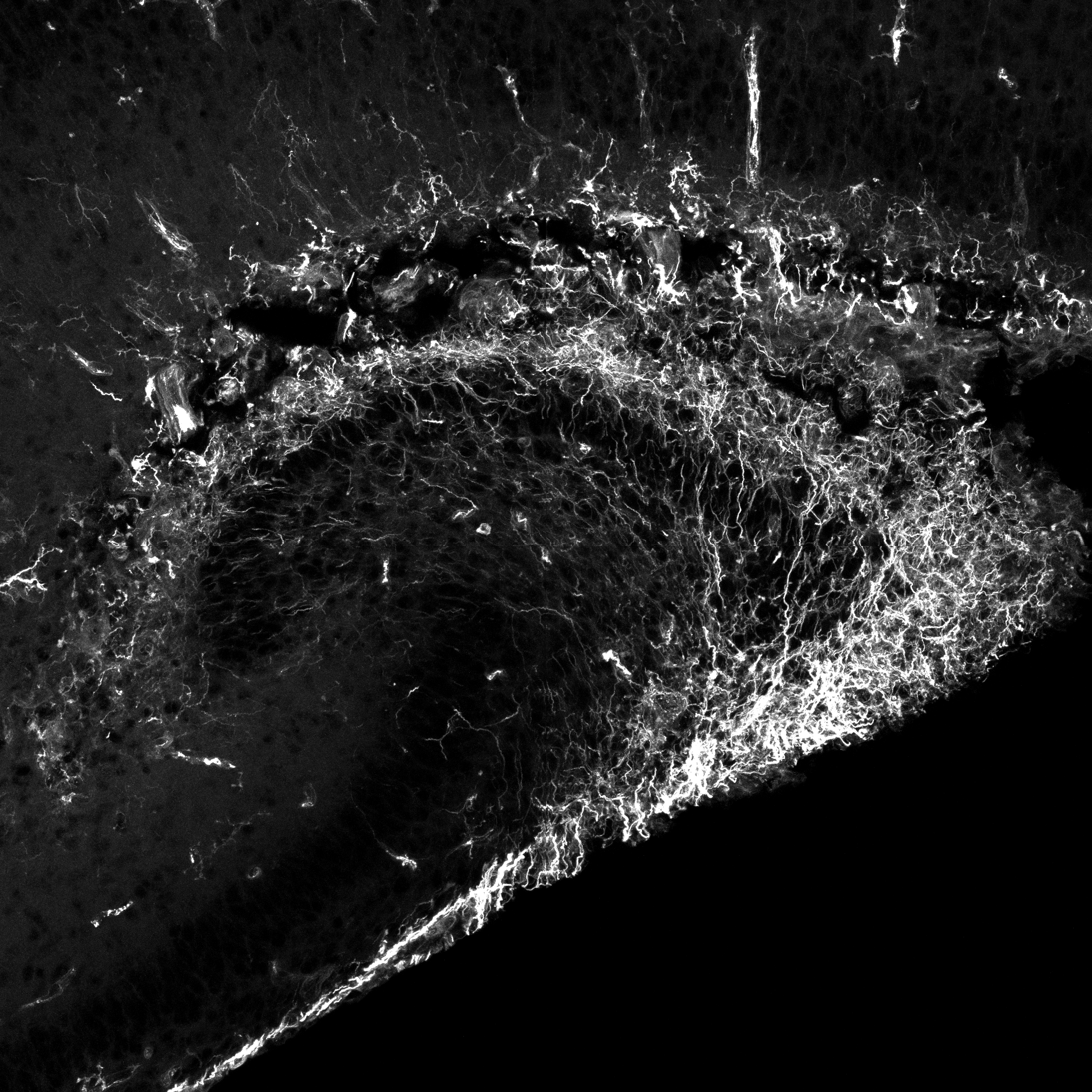

Supplement: Supplementary file 3 — Source Data Fig. 2 [file 44318_2023_11_MOESM3_ESM.zip › EMBOJ-2023-113564_SourceDataForFigure2/2B/P0_GFAP_replicate.png]

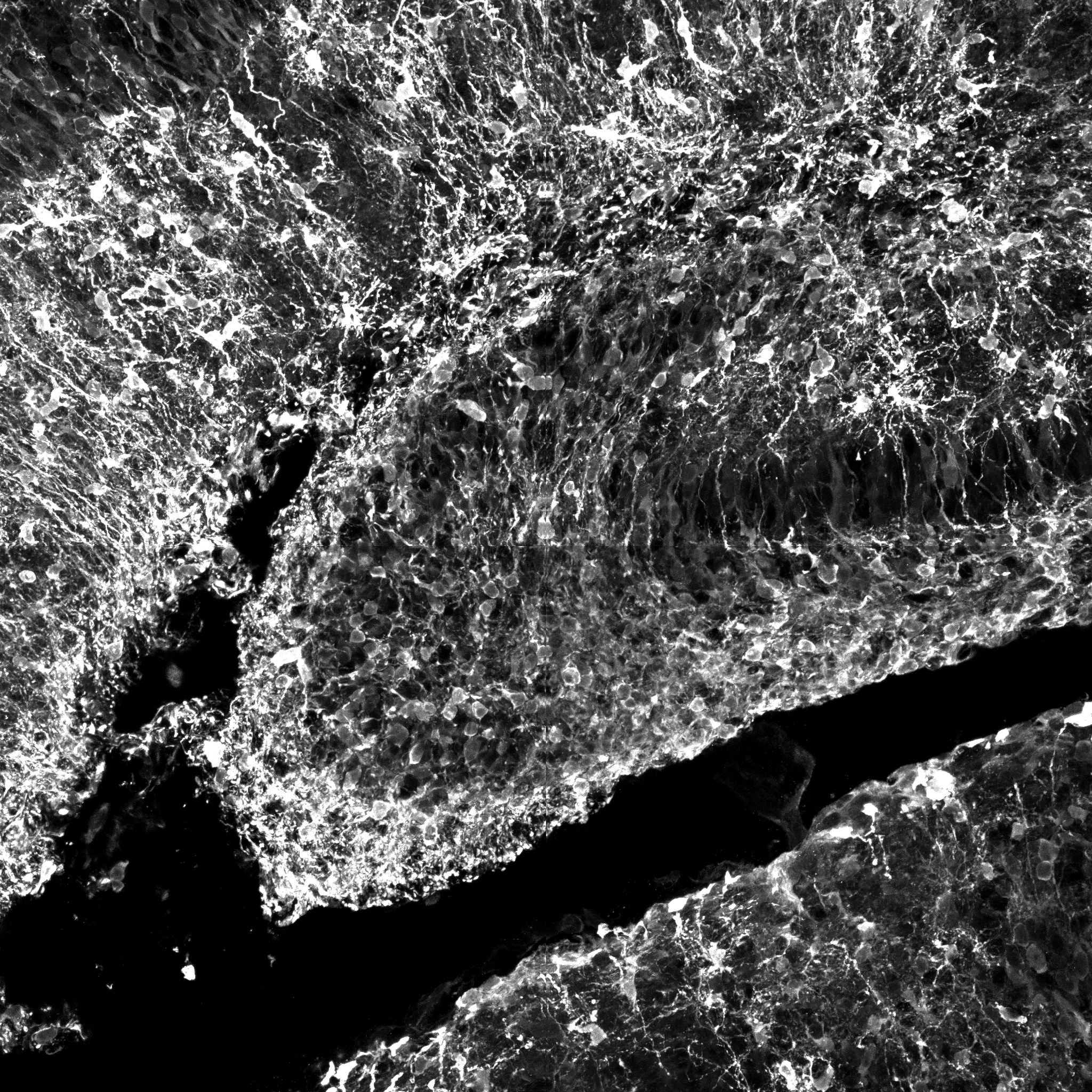

Supplement: Supplementary file 3 — Source Data Fig. 2 [file 44318_2023_11_MOESM3_ESM.zip › EMBOJ-2023-113564_SourceDataForFigure2/2B/P0_GFP.png]

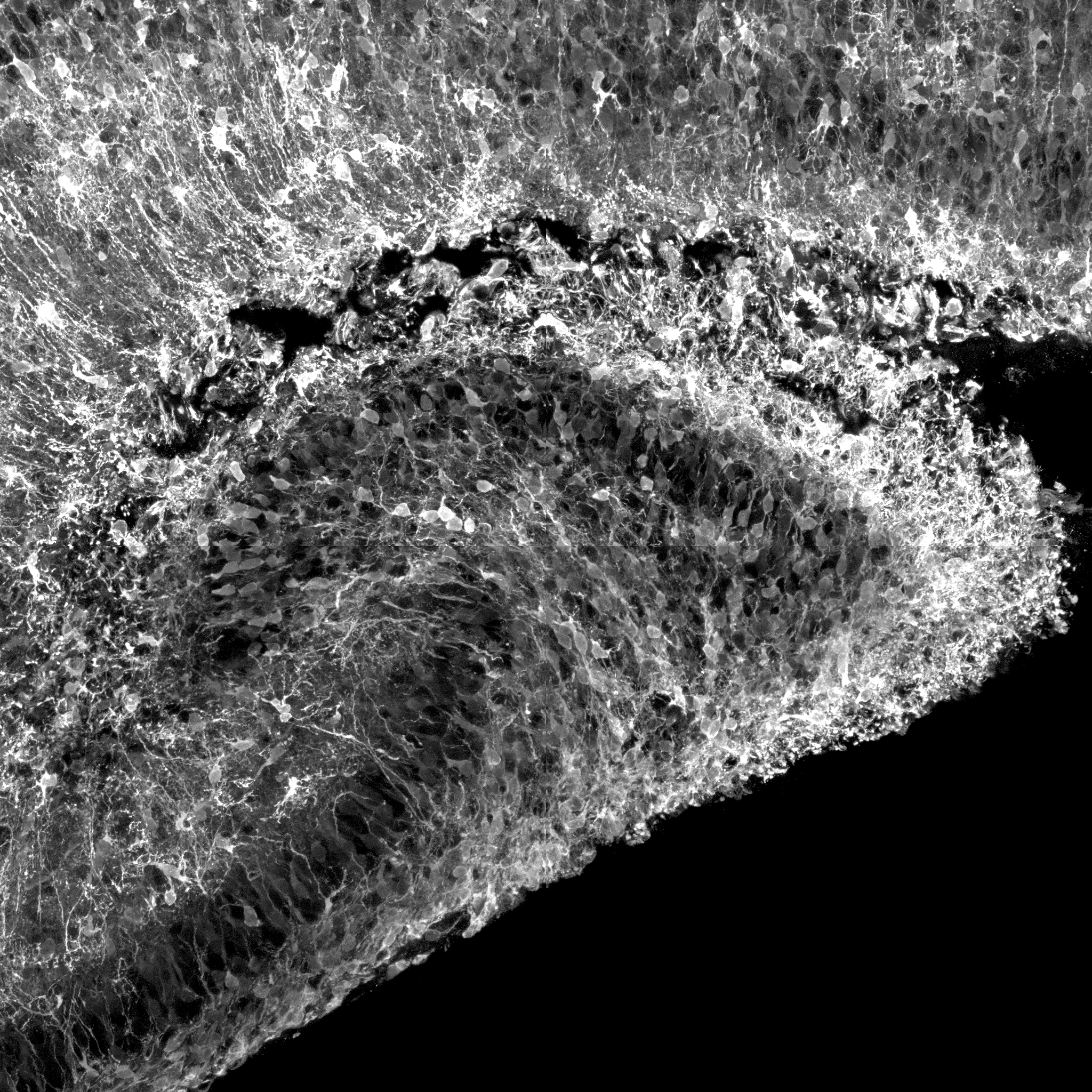

Supplement: Supplementary file 3 — Source Data Fig. 2 [file 44318_2023_11_MOESM3_ESM.zip › EMBOJ-2023-113564_SourceDataForFigure2/2B/P0_GFP_replicate.png]

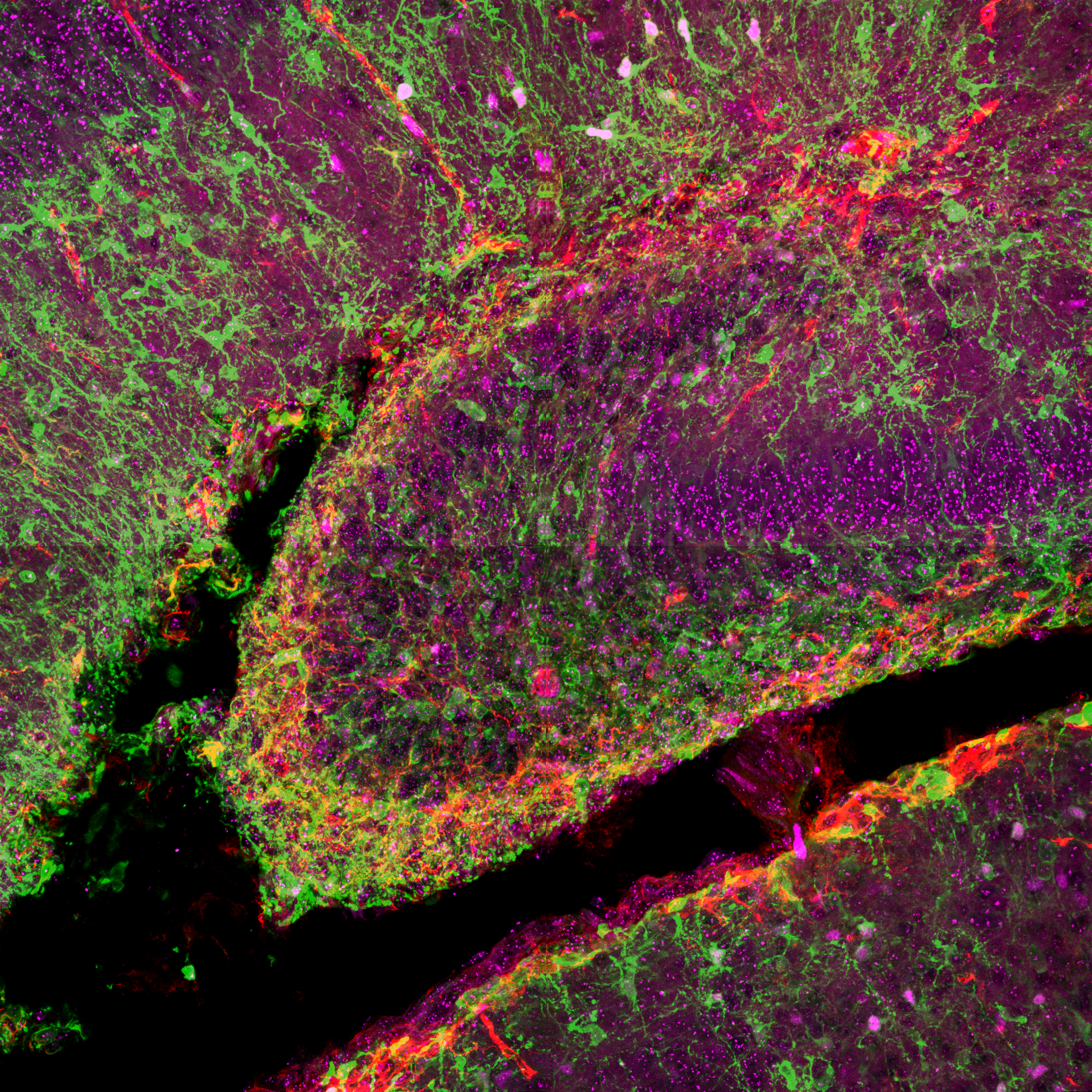

Supplement: Supplementary file 3 — Source Data Fig. 2 [file 44318_2023_11_MOESM3_ESM.zip › EMBOJ-2023-113564_SourceDataForFigure2/2B/P0_merge.png]

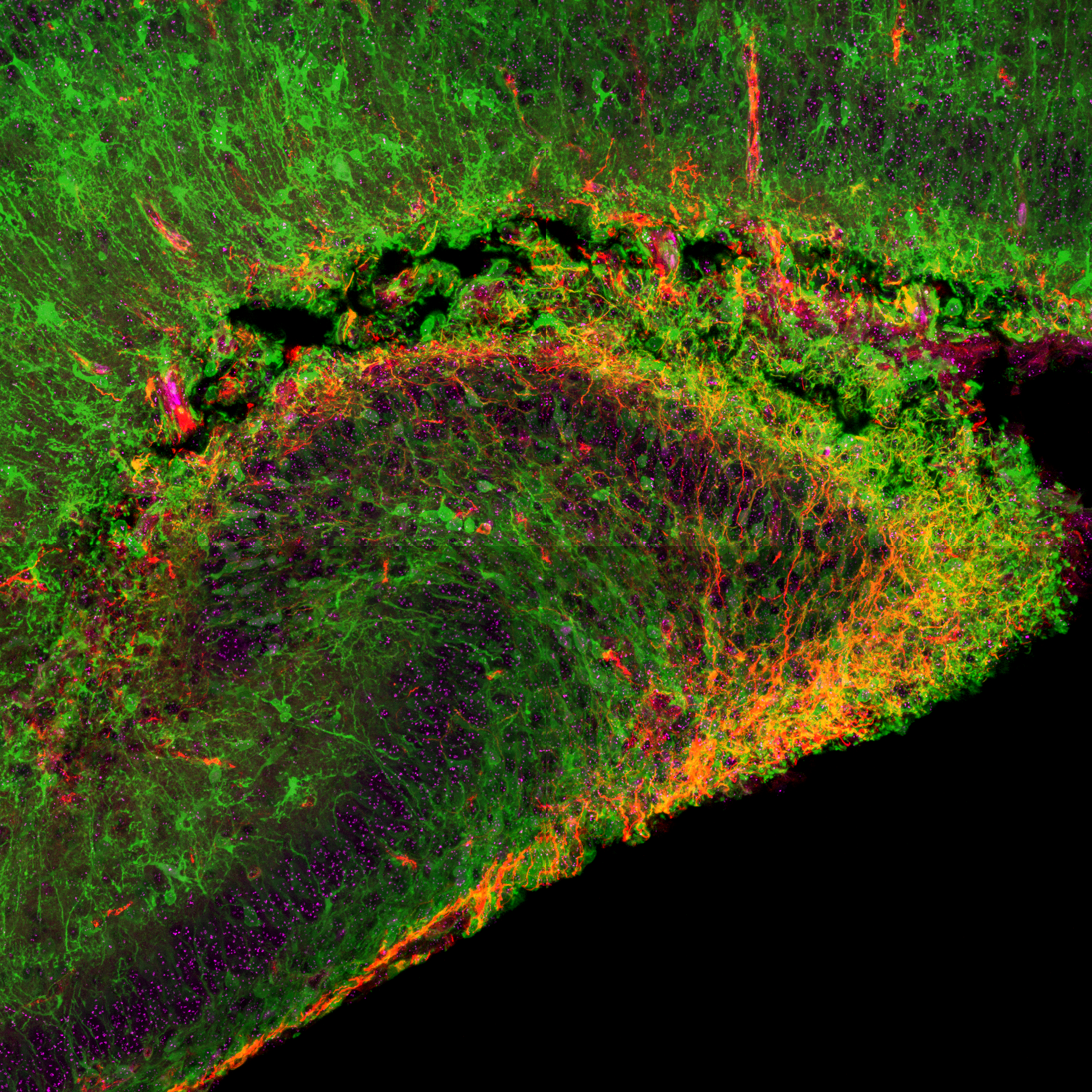

Supplement: Supplementary file 3 — Source Data Fig. 2 [file 44318_2023_11_MOESM3_ESM.zip › EMBOJ-2023-113564_SourceDataForFigure2/2B/P0_merge_replicate.png]

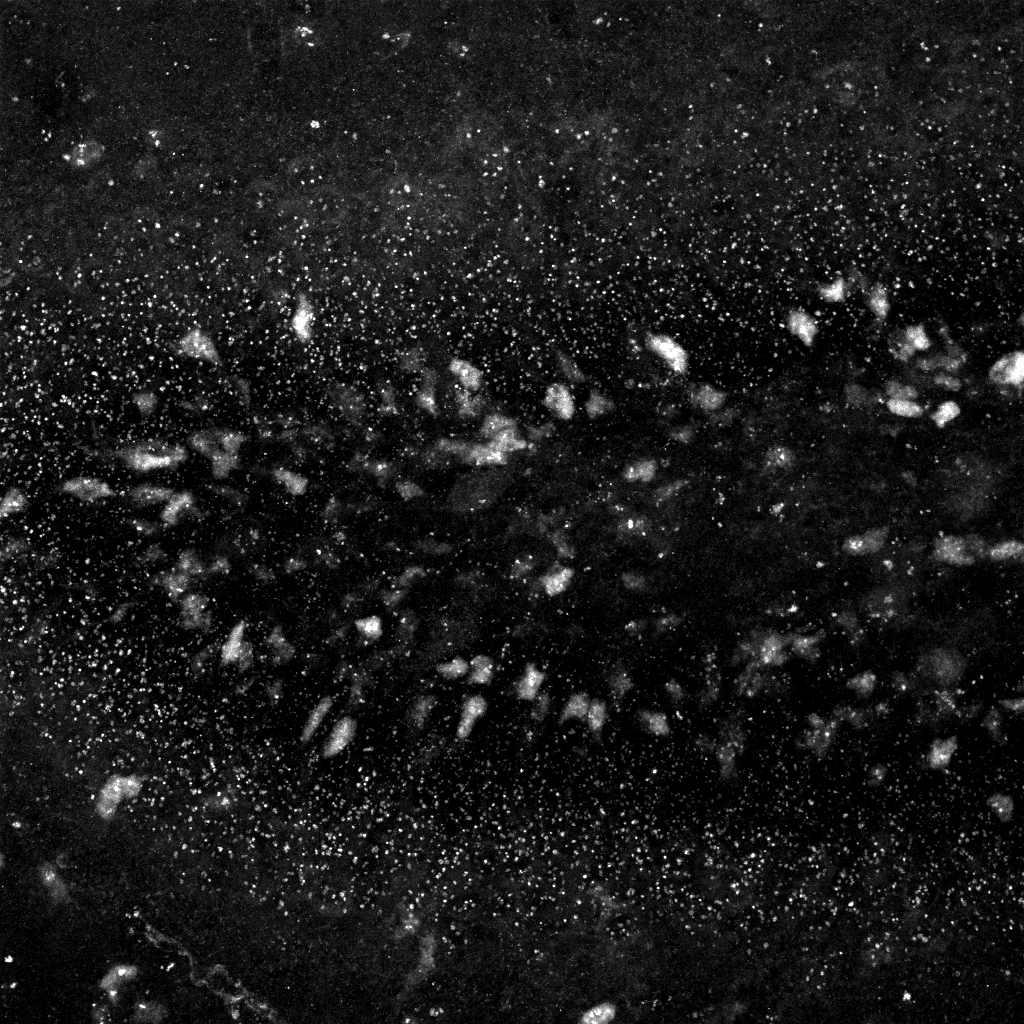

Supplement: Supplementary file 3 — Source Data Fig. 2 [file 44318_2023_11_MOESM3_ESM.zip › EMBOJ-2023-113564_SourceDataForFigure2/2B/P10_D2.png]

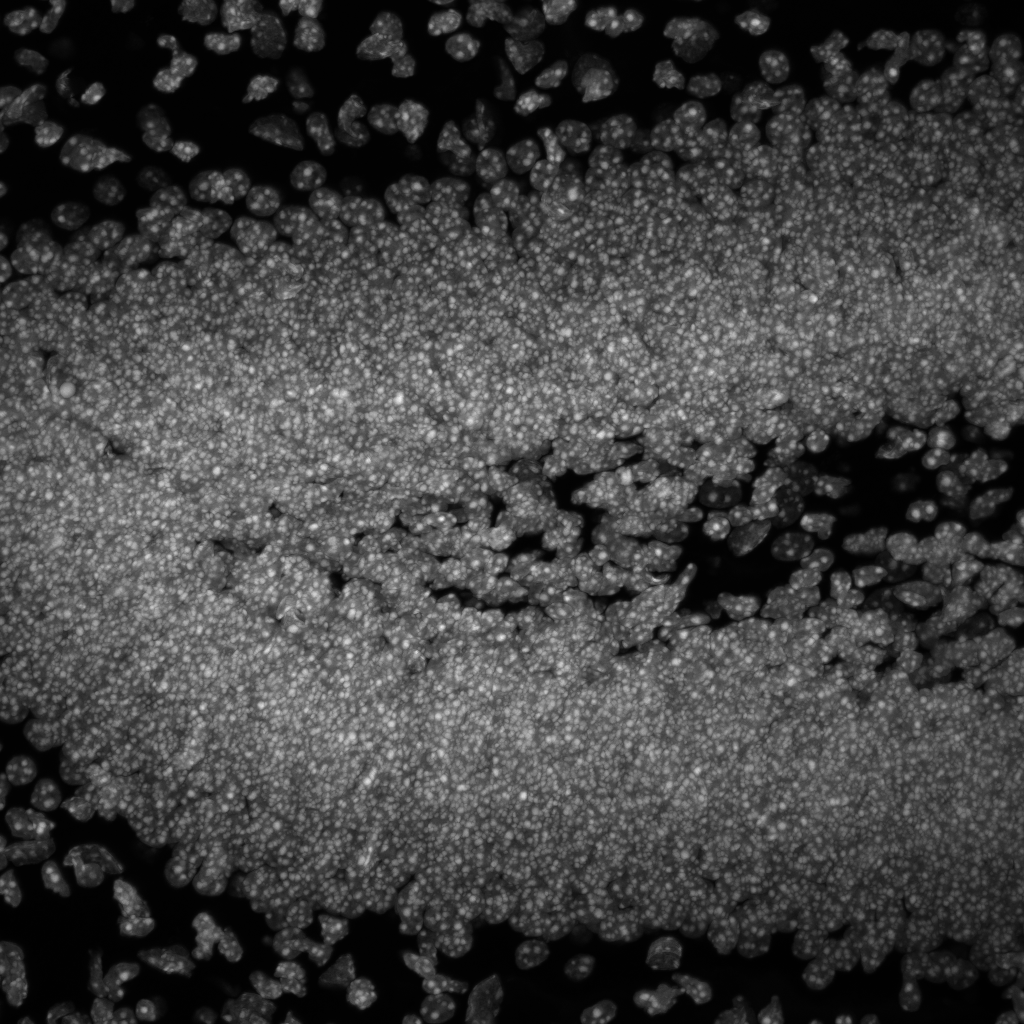

Supplement: Supplementary file 3 — Source Data Fig. 2 [file 44318_2023_11_MOESM3_ESM.zip › EMBOJ-2023-113564_SourceDataForFigure2/2B/P10_DAPI.png]

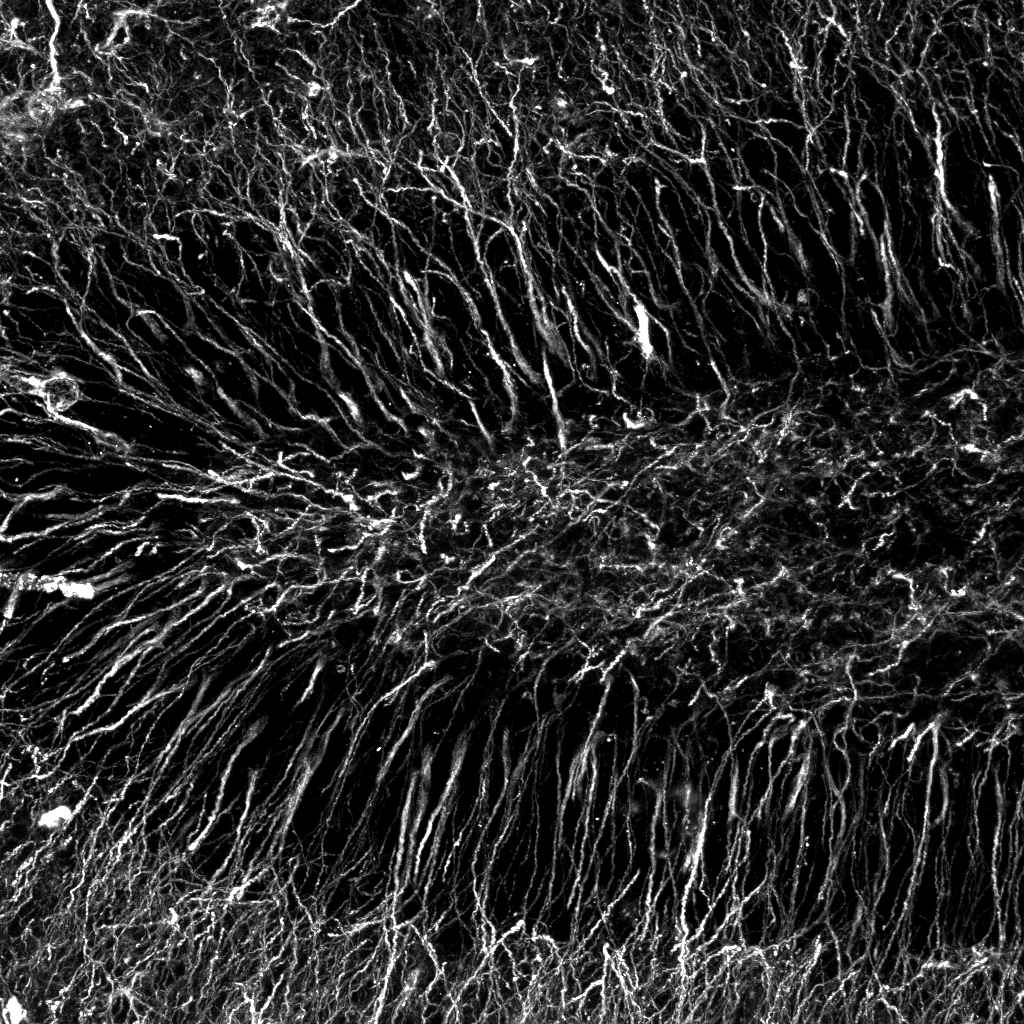

Supplement: Supplementary file 3 — Source Data Fig. 2 [file 44318_2023_11_MOESM3_ESM.zip › EMBOJ-2023-113564_SourceDataForFigure2/2B/P10_GFAP.png]

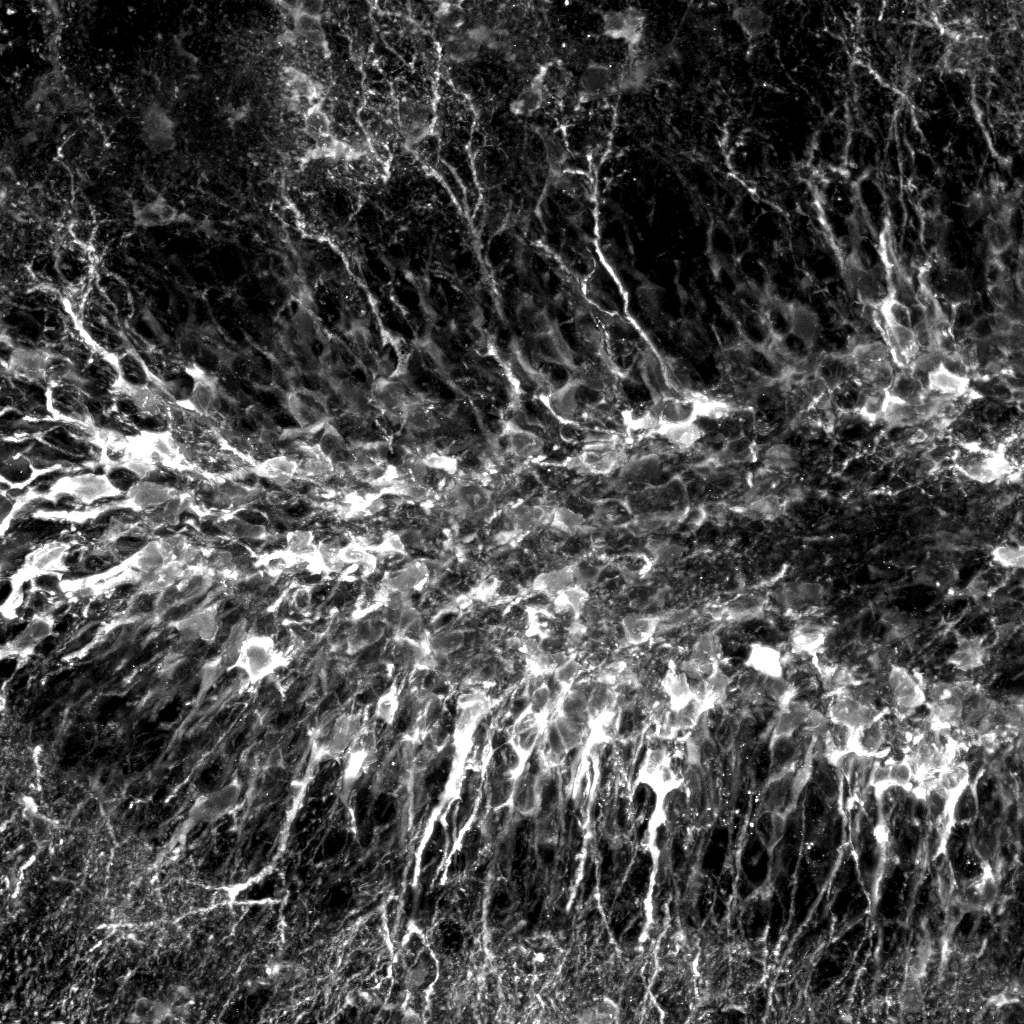

Supplement: Supplementary file 3 — Source Data Fig. 2 [file 44318_2023_11_MOESM3_ESM.zip › EMBOJ-2023-113564_SourceDataForFigure2/2B/P10_GFP.png]

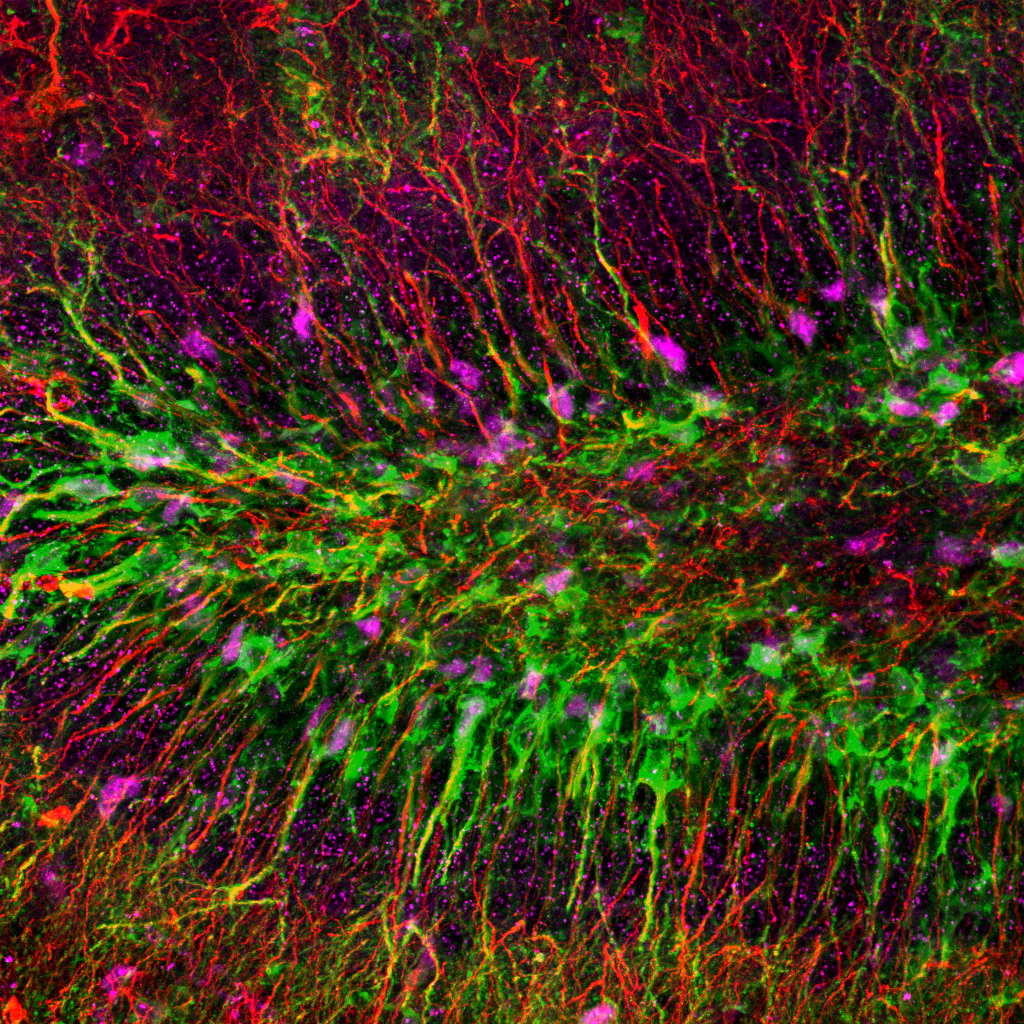

Supplement: Supplementary file 3 — Source Data Fig. 2 [file 44318_2023_11_MOESM3_ESM.zip › EMBOJ-2023-113564_SourceDataForFigure2/2B/P10_merge.png]

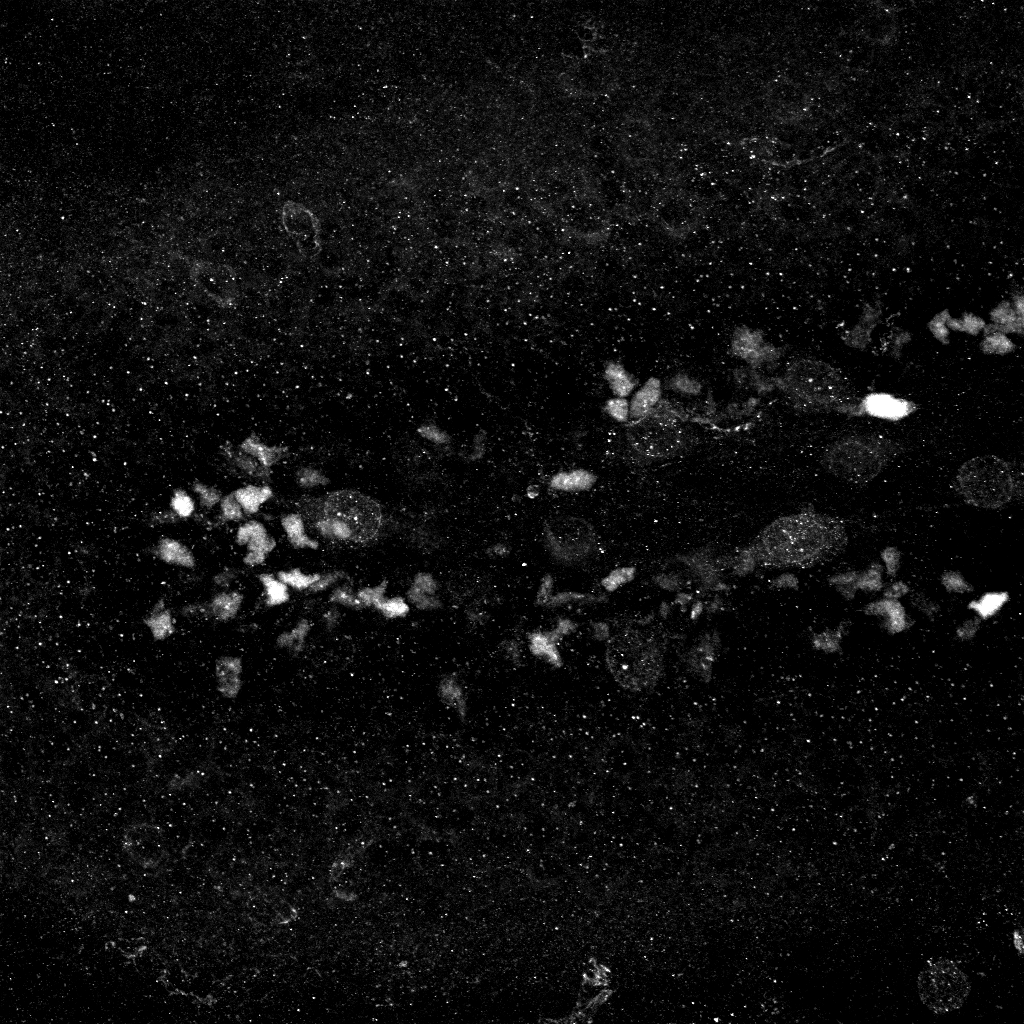

Supplement: Supplementary file 3 — Source Data Fig. 2 [file 44318_2023_11_MOESM3_ESM.zip › EMBOJ-2023-113564_SourceDataForFigure2/2B/P14_D2.png]

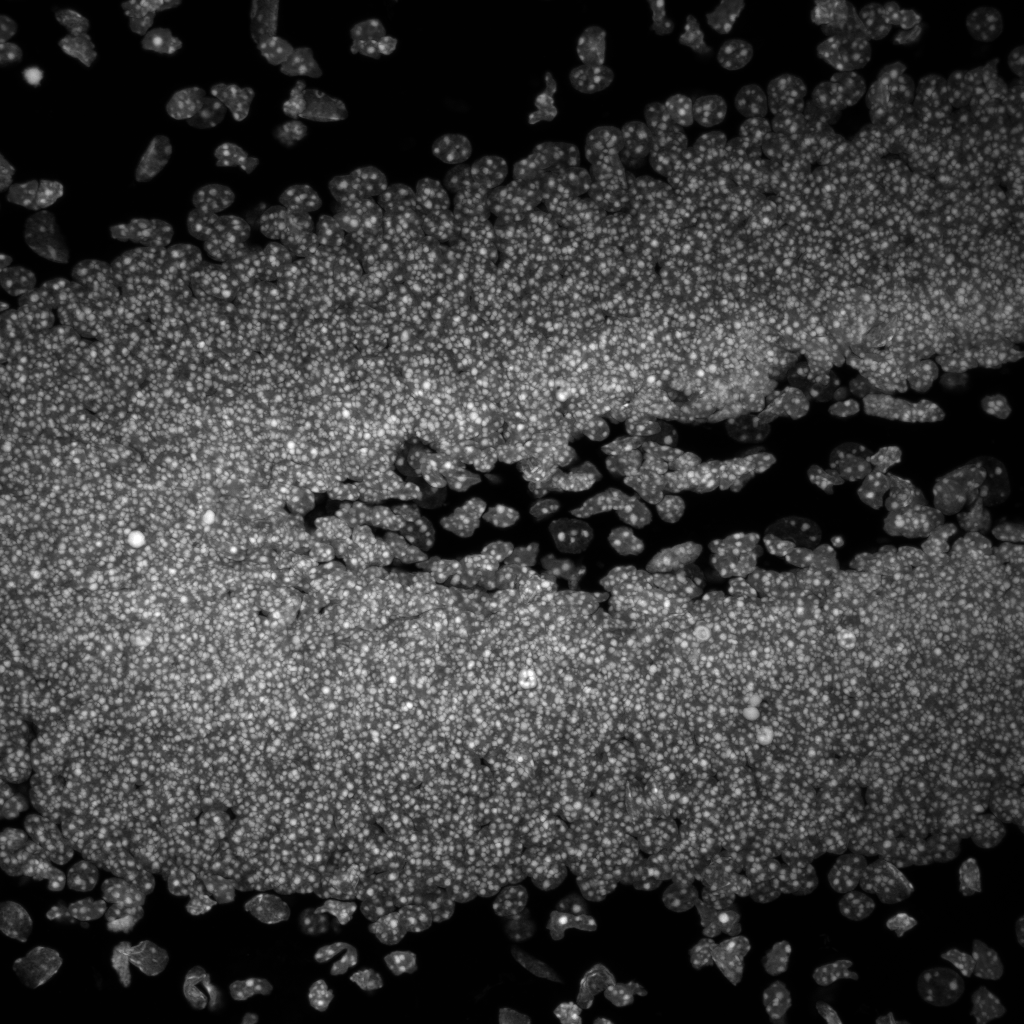

Supplement: Supplementary file 3 — Source Data Fig. 2 [file 44318_2023_11_MOESM3_ESM.zip › EMBOJ-2023-113564_SourceDataForFigure2/2B/P14_DAPI.png]

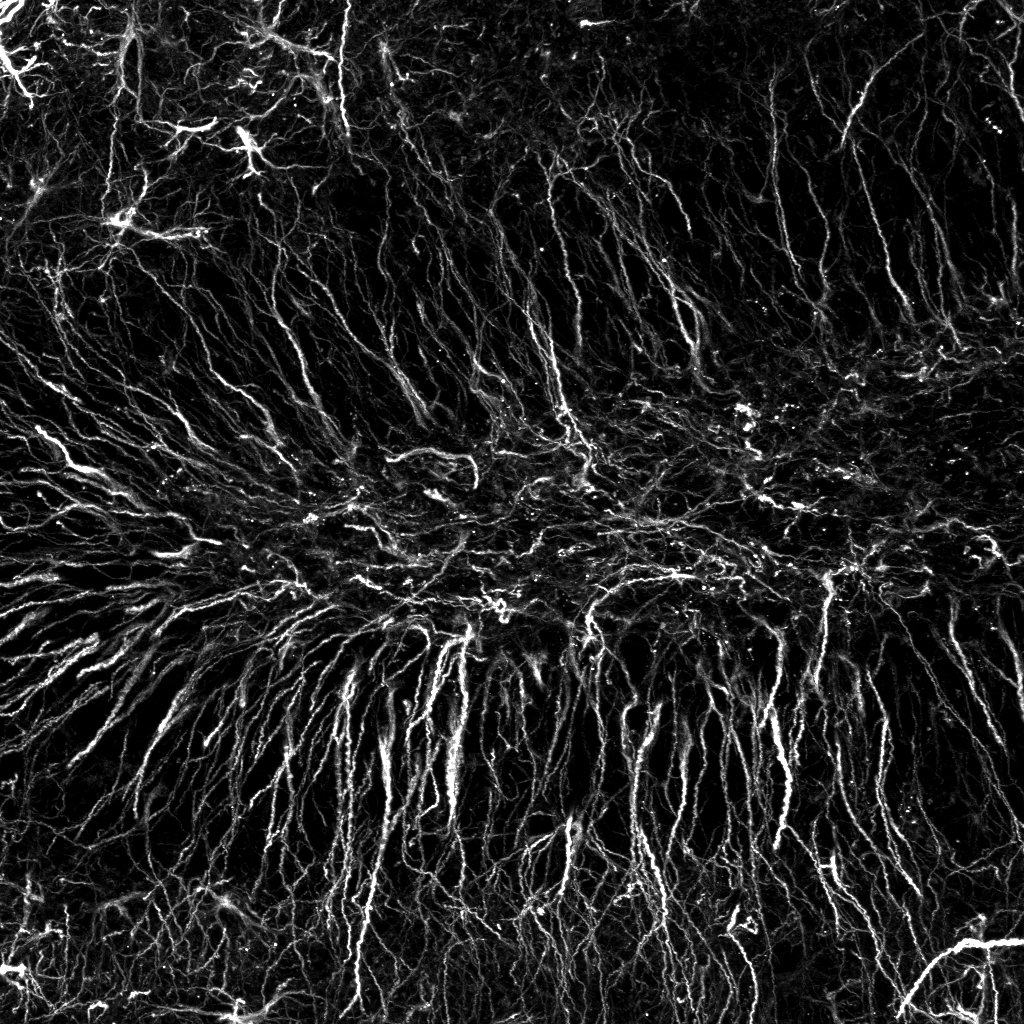

Supplement: Supplementary file 3 — Source Data Fig. 2 [file 44318_2023_11_MOESM3_ESM.zip › EMBOJ-2023-113564_SourceDataForFigure2/2B/P14_GFAP.png]

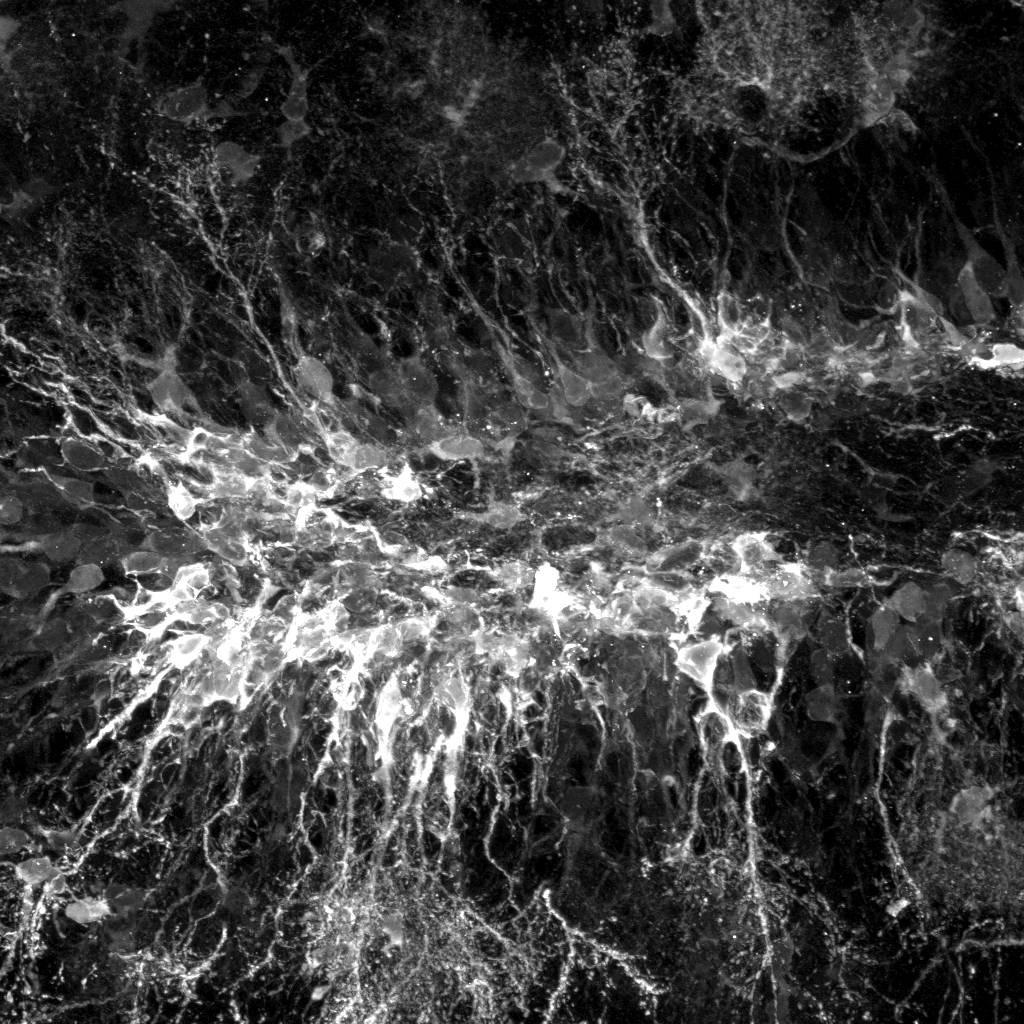

Supplement: Supplementary file 3 — Source Data Fig. 2 [file 44318_2023_11_MOESM3_ESM.zip › EMBOJ-2023-113564_SourceDataForFigure2/2B/P14_GFP.png]

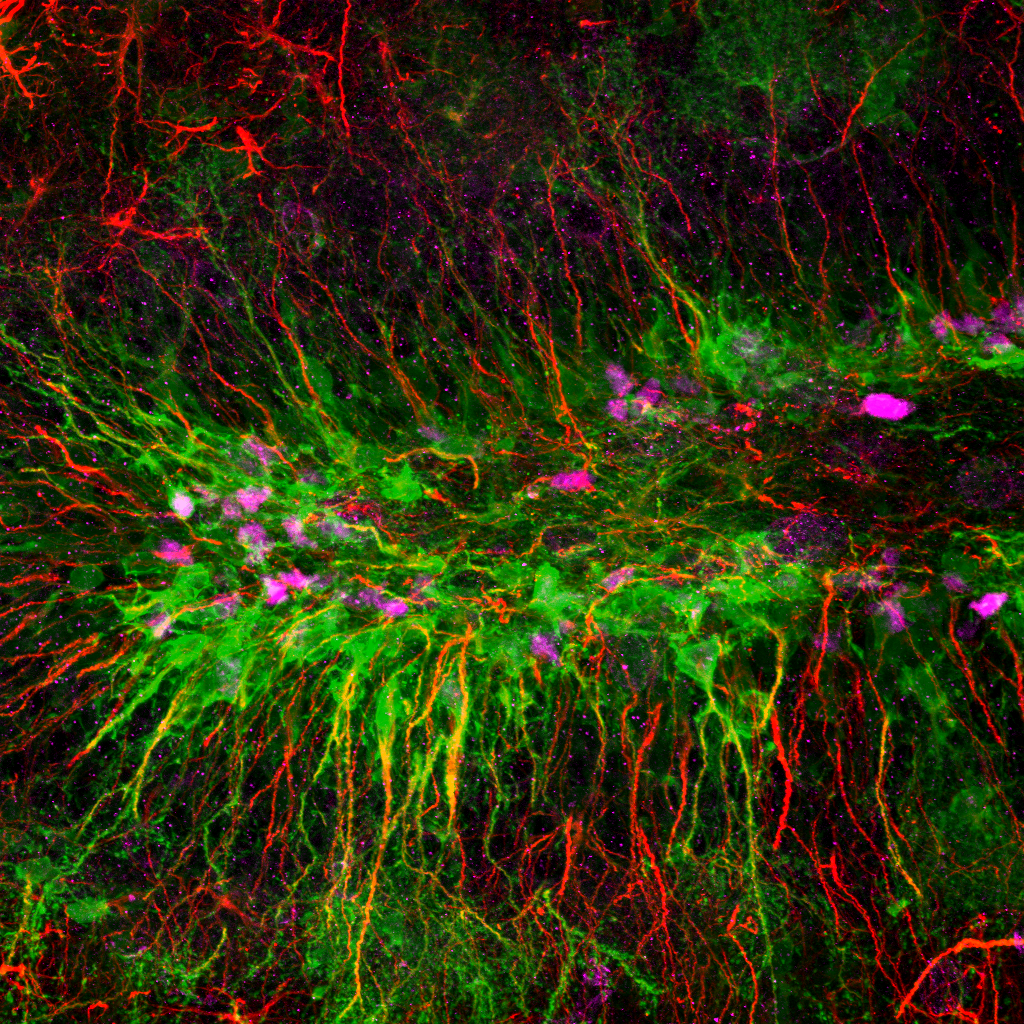

Supplement: Supplementary file 3 — Source Data Fig. 2 [file 44318_2023_11_MOESM3_ESM.zip › EMBOJ-2023-113564_SourceDataForFigure2/2B/P14_merge.png]

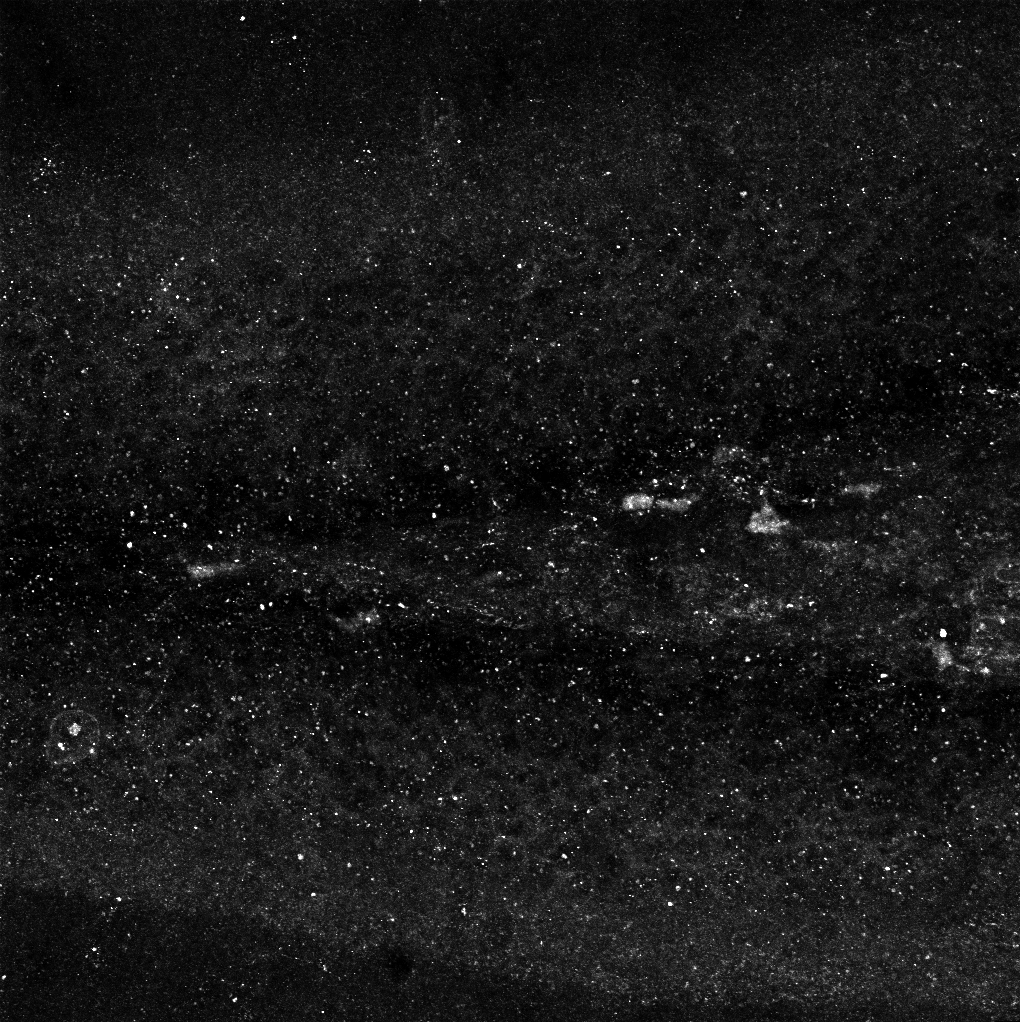

Supplement: Supplementary file 3 — Source Data Fig. 2 [file 44318_2023_11_MOESM3_ESM.zip › EMBOJ-2023-113564_SourceDataForFigure2/2B/P28_D2.png]

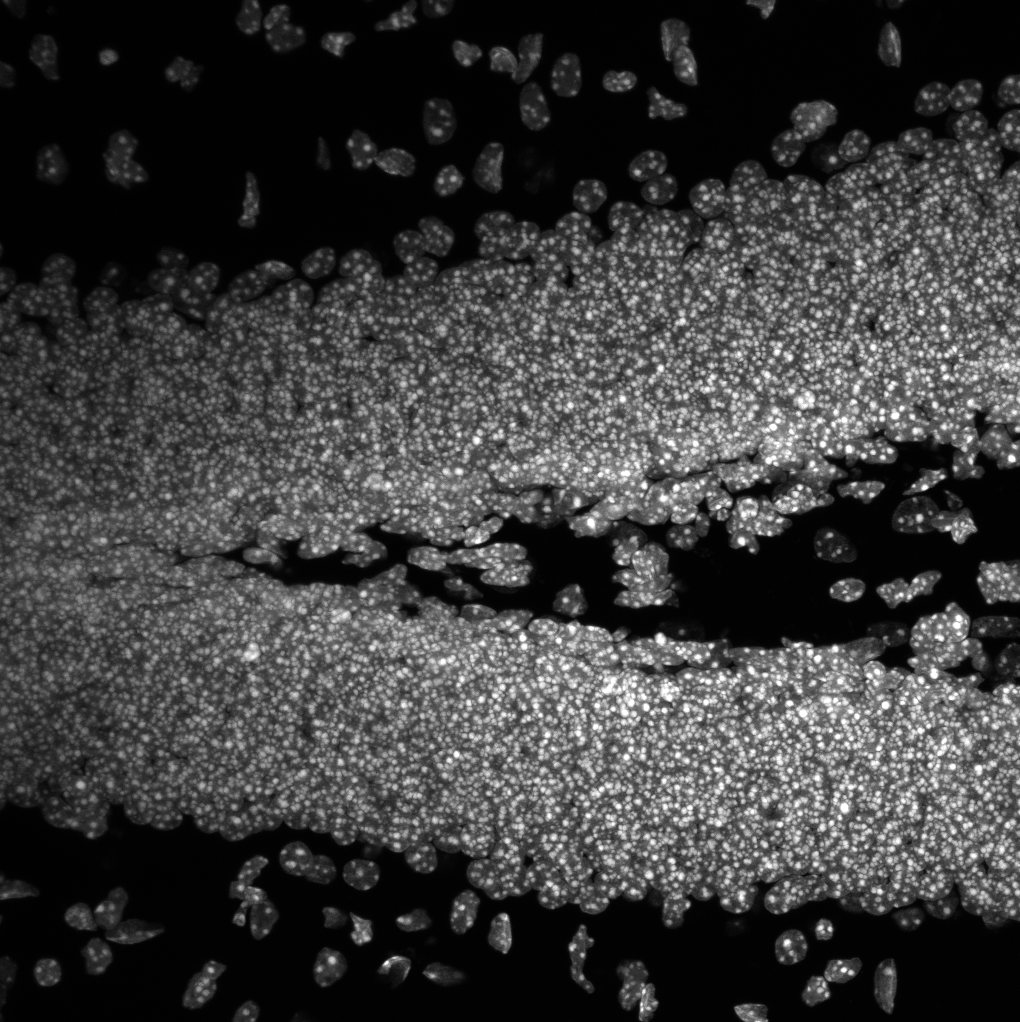

Supplement: Supplementary file 3 — Source Data Fig. 2 [file 44318_2023_11_MOESM3_ESM.zip › EMBOJ-2023-113564_SourceDataForFigure2/2B/P28_DAPI.png]

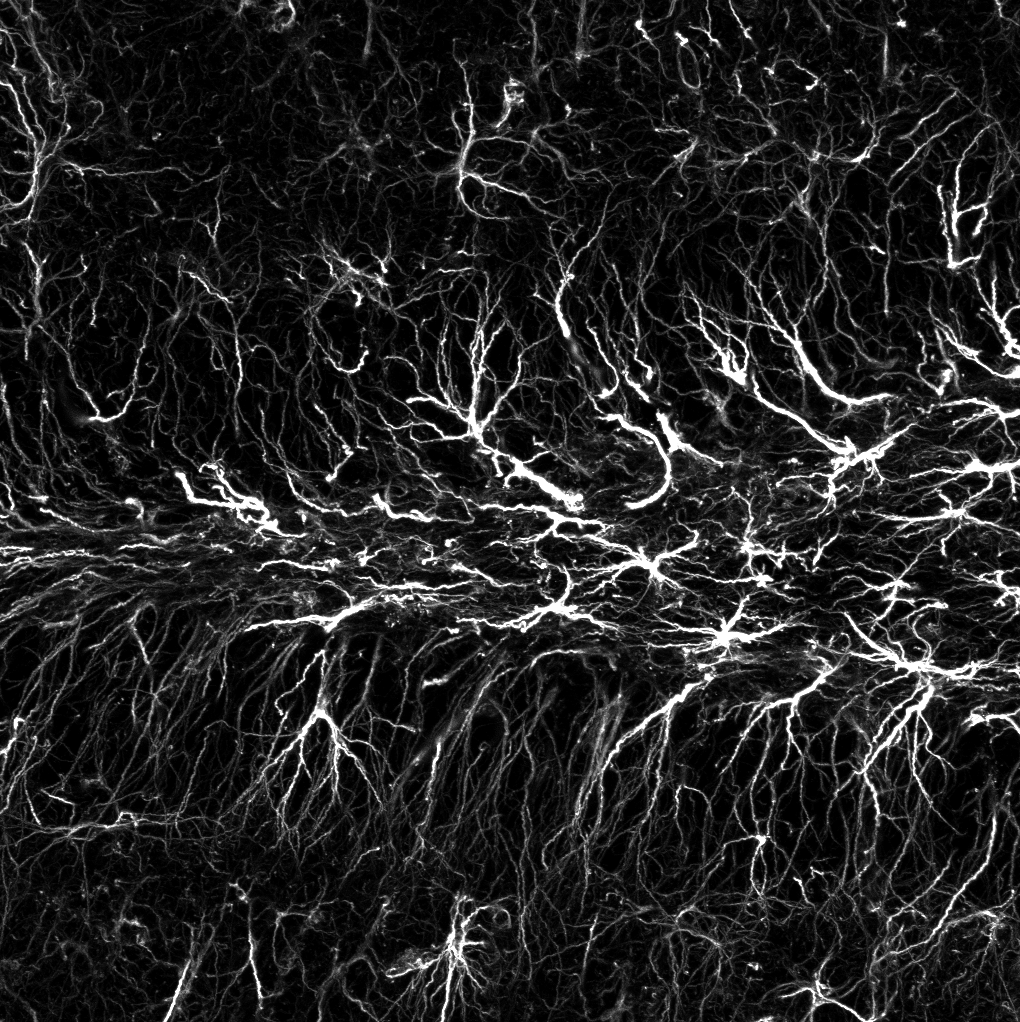

Supplement: Supplementary file 3 — Source Data Fig. 2 [file 44318_2023_11_MOESM3_ESM.zip › EMBOJ-2023-113564_SourceDataForFigure2/2B/P28_GFAP.png]

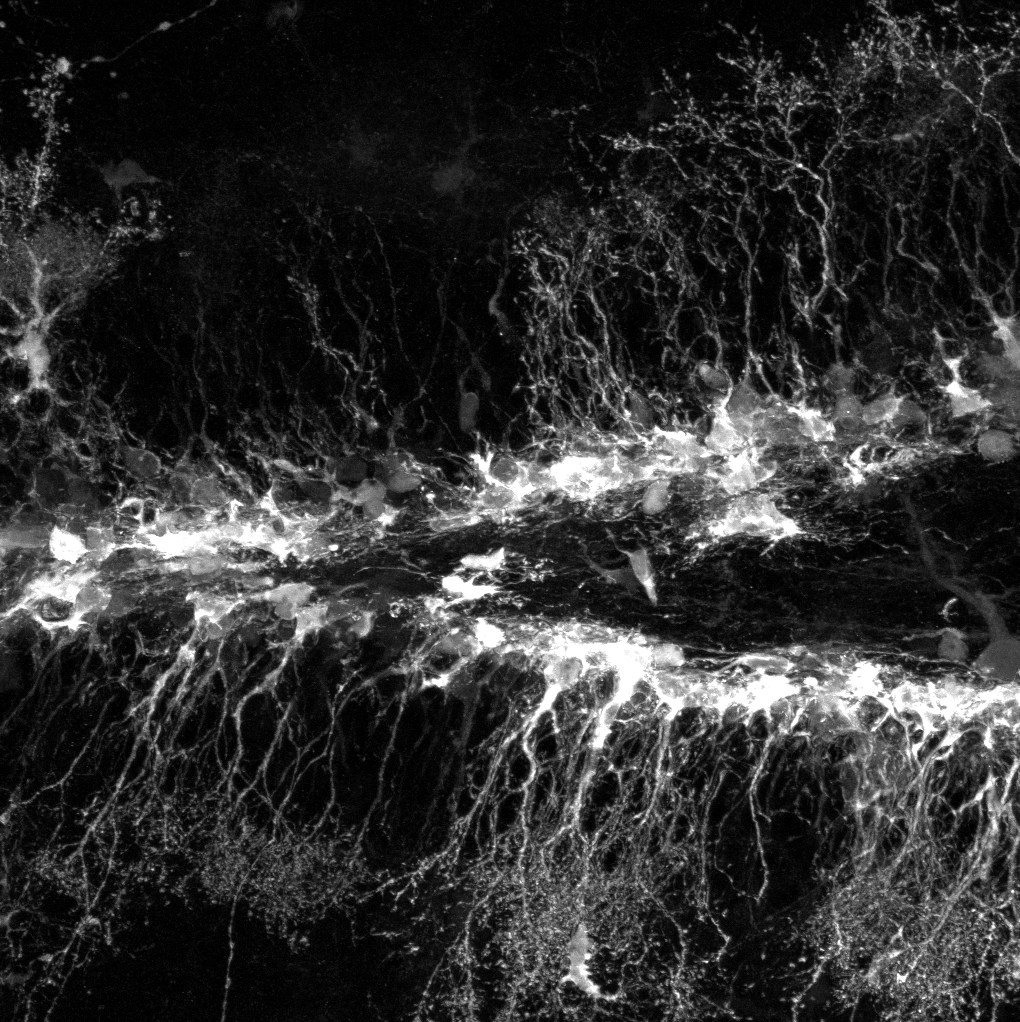

Supplement: Supplementary file 3 — Source Data Fig. 2 [file 44318_2023_11_MOESM3_ESM.zip › EMBOJ-2023-113564_SourceDataForFigure2/2B/P28_GFP.png]

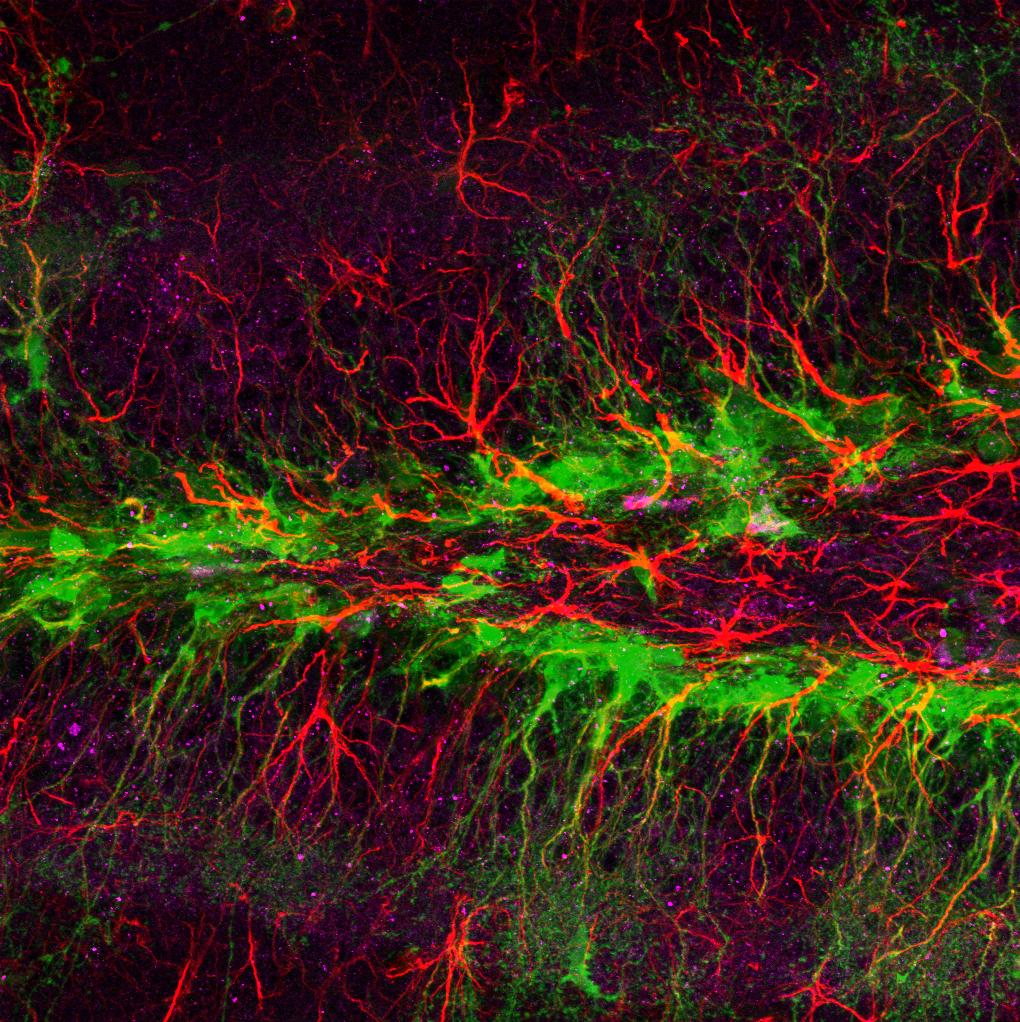

Supplement: Supplementary file 3 — Source Data Fig. 2 [file 44318_2023_11_MOESM3_ESM.zip › EMBOJ-2023-113564_SourceDataForFigure2/2B/P28_merge.png]

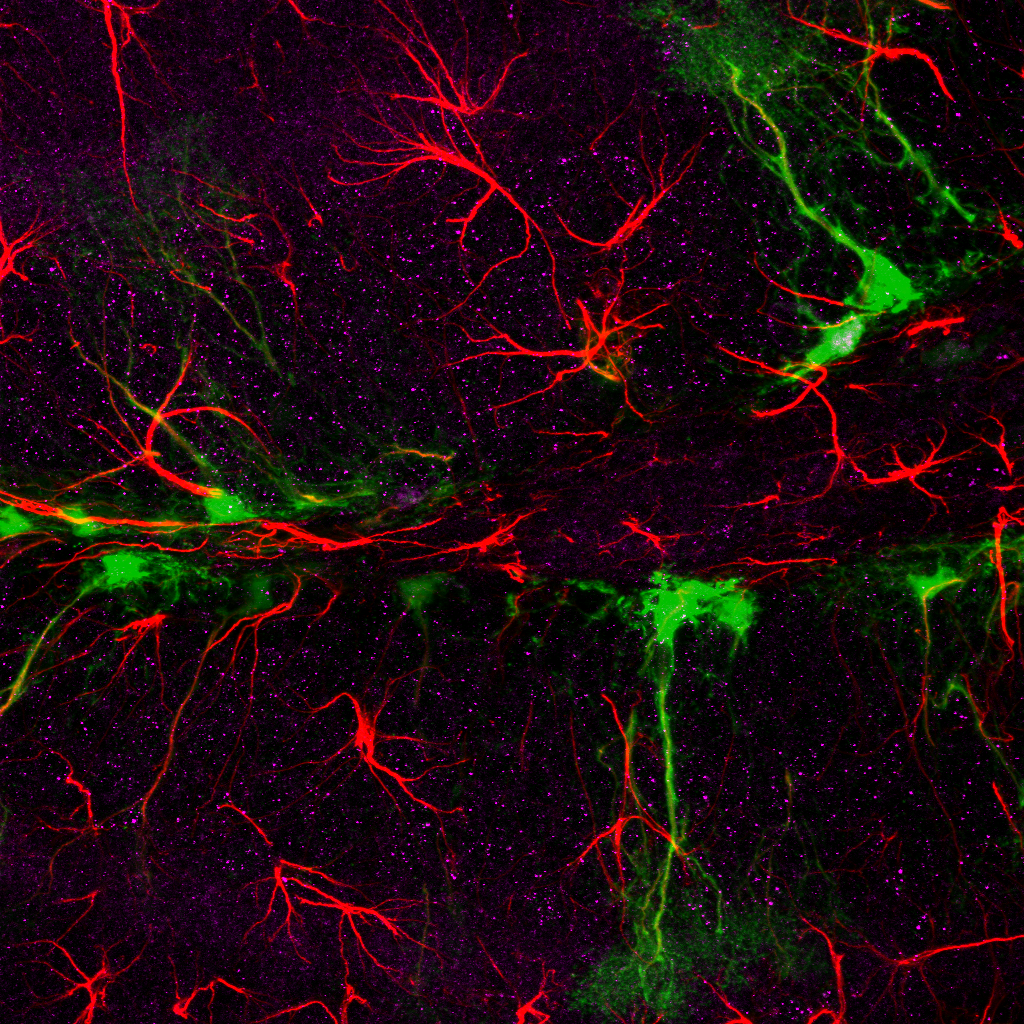

Supplement: Supplementary file 3 — Source Data Fig. 2 [file 44318_2023_11_MOESM3_ESM.zip › EMBOJ-2023-113564_SourceDataForFigure2/2B/P76_merge.png]

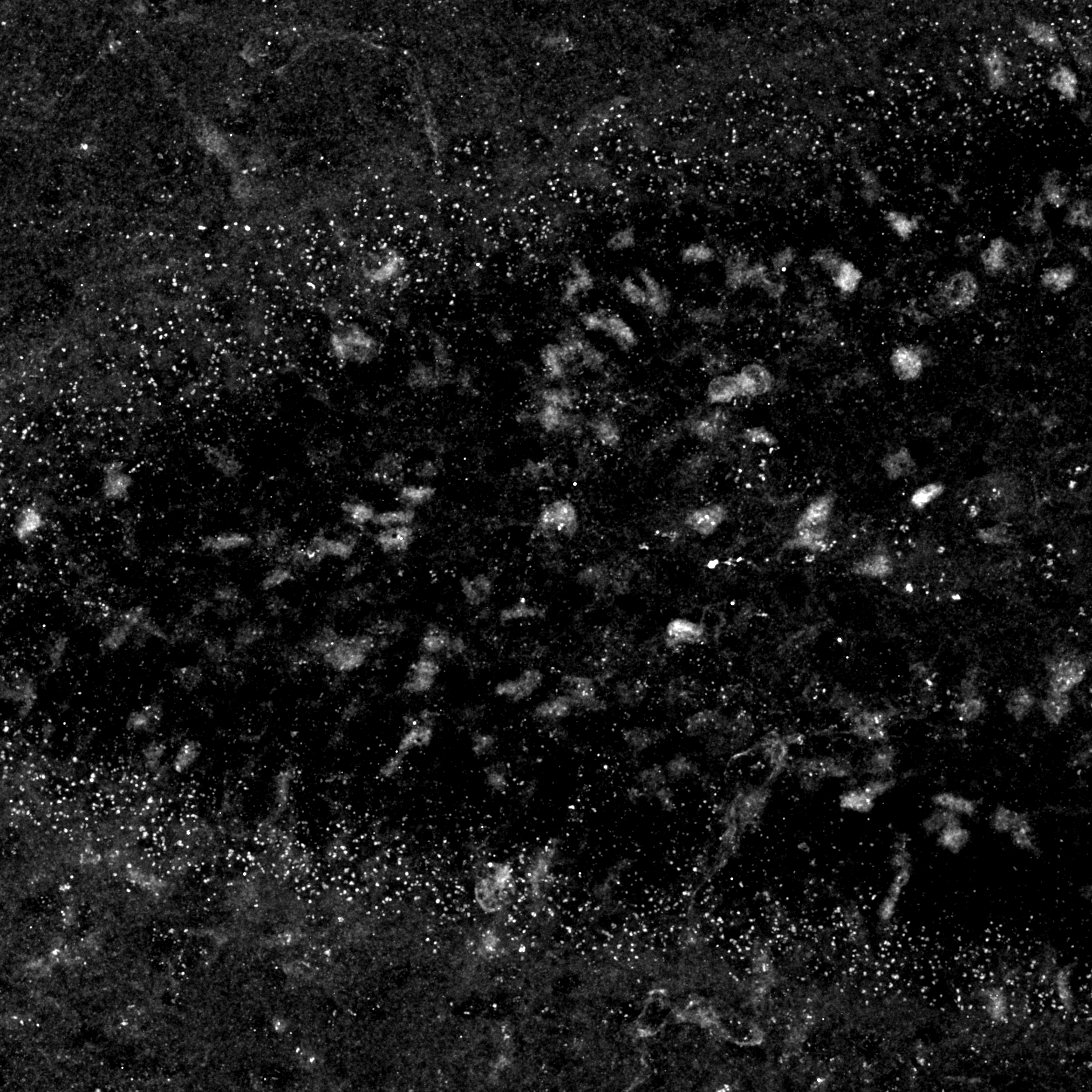

Supplement: Supplementary file 3 — Source Data Fig. 2 [file 44318_2023_11_MOESM3_ESM.zip › EMBOJ-2023-113564_SourceDataForFigure2/2B/P7_D2.png]

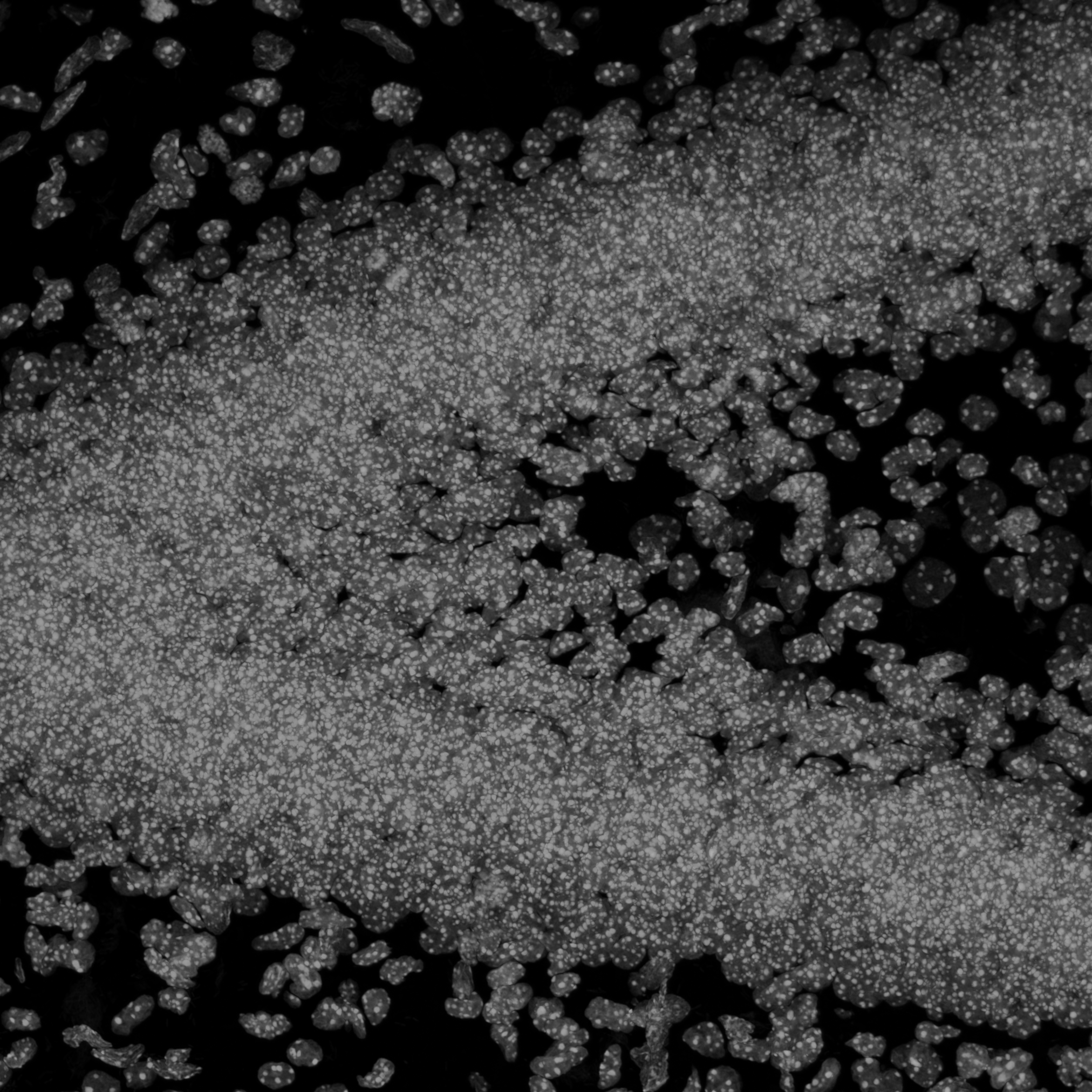

Supplement: Supplementary file 3 — Source Data Fig. 2 [file 44318_2023_11_MOESM3_ESM.zip › EMBOJ-2023-113564_SourceDataForFigure2/2B/P7_DAPI.png]

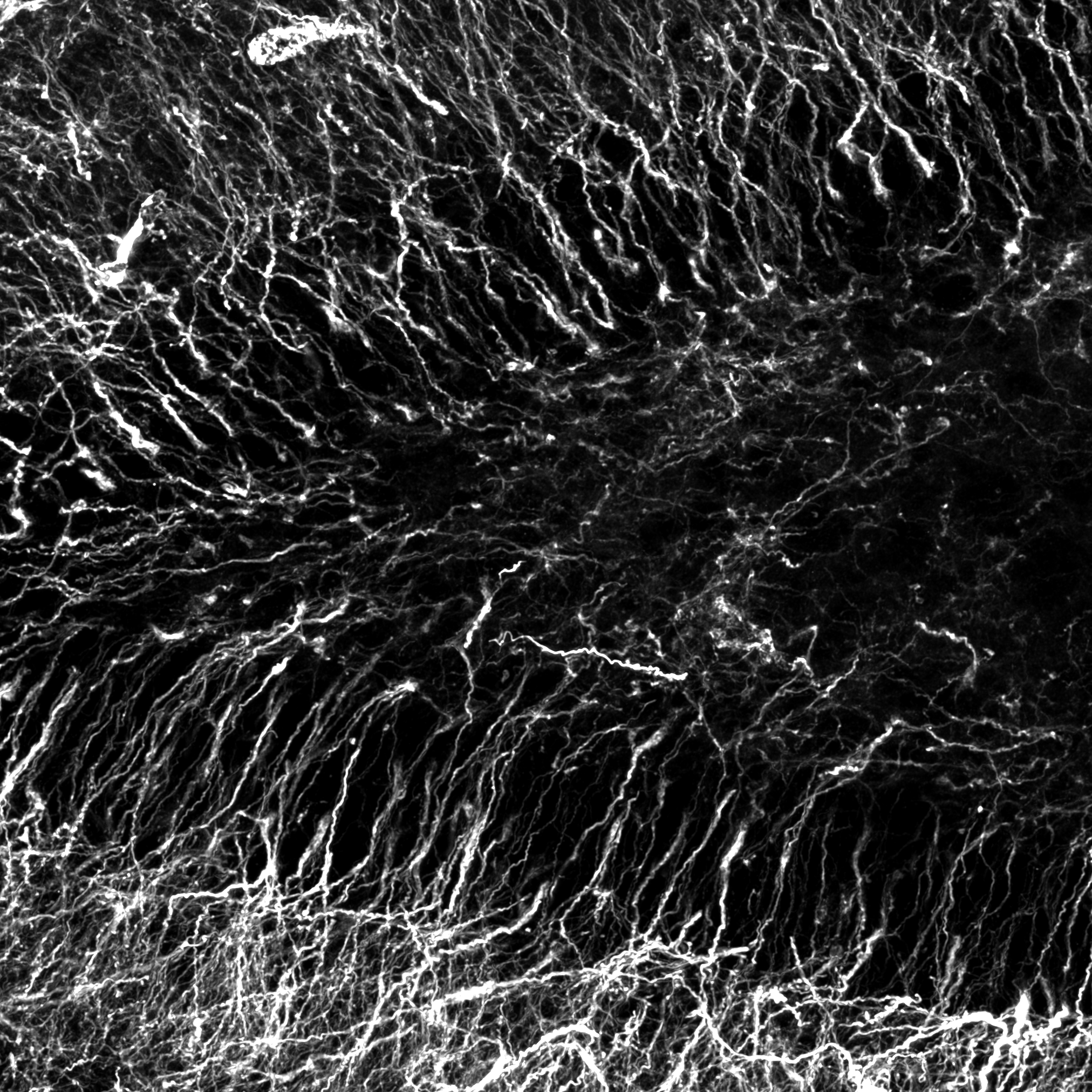

Supplement: Supplementary file 3 — Source Data Fig. 2 [file 44318_2023_11_MOESM3_ESM.zip › EMBOJ-2023-113564_SourceDataForFigure2/2B/P7_GFAP.png]

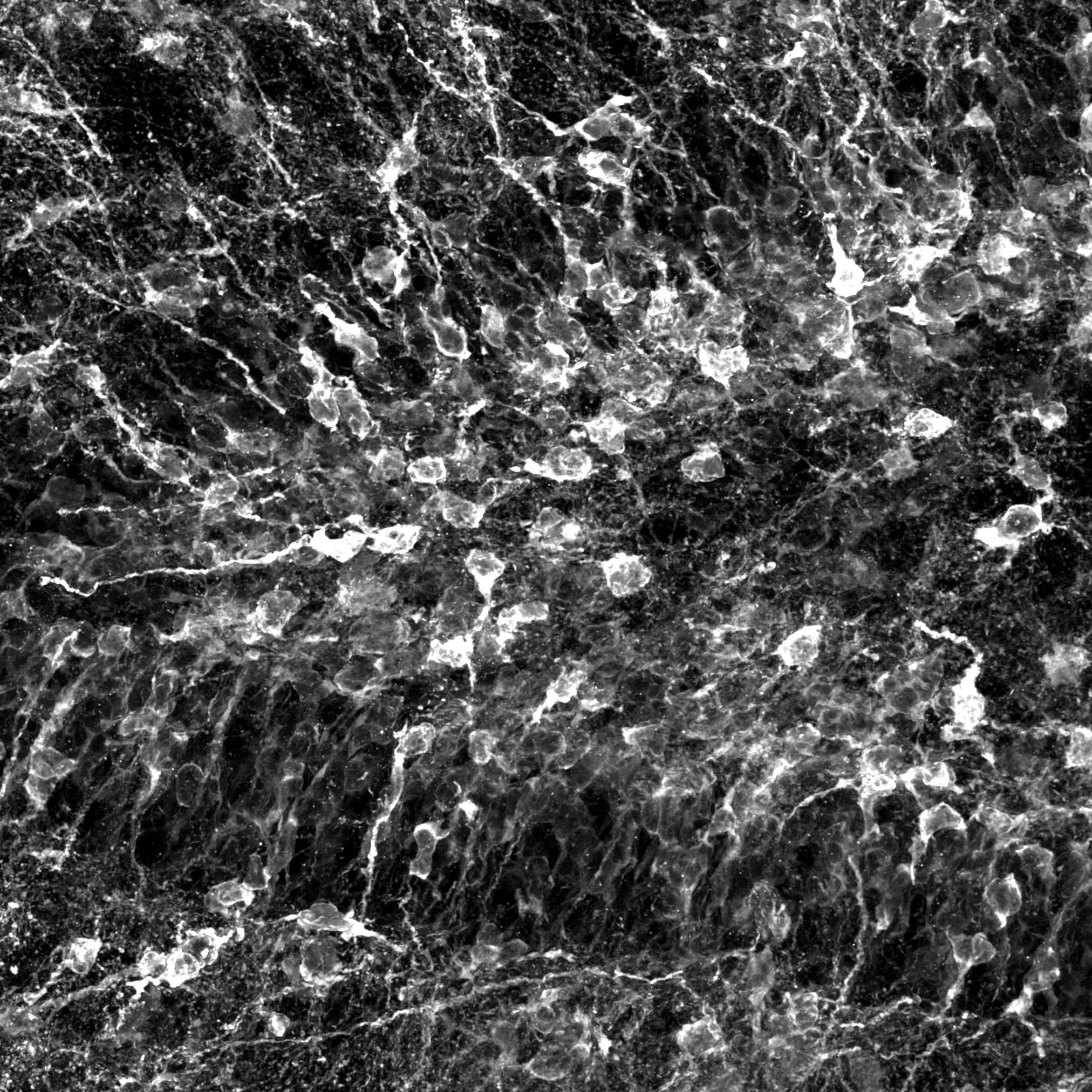

Supplement: Supplementary file 3 — Source Data Fig. 2 [file 44318_2023_11_MOESM3_ESM.zip › EMBOJ-2023-113564_SourceDataForFigure2/2B/P7_GFP.png]

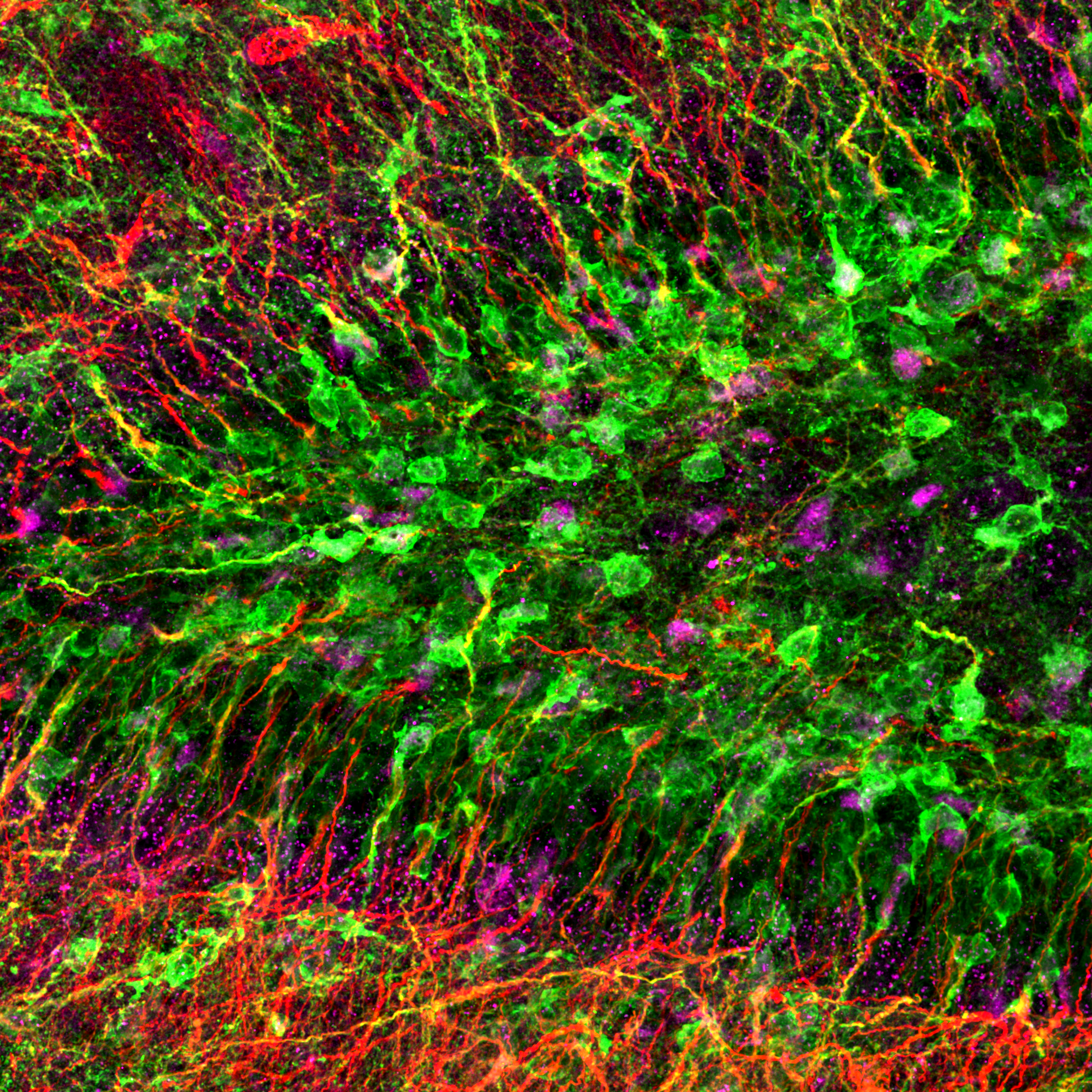

Supplement: Supplementary file 3 — Source Data Fig. 2 [file 44318_2023_11_MOESM3_ESM.zip › EMBOJ-2023-113564_SourceDataForFigure2/2B/P7_merge.png]

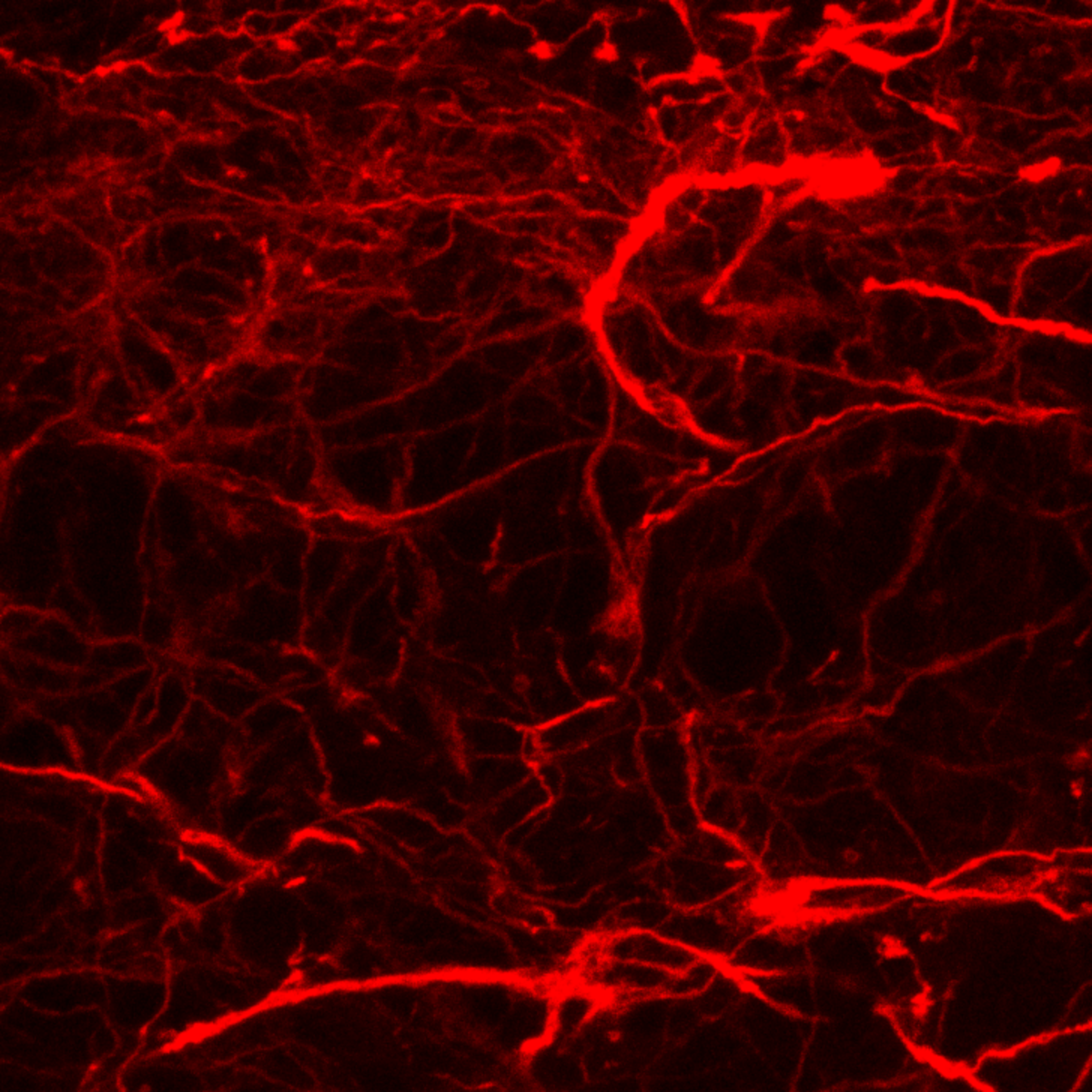

Supplement: Supplementary file 4 — Source Data Fig. 3 [file 44318_2023_11_MOESM4_ESM.zip › EMBOJ-2023-113564_SourceDataForFigure3/3A/P0/CloseUp KO P0 GCL_GFAP.tiff]

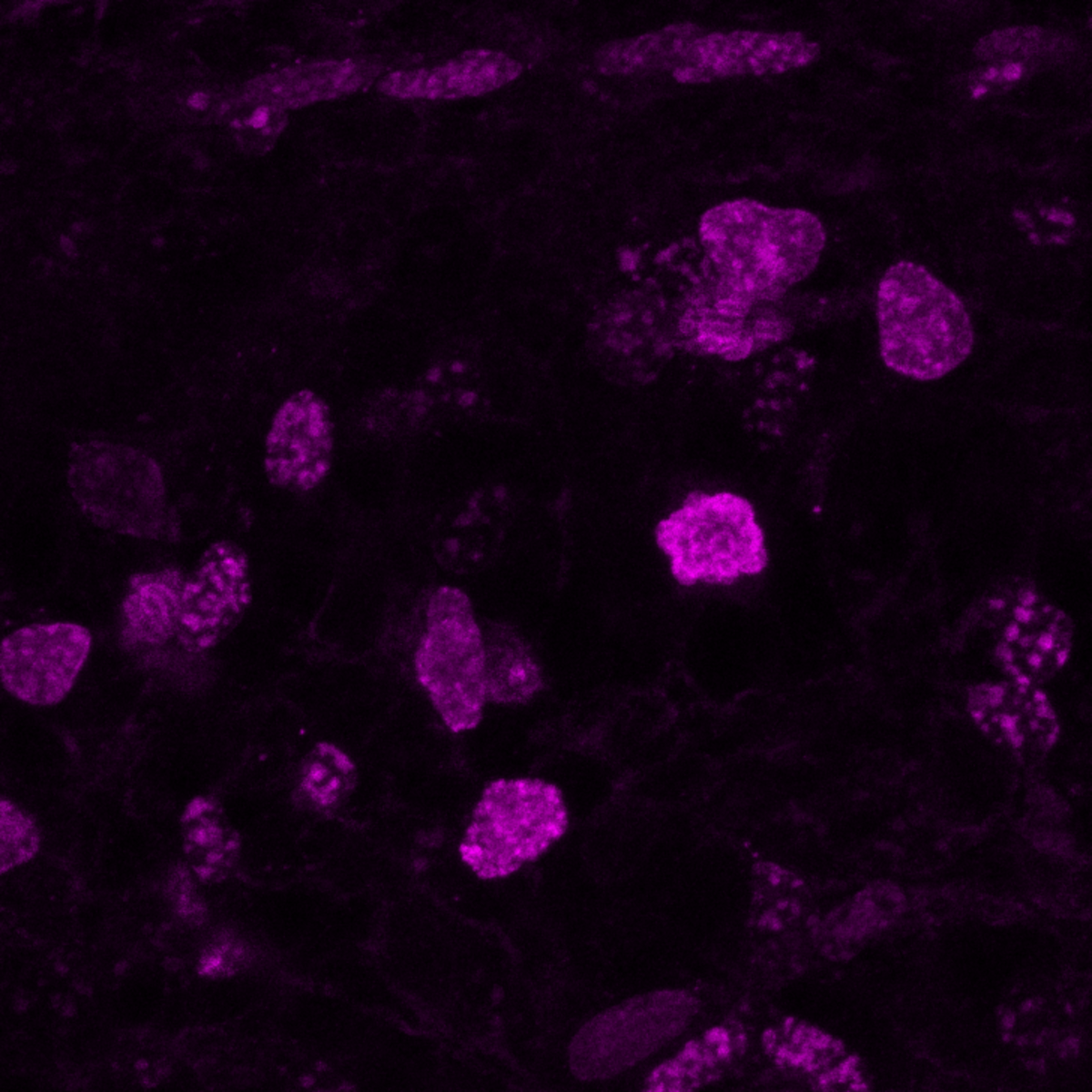

Supplement: Supplementary file 4 — Source Data Fig. 3 [file 44318_2023_11_MOESM4_ESM.zip › EMBOJ-2023-113564_SourceDataForFigure3/3A/P0/CloseUp KO P0 GCL_Ki67.tiff]

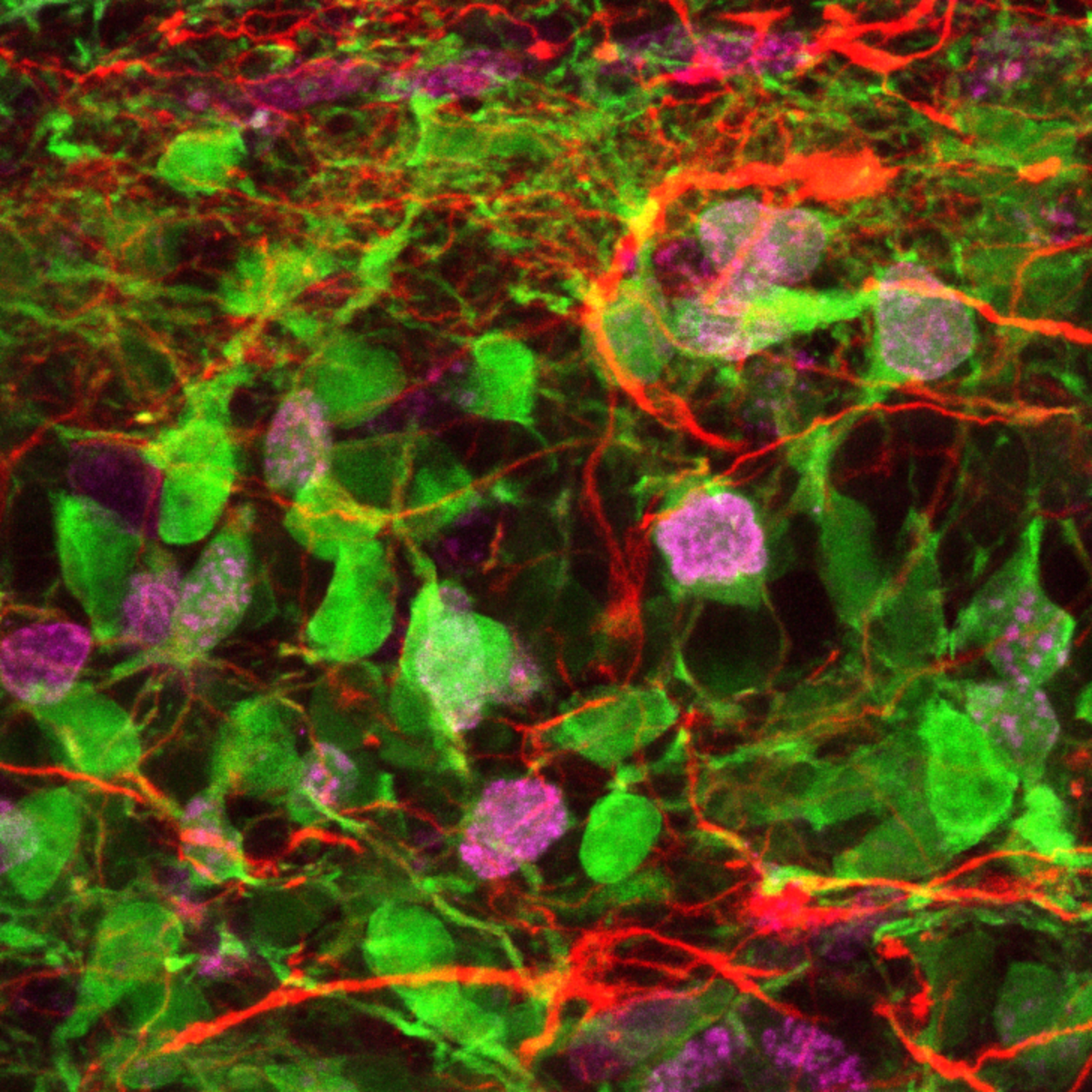

Supplement: Supplementary file 4 — Source Data Fig. 3 [file 44318_2023_11_MOESM4_ESM.zip › EMBOJ-2023-113564_SourceDataForFigure3/3A/P0/CloseUp KO P0 GCL_Merge.tiff]

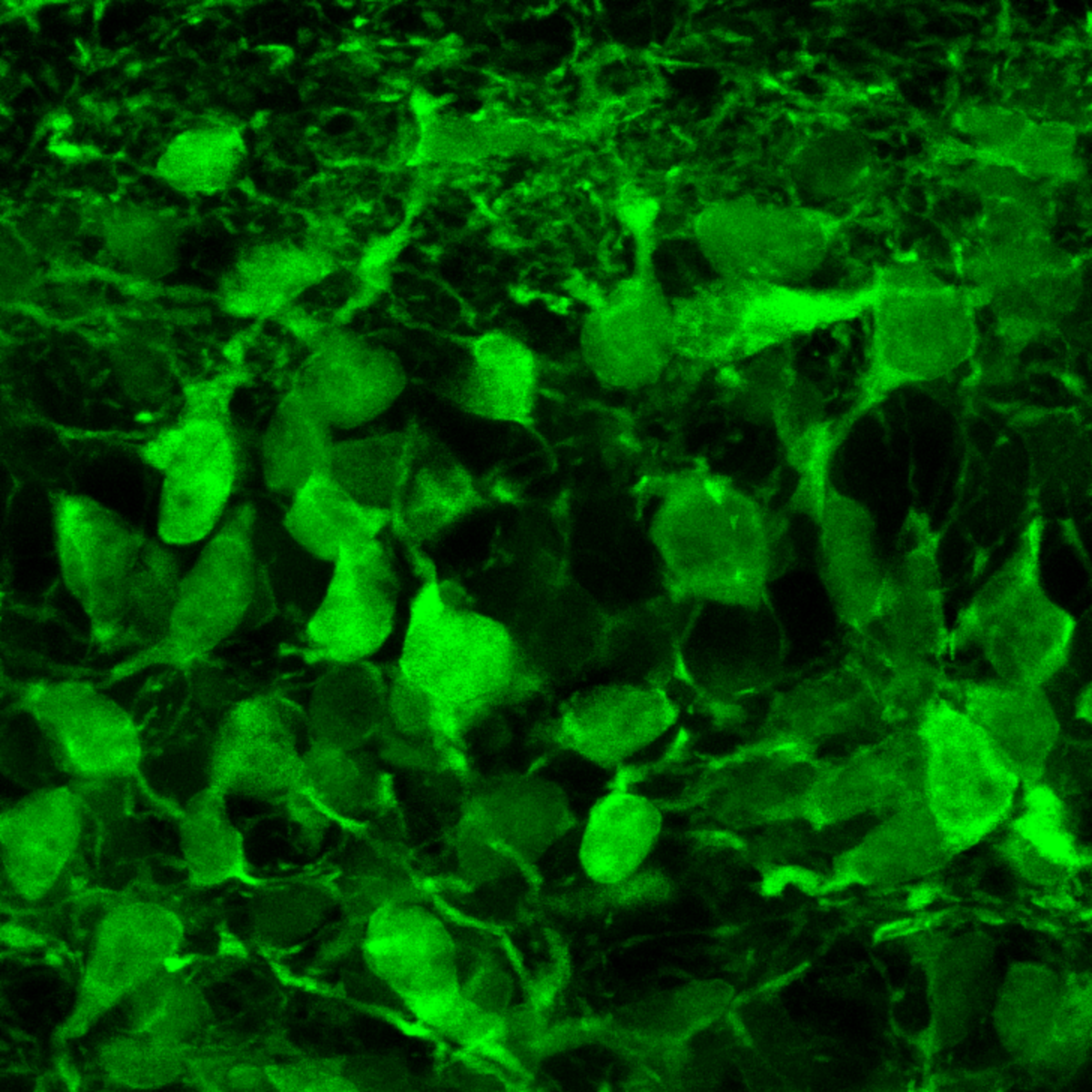

Supplement: Supplementary file 4 — Source Data Fig. 3 [file 44318_2023_11_MOESM4_ESM.zip › EMBOJ-2023-113564_SourceDataForFigure3/3A/P0/CloseUp KO P0 GCL_Nestin.tiff]

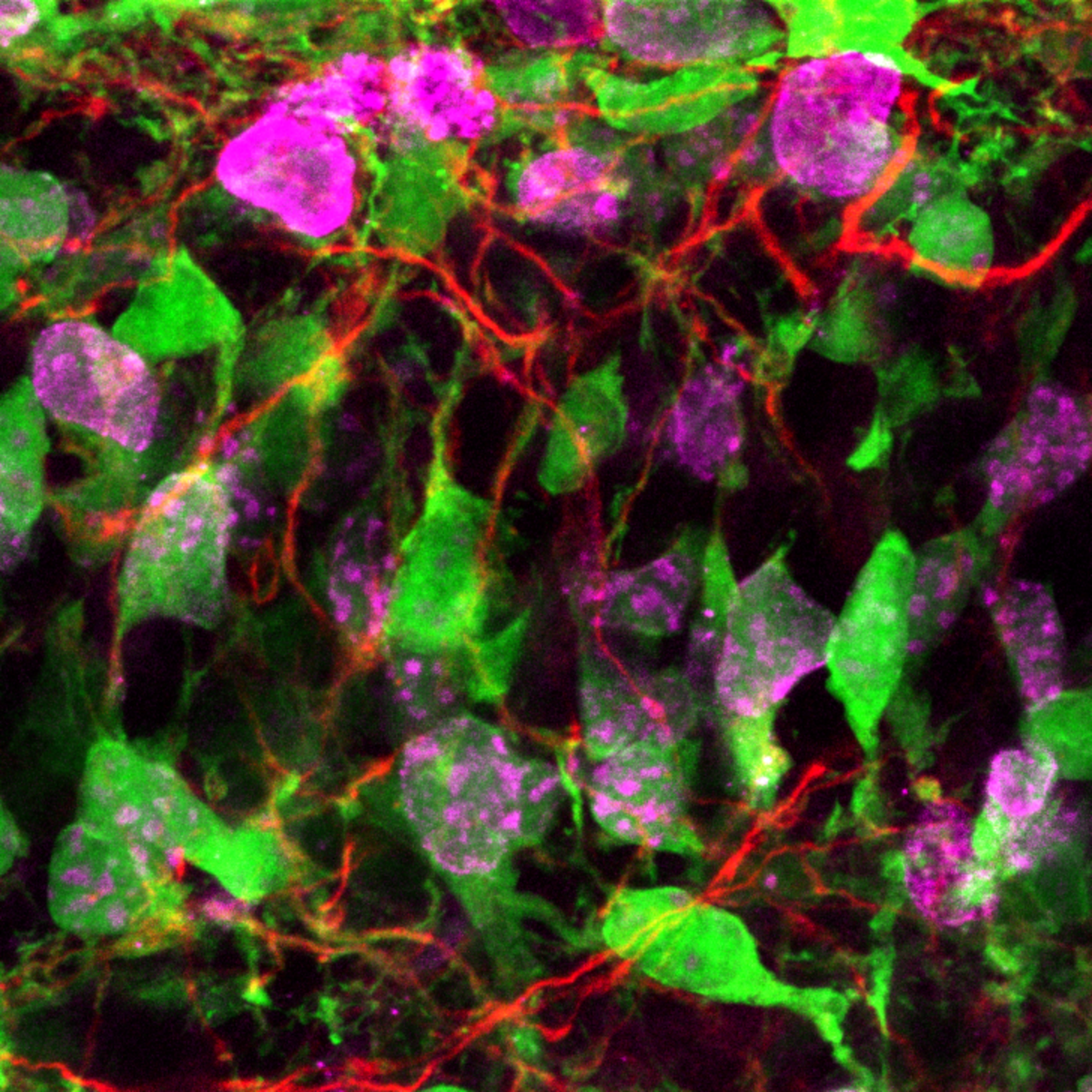

Supplement: Supplementary file 4 — Source Data Fig. 3 [file 44318_2023_11_MOESM4_ESM.zip › EMBOJ-2023-113564_SourceDataForFigure3/3A/P0/CloseUp WT P0 GCL_Merge.tiff]

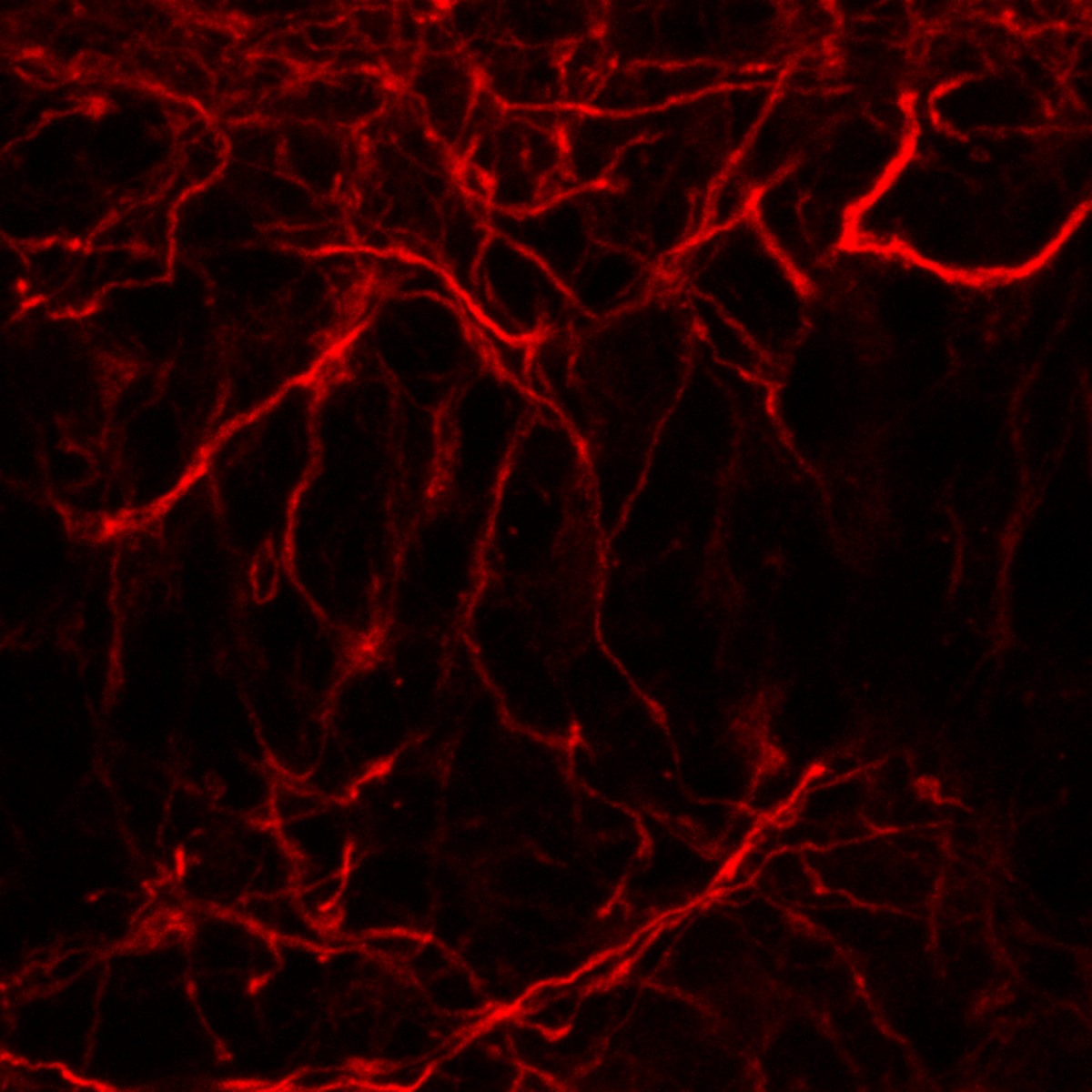

Supplement: Supplementary file 4 — Source Data Fig. 3 [file 44318_2023_11_MOESM4_ESM.zip › EMBOJ-2023-113564_SourceDataForFigure3/3A/P0/CloseUp WT P0 GCL_Merge_GFAP.tiff]

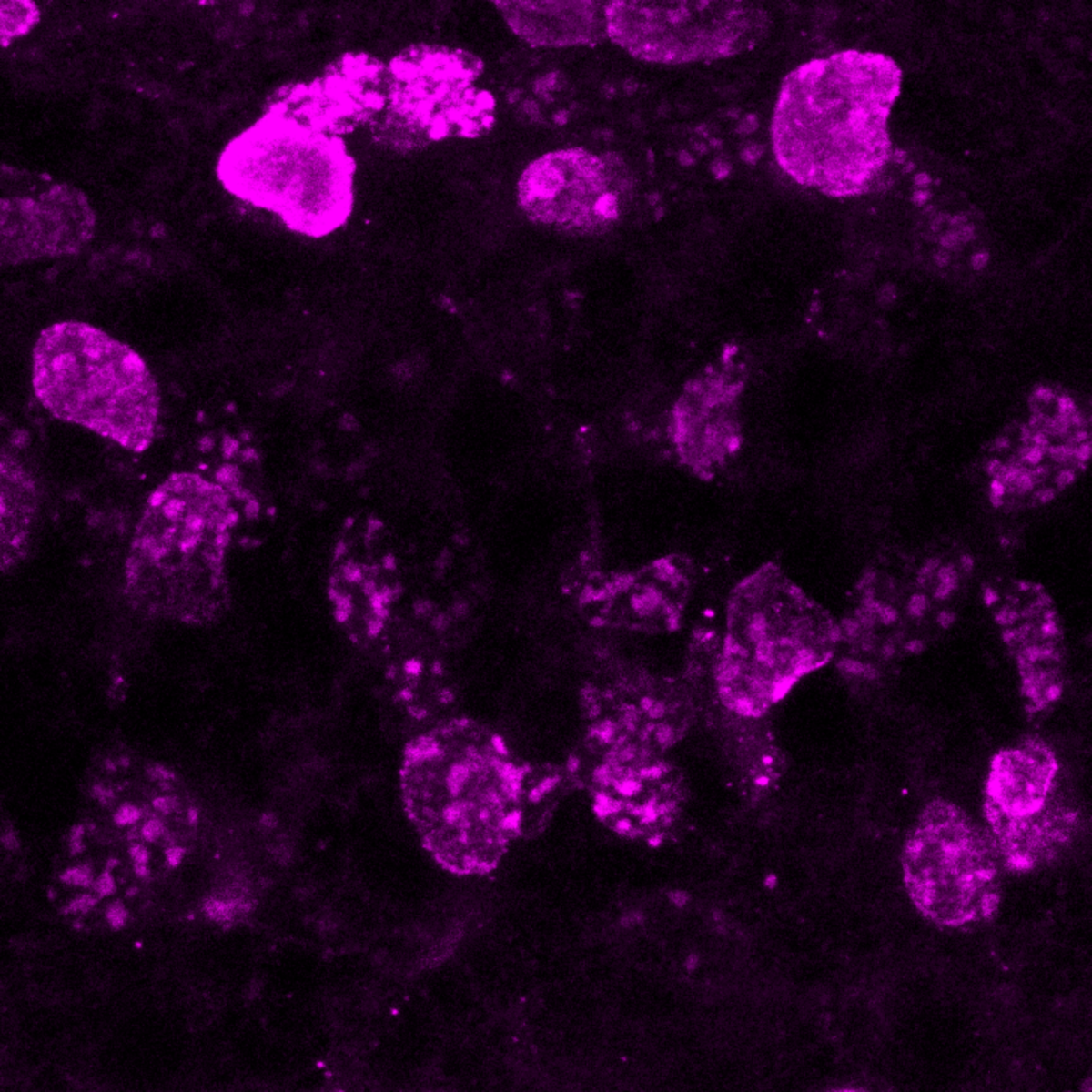

Supplement: Supplementary file 4 — Source Data Fig. 3 [file 44318_2023_11_MOESM4_ESM.zip › EMBOJ-2023-113564_SourceDataForFigure3/3A/P0/CloseUp WT P0 GCL_Merge_Ki67.tiff]

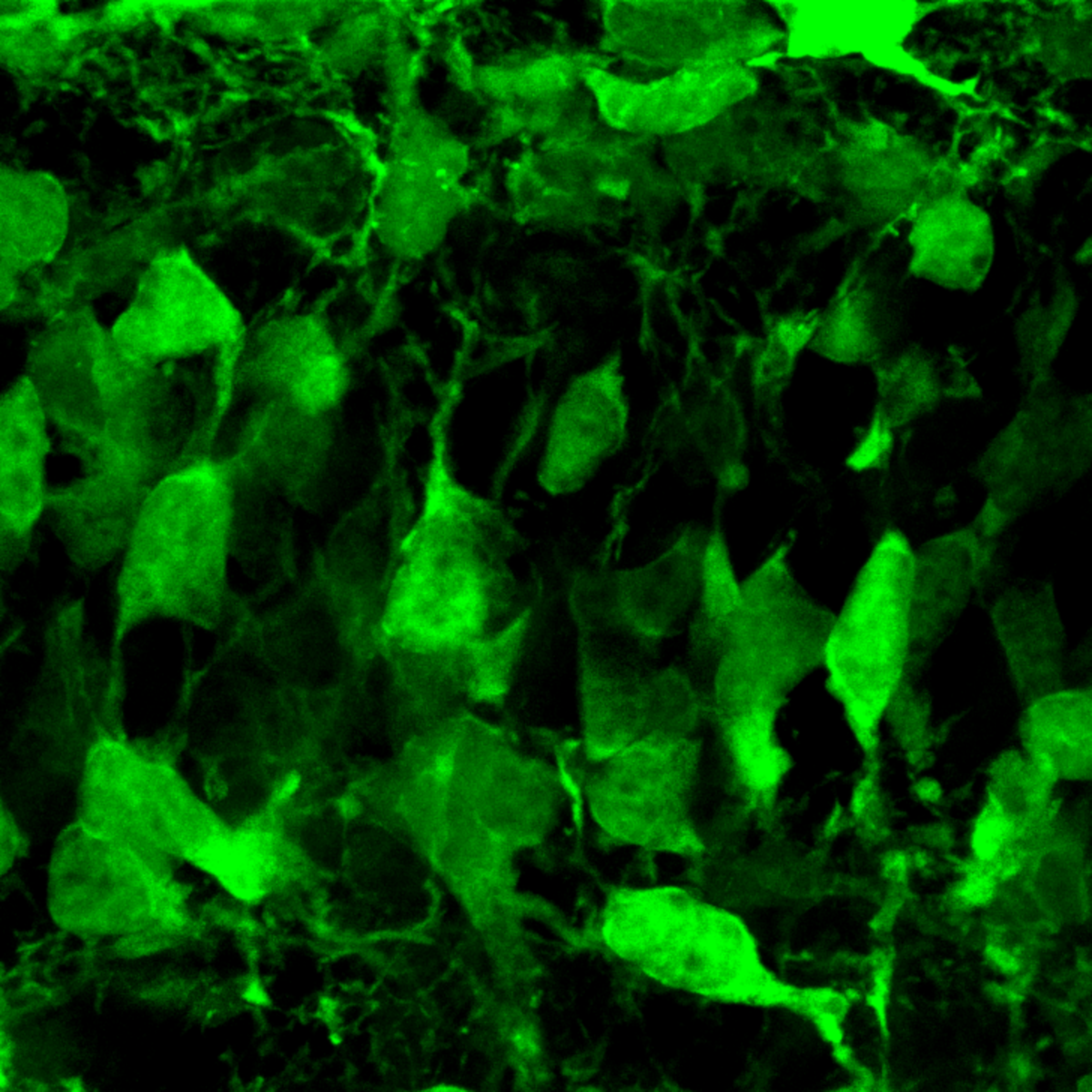

Supplement: Supplementary file 4 — Source Data Fig. 3 [file 44318_2023_11_MOESM4_ESM.zip › EMBOJ-2023-113564_SourceDataForFigure3/3A/P0/CloseUp WT P0 GCL_Merge_Nestin.tiff]

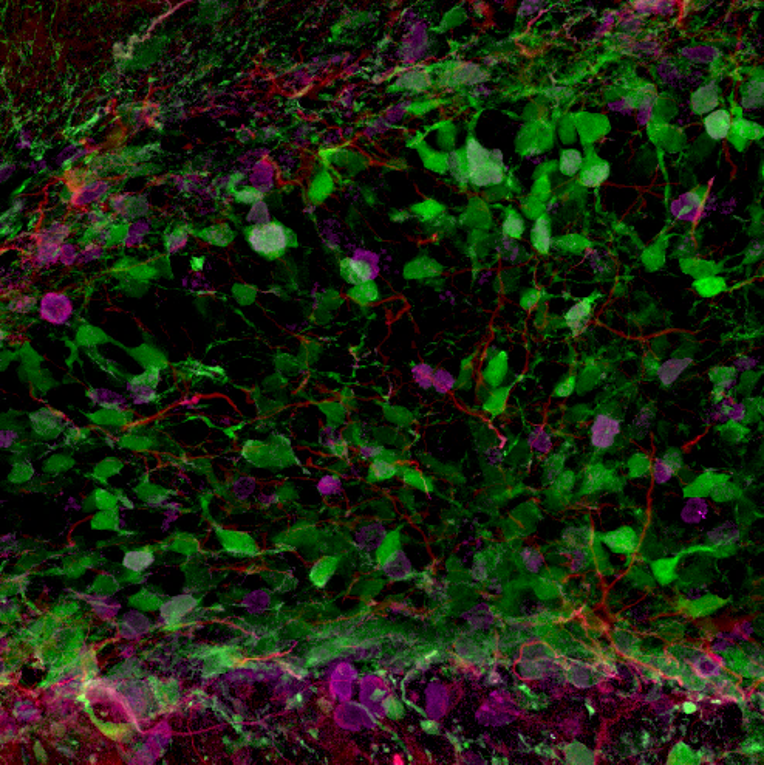

Supplement: Supplementary file 4 — Source Data Fig. 3 [file 44318_2023_11_MOESM4_ESM.zip › EMBOJ-2023-113564_SourceDataForFigure3/3A/P0/KO P0 Dentate gyrus_Merge.tif]

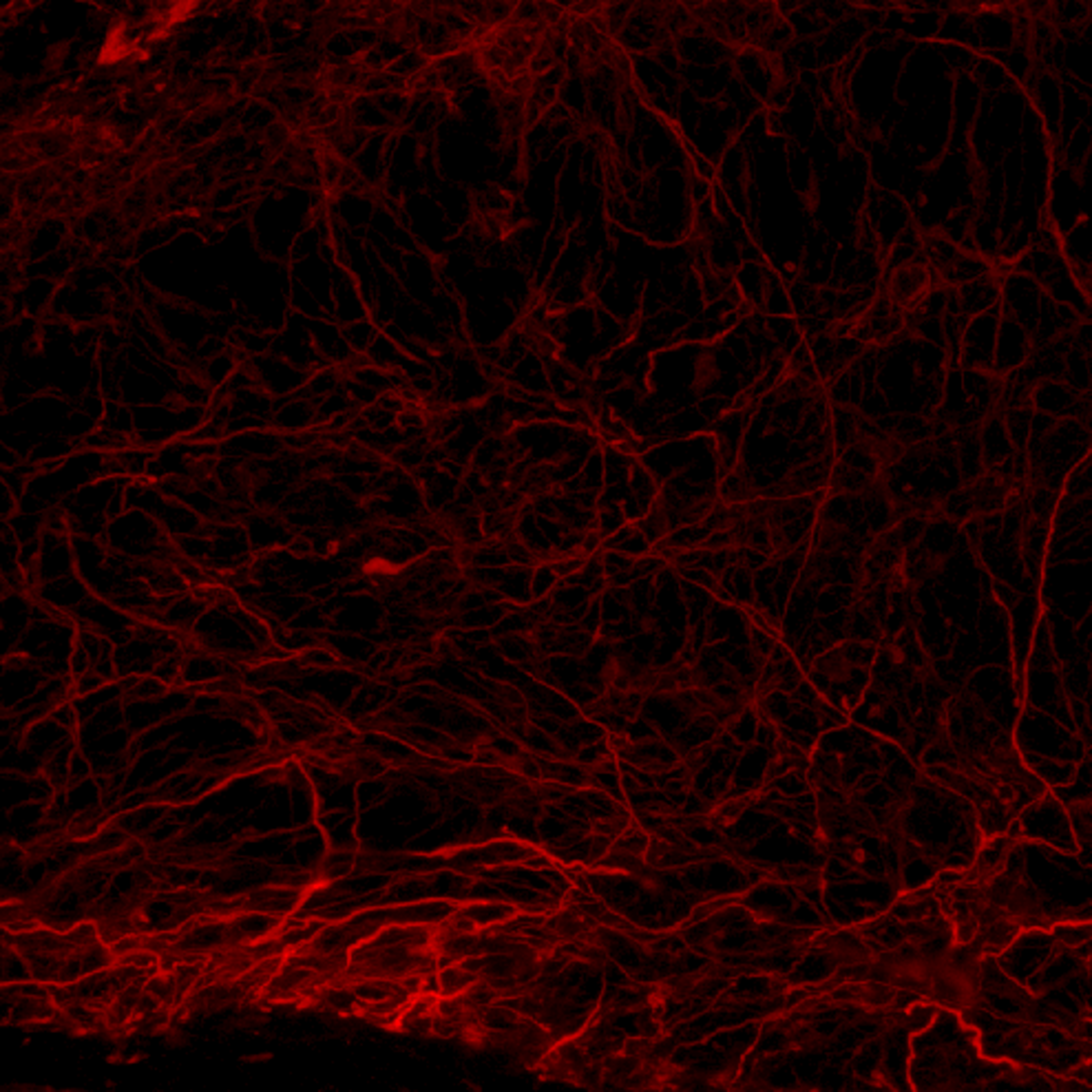

Supplement: Supplementary file 4 — Source Data Fig. 3 [file 44318_2023_11_MOESM4_ESM.zip › EMBOJ-2023-113564_SourceDataForFigure3/3A/P0/WT P0 Dentate gyrus_GFAP.tiff]

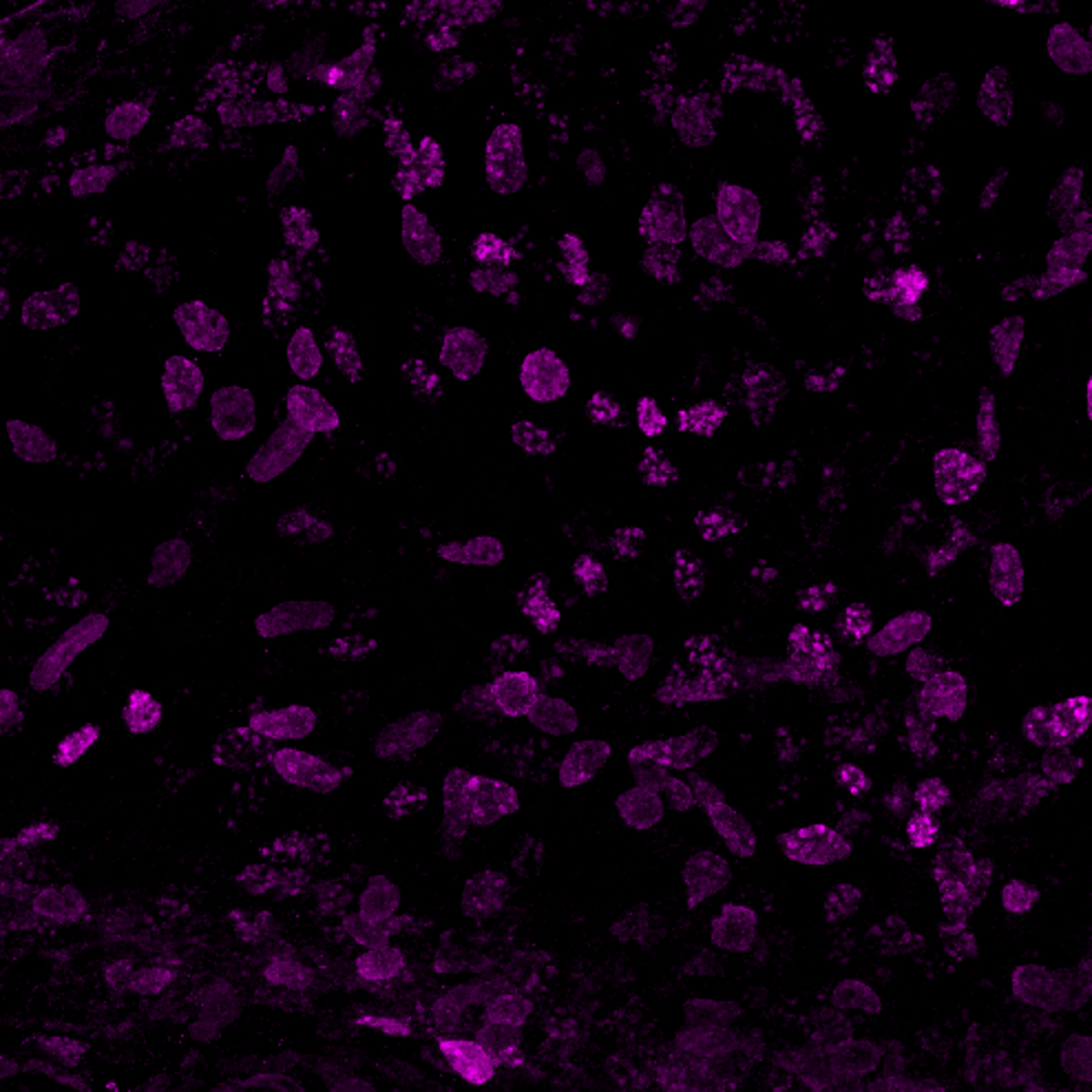

Supplement: Supplementary file 4 — Source Data Fig. 3 [file 44318_2023_11_MOESM4_ESM.zip › EMBOJ-2023-113564_SourceDataForFigure3/3A/P0/WT P0 Dentate gyrus_Ki67.tiff]

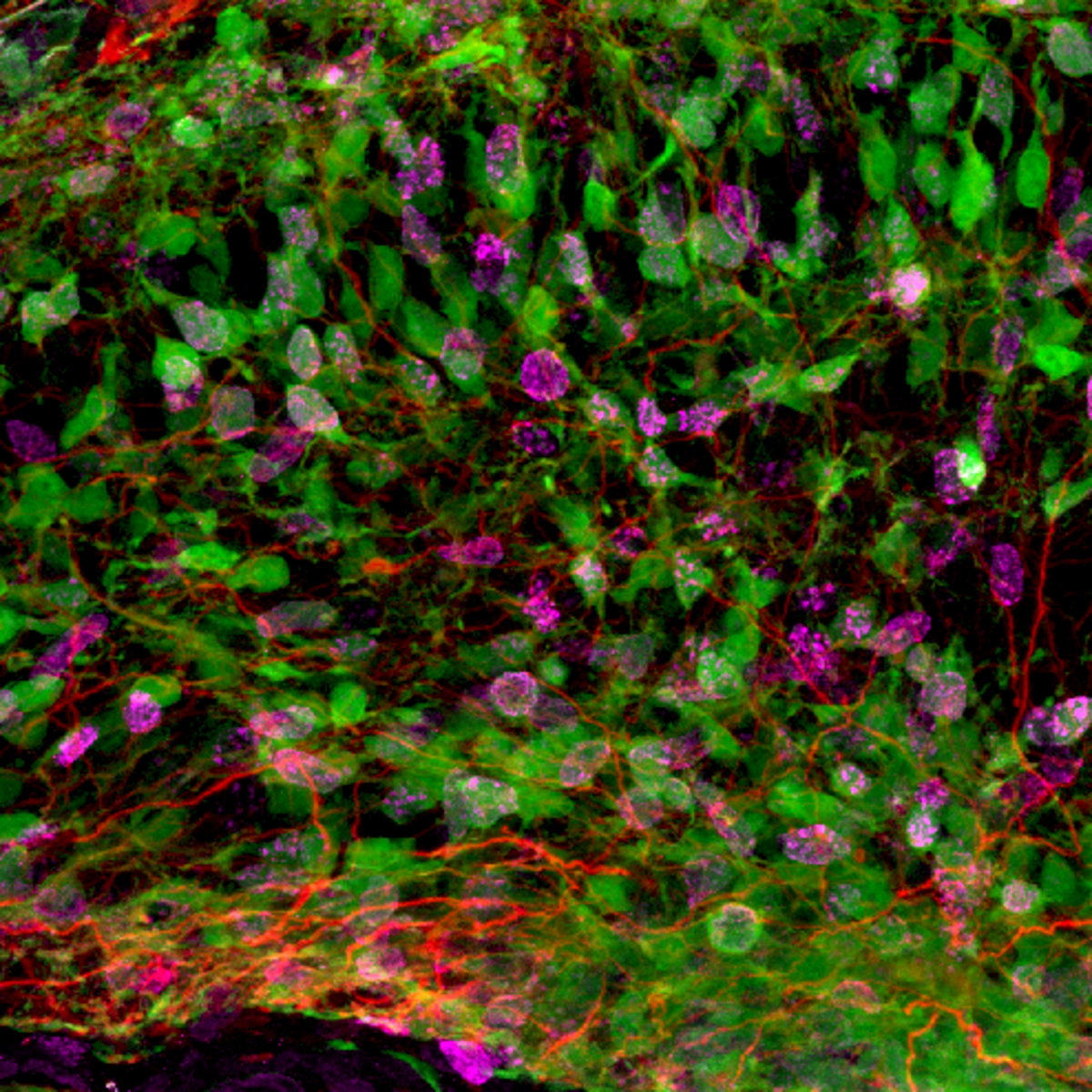

Supplement: Supplementary file 4 — Source Data Fig. 3 [file 44318_2023_11_MOESM4_ESM.zip › EMBOJ-2023-113564_SourceDataForFigure3/3A/P0/WT P0 Dentate gyrus_Merge.tiff]

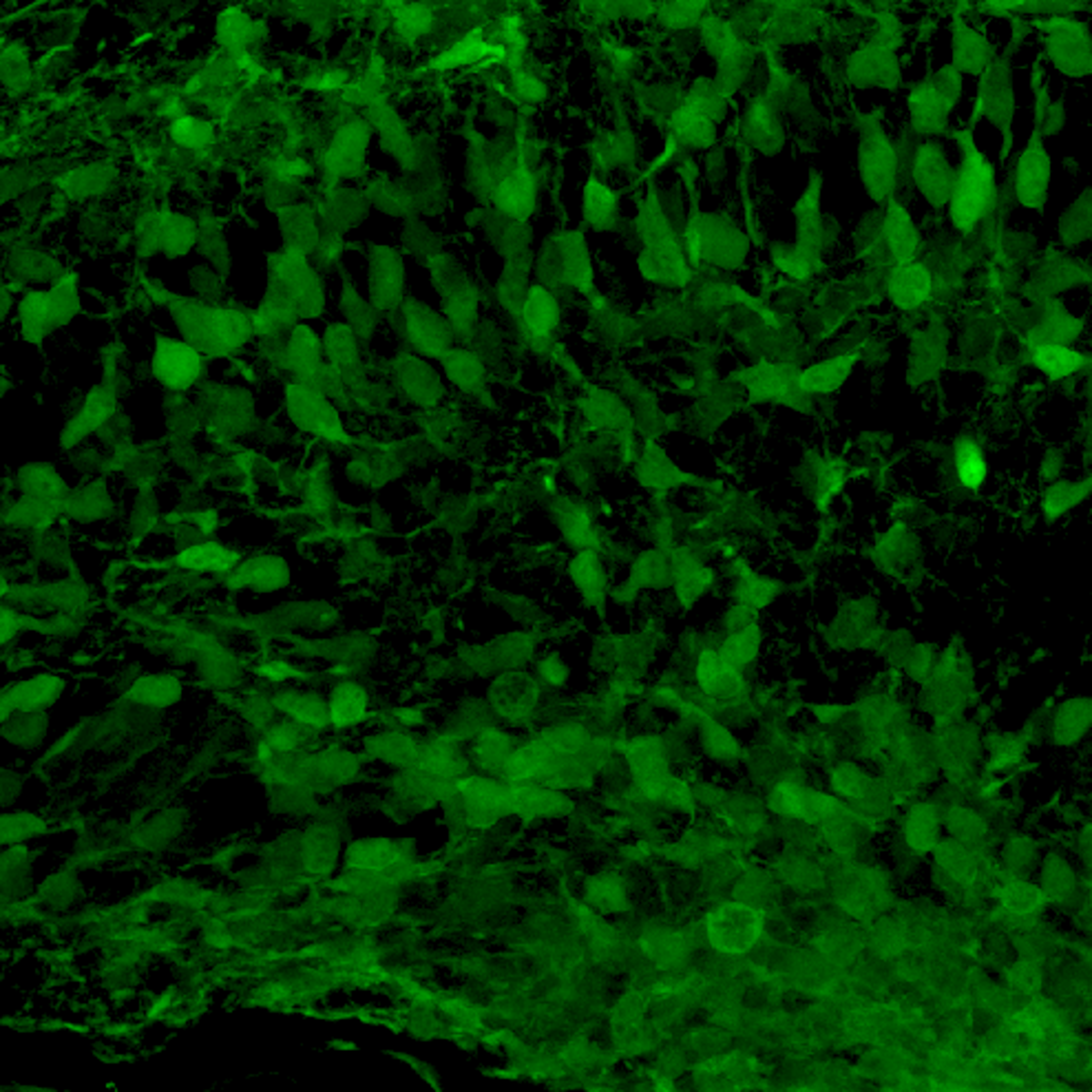

Supplement: Supplementary file 4 — Source Data Fig. 3 [file 44318_2023_11_MOESM4_ESM.zip › EMBOJ-2023-113564_SourceDataForFigure3/3A/P0/WT P0 Dentate gyrus_Nestin.tiff]

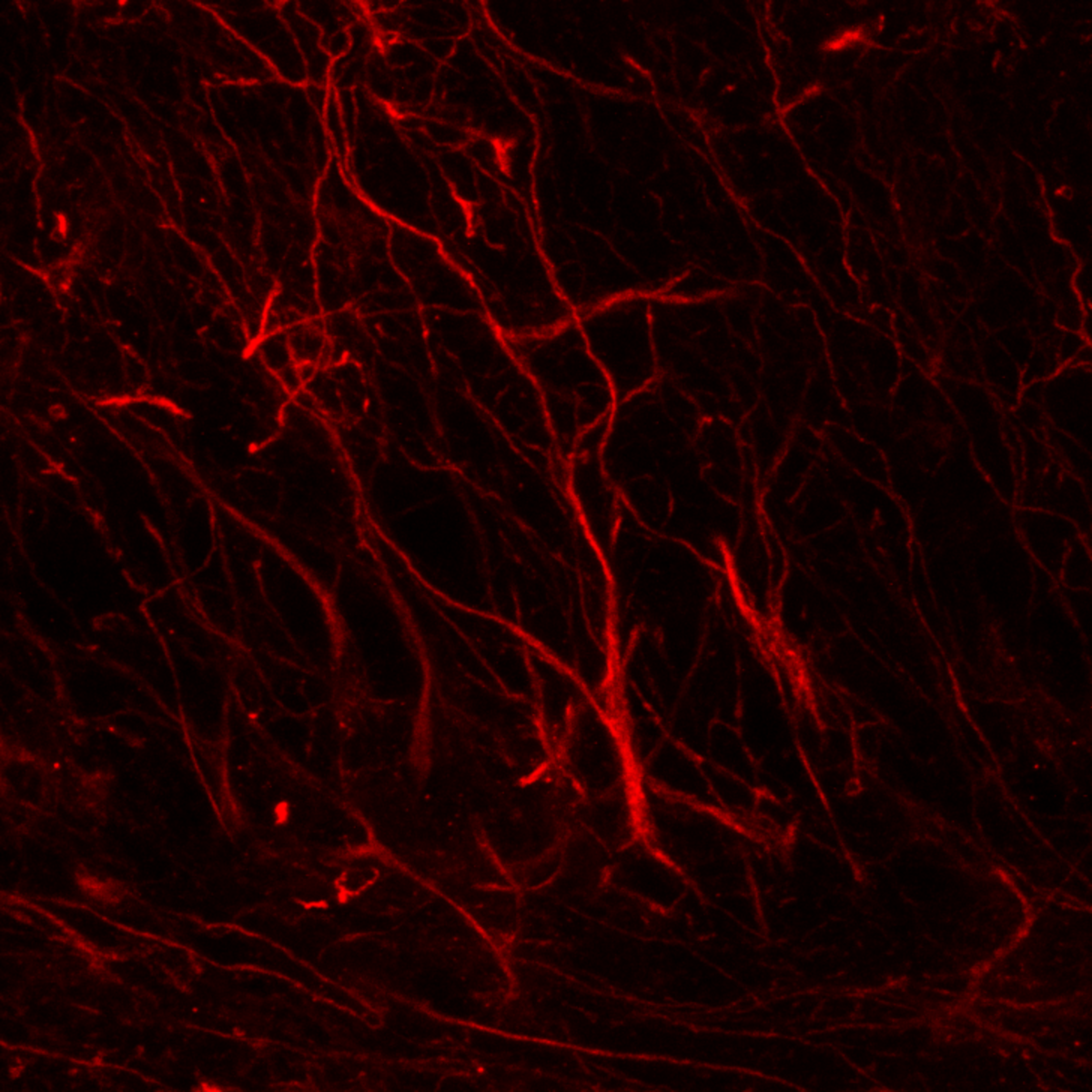

Supplement: Supplementary file 4 — Source Data Fig. 3 [file 44318_2023_11_MOESM4_ESM.zip › EMBOJ-2023-113564_SourceDataForFigure3/3A/P10/CloseUp KO P10 GCL_GFAP.tiff]

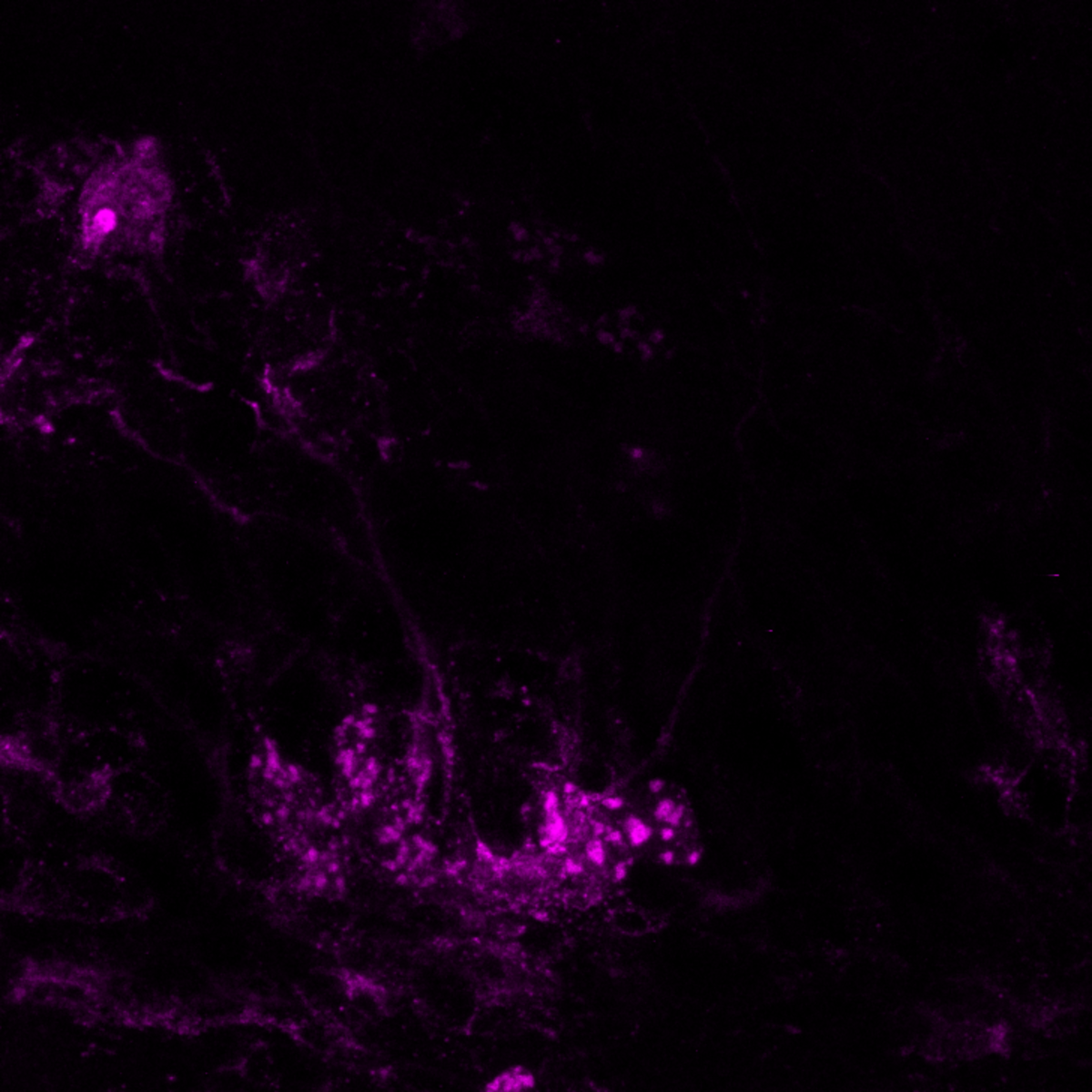

Supplement: Supplementary file 4 — Source Data Fig. 3 [file 44318_2023_11_MOESM4_ESM.zip › EMBOJ-2023-113564_SourceDataForFigure3/3A/P10/CloseUp KO P10 GCL_Ki67.tiff]

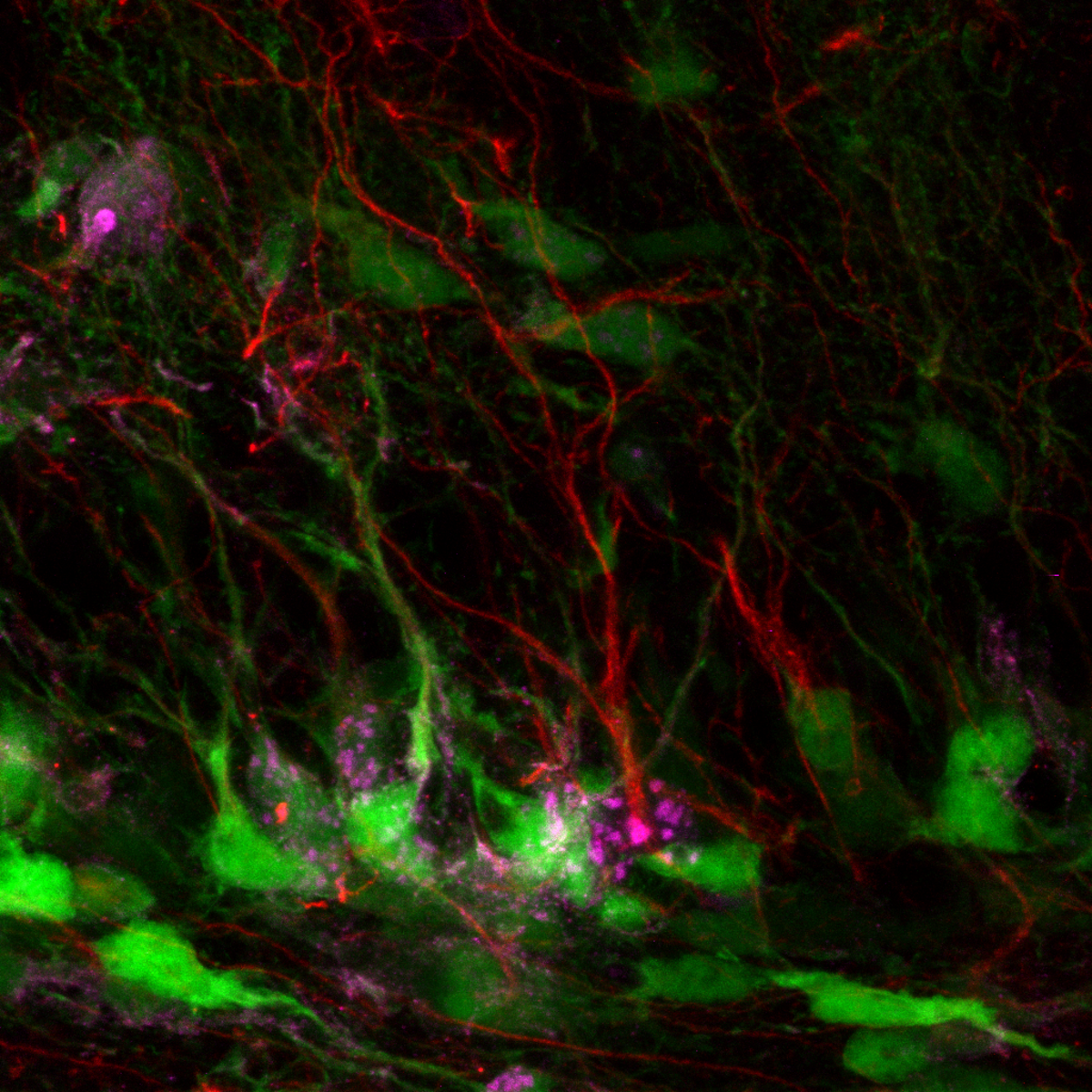

Supplement: Supplementary file 4 — Source Data Fig. 3 [file 44318_2023_11_MOESM4_ESM.zip › EMBOJ-2023-113564_SourceDataForFigure3/3A/P10/CloseUp KO P10 GCL_Merge.tiff]

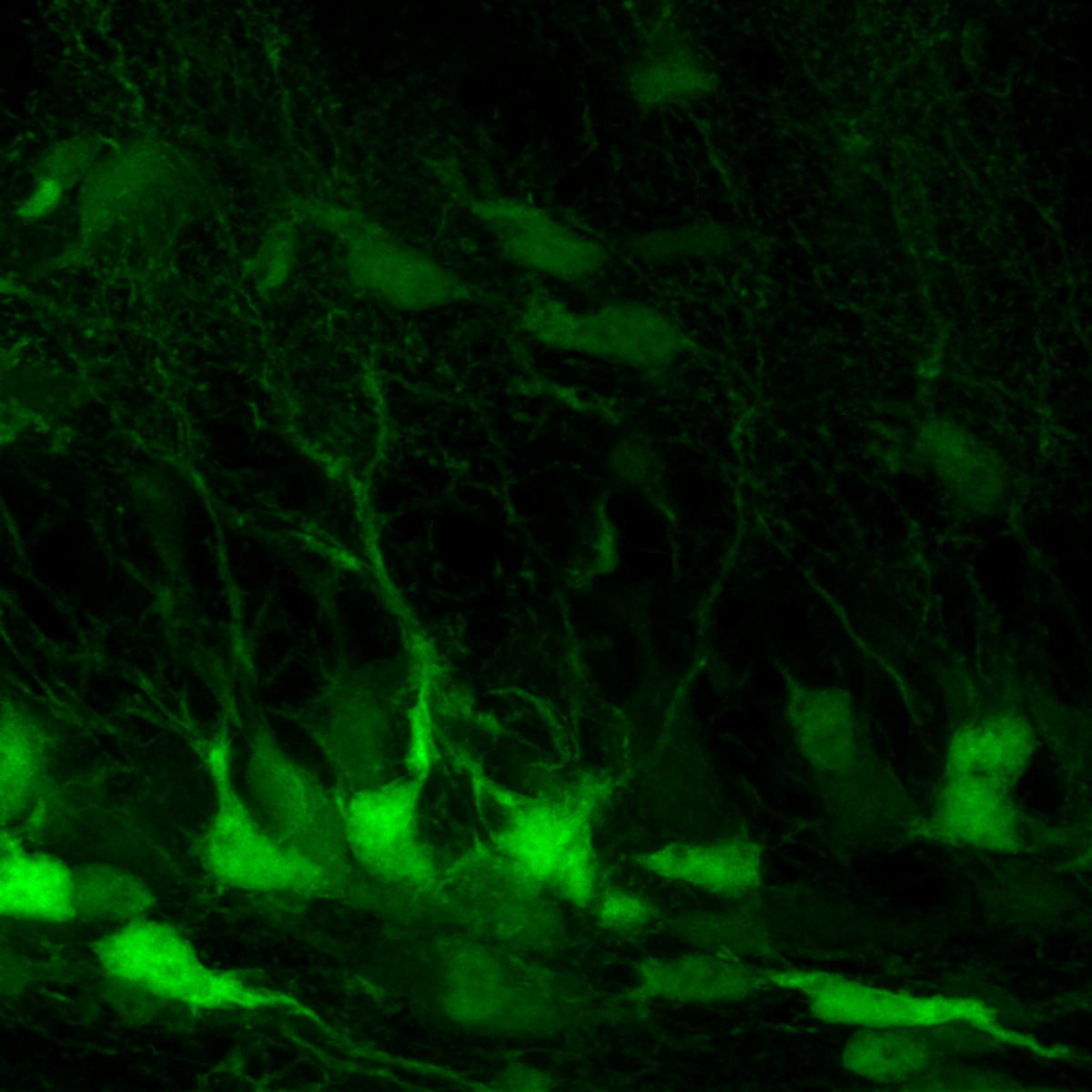

Supplement: Supplementary file 4 — Source Data Fig. 3 [file 44318_2023_11_MOESM4_ESM.zip › EMBOJ-2023-113564_SourceDataForFigure3/3A/P10/CloseUp KO P10 GCL_Nestin.tiff]

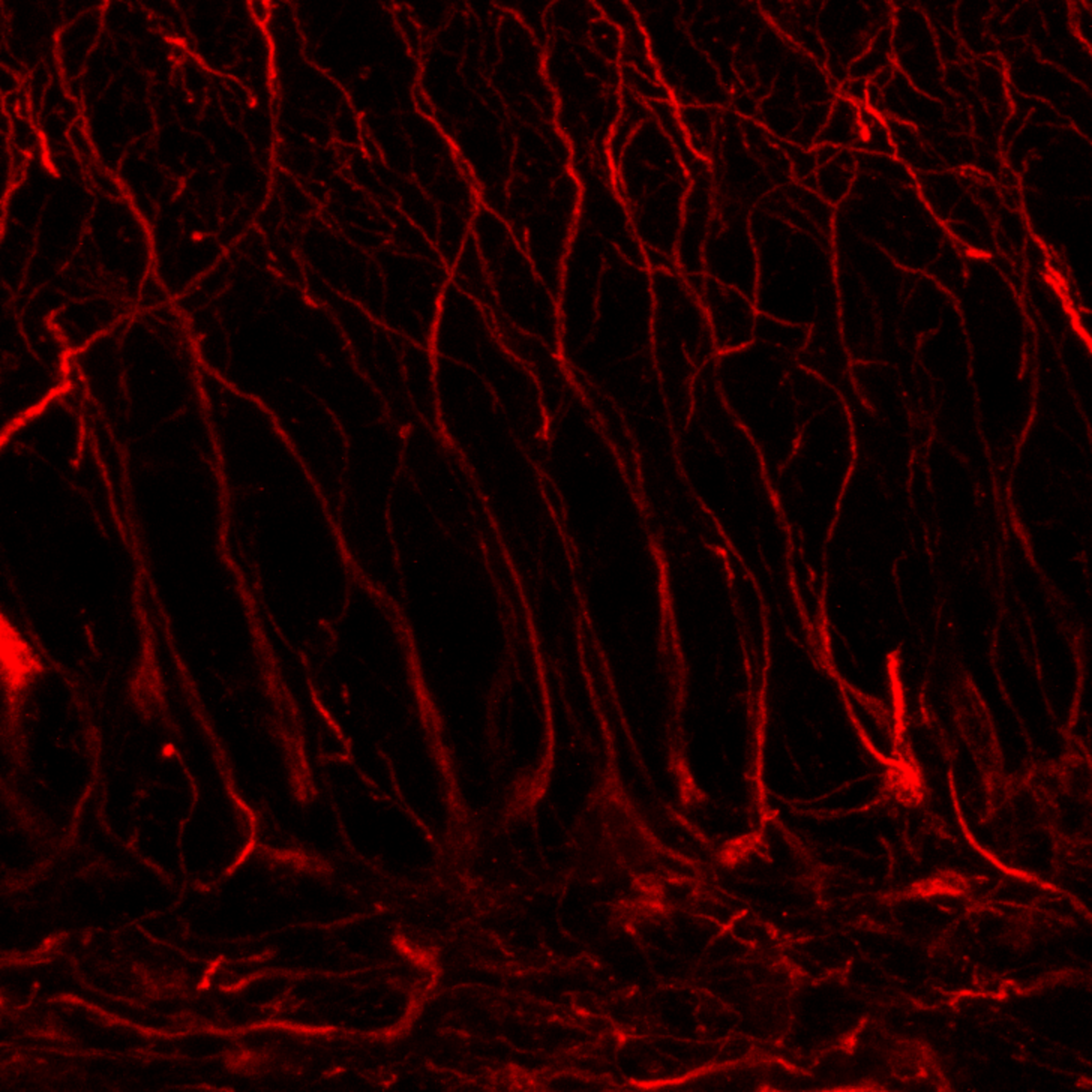

Supplement: Supplementary file 4 — Source Data Fig. 3 [file 44318_2023_11_MOESM4_ESM.zip › EMBOJ-2023-113564_SourceDataForFigure3/3A/P10/CloseUp WT P10 GCL_GFAP.tiff]

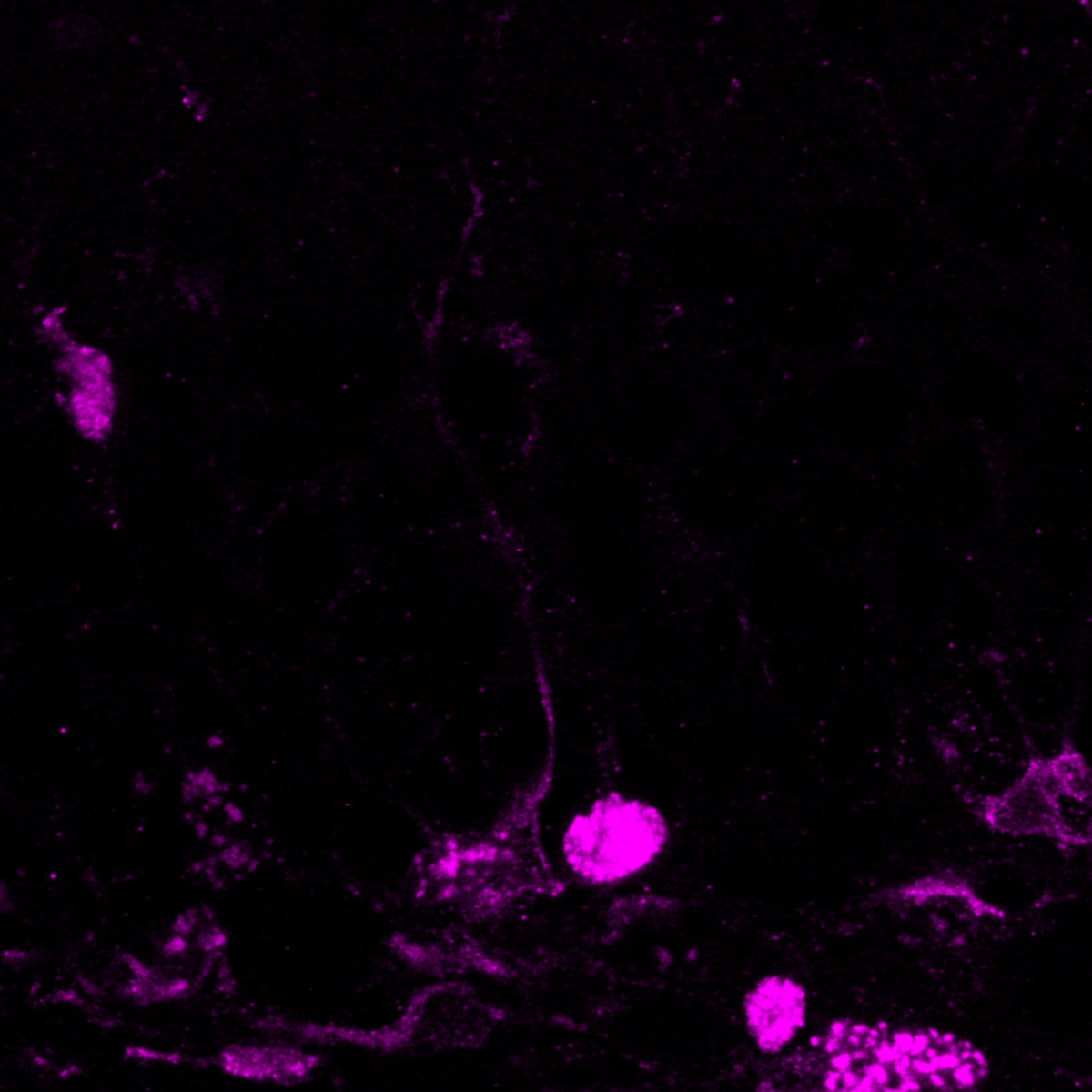

Supplement: Supplementary file 4 — Source Data Fig. 3 [file 44318_2023_11_MOESM4_ESM.zip › EMBOJ-2023-113564_SourceDataForFigure3/3A/P10/CloseUp WT P10 GCL_Ki67.tiff]

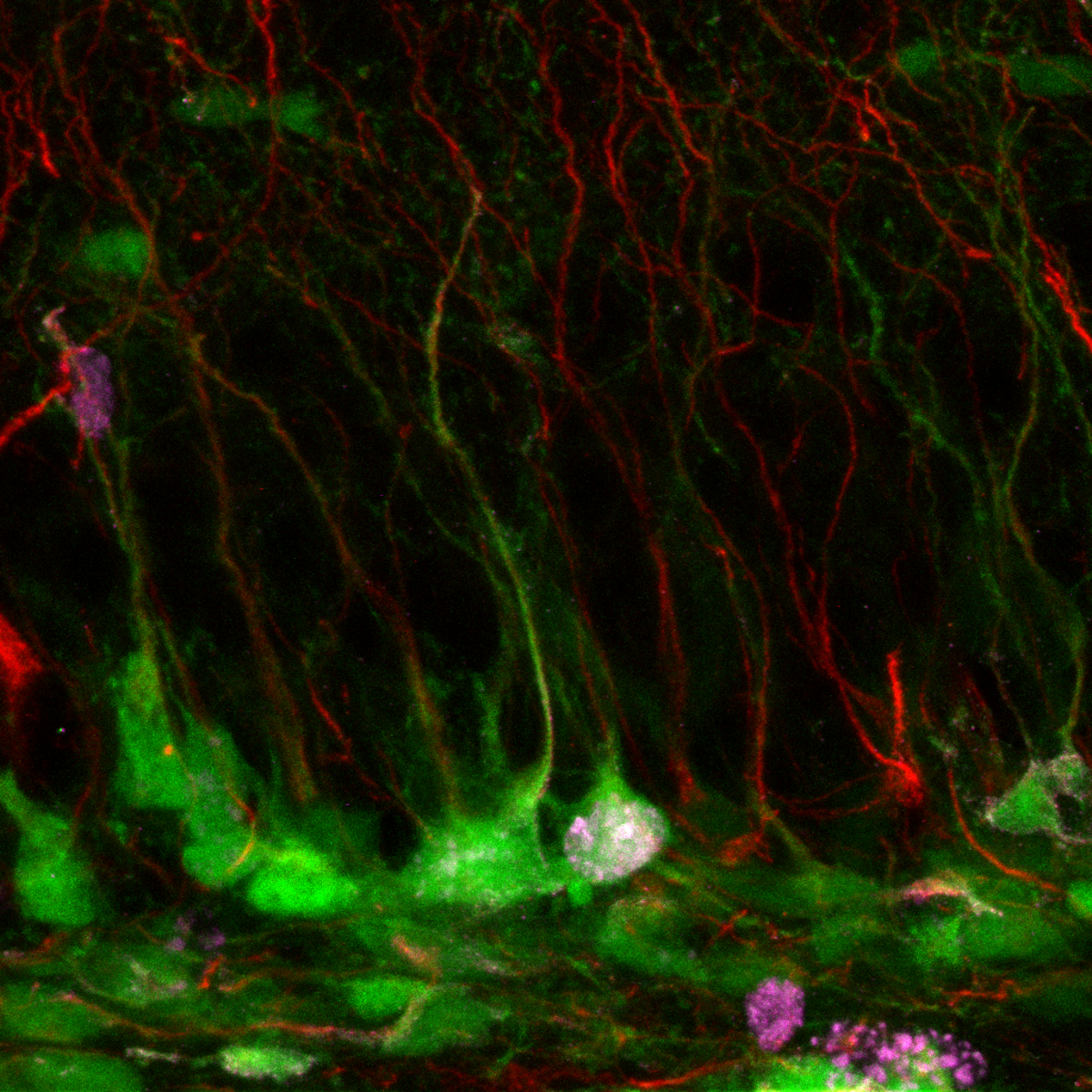

Supplement: Supplementary file 4 — Source Data Fig. 3 [file 44318_2023_11_MOESM4_ESM.zip › EMBOJ-2023-113564_SourceDataForFigure3/3A/P10/CloseUp WT P10 GCL_Merge.tiff]

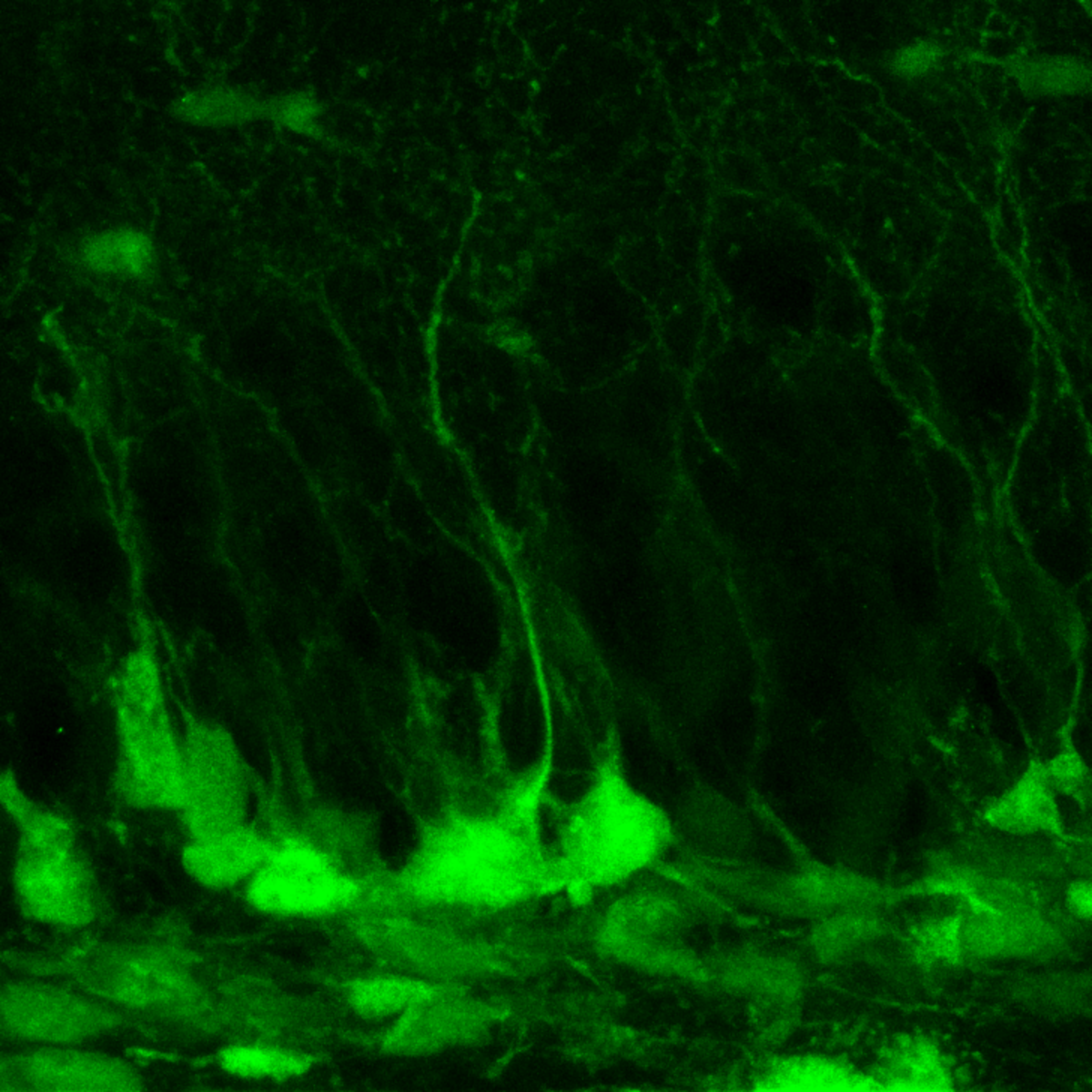

Supplement: Supplementary file 4 — Source Data Fig. 3 [file 44318_2023_11_MOESM4_ESM.zip › EMBOJ-2023-113564_SourceDataForFigure3/3A/P10/CloseUp WT P10 GCL_Nestin.tiff]

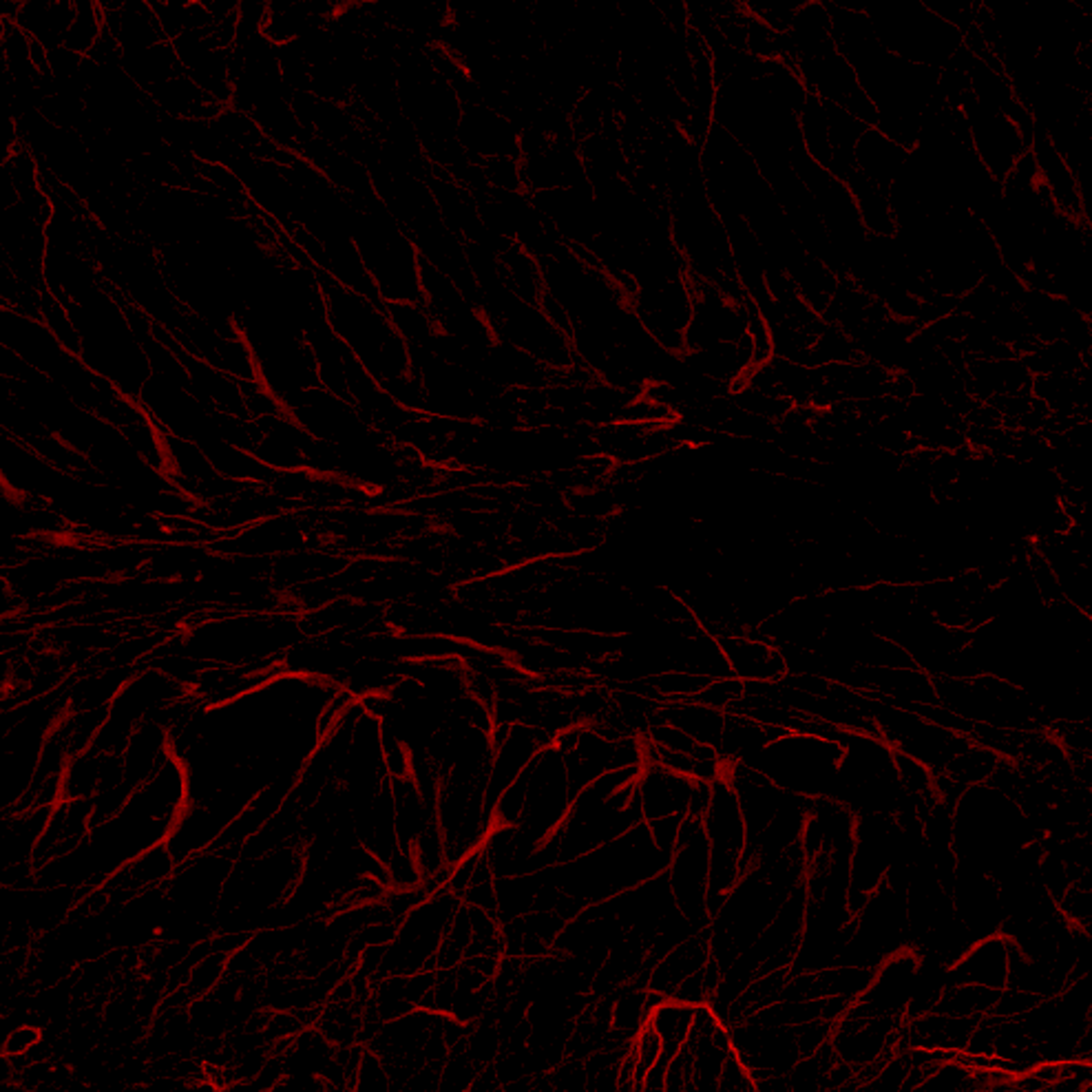

Supplement: Supplementary file 4 — Source Data Fig. 3 [file 44318_2023_11_MOESM4_ESM.zip › EMBOJ-2023-113564_SourceDataForFigure3/3A/P10/KO P10 Dentate Gyrus_GFAP.tiff]

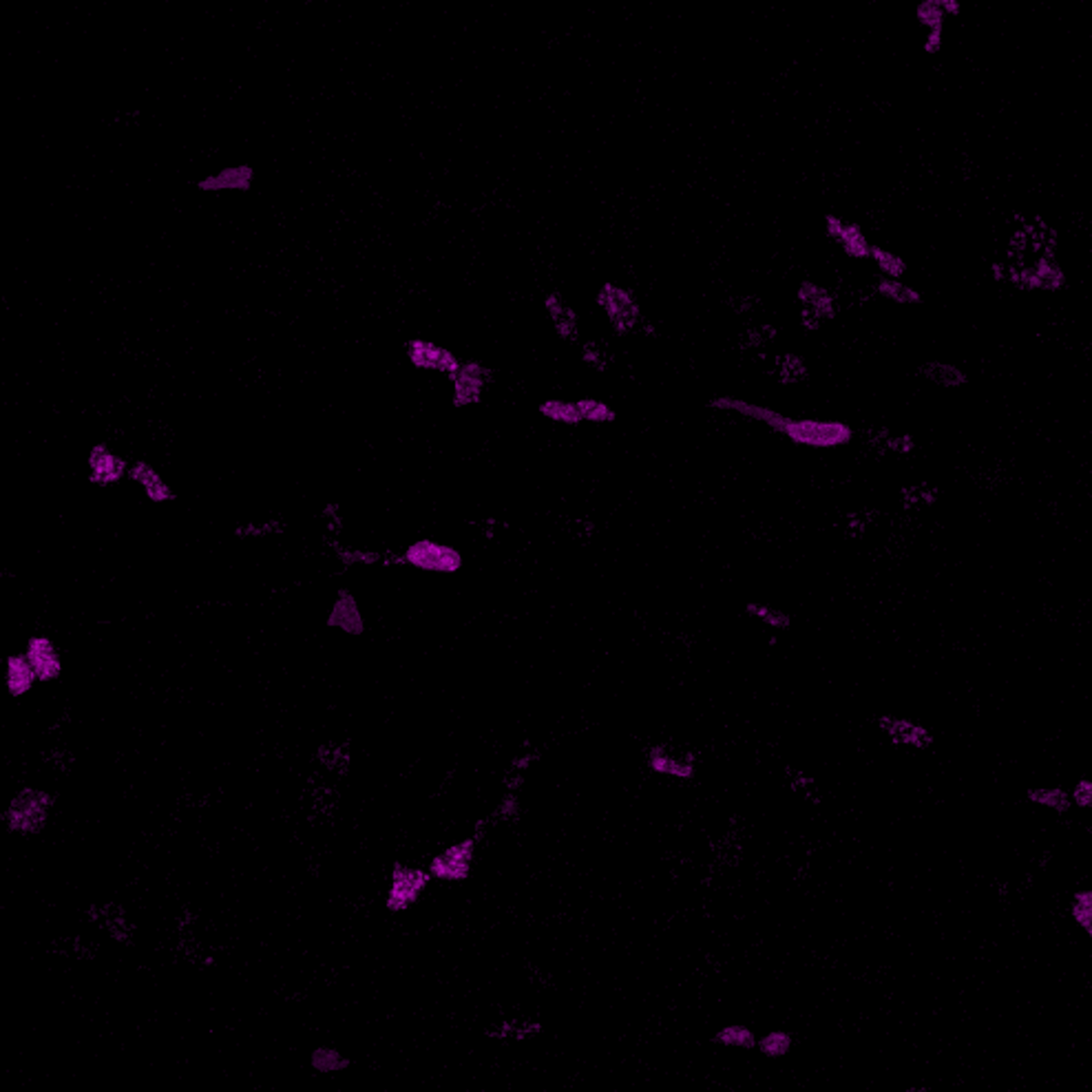

Supplement: Supplementary file 4 — Source Data Fig. 3 [file 44318_2023_11_MOESM4_ESM.zip › EMBOJ-2023-113564_SourceDataForFigure3/3A/P10/KO P10 Dentate Gyrus_Ki67.tiff]

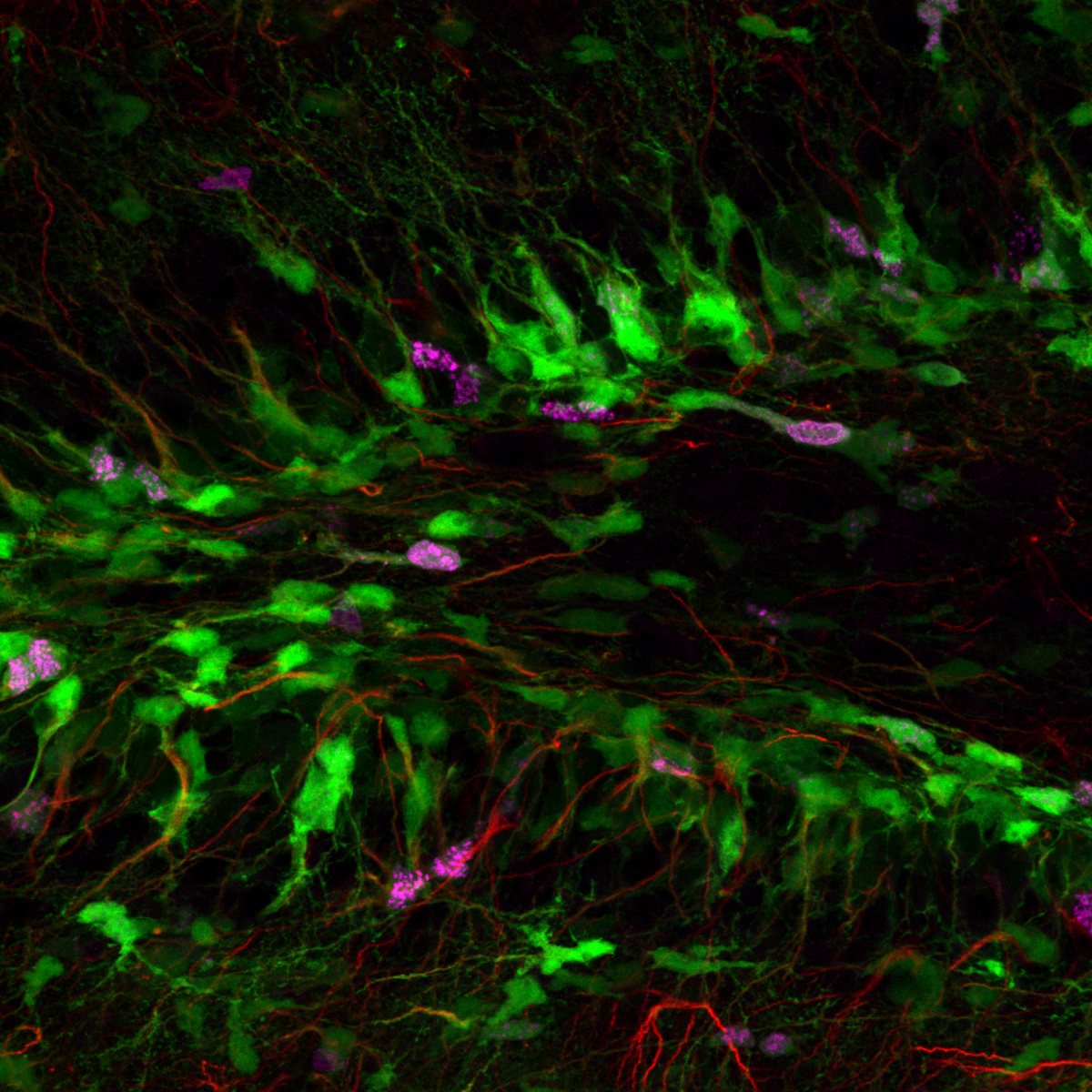

Supplement: Supplementary file 4 — Source Data Fig. 3 [file 44318_2023_11_MOESM4_ESM.zip › EMBOJ-2023-113564_SourceDataForFigure3/3A/P10/KO P10 Dentate Gyrus_Merge.tiff]

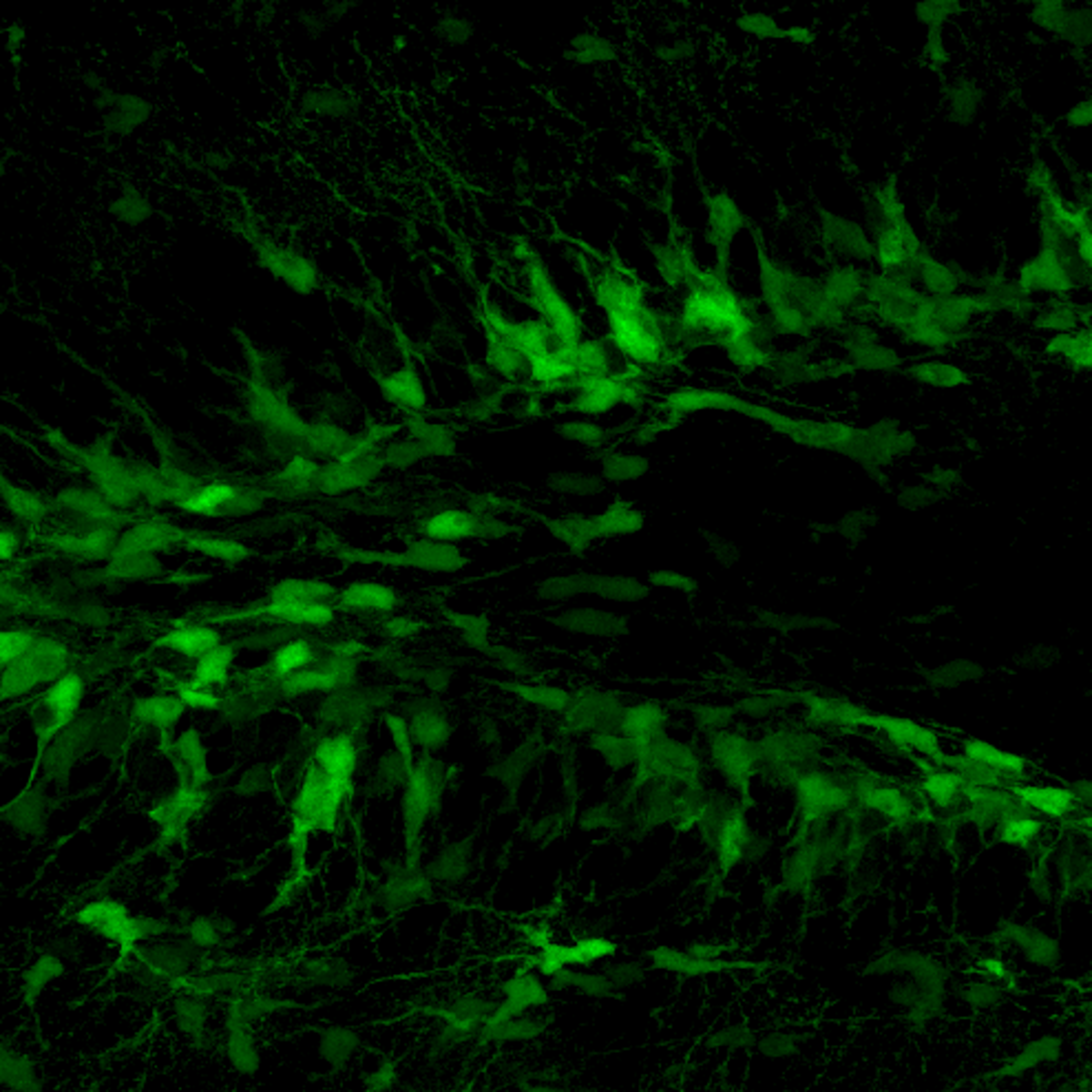

Supplement: Supplementary file 4 — Source Data Fig. 3 [file 44318_2023_11_MOESM4_ESM.zip › EMBOJ-2023-113564_SourceDataForFigure3/3A/P10/KO P10 Dentate Gyrus_Nestin.tiff]

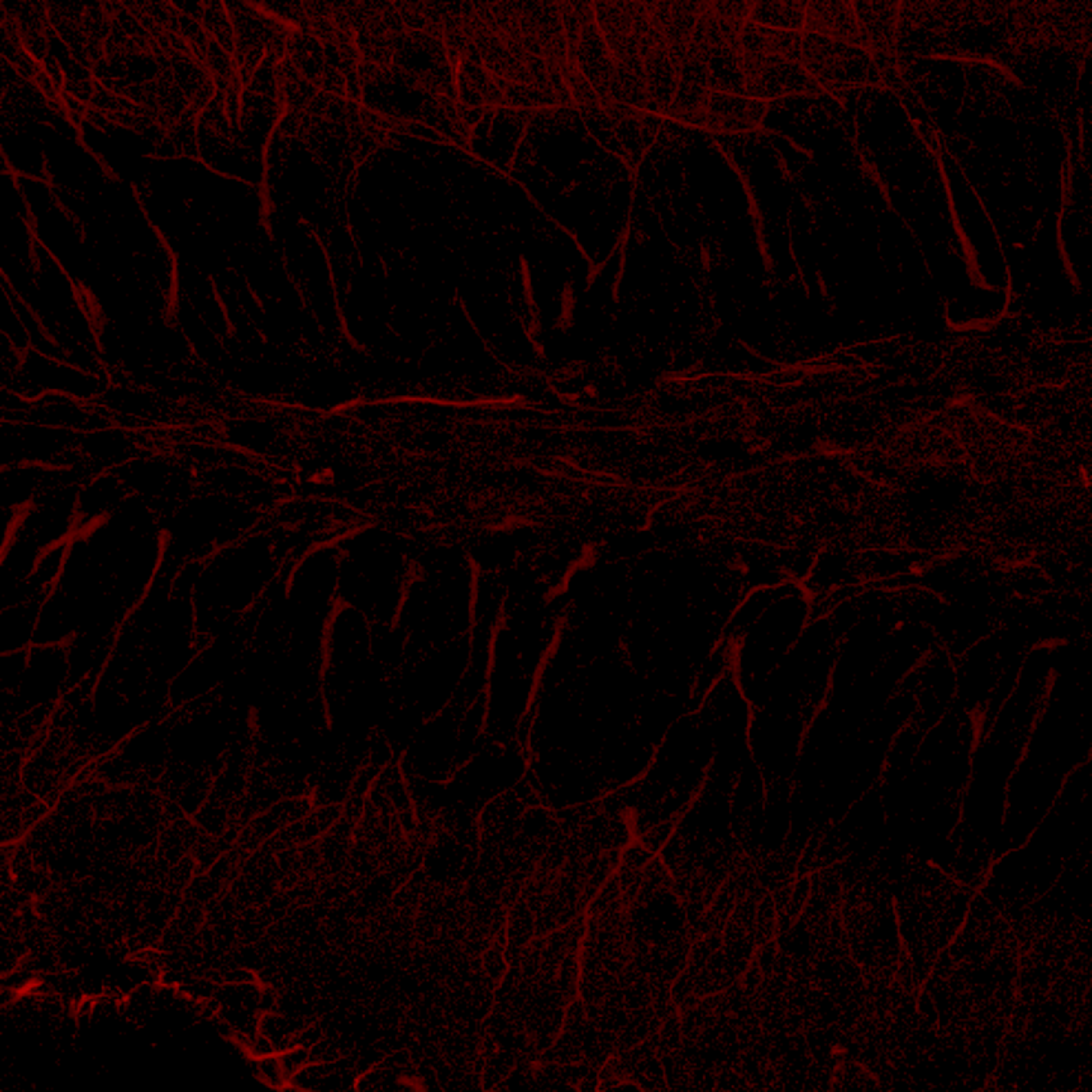

Supplement: Supplementary file 4 — Source Data Fig. 3 [file 44318_2023_11_MOESM4_ESM.zip › EMBOJ-2023-113564_SourceDataForFigure3/3A/P10/WT P10 Dentate Gyrus_GFAP_.tif]

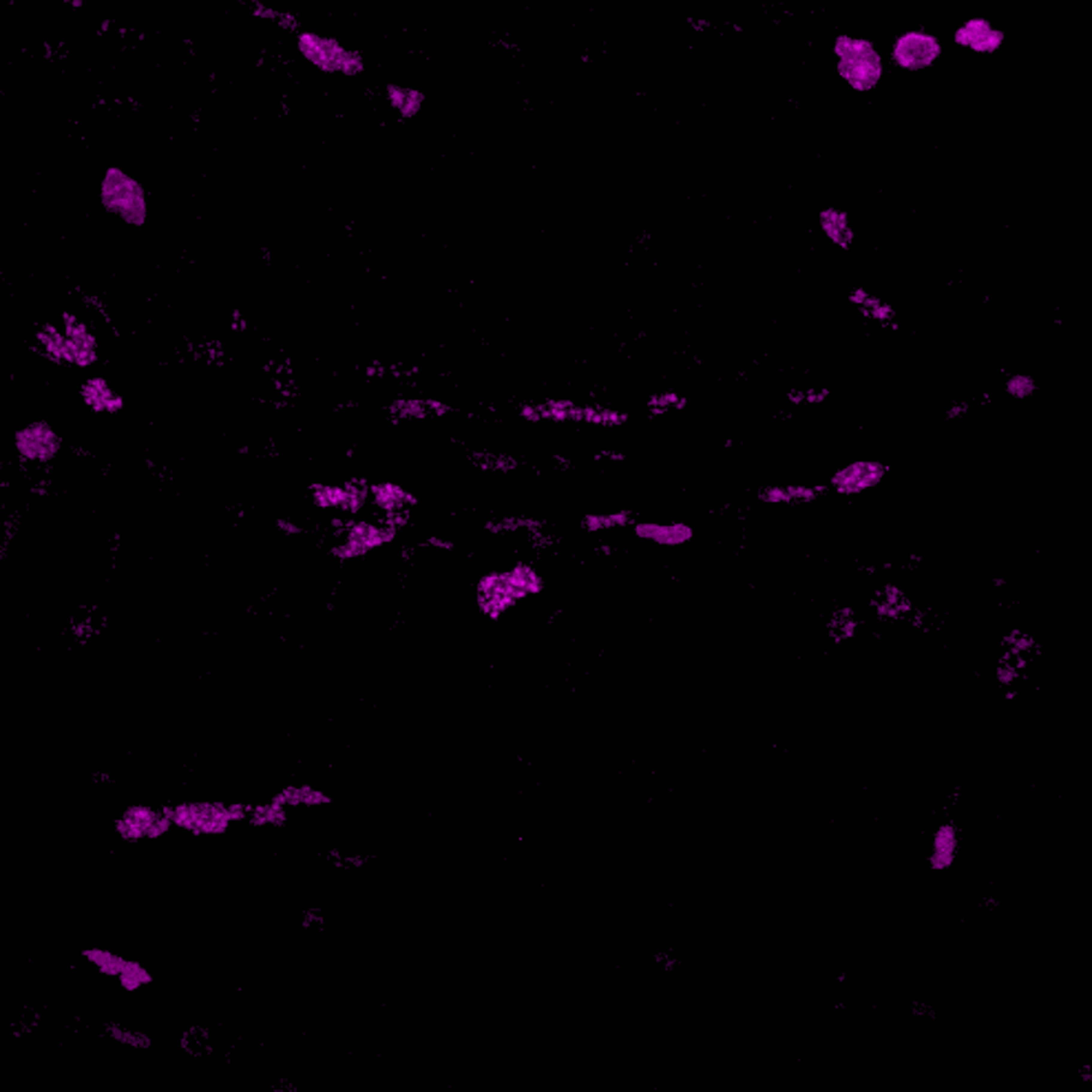

Supplement: Supplementary file 4 — Source Data Fig. 3 [file 44318_2023_11_MOESM4_ESM.zip › EMBOJ-2023-113564_SourceDataForFigure3/3A/P10/WT P10 Dentate Gyrus_Ki67_.tif]

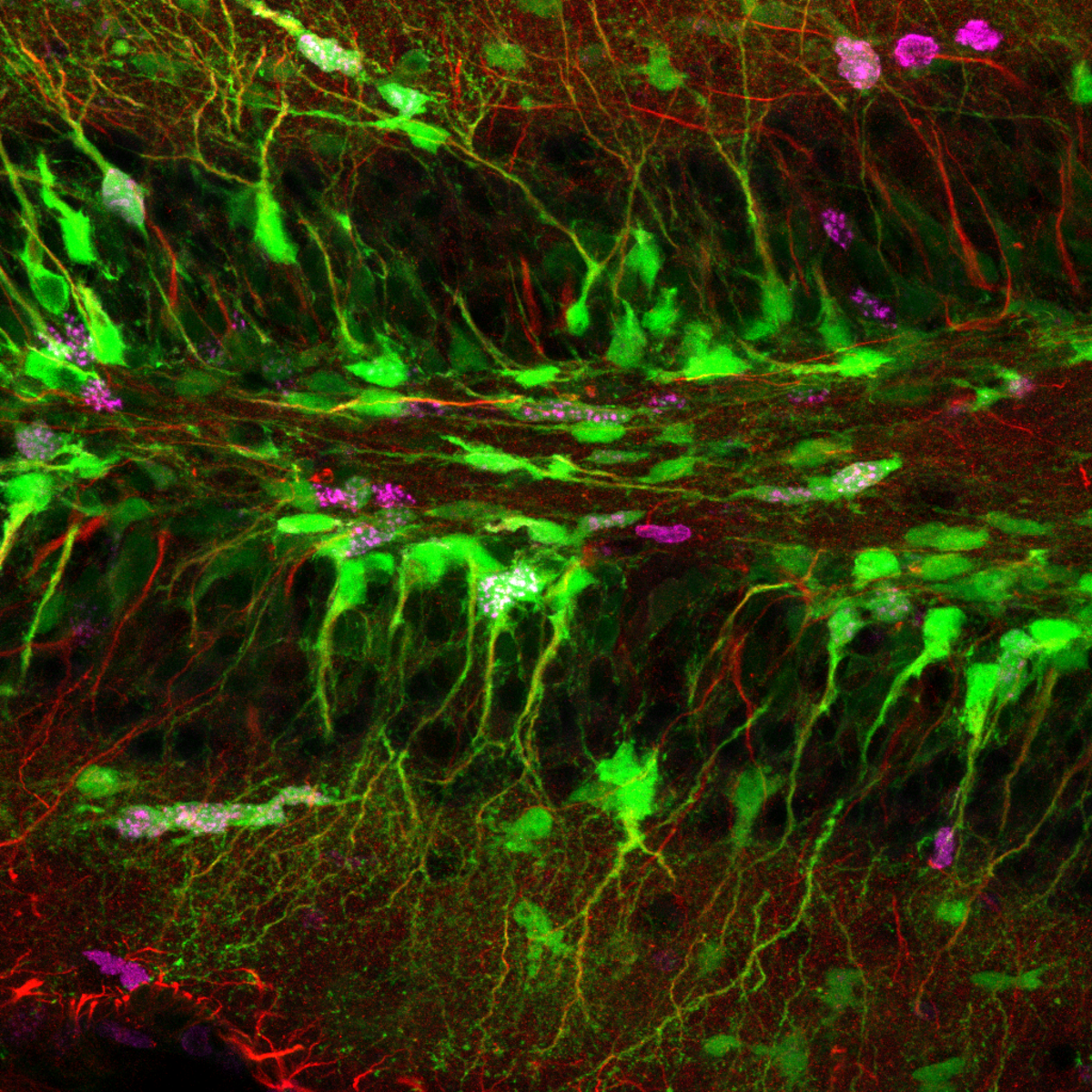

Supplement: Supplementary file 4 — Source Data Fig. 3 [file 44318_2023_11_MOESM4_ESM.zip › EMBOJ-2023-113564_SourceDataForFigure3/3A/P10/WT P10 Dentate Gyrus_Merge_.tif]

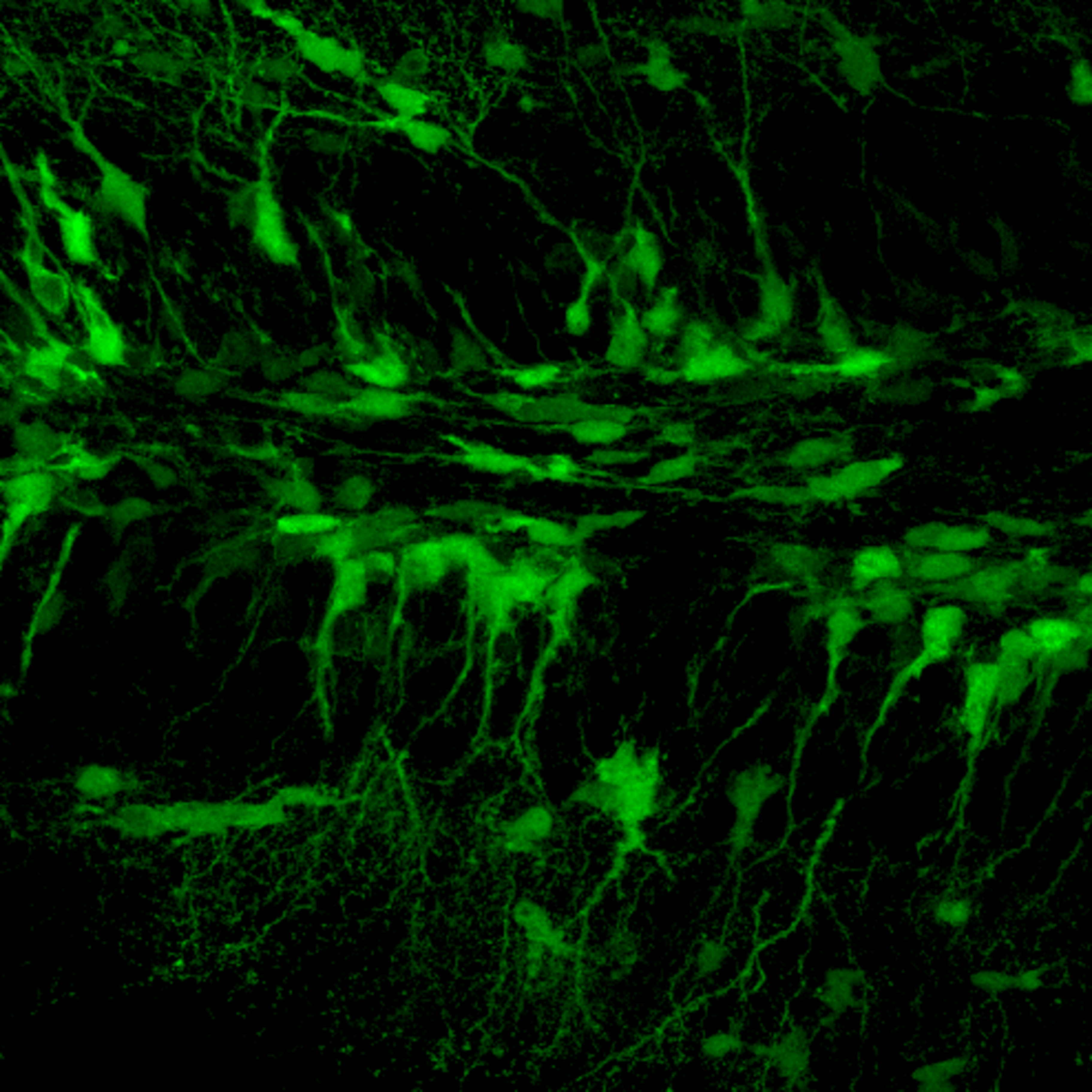

Supplement: Supplementary file 4 — Source Data Fig. 3 [file 44318_2023_11_MOESM4_ESM.zip › EMBOJ-2023-113564_SourceDataForFigure3/3A/P10/WT P10 Dentate Gyrus_Nestin_.tif]

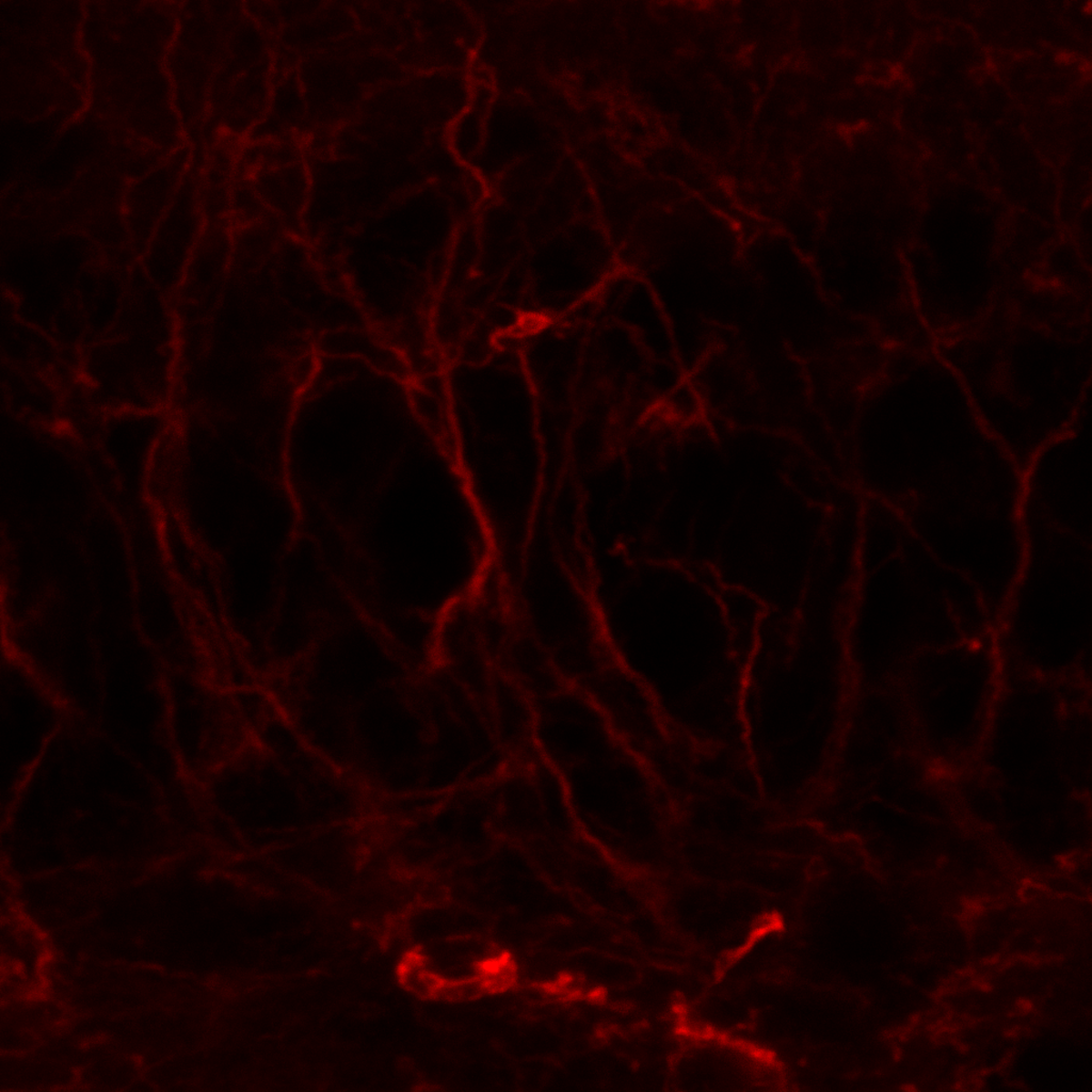

Supplement: Supplementary file 4 — Source Data Fig. 3 [file 44318_2023_11_MOESM4_ESM.zip › EMBOJ-2023-113564_SourceDataForFigure3/3A/P14/CloseUp KO P14 GCL_GFAP.tiff]

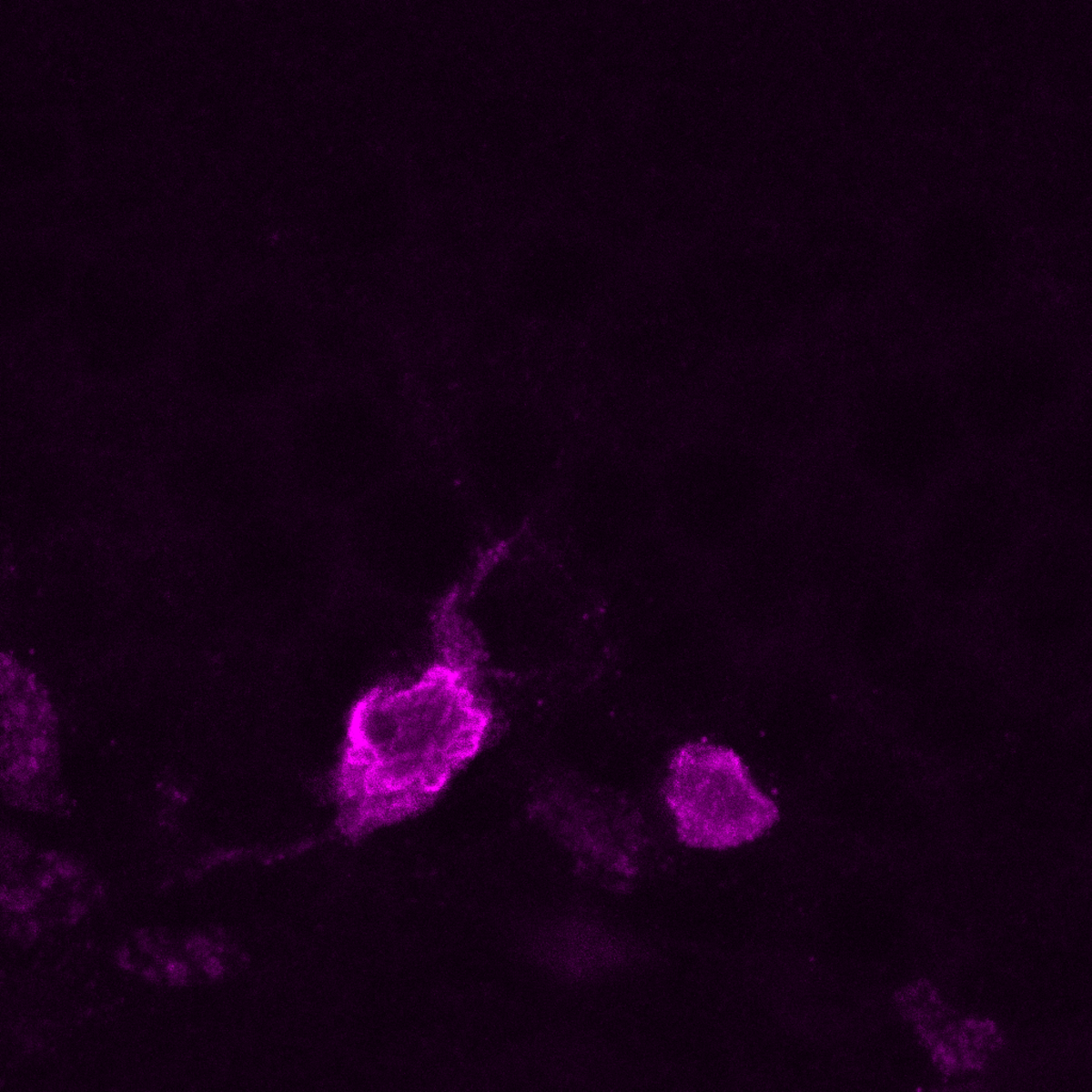

Supplement: Supplementary file 4 — Source Data Fig. 3 [file 44318_2023_11_MOESM4_ESM.zip › EMBOJ-2023-113564_SourceDataForFigure3/3A/P14/CloseUp KO P14 GCL_Ki67.tiff]

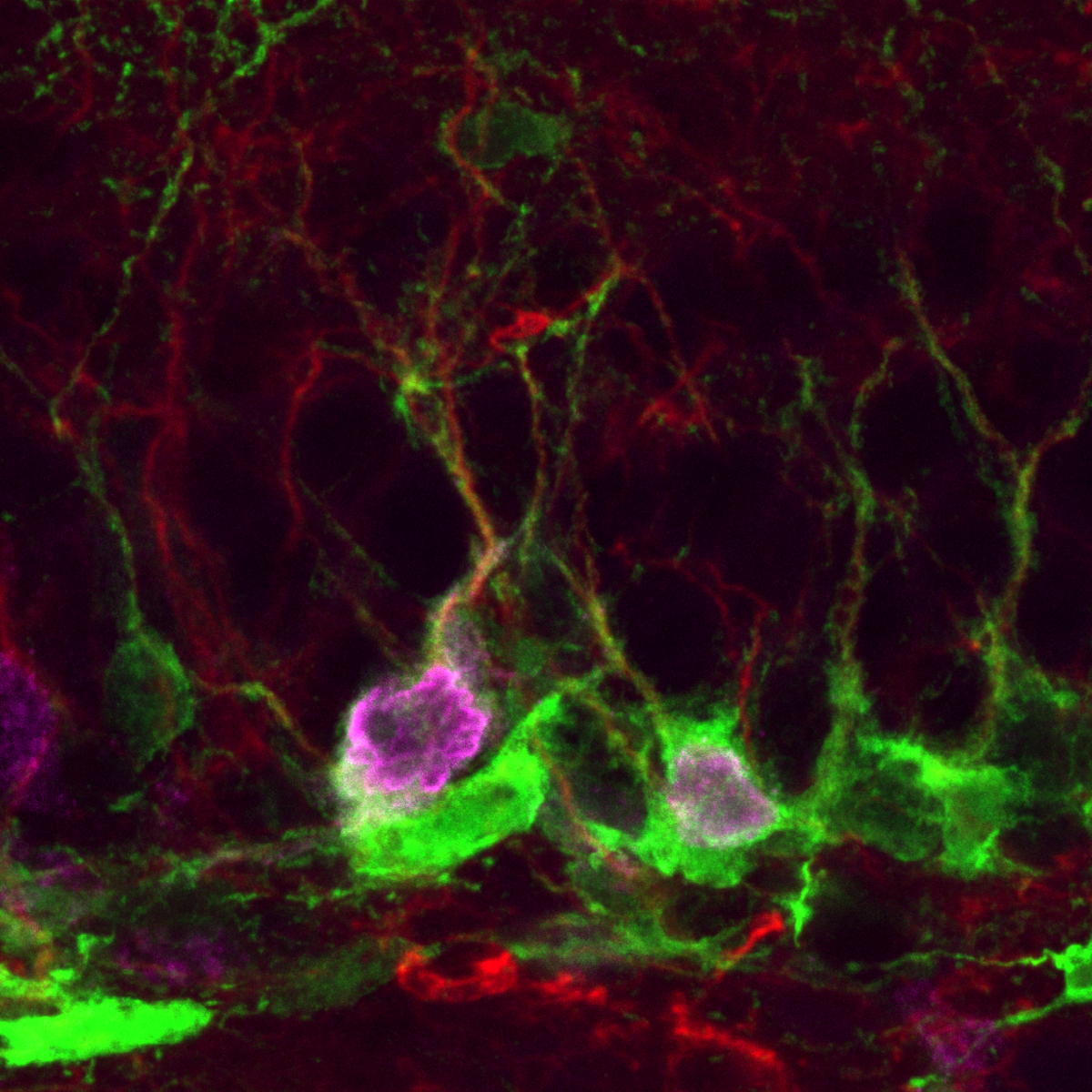

Supplement: Supplementary file 4 — Source Data Fig. 3 [file 44318_2023_11_MOESM4_ESM.zip › EMBOJ-2023-113564_SourceDataForFigure3/3A/P14/CloseUp KO P14 GCL_Merge.tiff]

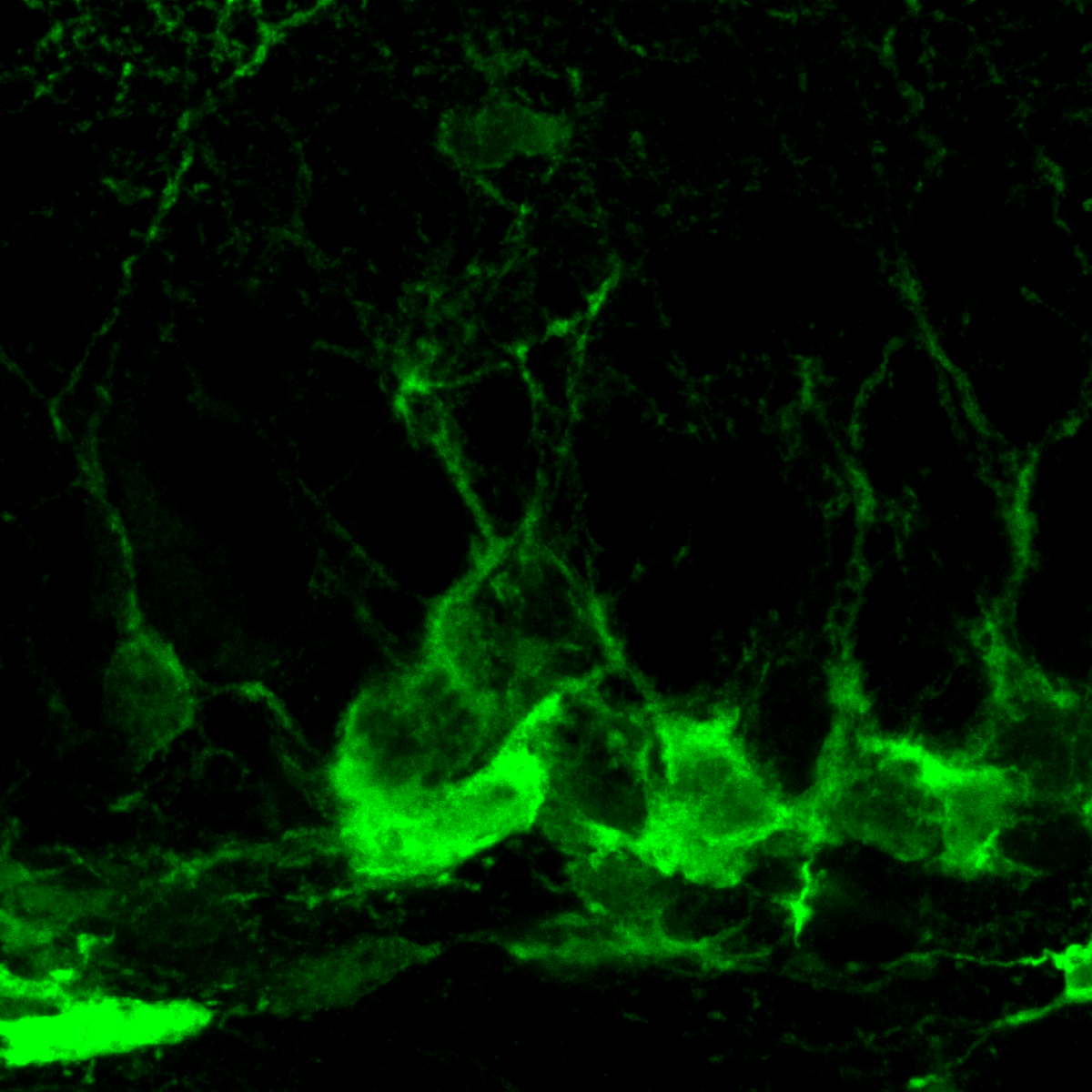

Supplement: Supplementary file 4 — Source Data Fig. 3 [file 44318_2023_11_MOESM4_ESM.zip › EMBOJ-2023-113564_SourceDataForFigure3/3A/P14/CloseUp KO P14 GCL_Nestin.tiff]

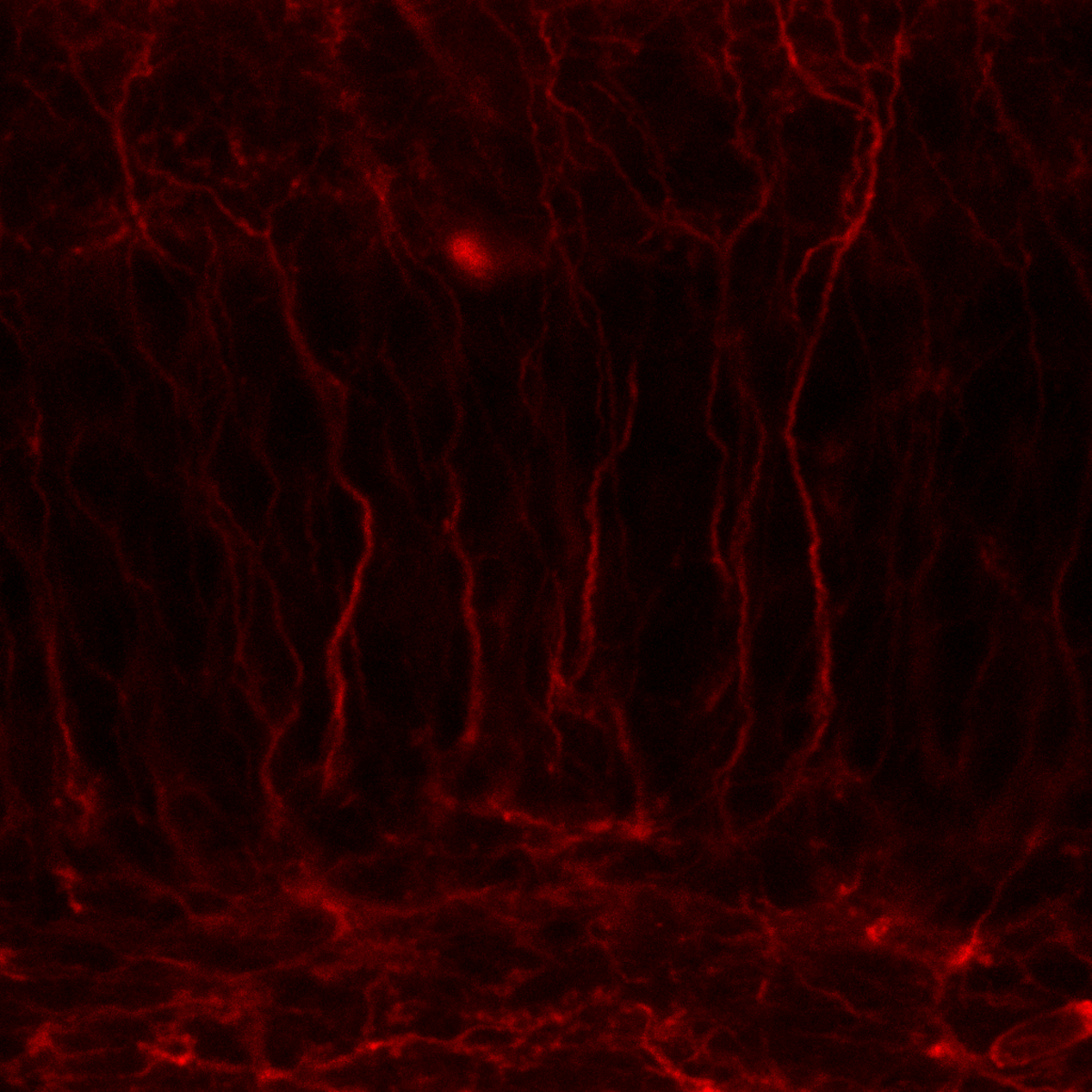

Supplement: Supplementary file 4 — Source Data Fig. 3 [file 44318_2023_11_MOESM4_ESM.zip › EMBOJ-2023-113564_SourceDataForFigure3/3A/P14/CloseUp WT P14 GCL_GFAP.tiff]

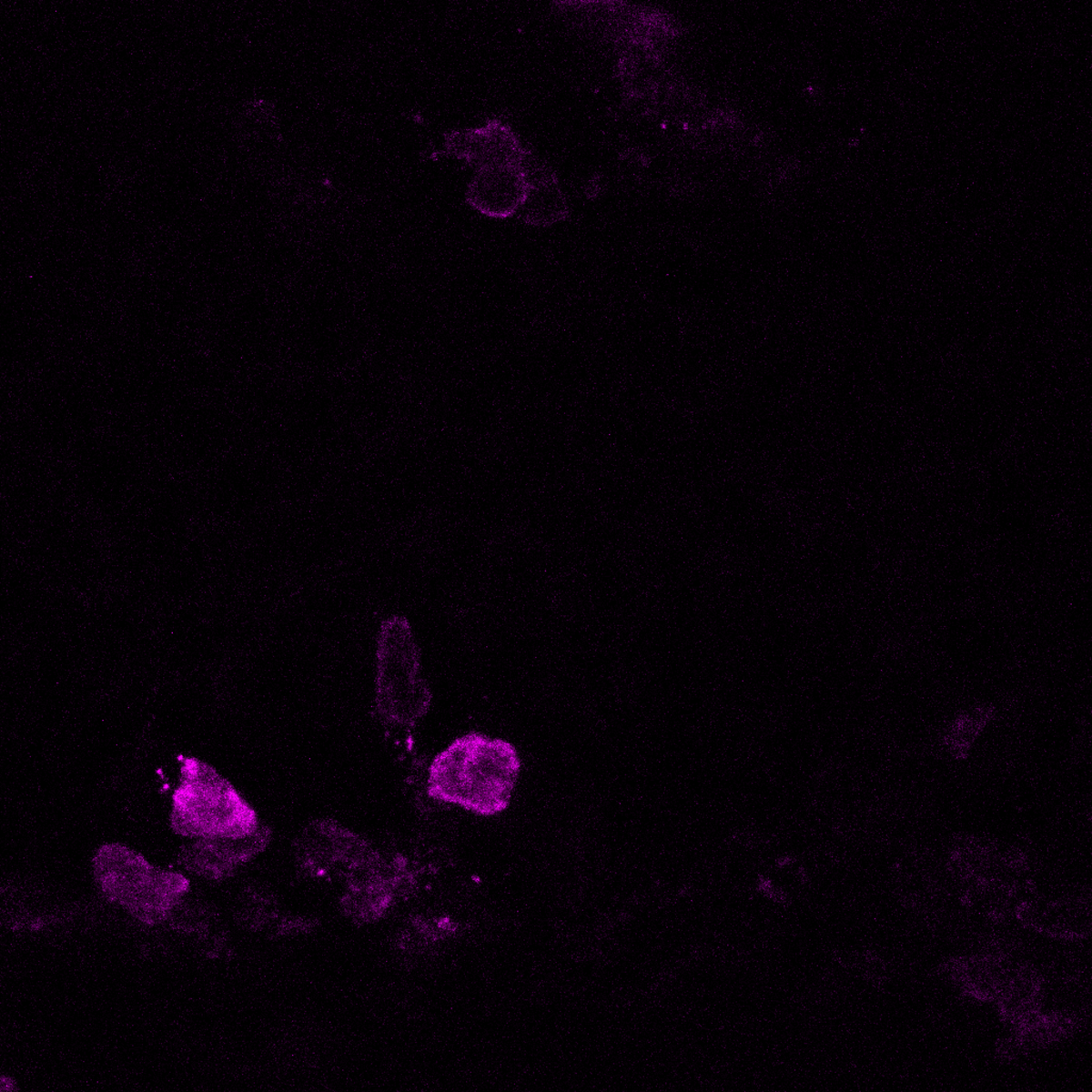

Supplement: Supplementary file 4 — Source Data Fig. 3 [file 44318_2023_11_MOESM4_ESM.zip › EMBOJ-2023-113564_SourceDataForFigure3/3A/P14/CloseUp WT P14 GCL_Ki67.tiff]

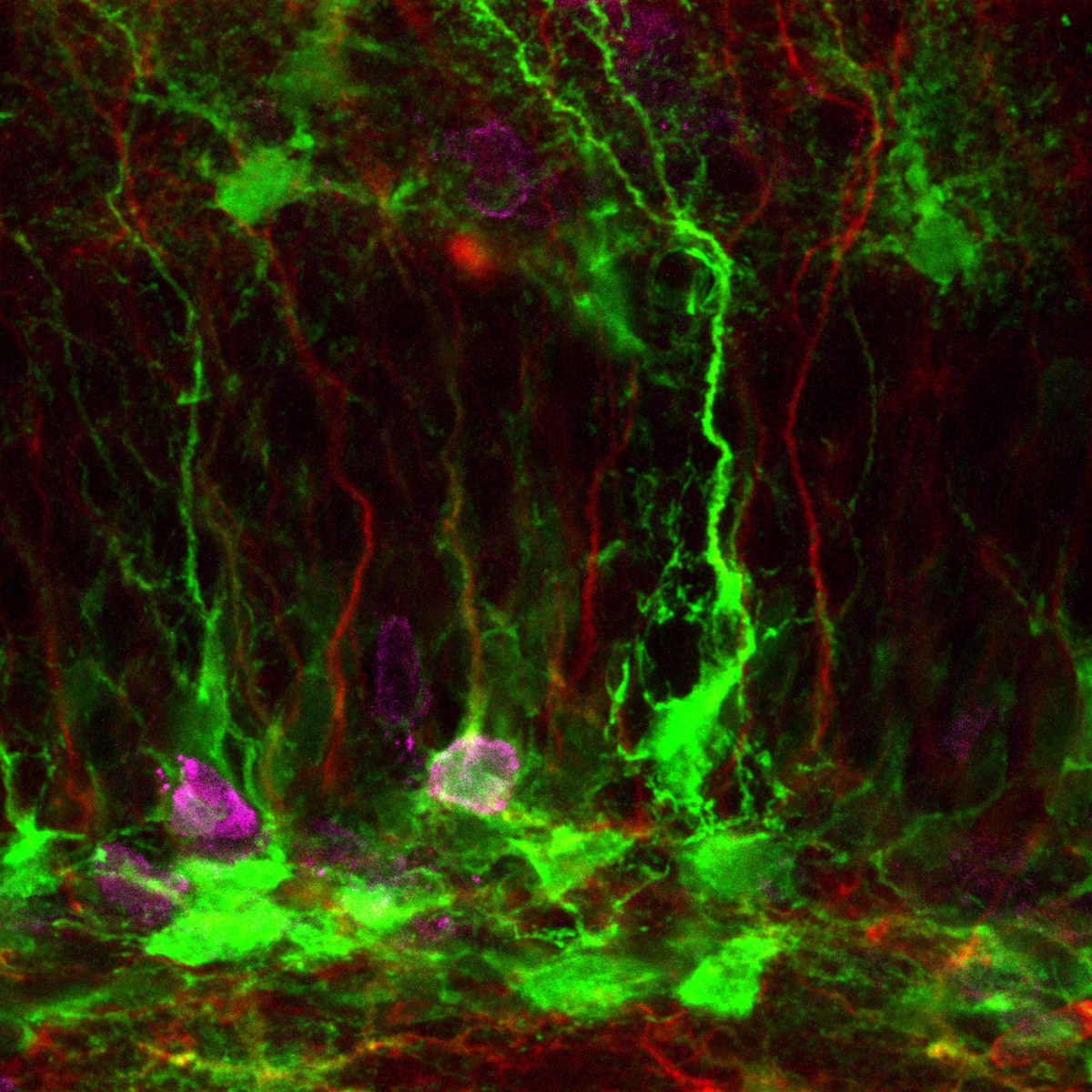

Supplement: Supplementary file 4 — Source Data Fig. 3 [file 44318_2023_11_MOESM4_ESM.zip › EMBOJ-2023-113564_SourceDataForFigure3/3A/P14/CloseUp WT P14 GCL_Merge.tiff]

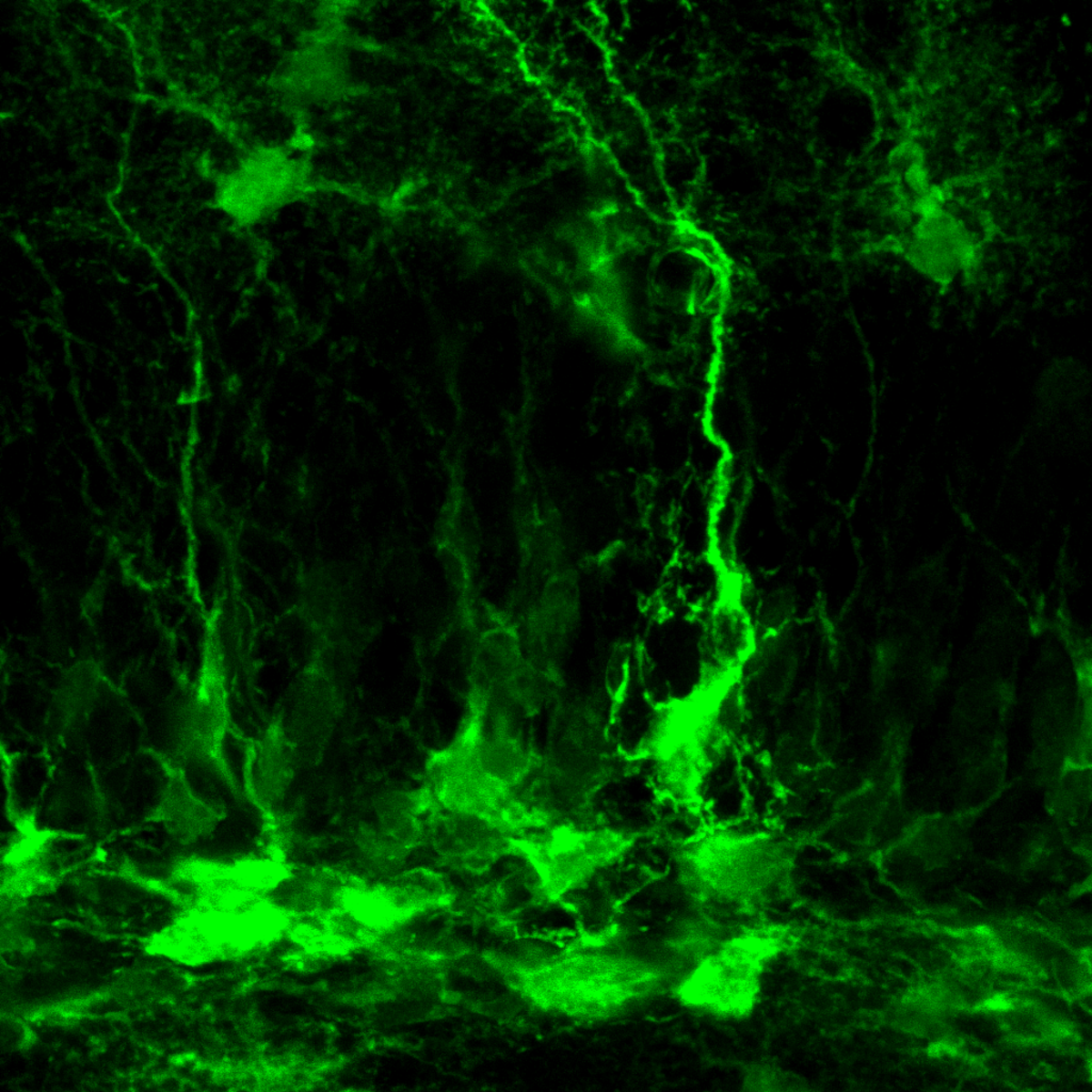

Supplement: Supplementary file 4 — Source Data Fig. 3 [file 44318_2023_11_MOESM4_ESM.zip › EMBOJ-2023-113564_SourceDataForFigure3/3A/P14/CloseUp WT P14 GCL_Nestin.tiff]

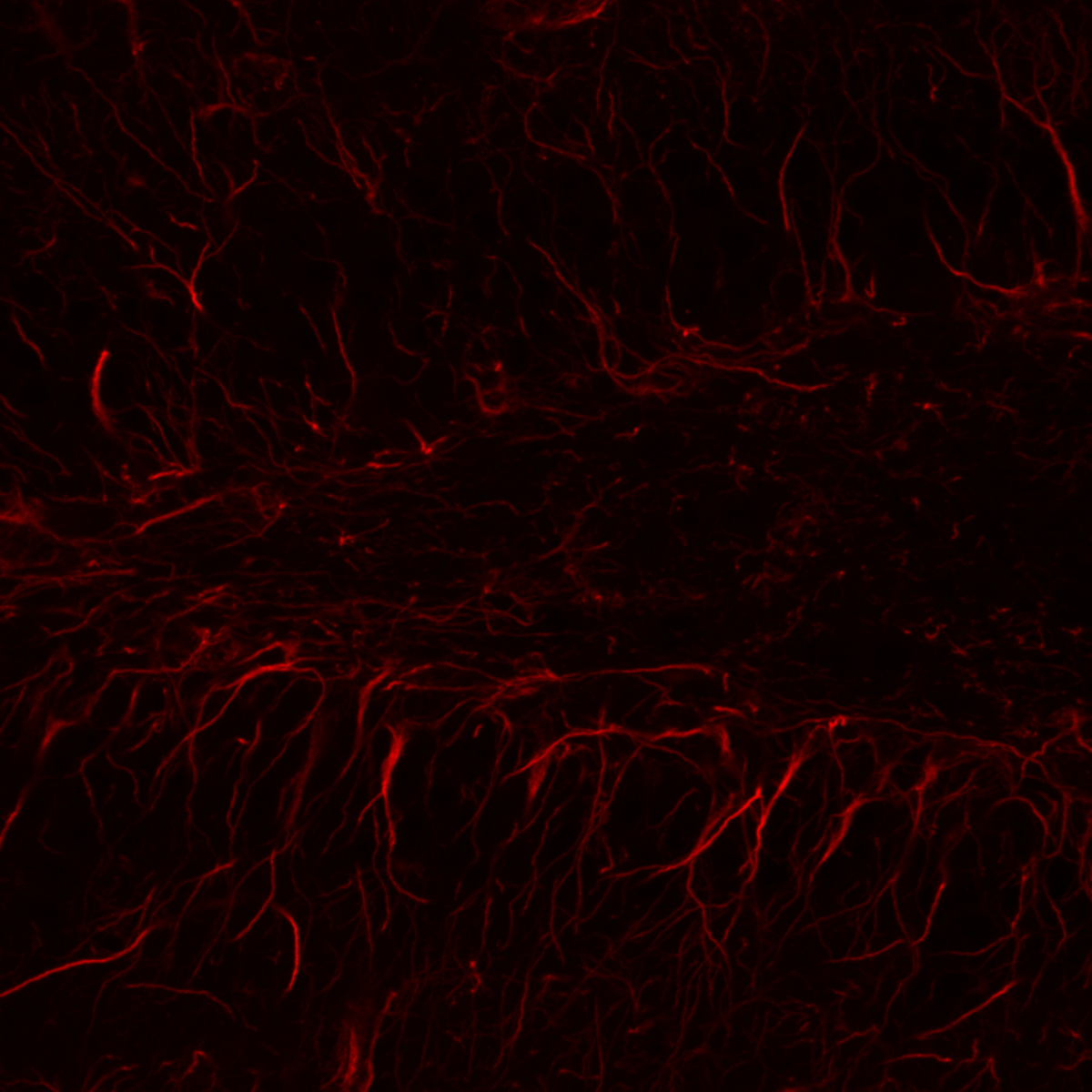

Supplement: Supplementary file 4 — Source Data Fig. 3 [file 44318_2023_11_MOESM4_ESM.zip › EMBOJ-2023-113564_SourceDataForFigure3/3A/P14/KO P14 Dentate Gyrus_GFAP.tiff]

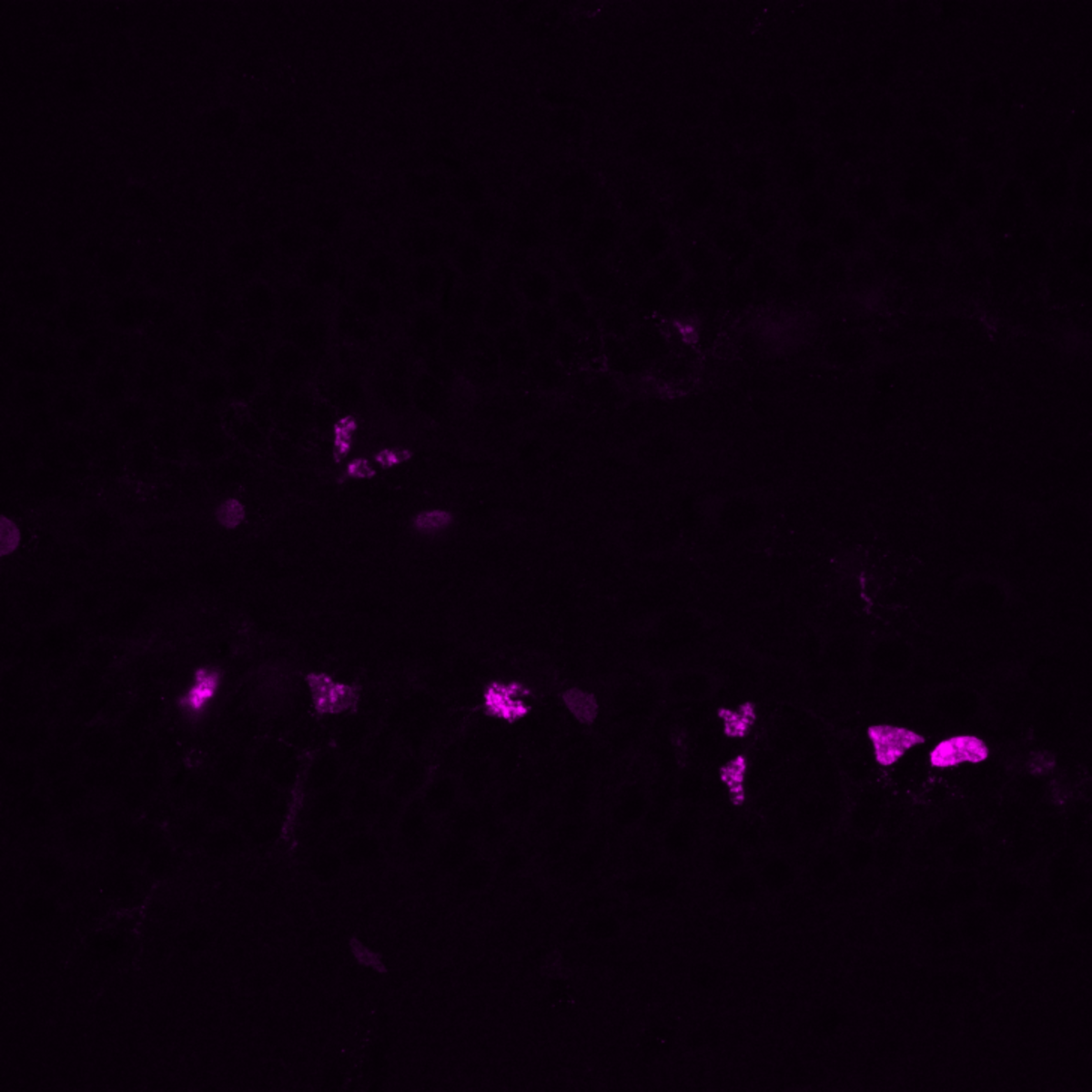

Supplement: Supplementary file 4 — Source Data Fig. 3 [file 44318_2023_11_MOESM4_ESM.zip › EMBOJ-2023-113564_SourceDataForFigure3/3A/P14/KO P14 Dentate Gyrus_Ki67.tiff]

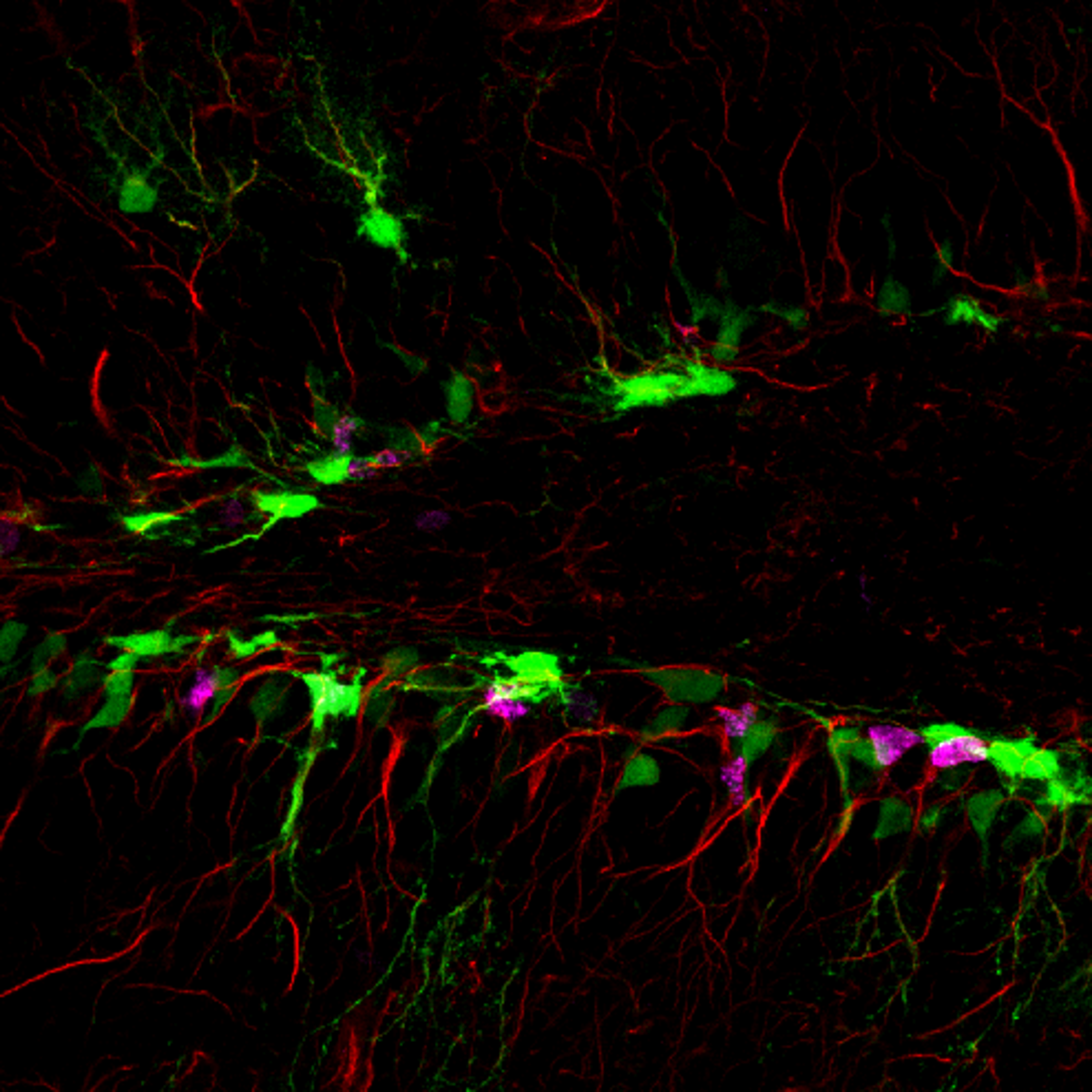

Supplement: Supplementary file 4 — Source Data Fig. 3 [file 44318_2023_11_MOESM4_ESM.zip › EMBOJ-2023-113564_SourceDataForFigure3/3A/P14/KO P14 Dentate Gyrus_Merge.tiff]

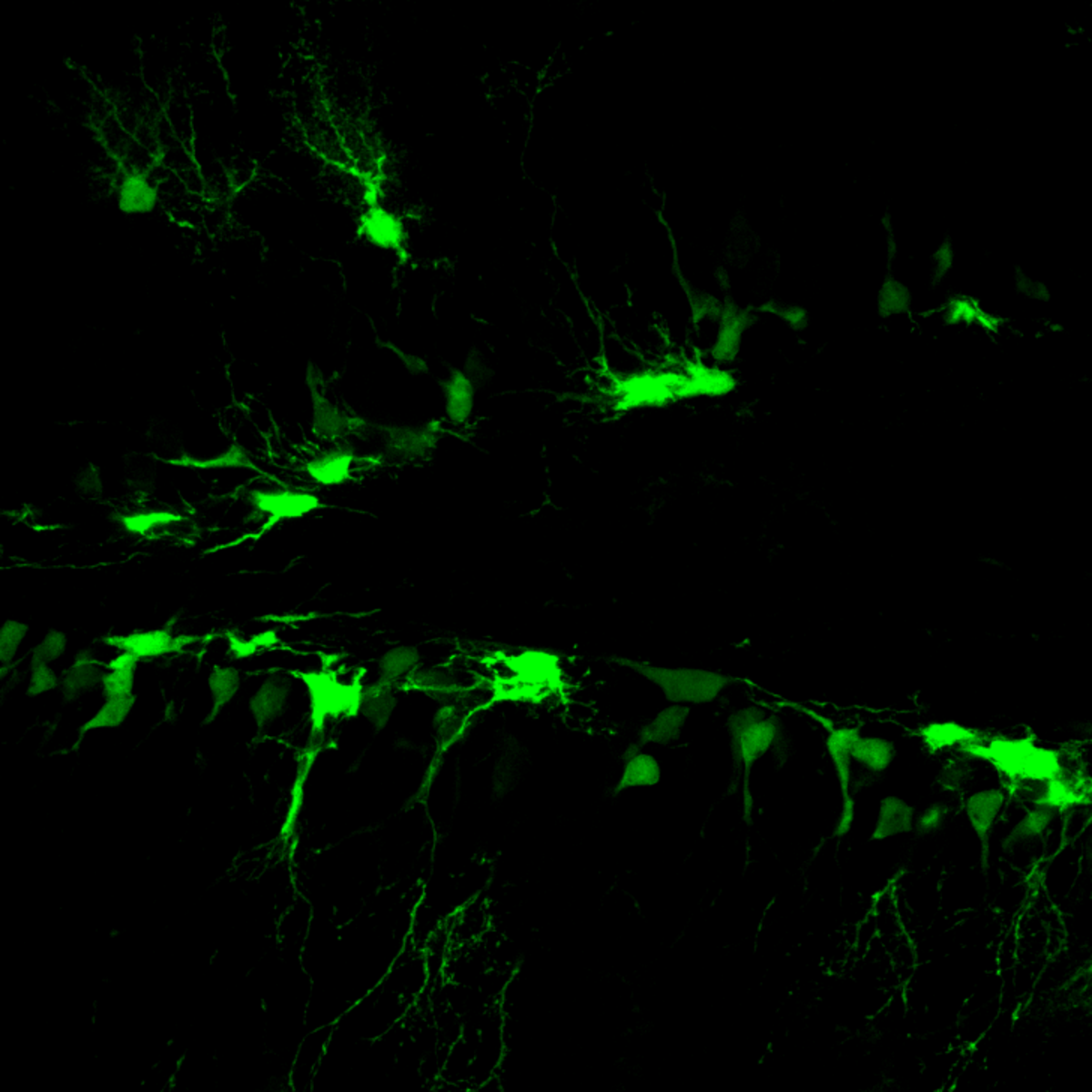

Supplement: Supplementary file 4 — Source Data Fig. 3 [file 44318_2023_11_MOESM4_ESM.zip › EMBOJ-2023-113564_SourceDataForFigure3/3A/P14/KO P14 Dentate Gyrus_Nestin.tiff]

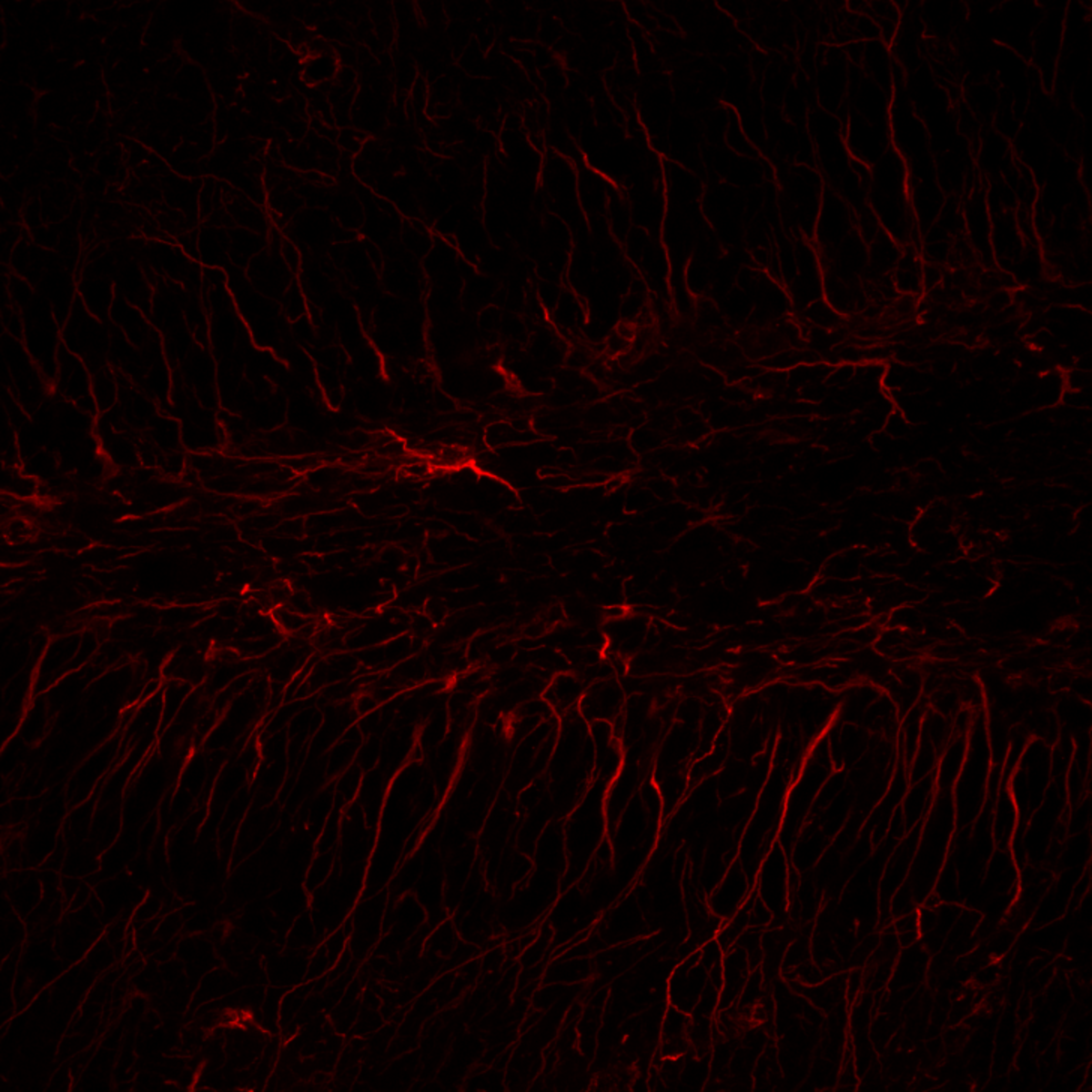

Supplement: Supplementary file 4 — Source Data Fig. 3 [file 44318_2023_11_MOESM4_ESM.zip › EMBOJ-2023-113564_SourceDataForFigure3/3A/P14/WT P14 Dentate Gyrus_GFAP.tiff]

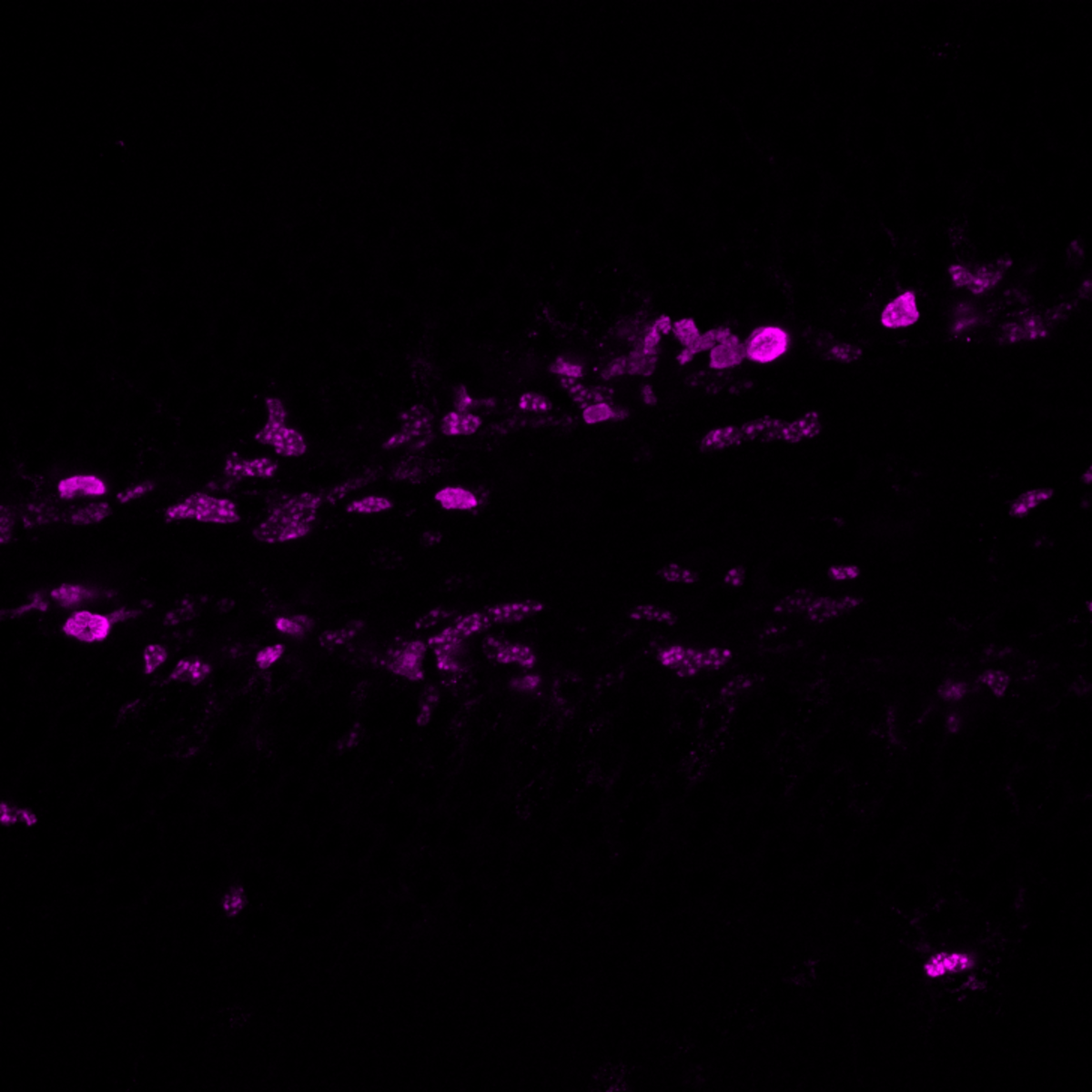

Supplement: Supplementary file 4 — Source Data Fig. 3 [file 44318_2023_11_MOESM4_ESM.zip › EMBOJ-2023-113564_SourceDataForFigure3/3A/P14/WT P14 Dentate Gyrus_Ki67.tiff]

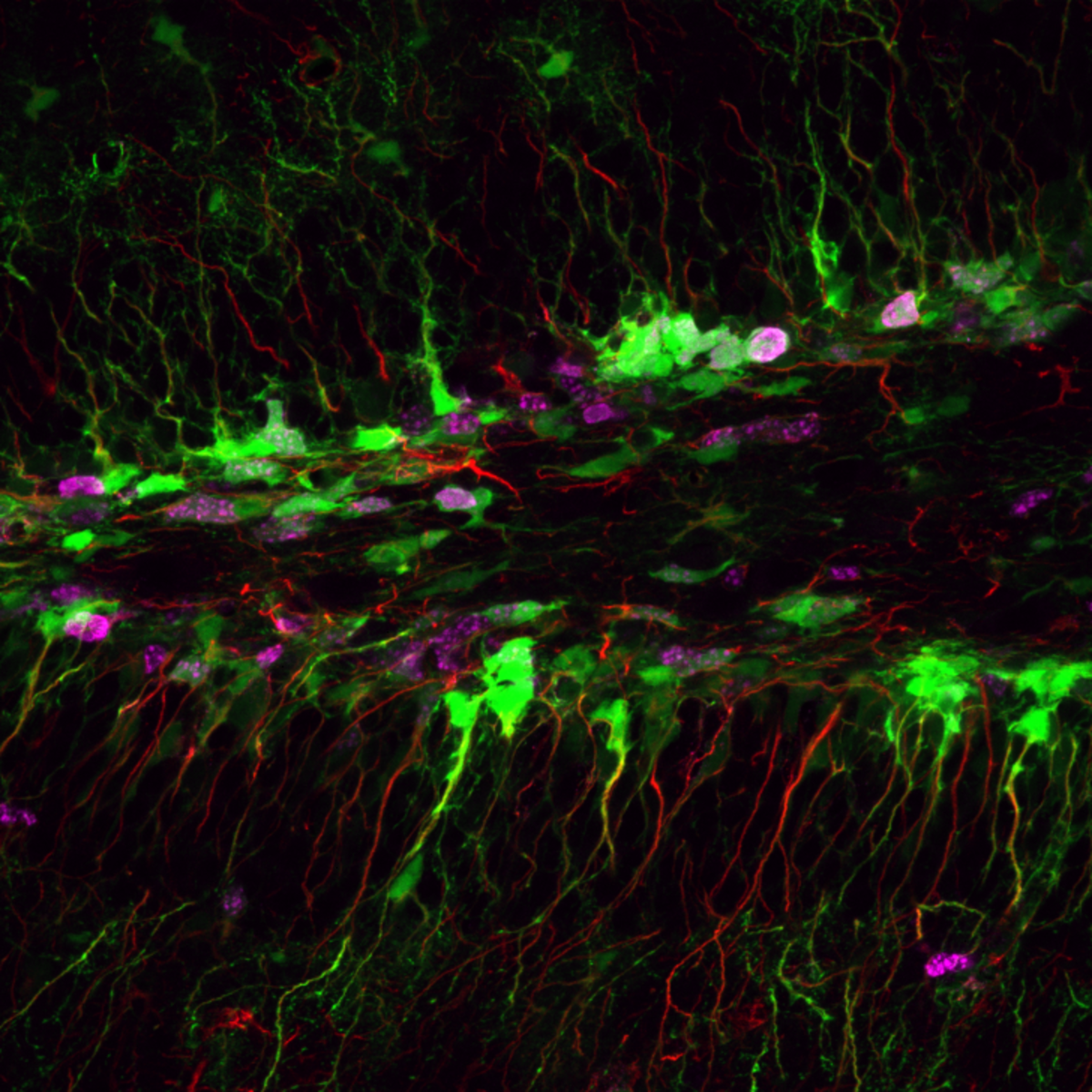

Supplement: Supplementary file 4 — Source Data Fig. 3 [file 44318_2023_11_MOESM4_ESM.zip › EMBOJ-2023-113564_SourceDataForFigure3/3A/P14/WT P14 Dentate Gyrus_Merge.tiff]

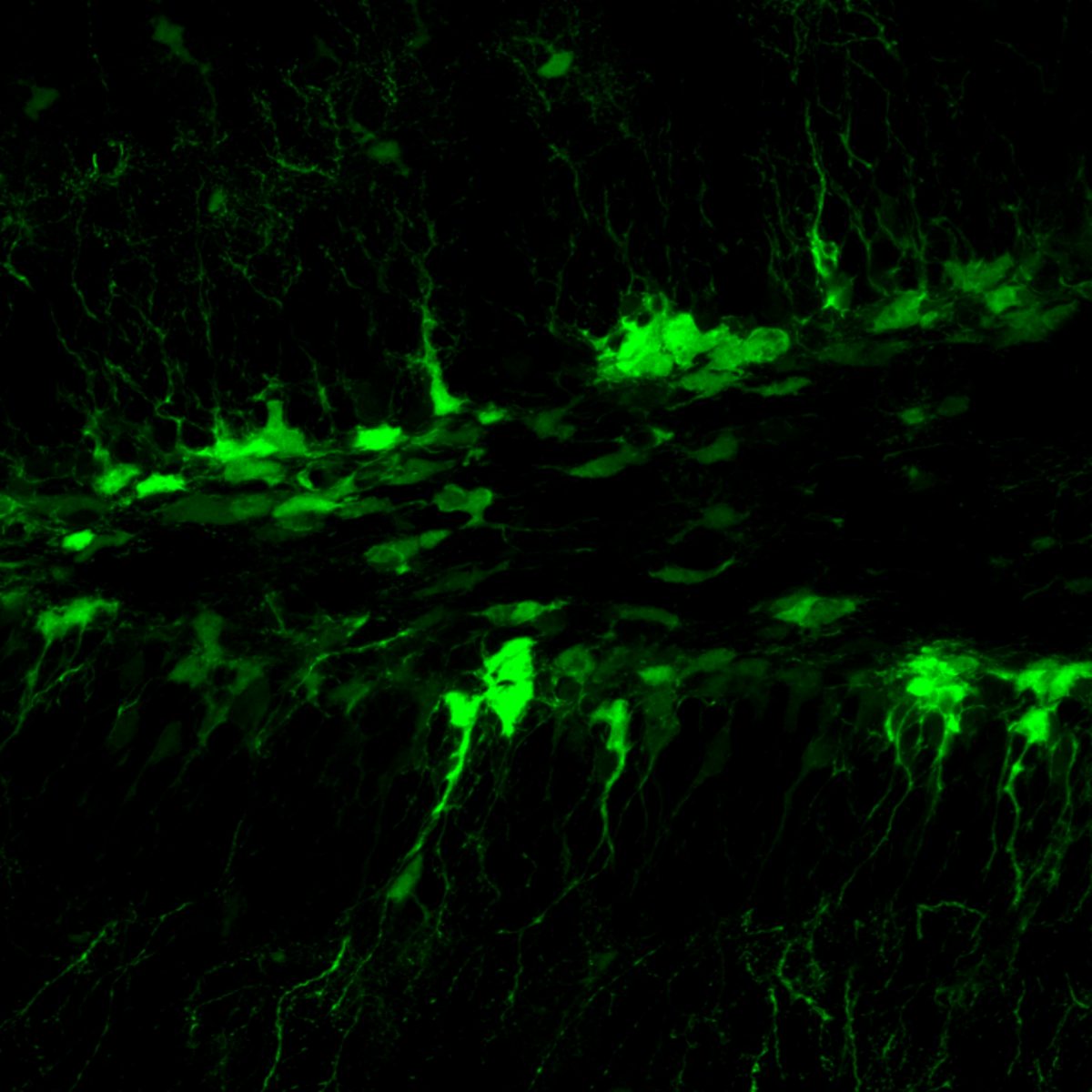

Supplement: Supplementary file 4 — Source Data Fig. 3 [file 44318_2023_11_MOESM4_ESM.zip › EMBOJ-2023-113564_SourceDataForFigure3/3A/P14/WT P14 Dentate Gyrus_Nestin.tiff]

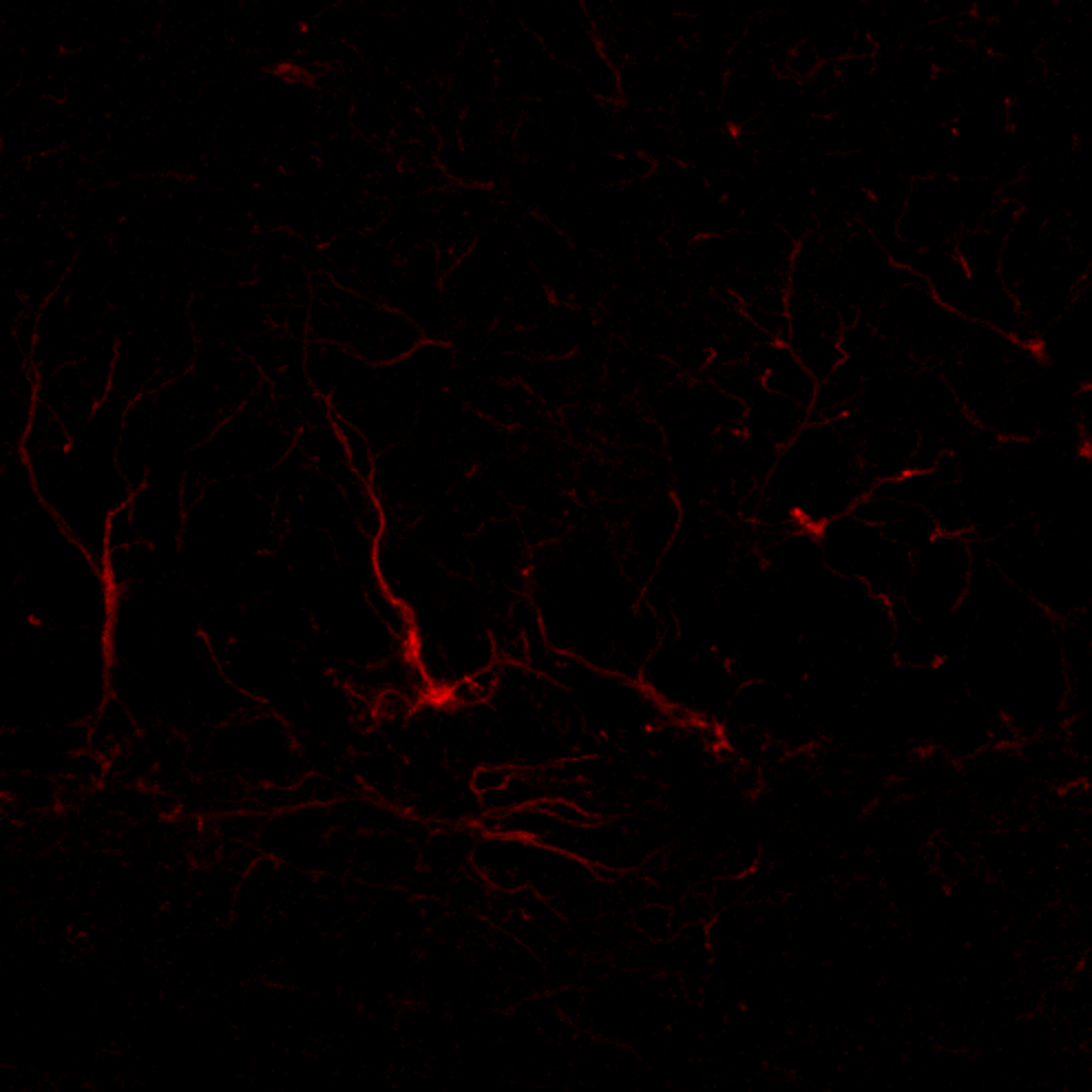

Supplement: Supplementary file 4 — Source Data Fig. 3 [file 44318_2023_11_MOESM4_ESM.zip › EMBOJ-2023-113564_SourceDataForFigure3/3A/P28/CloseUp KO P28 GCL_GFAP.tiff]

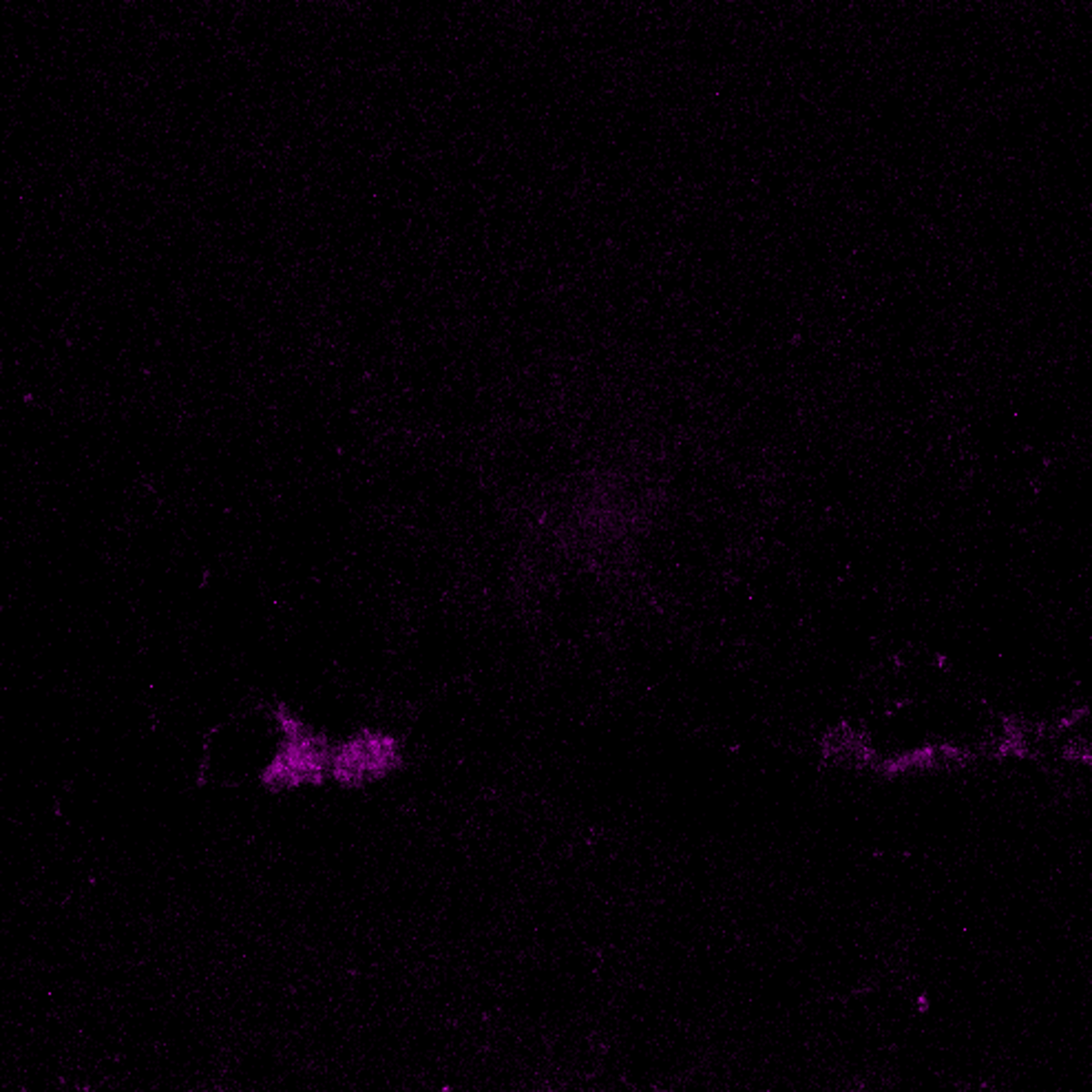

Supplement: Supplementary file 4 — Source Data Fig. 3 [file 44318_2023_11_MOESM4_ESM.zip › EMBOJ-2023-113564_SourceDataForFigure3/3A/P28/CloseUp KO P28 GCL_Ki67.tiff]

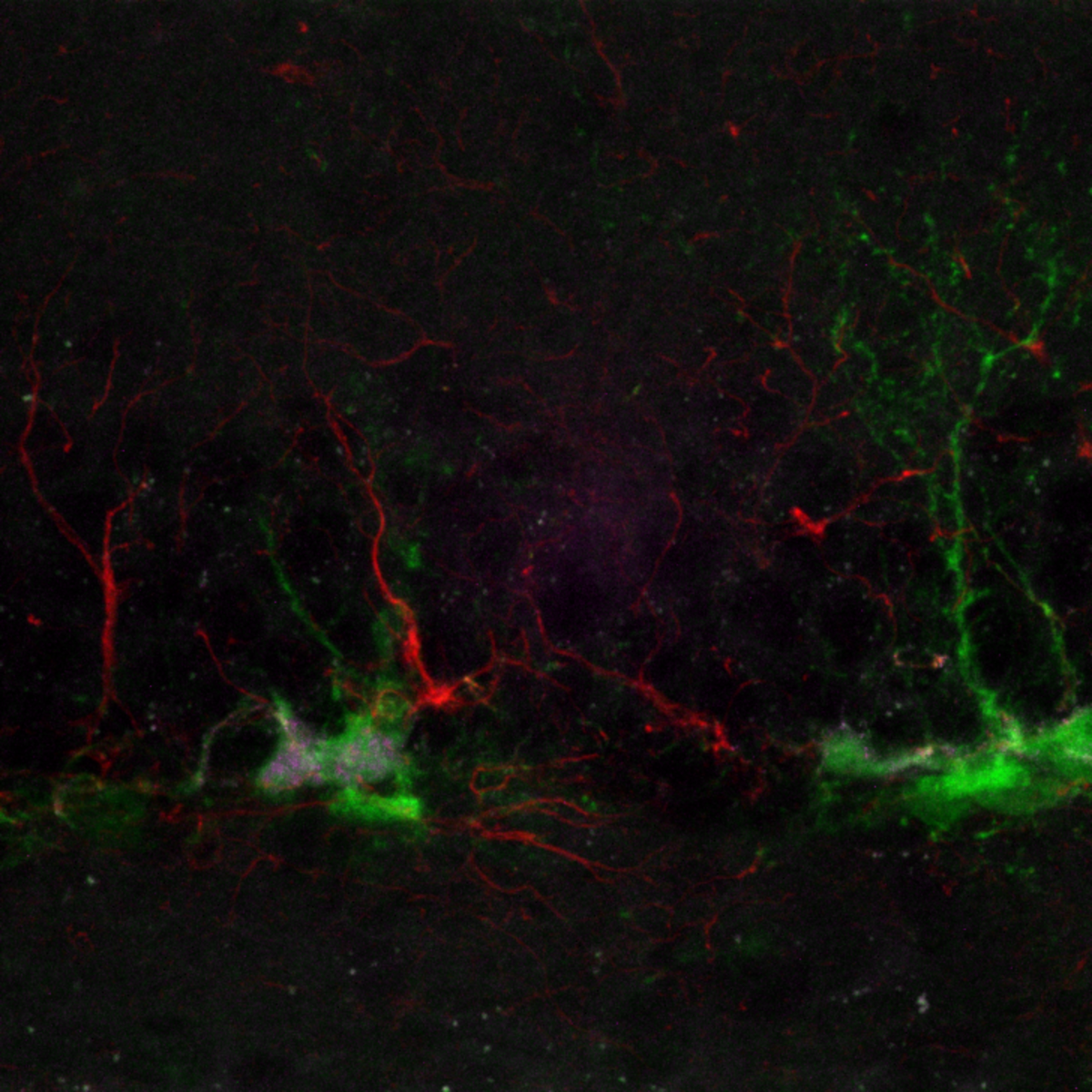

Supplement: Supplementary file 4 — Source Data Fig. 3 [file 44318_2023_11_MOESM4_ESM.zip › EMBOJ-2023-113564_SourceDataForFigure3/3A/P28/CloseUp KO P28 GCL_Merge.tiff]

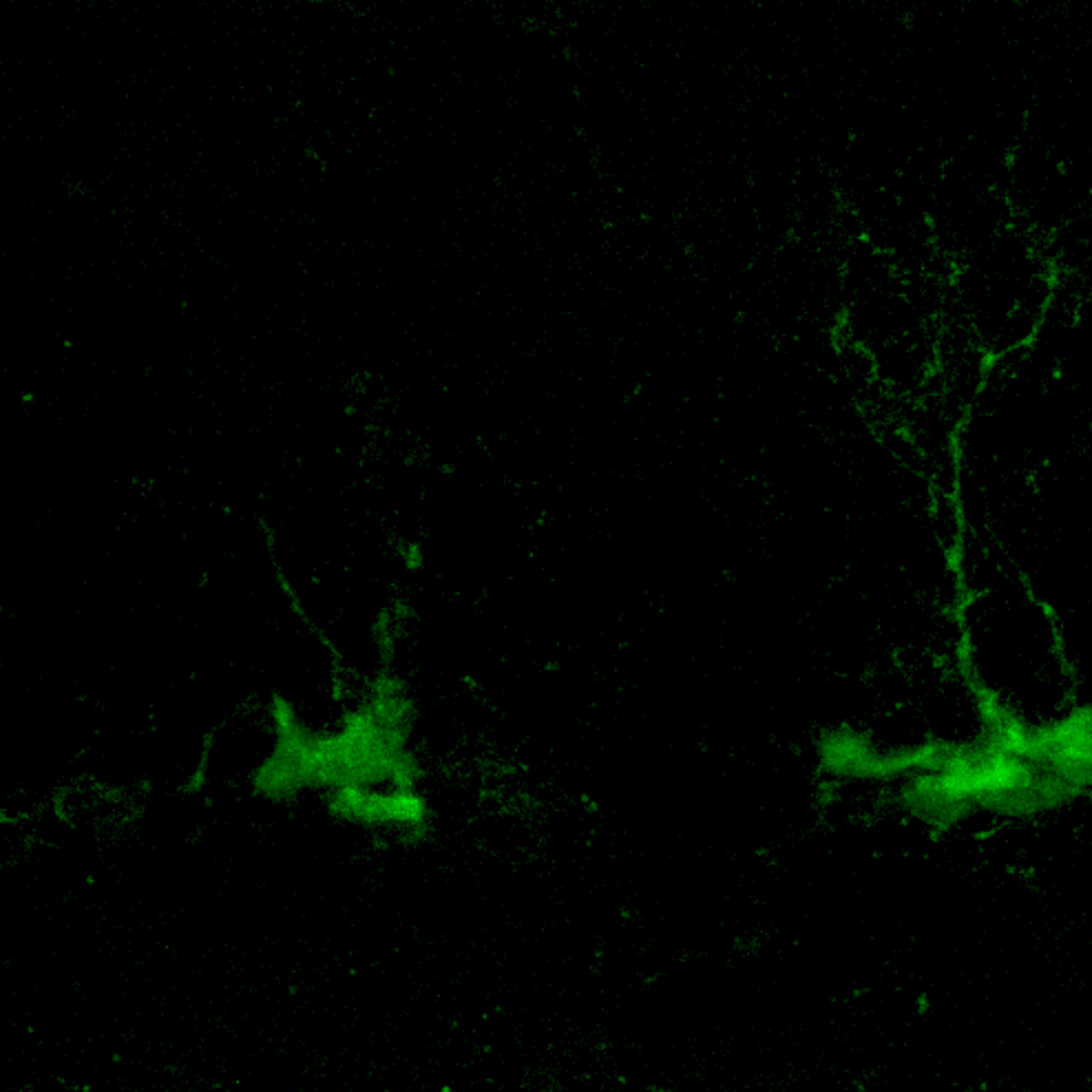

Supplement: Supplementary file 4 — Source Data Fig. 3 [file 44318_2023_11_MOESM4_ESM.zip › EMBOJ-2023-113564_SourceDataForFigure3/3A/P28/CloseUp KO P28 GCL_Nestin.tiff]

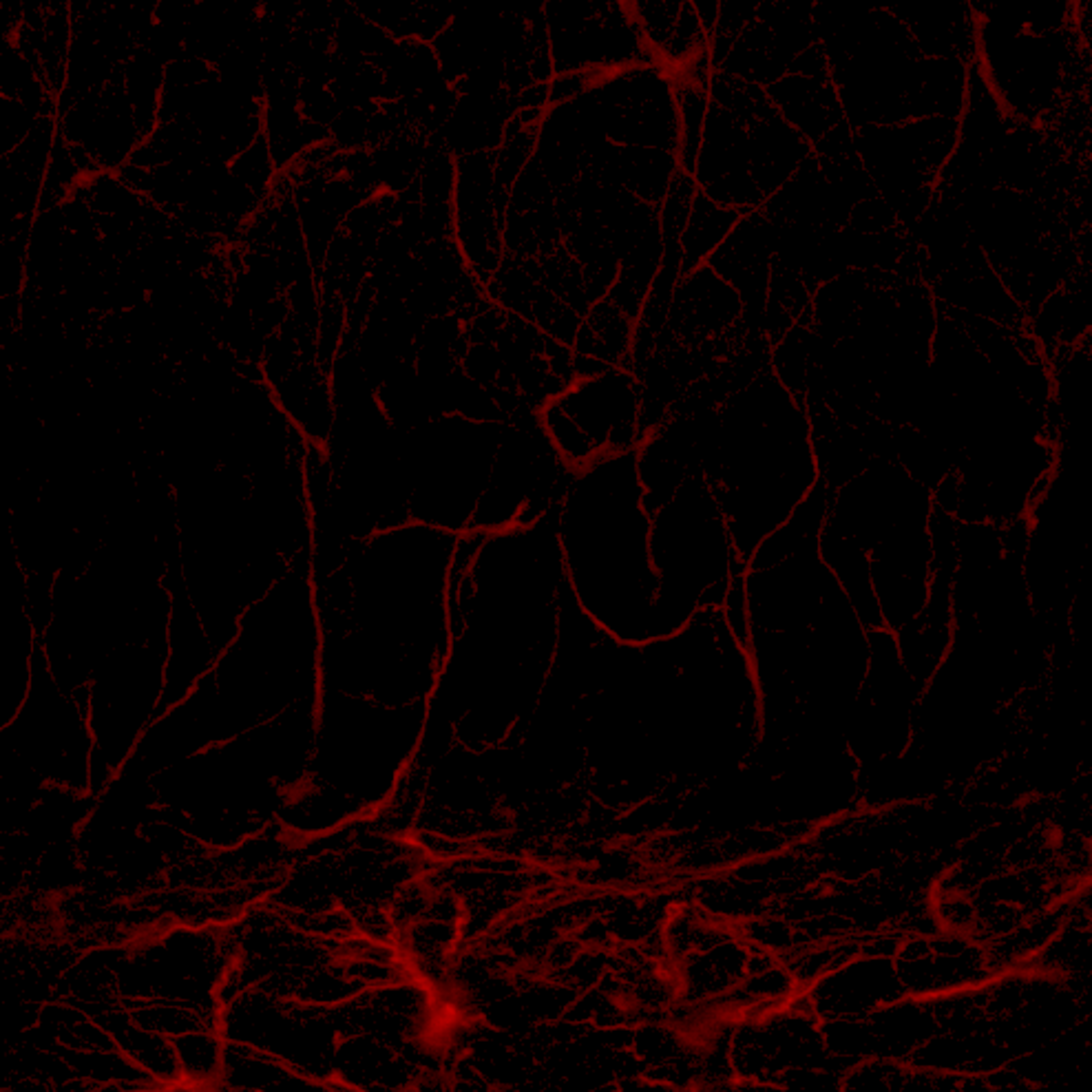

Supplement: Supplementary file 4 — Source Data Fig. 3 [file 44318_2023_11_MOESM4_ESM.zip › EMBOJ-2023-113564_SourceDataForFigure3/3A/P28/CloseUp WT P28 GCL_GFAP.tiff]

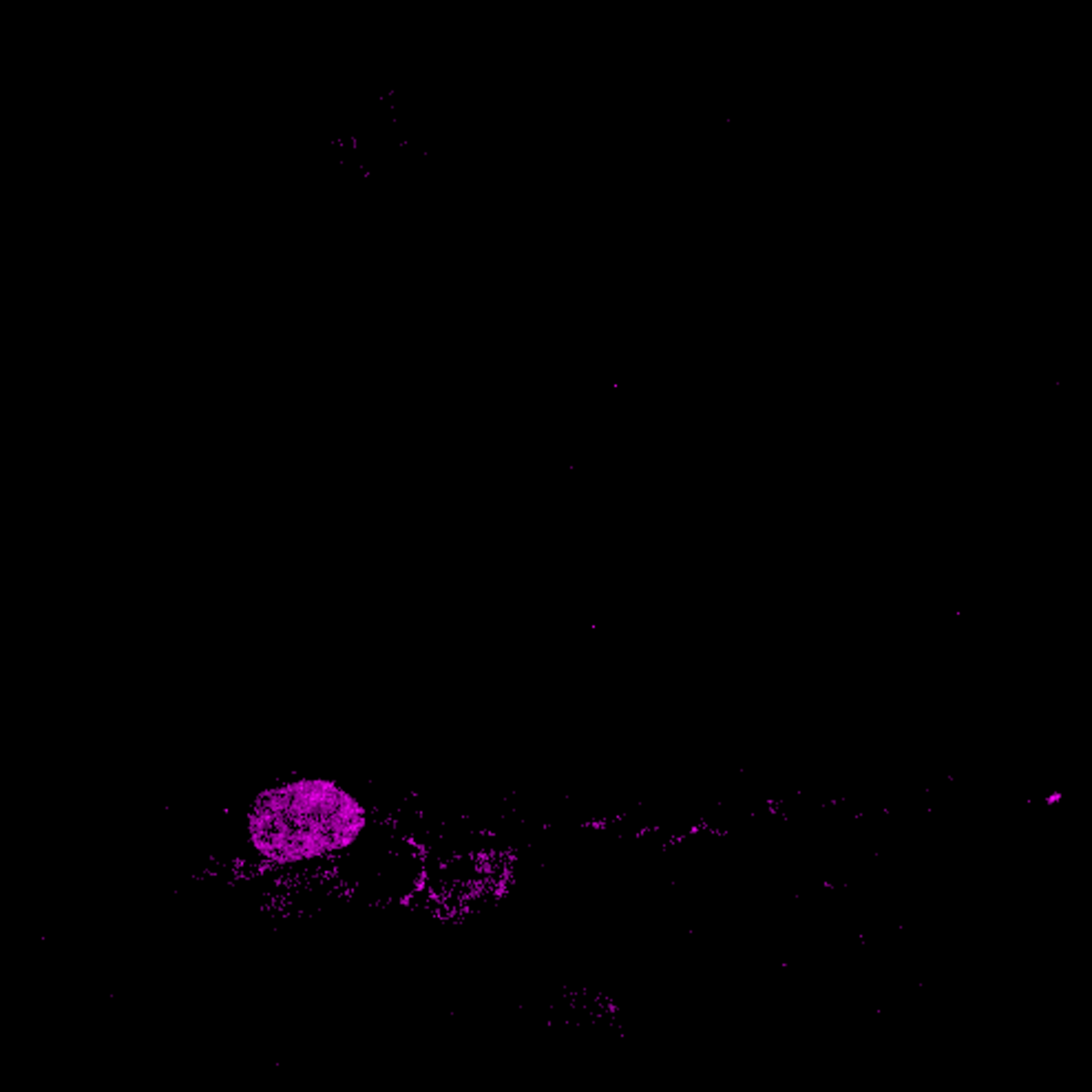

Supplement: Supplementary file 4 — Source Data Fig. 3 [file 44318_2023_11_MOESM4_ESM.zip › EMBOJ-2023-113564_SourceDataForFigure3/3A/P28/CloseUp WT P28 GCL_Ki67.tiff]
