# Supplementary figures and images for: Generation of adult hippocampal neural stem cells occurs in the early postnatal dentate gyrus and depends on cyclin D2 (part 2 of 2)
Source: EMBO J. 2023 Dec 20;43(3):1. doi: 10.1038/s44318-023-00011-2 (PMC10897295; doi:10.1038/s44318-023-00011-2)

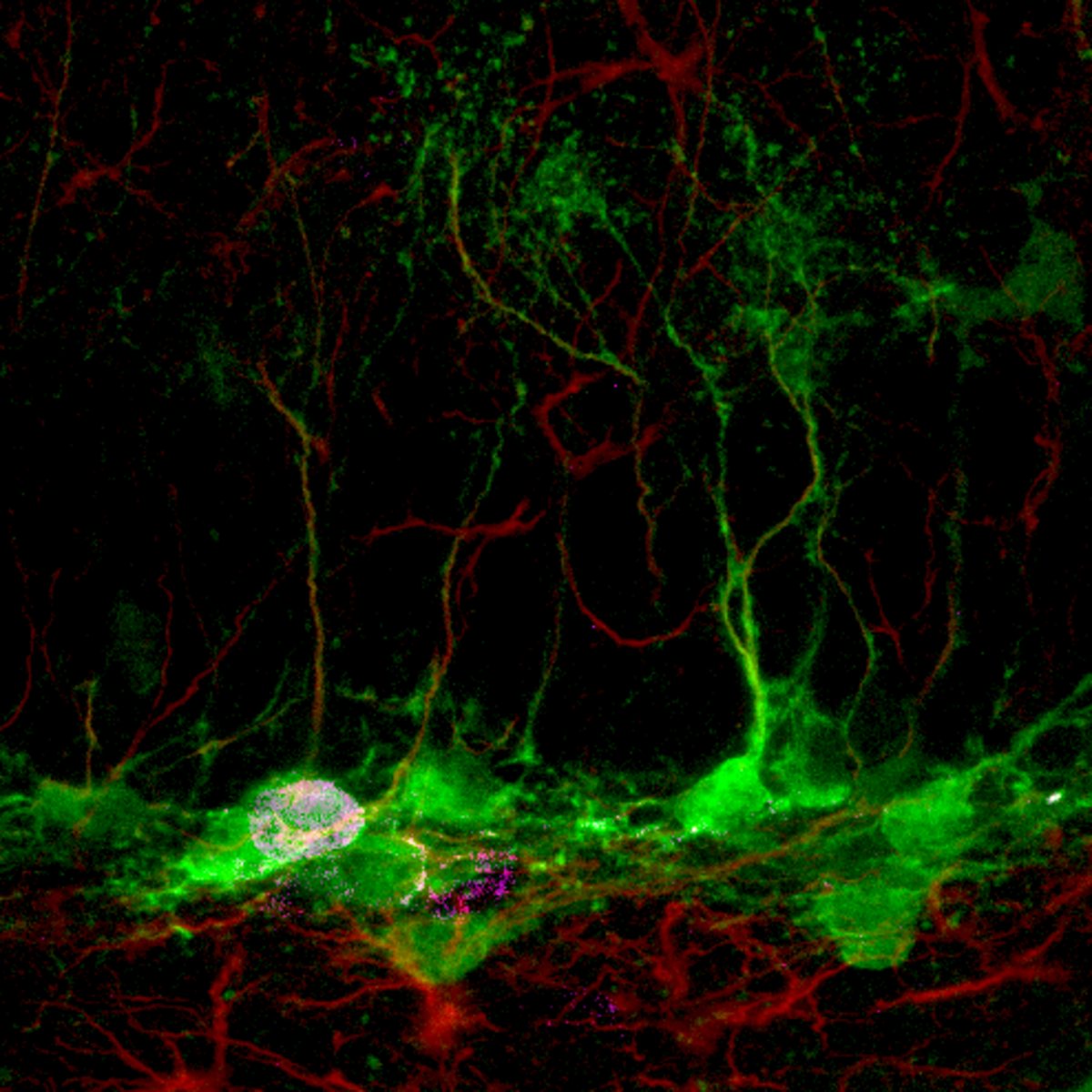

Supplement: Supplementary file 4 — Source Data Fig. 3 [file 44318_2023_11_MOESM4_ESM.zip › EMBOJ-2023-113564_SourceDataForFigure3/3A/P28/CloseUp WT P28 GCL_Merge.tiff]

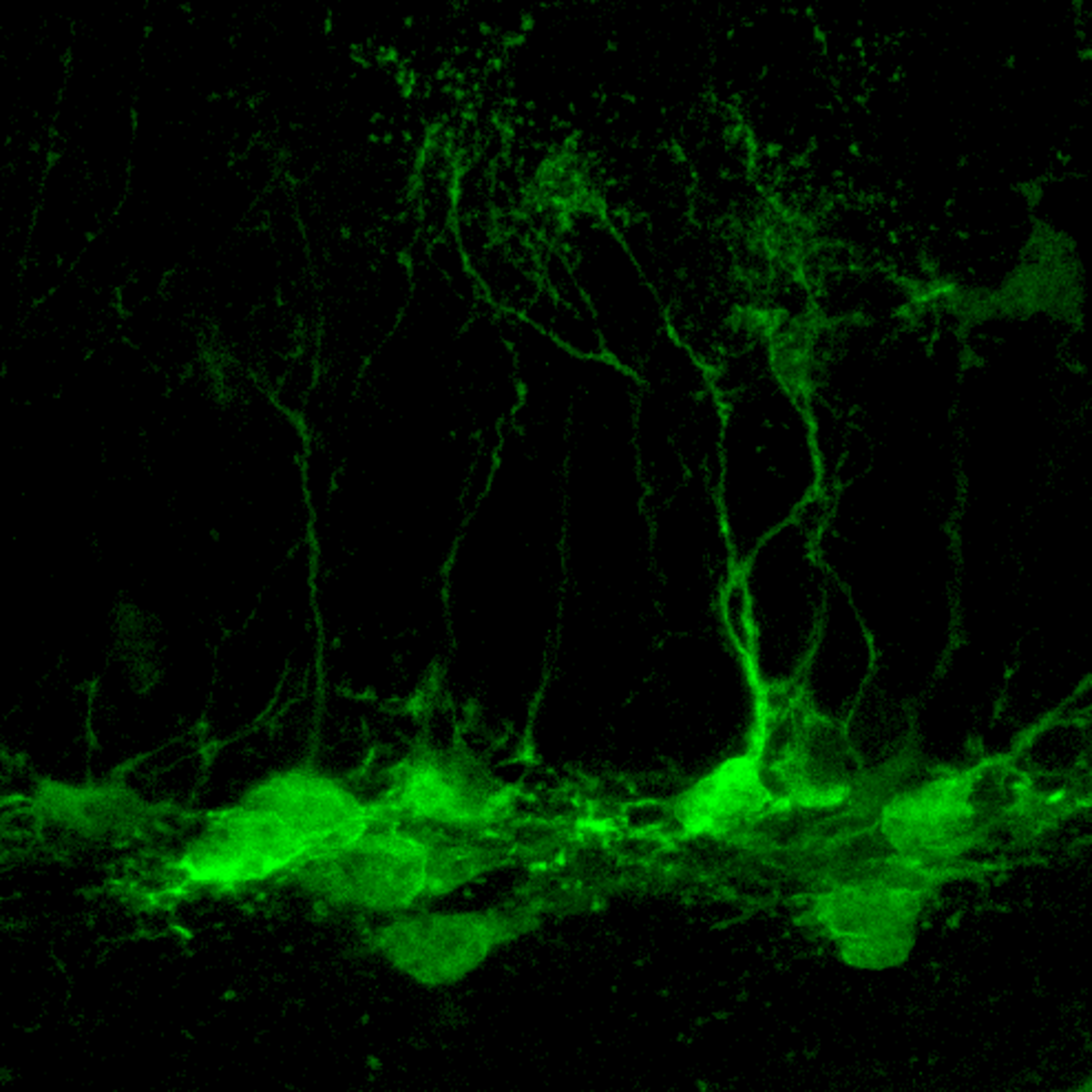

Supplement: Supplementary file 4 — Source Data Fig. 3 [file 44318_2023_11_MOESM4_ESM.zip › EMBOJ-2023-113564_SourceDataForFigure3/3A/P28/CloseUp WT P28 GCL_Nestin.tiff]

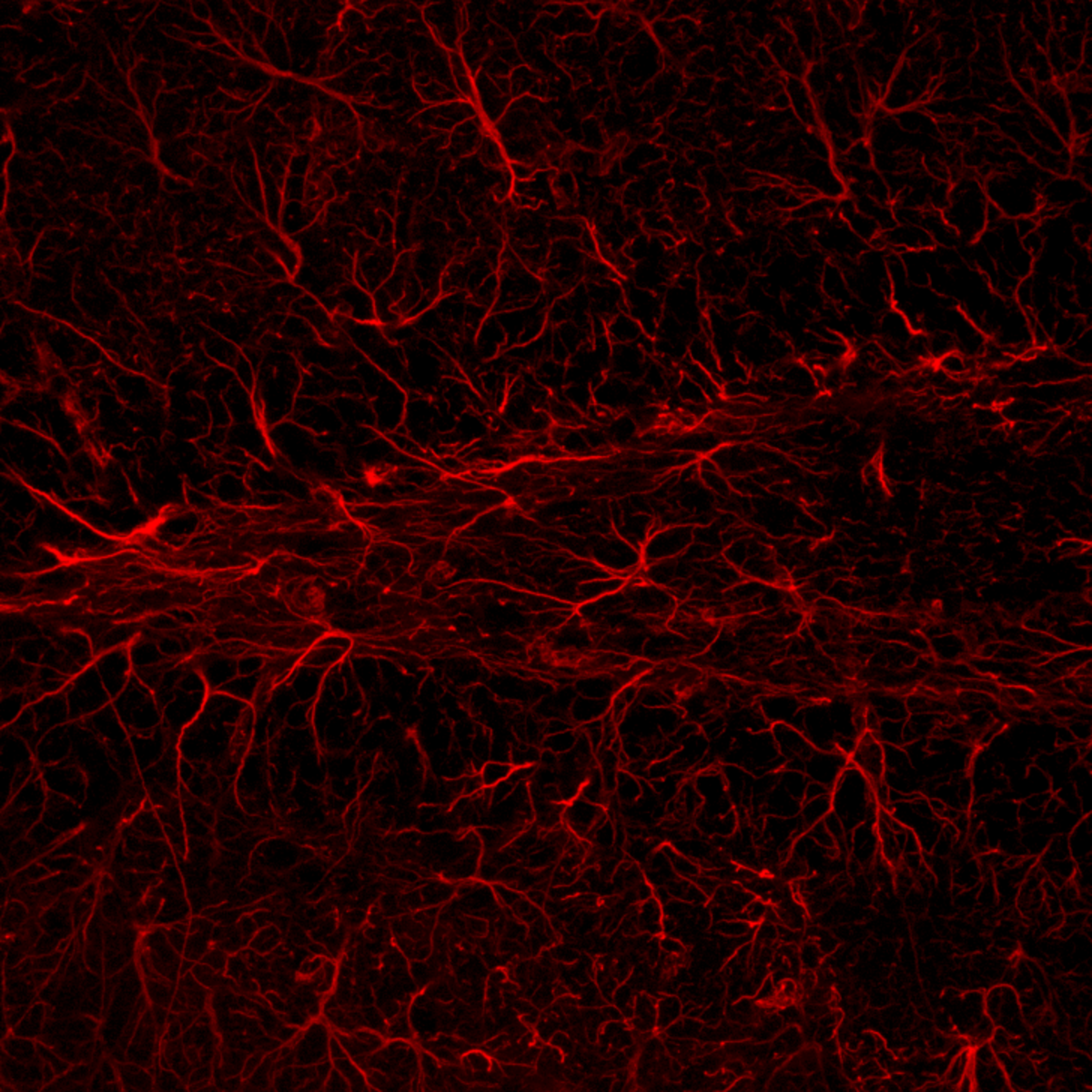

Supplement: Supplementary file 4 — Source Data Fig. 3 [file 44318_2023_11_MOESM4_ESM.zip › EMBOJ-2023-113564_SourceDataForFigure3/3A/P28/KO P28 Dentate Gyrus_GFAP.tiff]

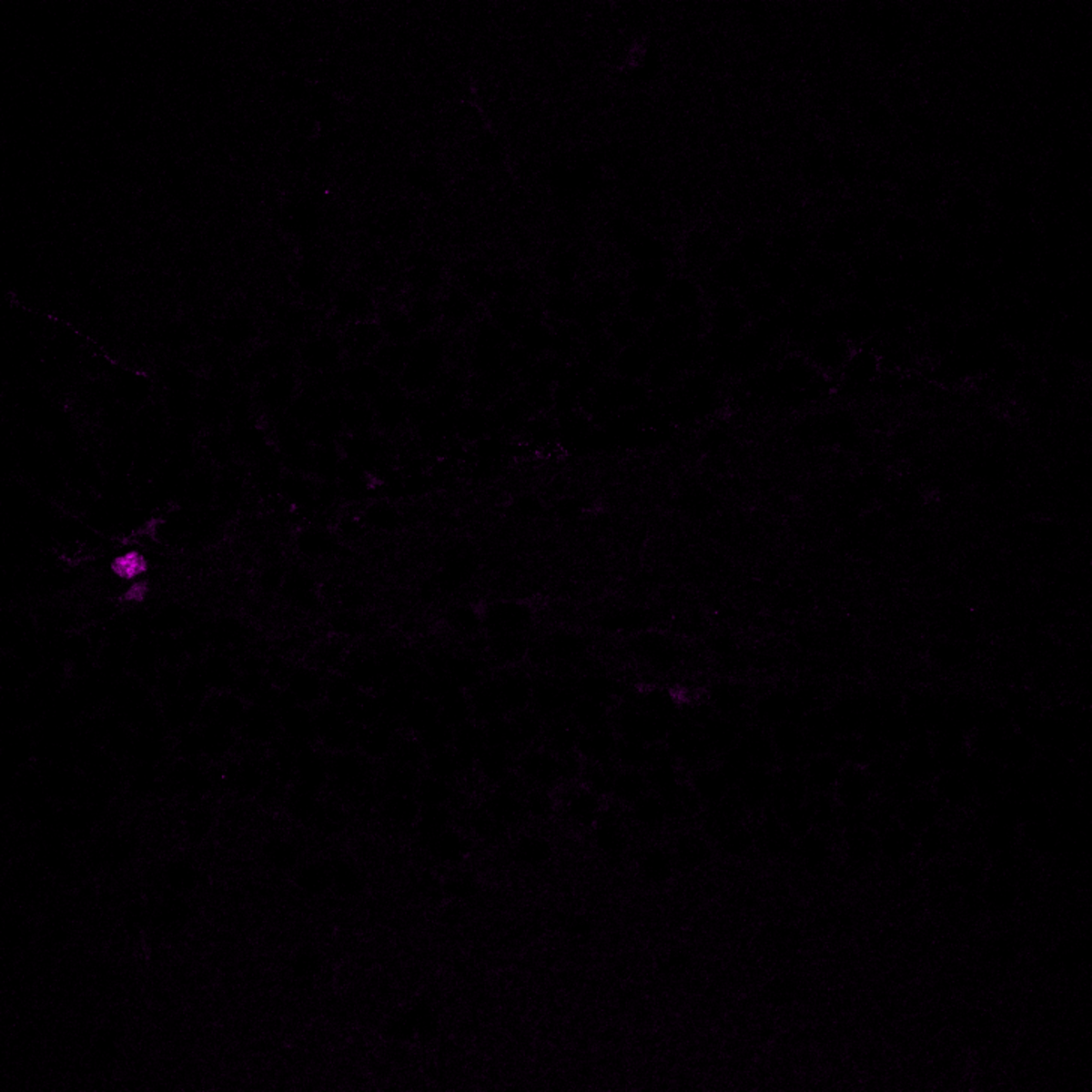

Supplement: Supplementary file 4 — Source Data Fig. 3 [file 44318_2023_11_MOESM4_ESM.zip › EMBOJ-2023-113564_SourceDataForFigure3/3A/P28/KO P28 Dentate Gyrus_Ki67.tiff]

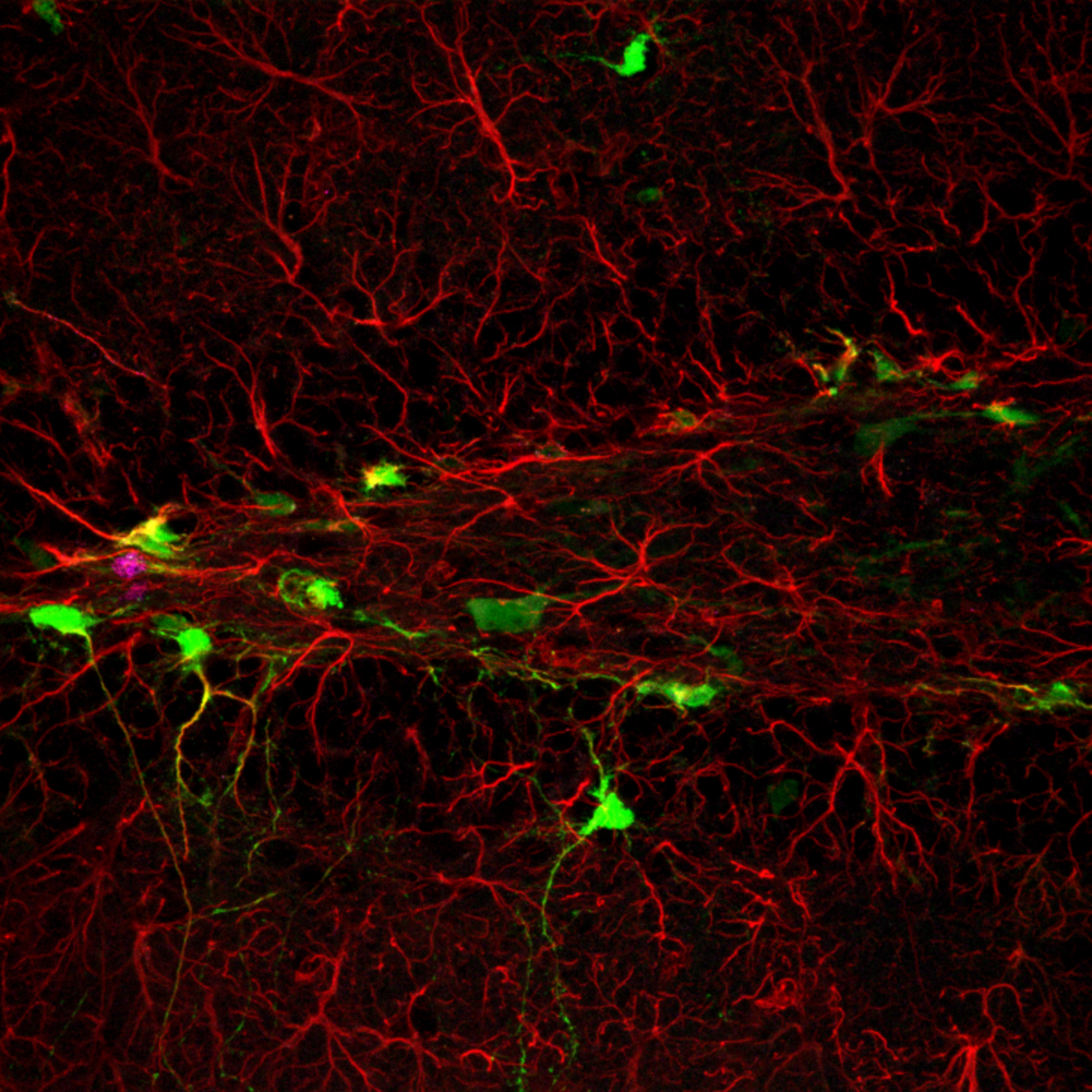

Supplement: Supplementary file 4 — Source Data Fig. 3 [file 44318_2023_11_MOESM4_ESM.zip › EMBOJ-2023-113564_SourceDataForFigure3/3A/P28/KO P28 Dentate Gyrus_Merge.tiff]

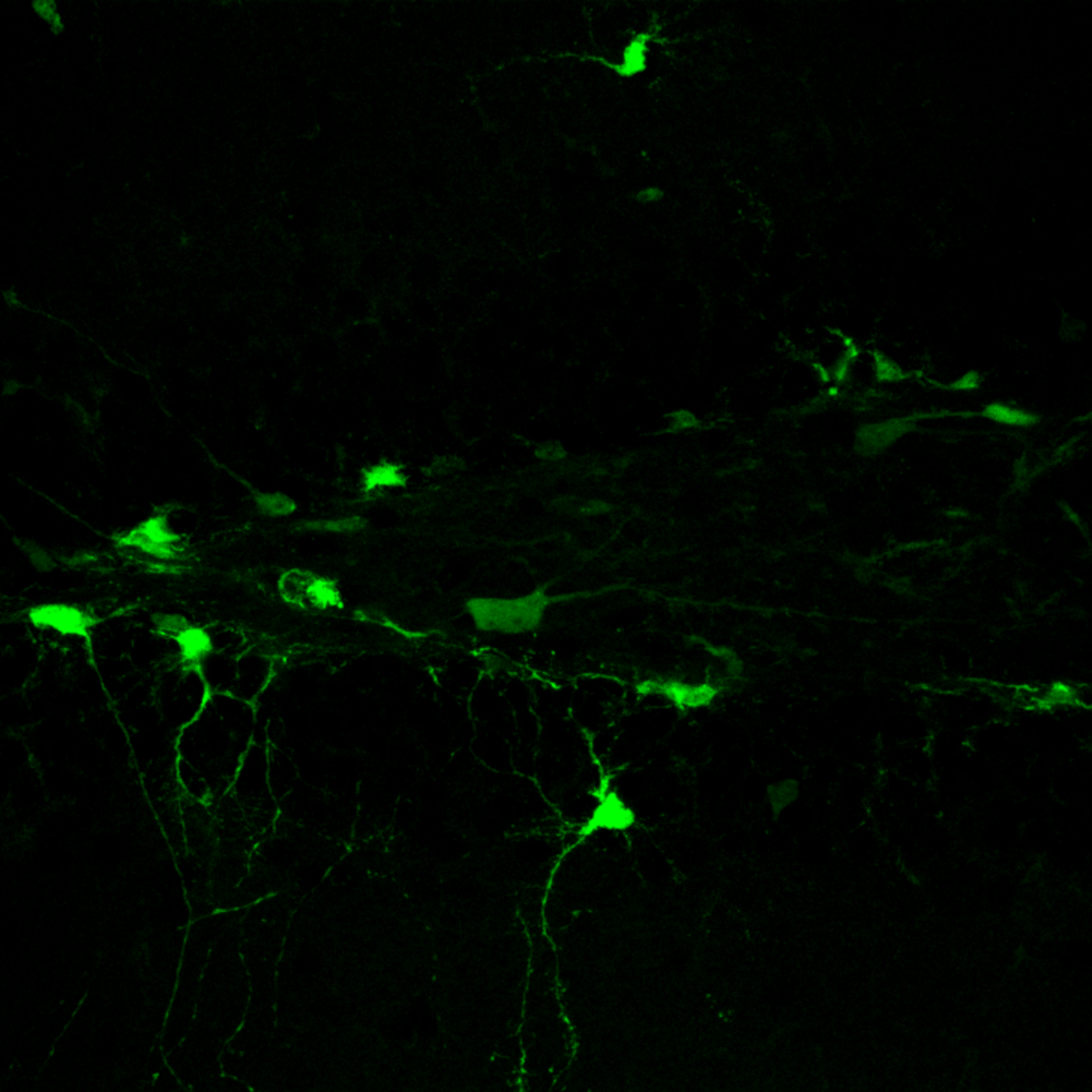

Supplement: Supplementary file 4 — Source Data Fig. 3 [file 44318_2023_11_MOESM4_ESM.zip › EMBOJ-2023-113564_SourceDataForFigure3/3A/P28/KO P28 Dentate Gyrus_Nestin.tiff]

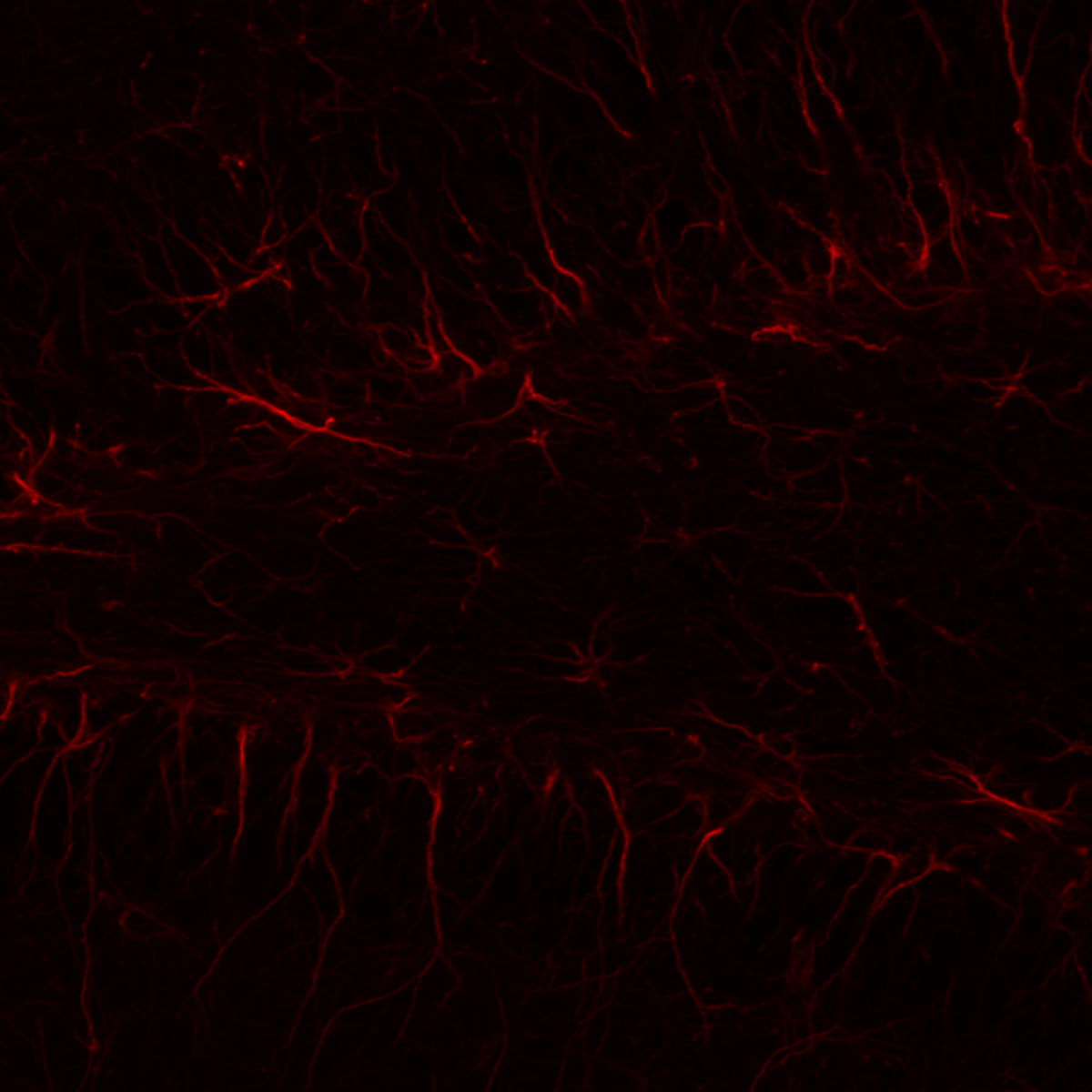

Supplement: Supplementary file 4 — Source Data Fig. 3 [file 44318_2023_11_MOESM4_ESM.zip › EMBOJ-2023-113564_SourceDataForFigure3/3A/P28/WT P28 Dentate Gyrus_GFAP.tiff]

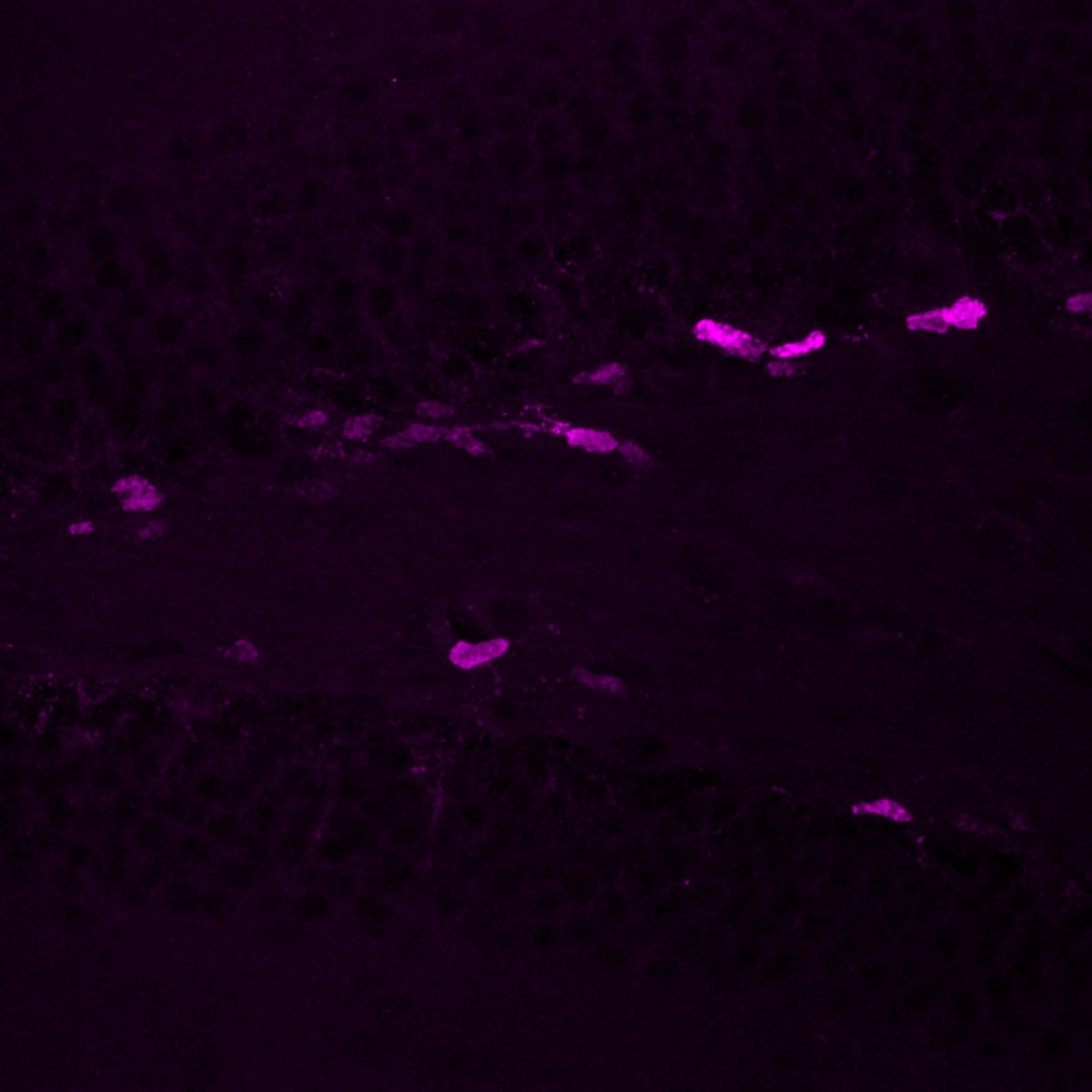

Supplement: Supplementary file 4 — Source Data Fig. 3 [file 44318_2023_11_MOESM4_ESM.zip › EMBOJ-2023-113564_SourceDataForFigure3/3A/P28/WT P28 Dentate Gyrus_Ki67.tiff]

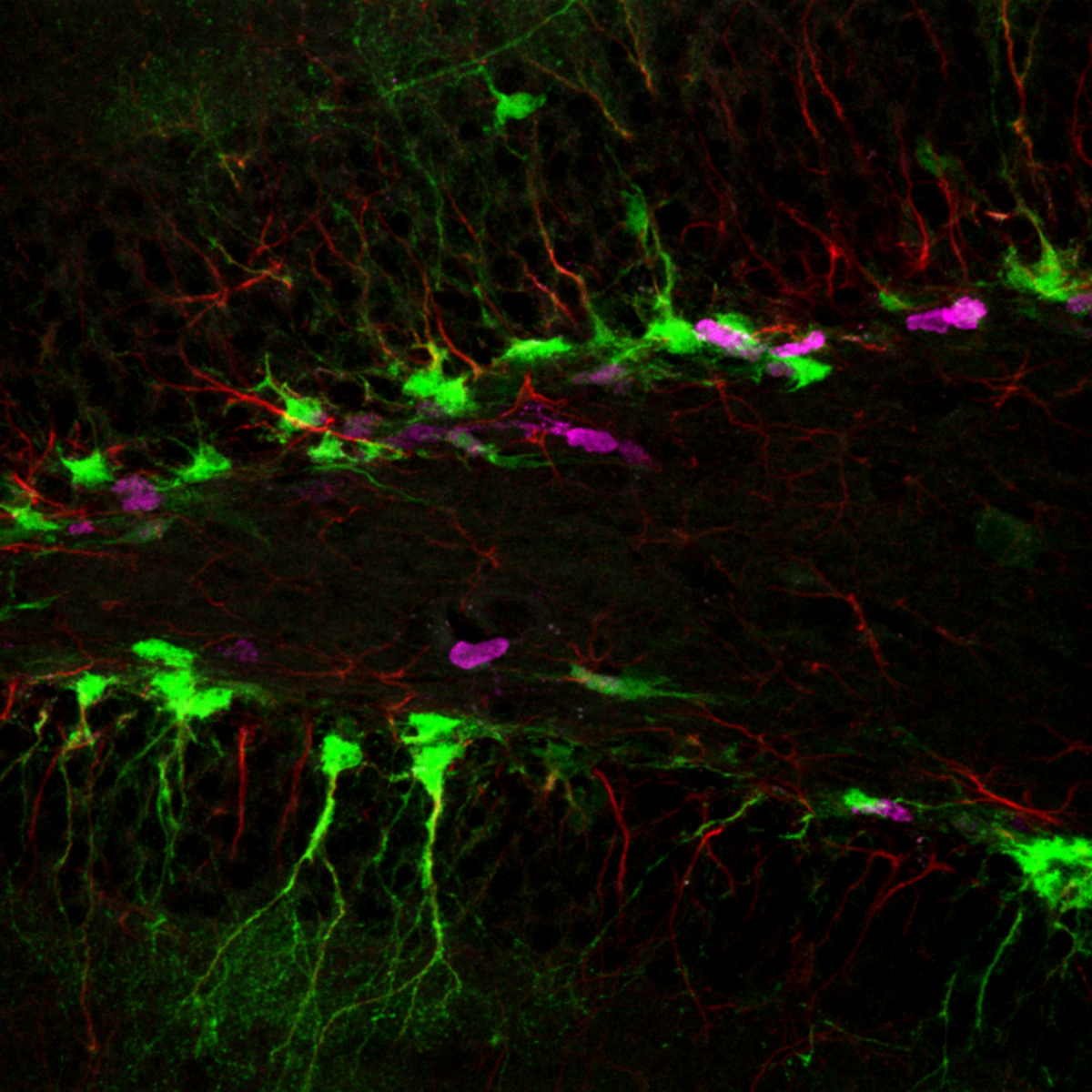

Supplement: Supplementary file 4 — Source Data Fig. 3 [file 44318_2023_11_MOESM4_ESM.zip › EMBOJ-2023-113564_SourceDataForFigure3/3A/P28/WT P28 Dentate Gyrus_Merge.tiff]

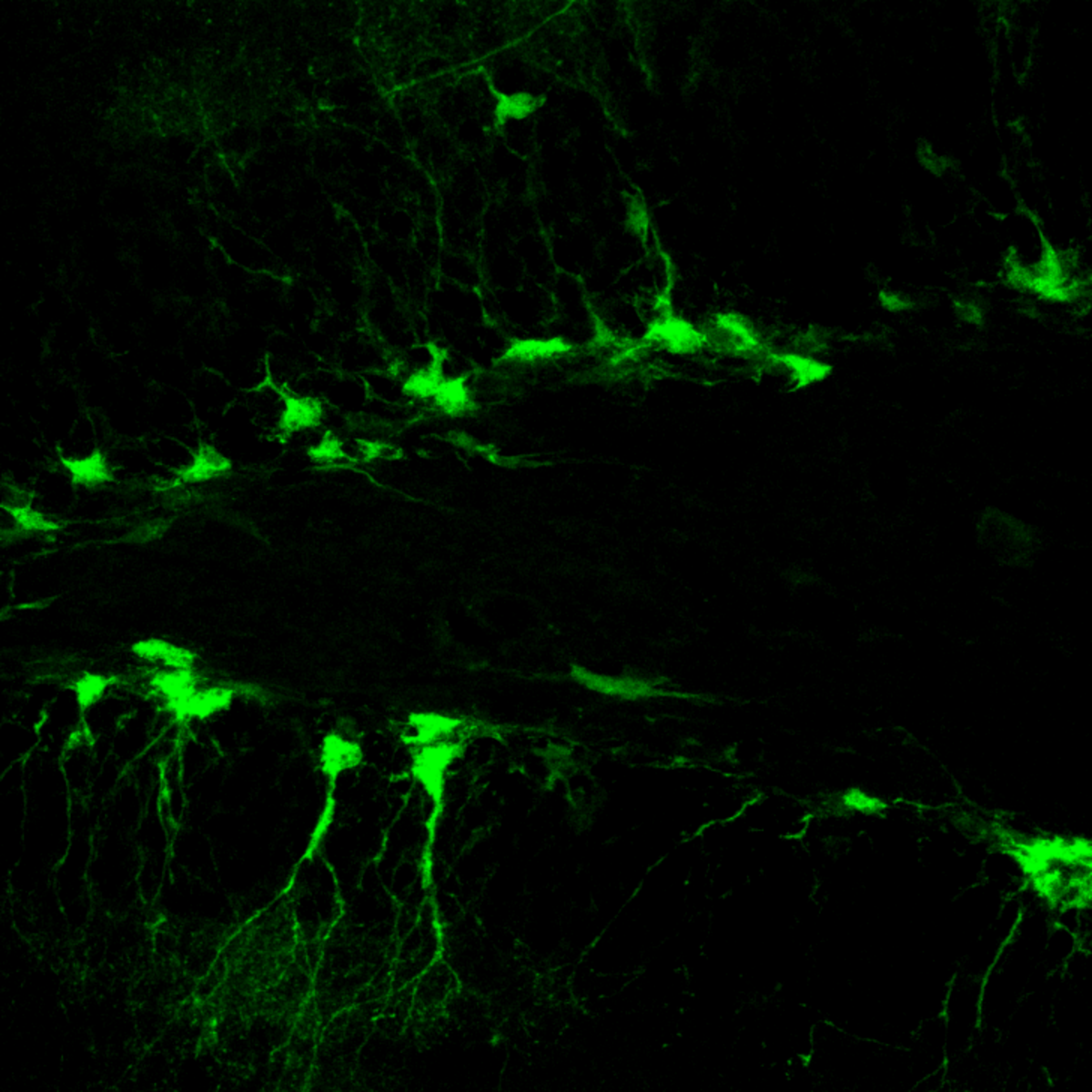

Supplement: Supplementary file 4 — Source Data Fig. 3 [file 44318_2023_11_MOESM4_ESM.zip › EMBOJ-2023-113564_SourceDataForFigure3/3A/P28/WT P28 Dentate Gyrus_Nestin.tiff]

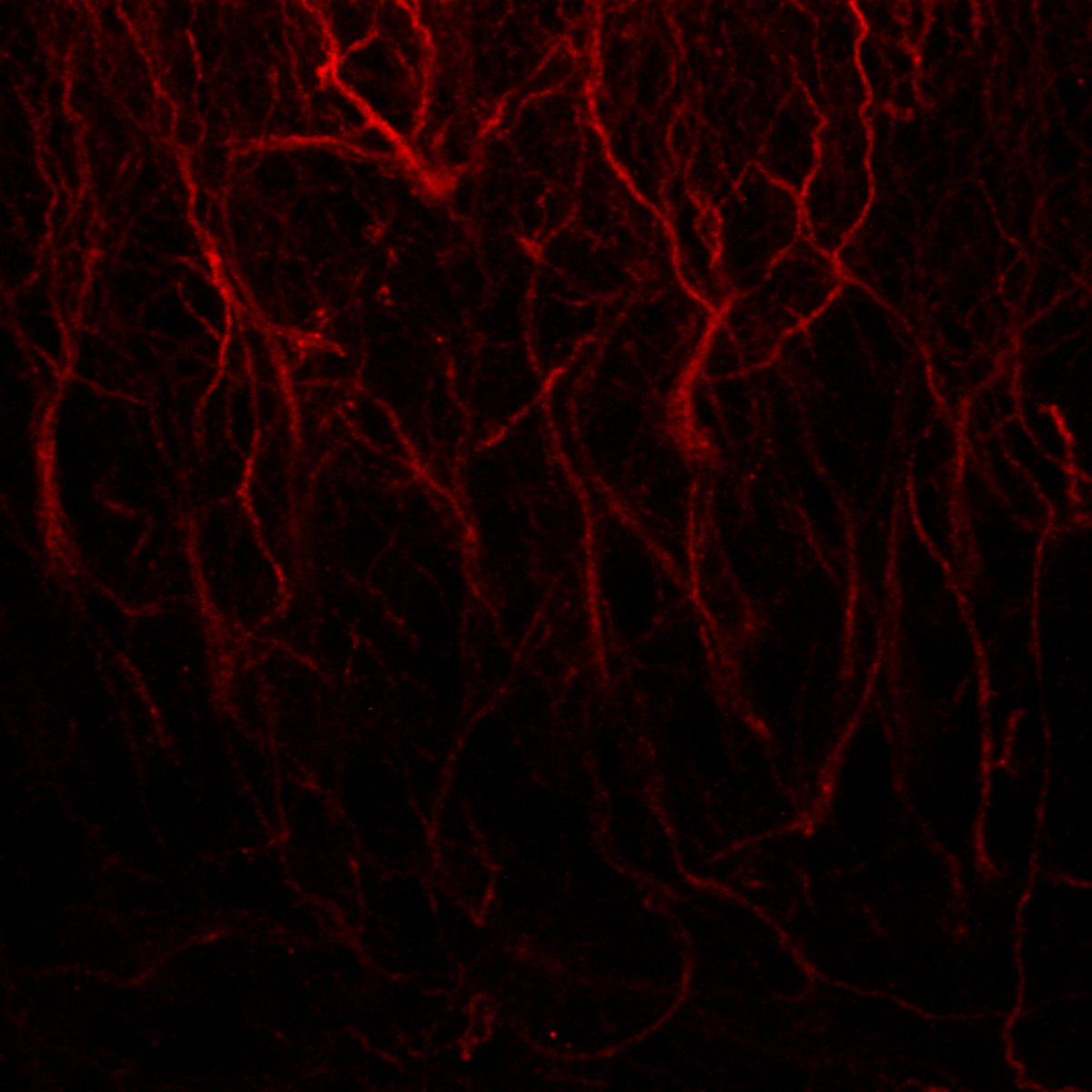

Supplement: Supplementary file 4 — Source Data Fig. 3 [file 44318_2023_11_MOESM4_ESM.zip › EMBOJ-2023-113564_SourceDataForFigure3/3A/P7/CloseUp Ko P7 GCL_GFAP.tiff]

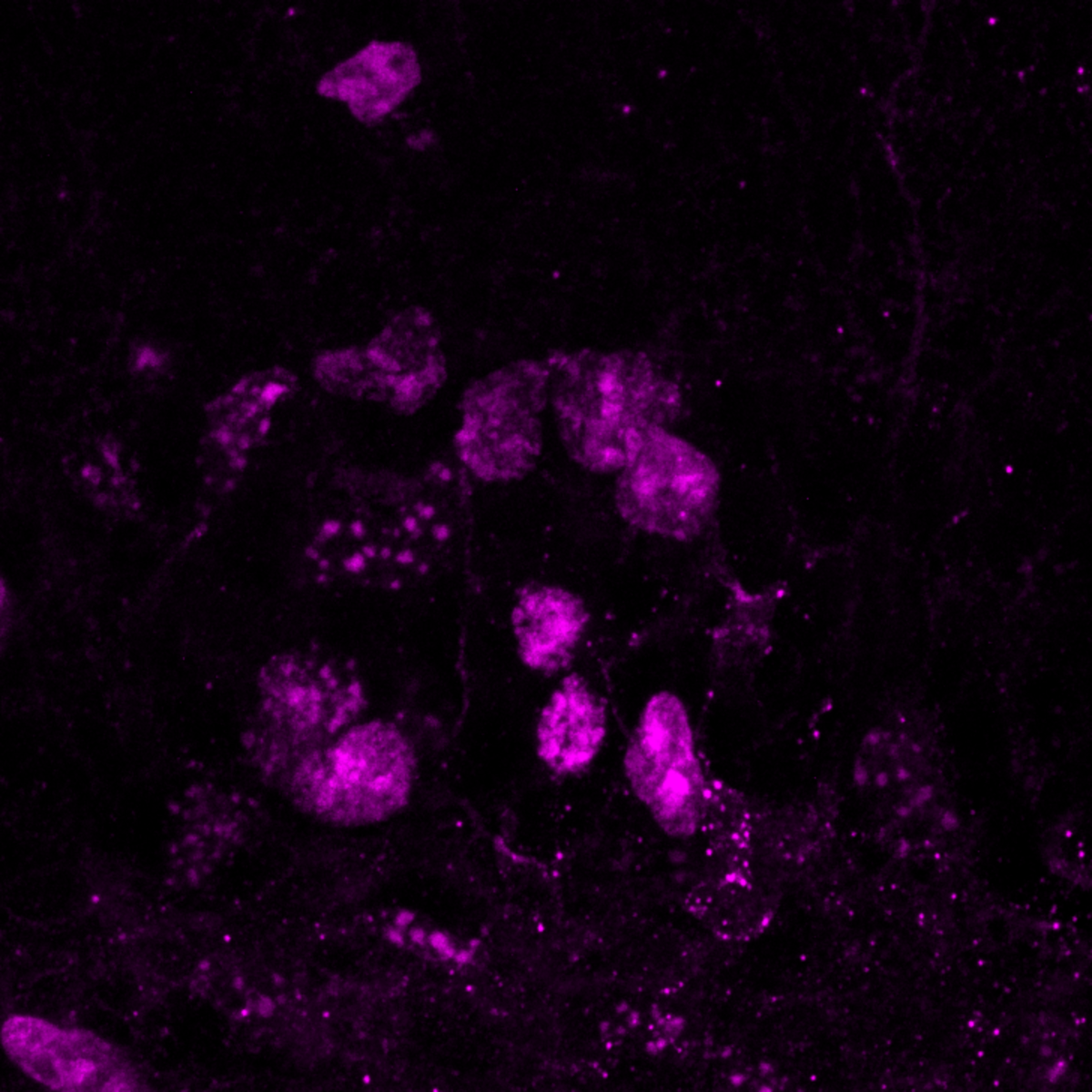

Supplement: Supplementary file 4 — Source Data Fig. 3 [file 44318_2023_11_MOESM4_ESM.zip › EMBOJ-2023-113564_SourceDataForFigure3/3A/P7/CloseUp Ko P7 GCL_Ki67.tiff]

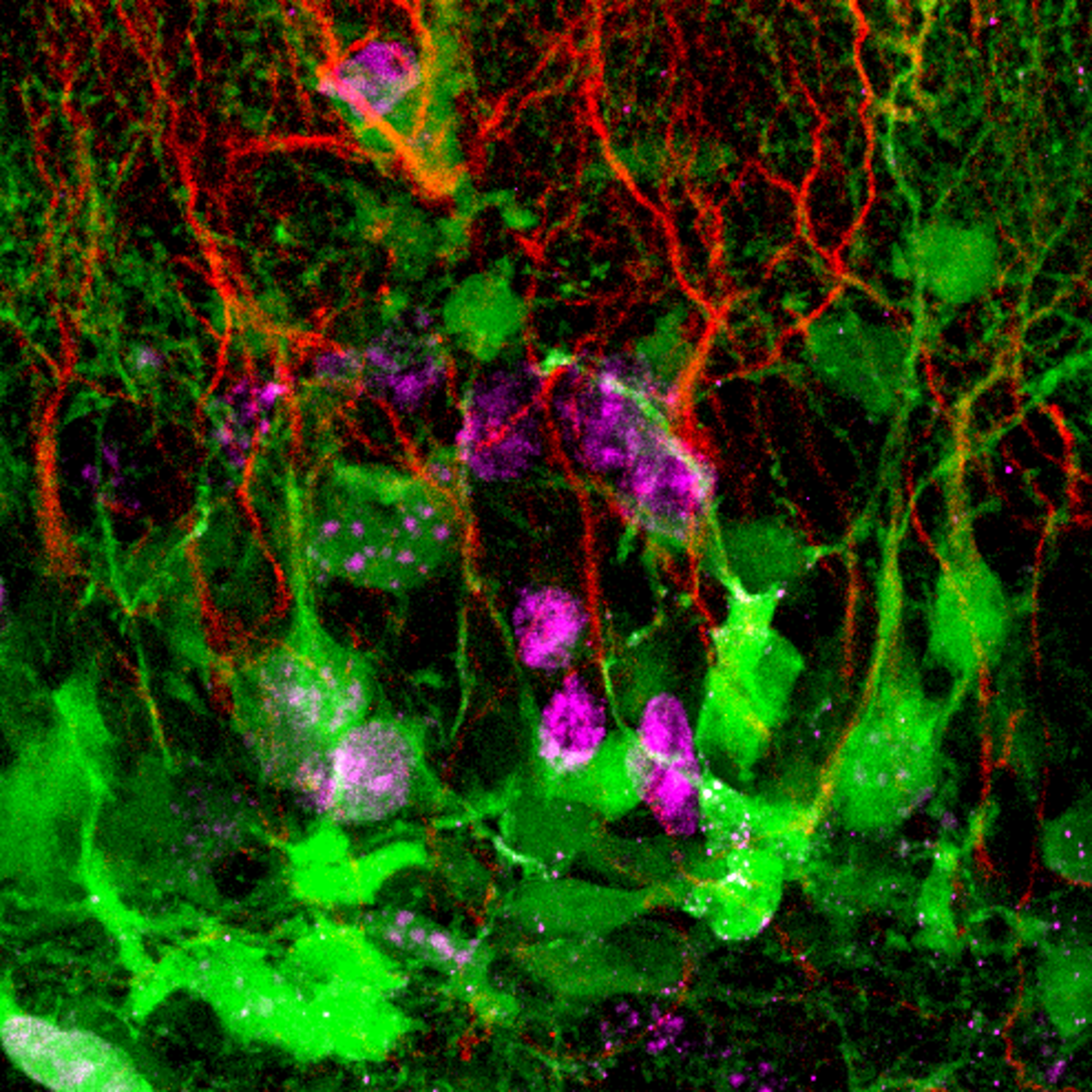

Supplement: Supplementary file 4 — Source Data Fig. 3 [file 44318_2023_11_MOESM4_ESM.zip › EMBOJ-2023-113564_SourceDataForFigure3/3A/P7/CloseUp Ko P7 GCL_Merge.tiff]

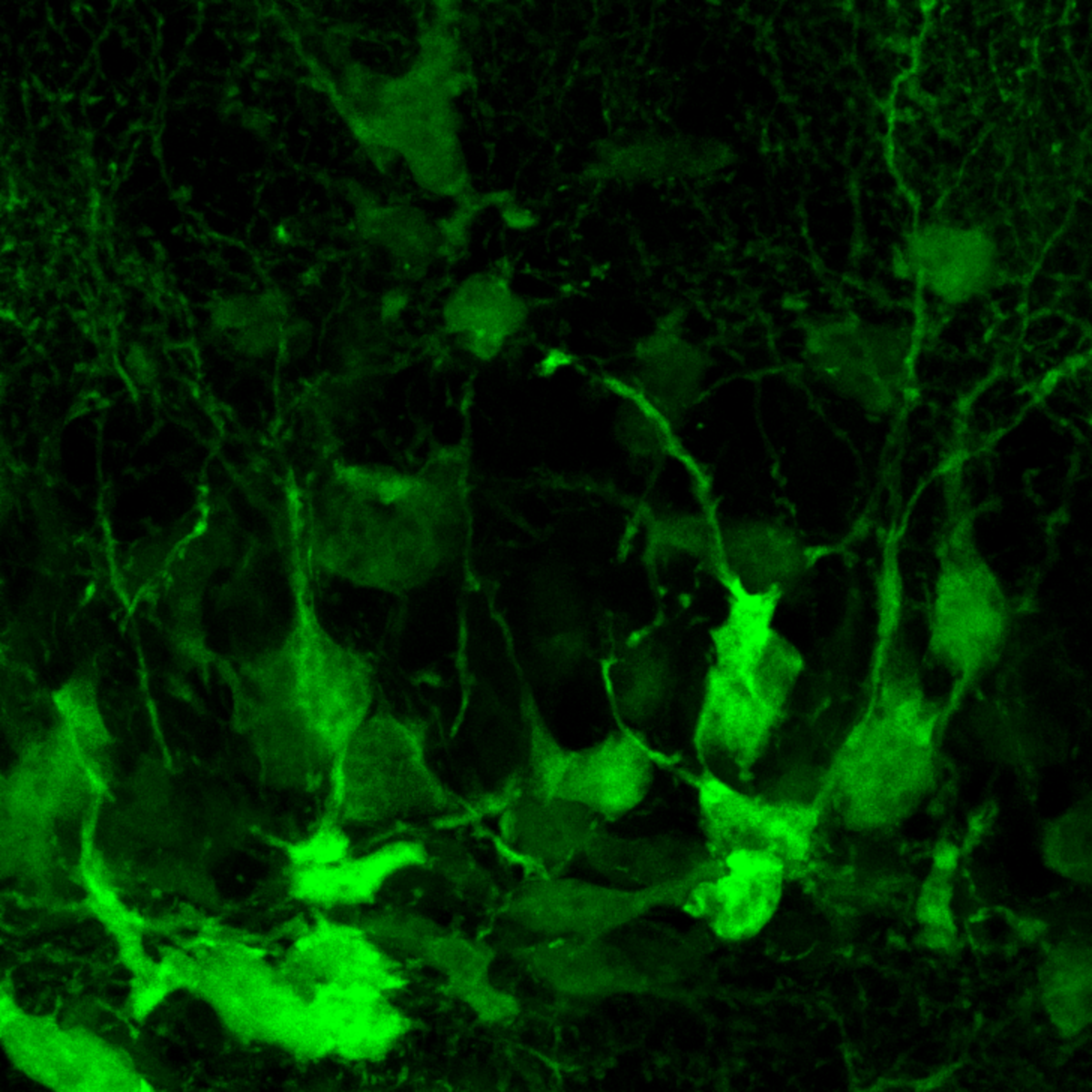

Supplement: Supplementary file 4 — Source Data Fig. 3 [file 44318_2023_11_MOESM4_ESM.zip › EMBOJ-2023-113564_SourceDataForFigure3/3A/P7/CloseUp Ko P7 GCL_Nestin.tiff]

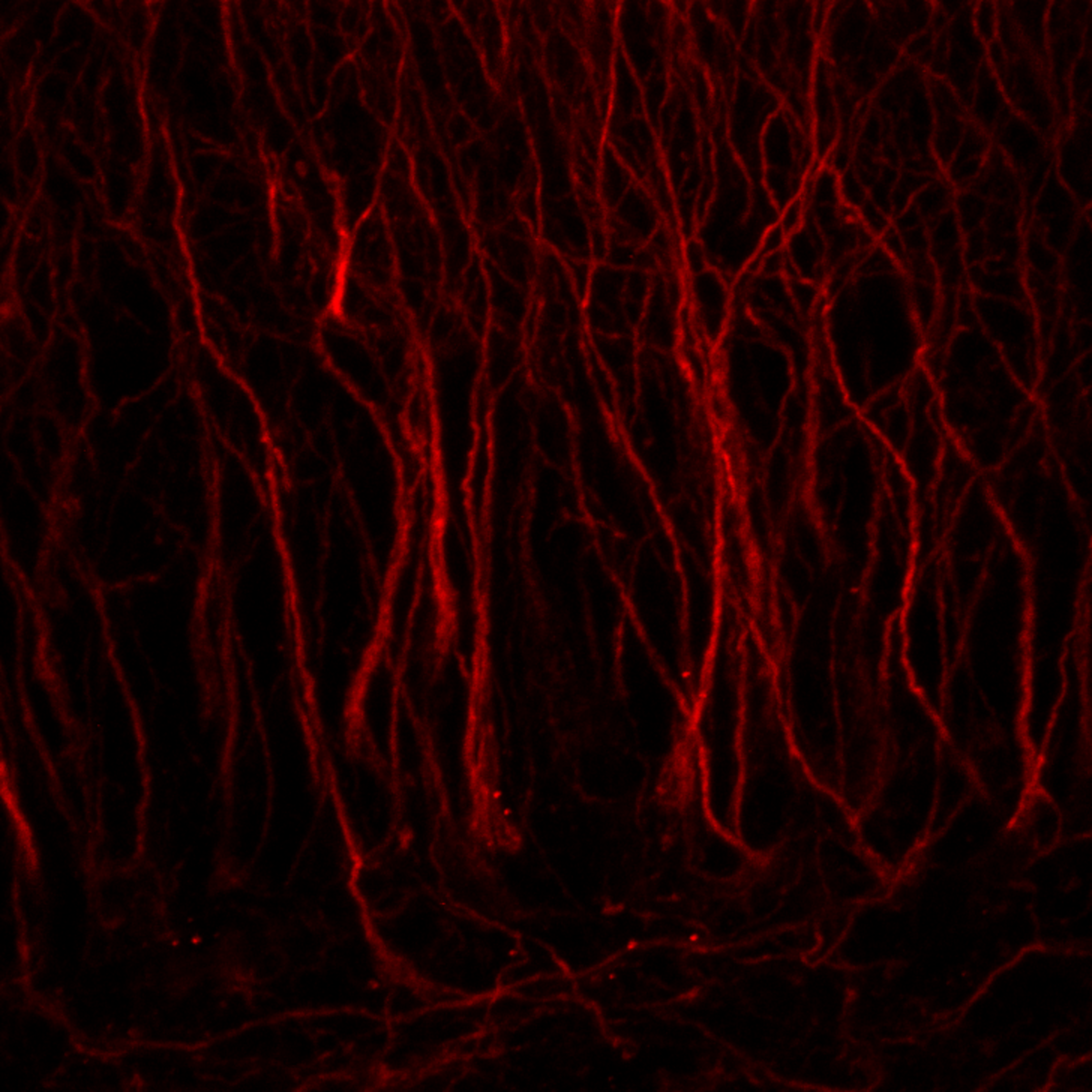

Supplement: Supplementary file 4 — Source Data Fig. 3 [file 44318_2023_11_MOESM4_ESM.zip › EMBOJ-2023-113564_SourceDataForFigure3/3A/P7/CloseUp WT P7 GCL_GFAP.tiff]

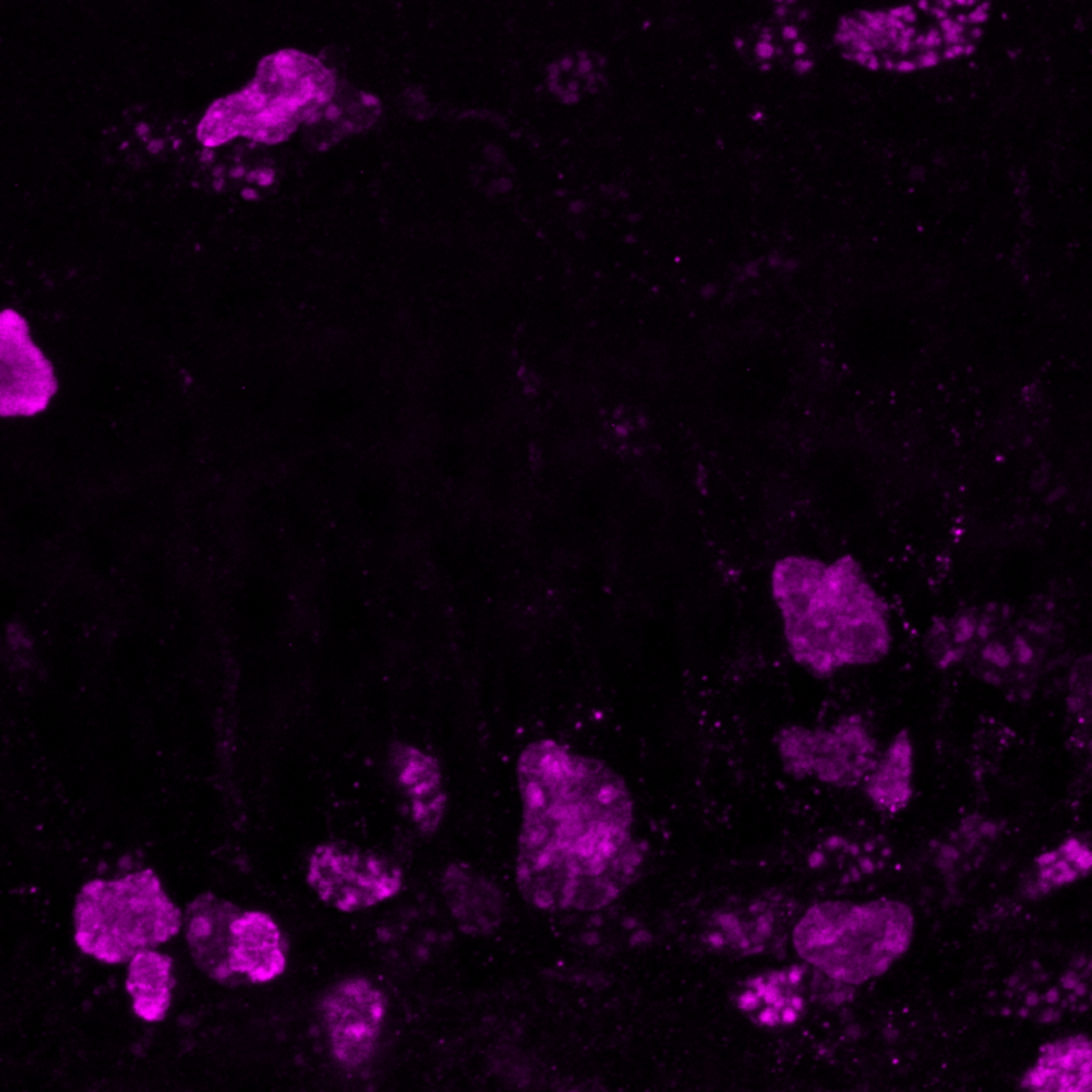

Supplement: Supplementary file 4 — Source Data Fig. 3 [file 44318_2023_11_MOESM4_ESM.zip › EMBOJ-2023-113564_SourceDataForFigure3/3A/P7/CloseUp WT P7 GCL_Ki67.tiff]

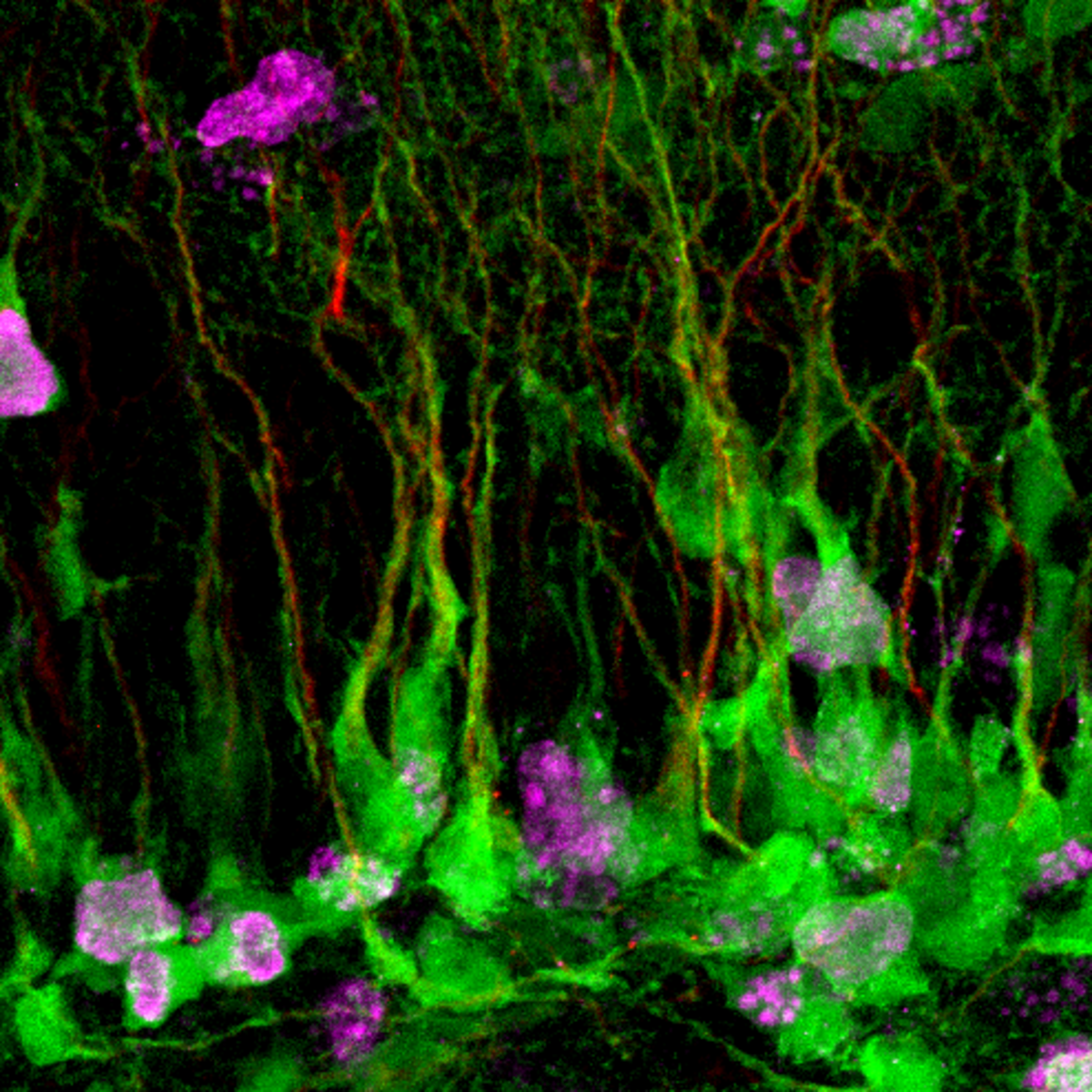

Supplement: Supplementary file 4 — Source Data Fig. 3 [file 44318_2023_11_MOESM4_ESM.zip › EMBOJ-2023-113564_SourceDataForFigure3/3A/P7/CloseUp WT P7 GCL_Merge.tiff]

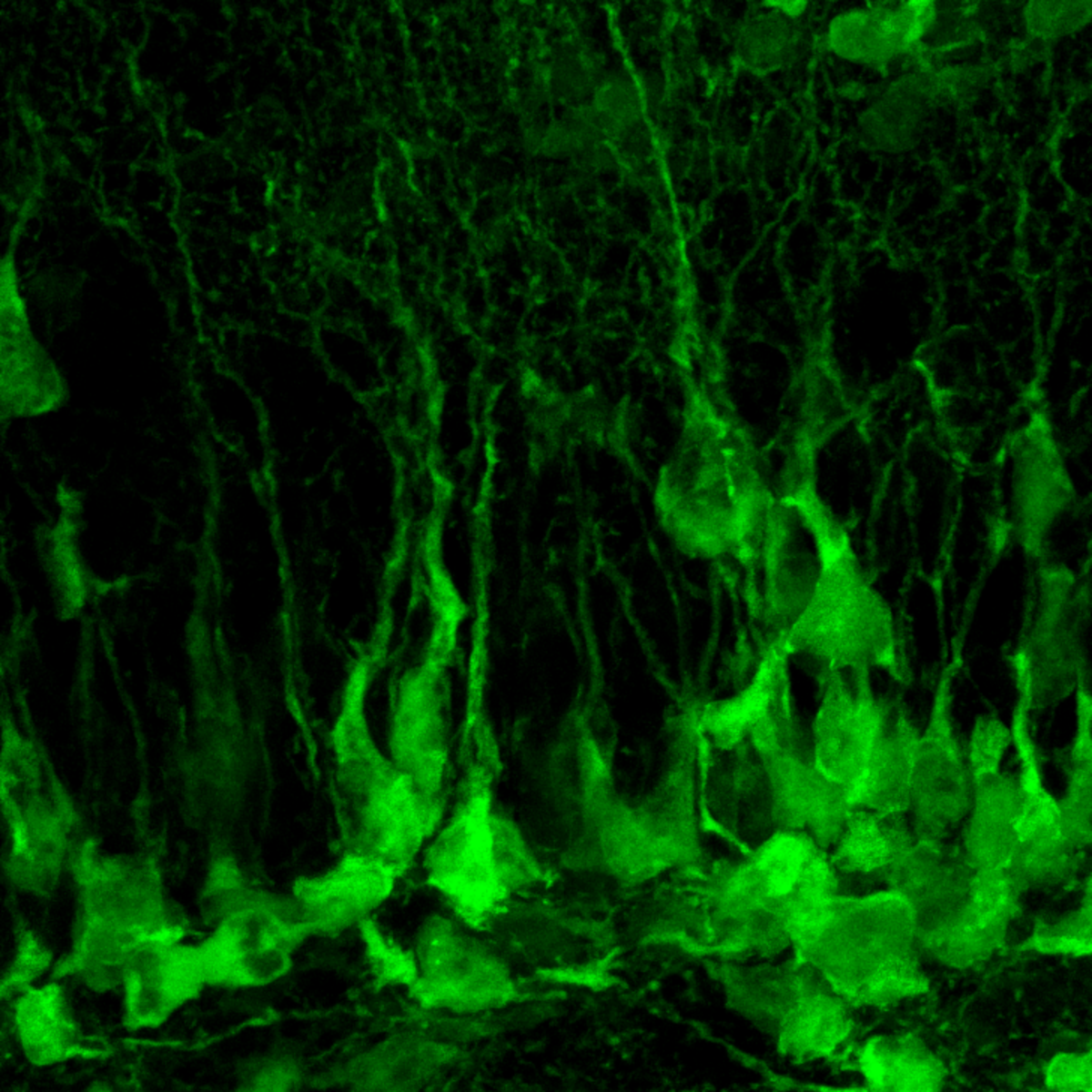

Supplement: Supplementary file 4 — Source Data Fig. 3 [file 44318_2023_11_MOESM4_ESM.zip › EMBOJ-2023-113564_SourceDataForFigure3/3A/P7/CloseUp WT P7 GCL_Nestin.tiff]

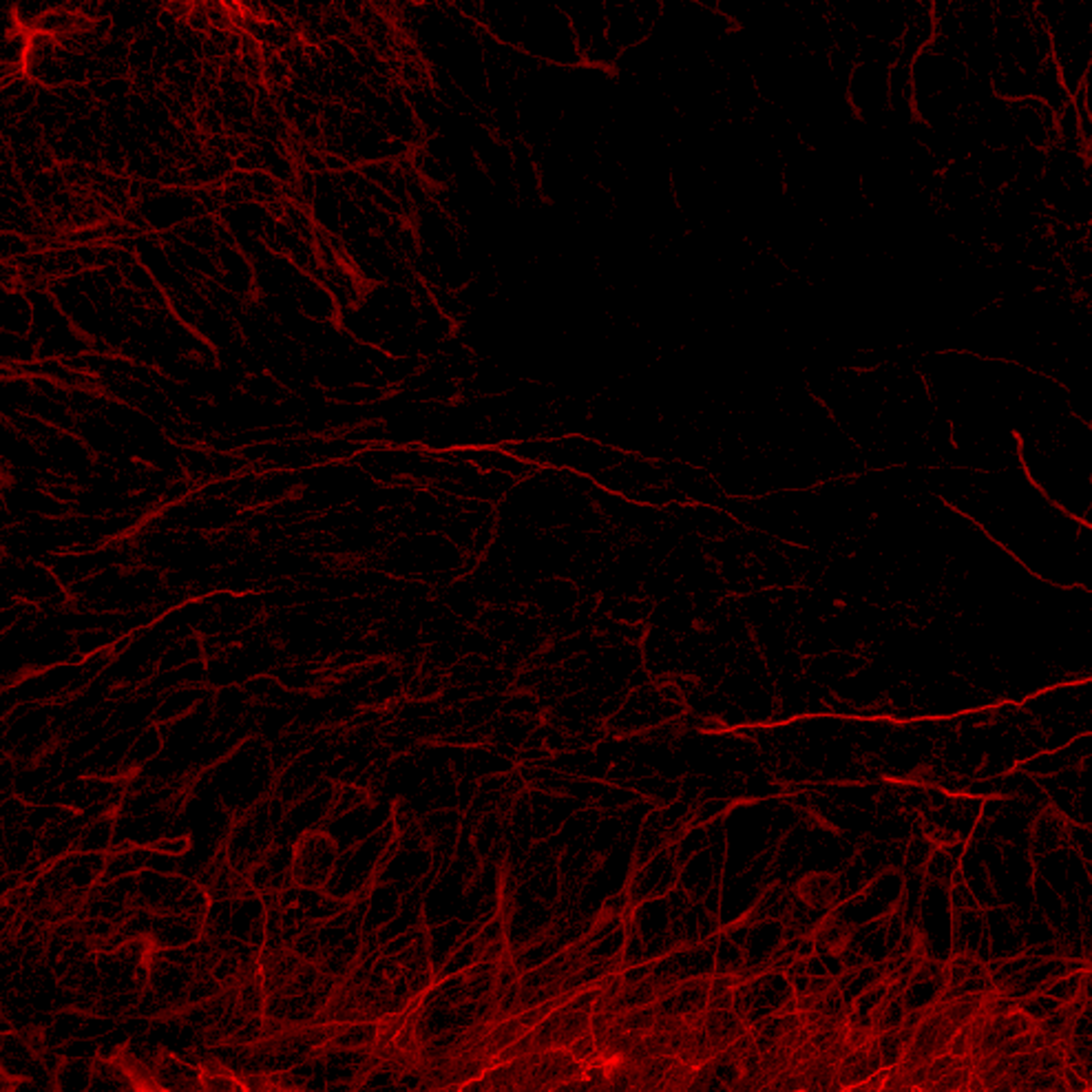

Supplement: Supplementary file 4 — Source Data Fig. 3 [file 44318_2023_11_MOESM4_ESM.zip › EMBOJ-2023-113564_SourceDataForFigure3/3A/P7/KO P7 Dentate Gyrus_GFAP.tiff]

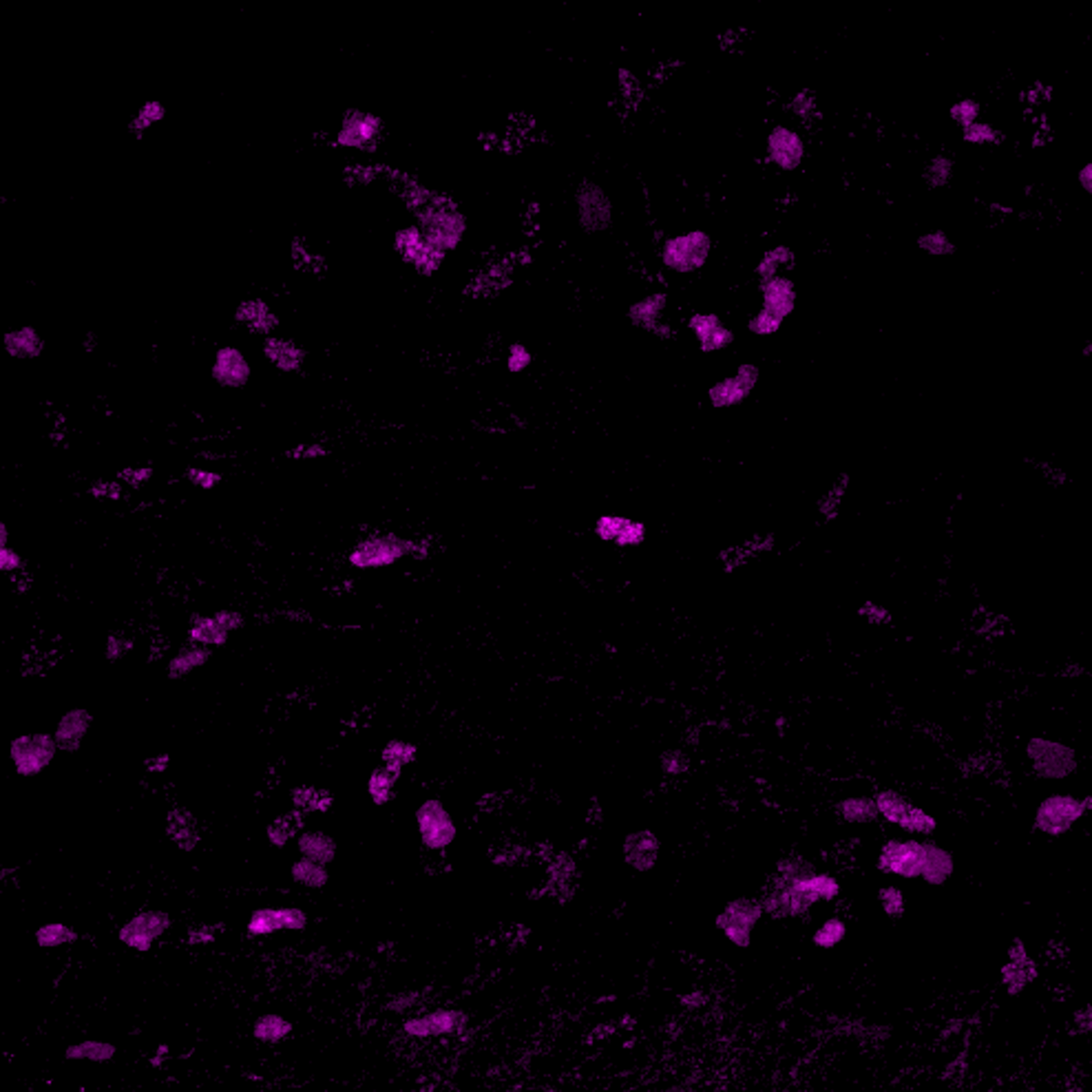

Supplement: Supplementary file 4 — Source Data Fig. 3 [file 44318_2023_11_MOESM4_ESM.zip › EMBOJ-2023-113564_SourceDataForFigure3/3A/P7/KO P7 Dentate Gyrus_Ki67.tiff]

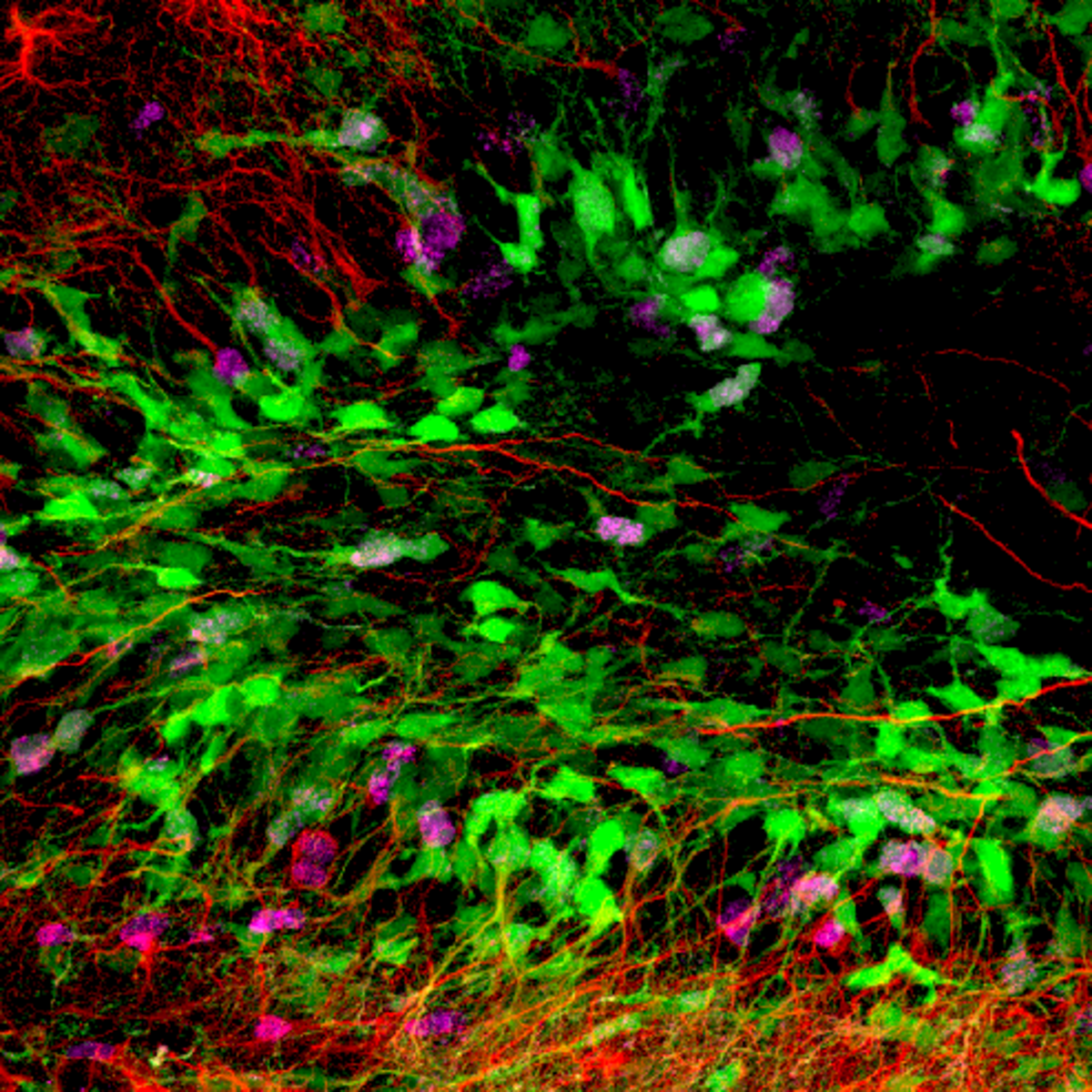

Supplement: Supplementary file 4 — Source Data Fig. 3 [file 44318_2023_11_MOESM4_ESM.zip › EMBOJ-2023-113564_SourceDataForFigure3/3A/P7/KO P7 Dentate Gyrus_Merge.tiff]

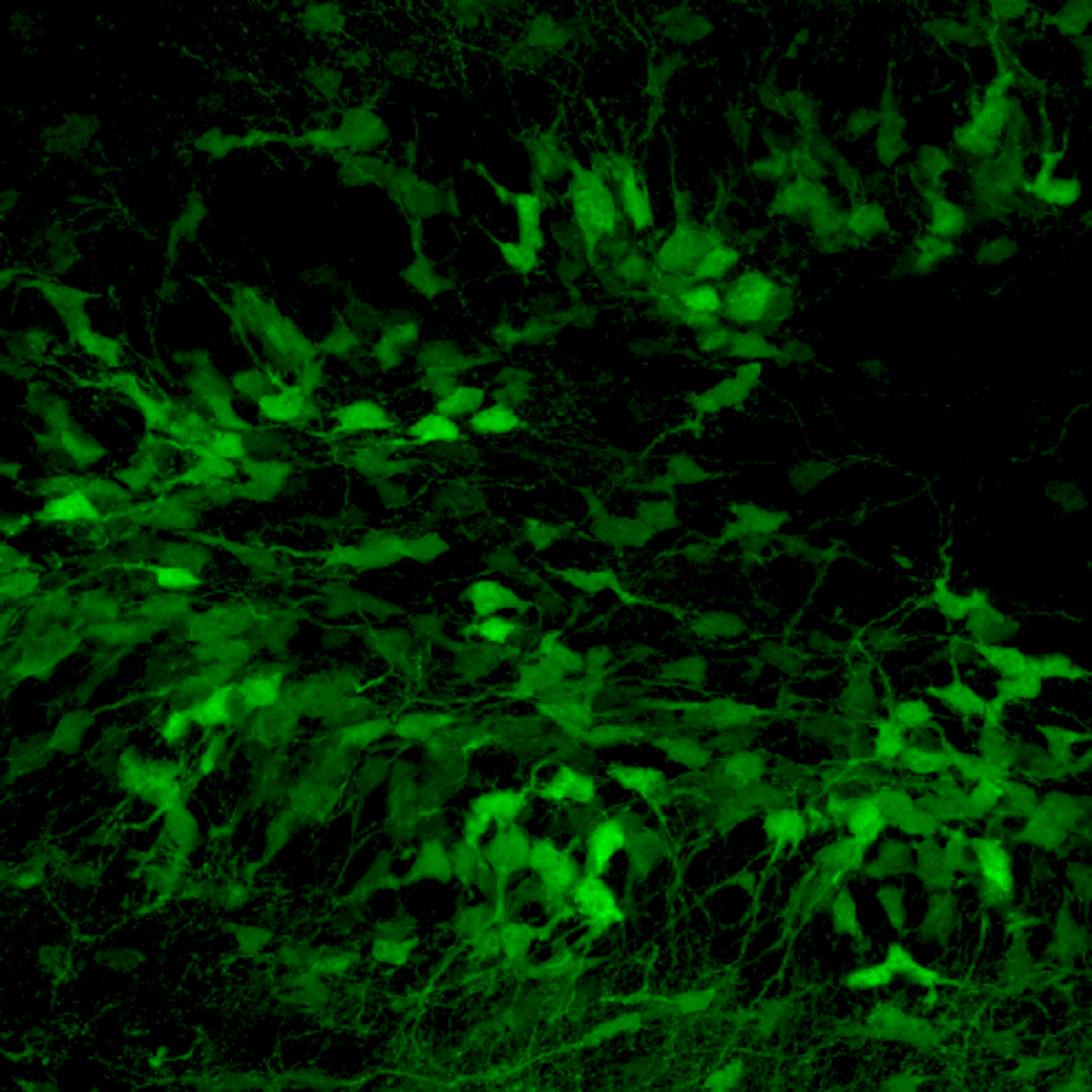

Supplement: Supplementary file 4 — Source Data Fig. 3 [file 44318_2023_11_MOESM4_ESM.zip › EMBOJ-2023-113564_SourceDataForFigure3/3A/P7/KO P7 Dentate Gyrus_Nestin.tiff]

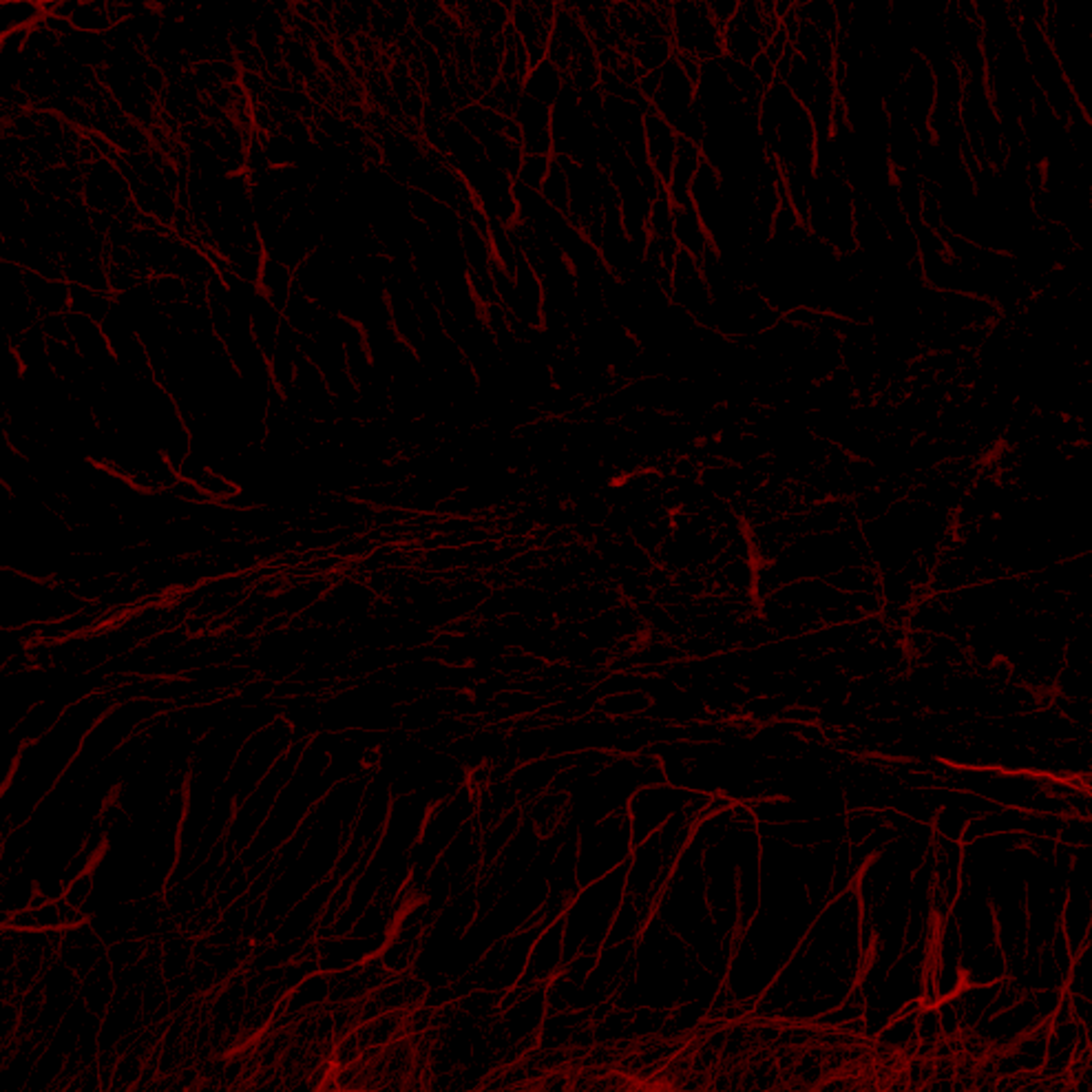

Supplement: Supplementary file 4 — Source Data Fig. 3 [file 44318_2023_11_MOESM4_ESM.zip › EMBOJ-2023-113564_SourceDataForFigure3/3A/P7/WT P7 Dentate Gyrus_GFAP.tiff]

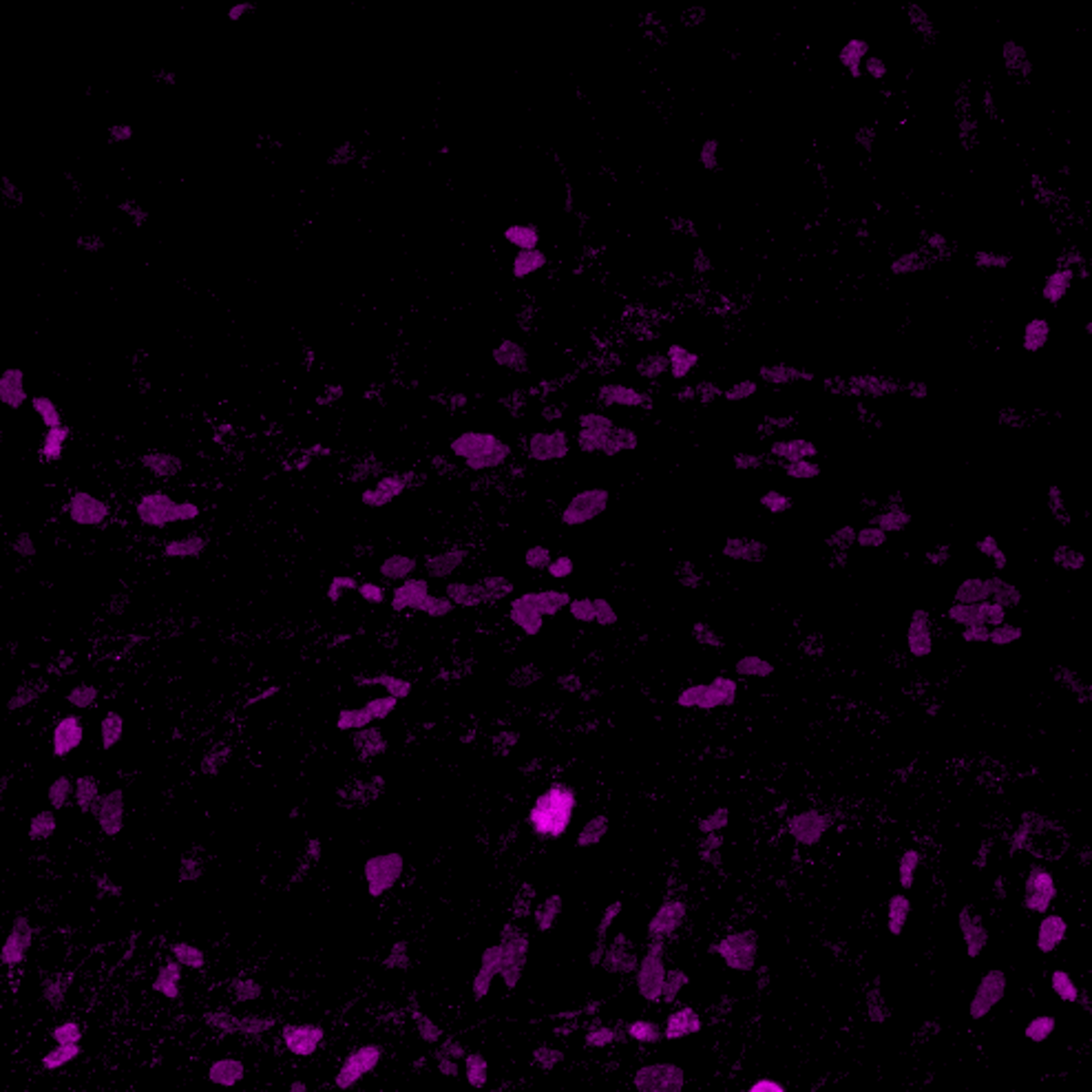

Supplement: Supplementary file 4 — Source Data Fig. 3 [file 44318_2023_11_MOESM4_ESM.zip › EMBOJ-2023-113564_SourceDataForFigure3/3A/P7/WT P7 Dentate Gyrus_Ki67.tiff]

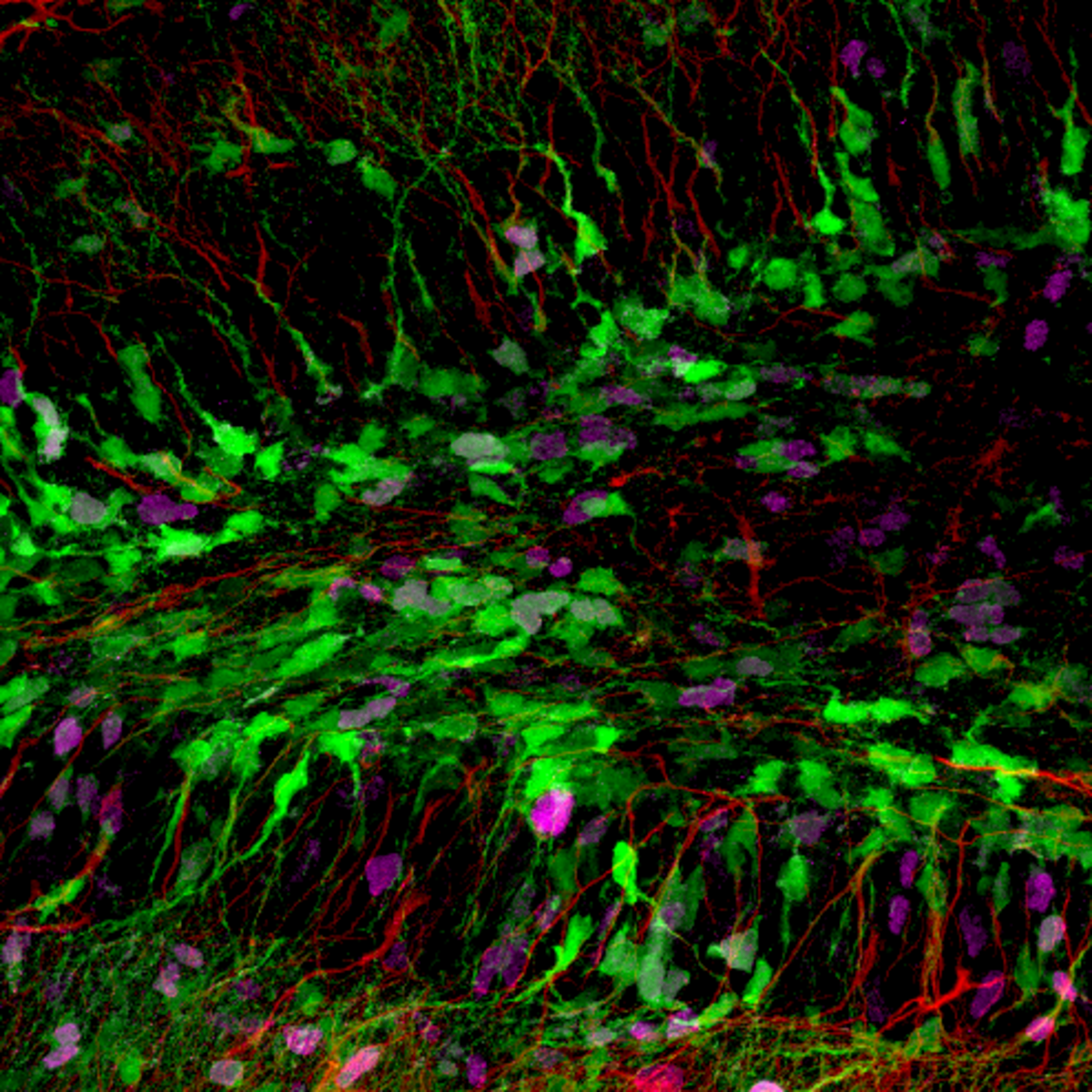

Supplement: Supplementary file 4 — Source Data Fig. 3 [file 44318_2023_11_MOESM4_ESM.zip › EMBOJ-2023-113564_SourceDataForFigure3/3A/P7/WT P7 Dentate Gyrus_Merge.tiff]

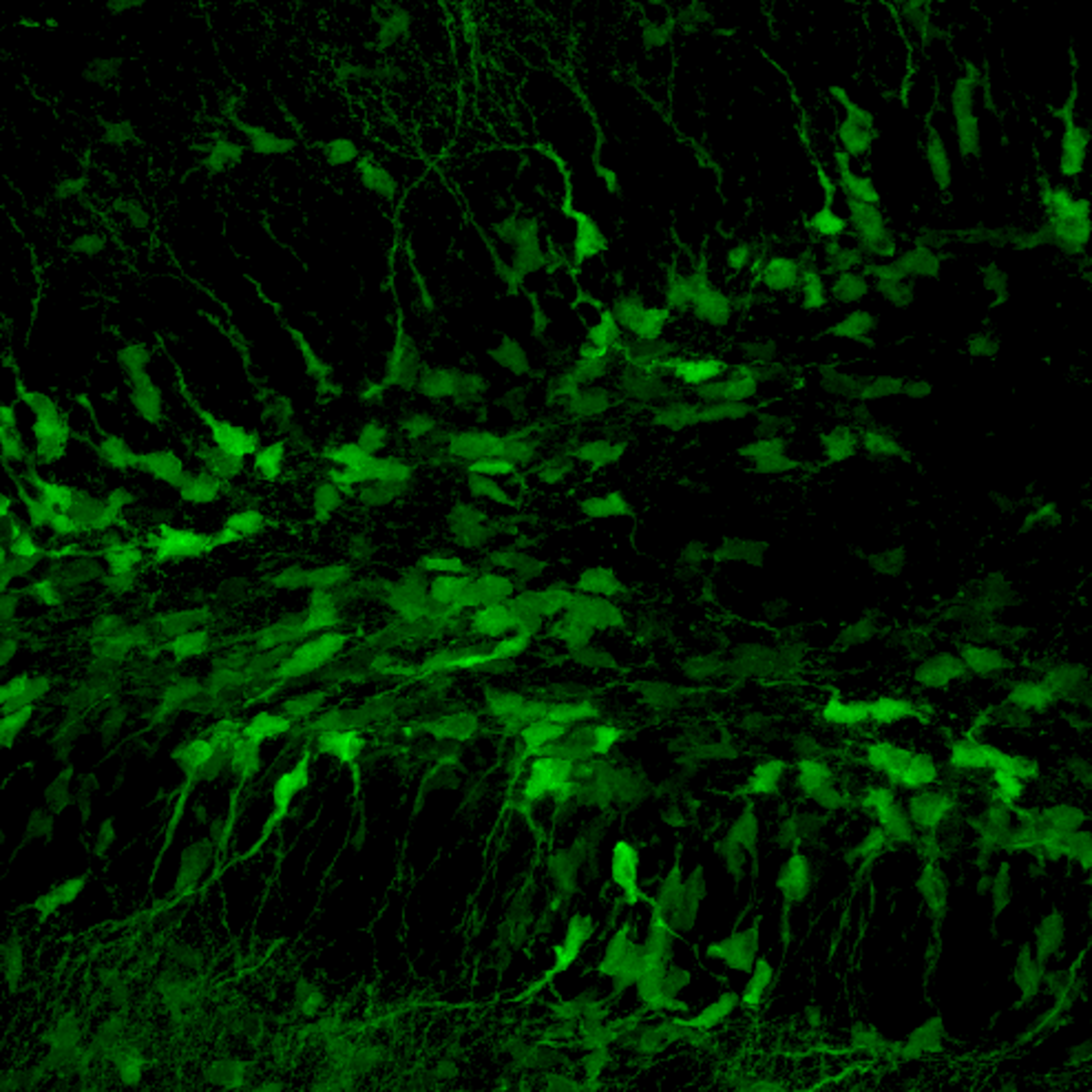

Supplement: Supplementary file 4 — Source Data Fig. 3 [file 44318_2023_11_MOESM4_ESM.zip › EMBOJ-2023-113564_SourceDataForFigure3/3A/P7/WT P7 Dentate Gyrus_Nestin.tiff]

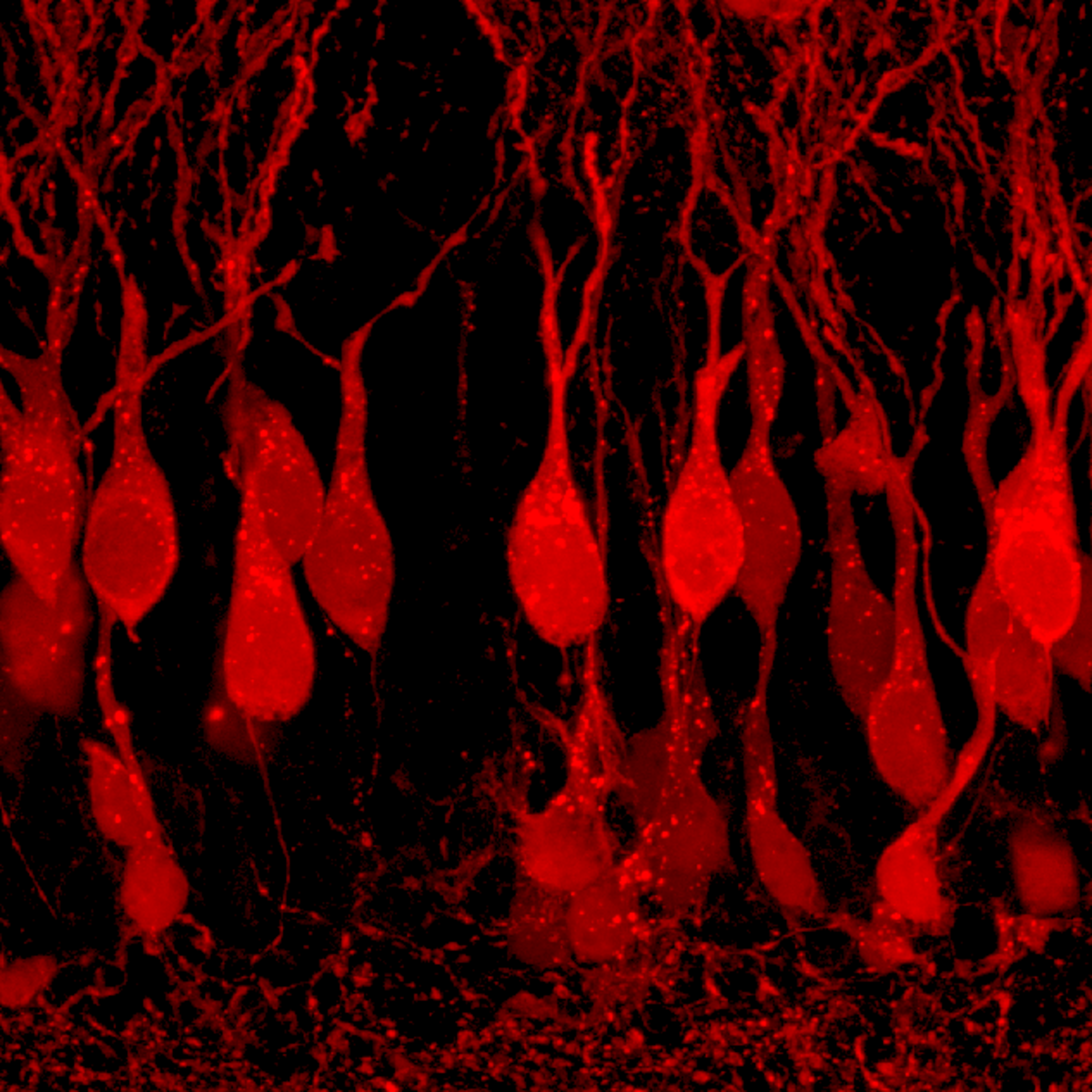

Supplement: Supplementary file 5 — Source Data Fig. 4 [file 44318_2023_11_MOESM5_ESM.zip › EMBOJ-2023-113564_SourceDataForFigure4/4A/P2_P14_DGinjection/p2_p14 DG inj_Cherry.tiff]

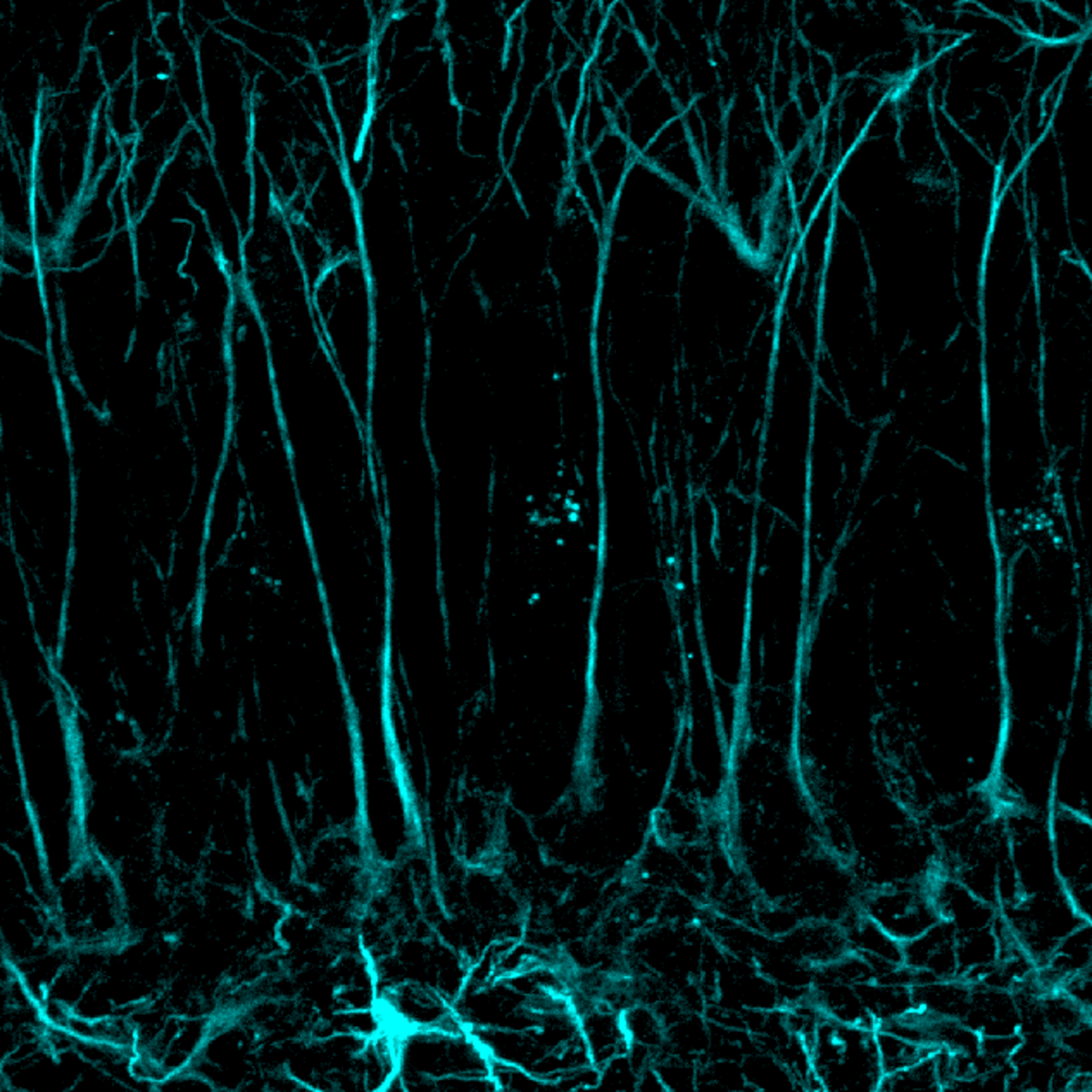

Supplement: Supplementary file 5 — Source Data Fig. 4 [file 44318_2023_11_MOESM5_ESM.zip › EMBOJ-2023-113564_SourceDataForFigure4/4A/P2_P14_DGinjection/p2_p14 DG inj_GFAP.tiff]

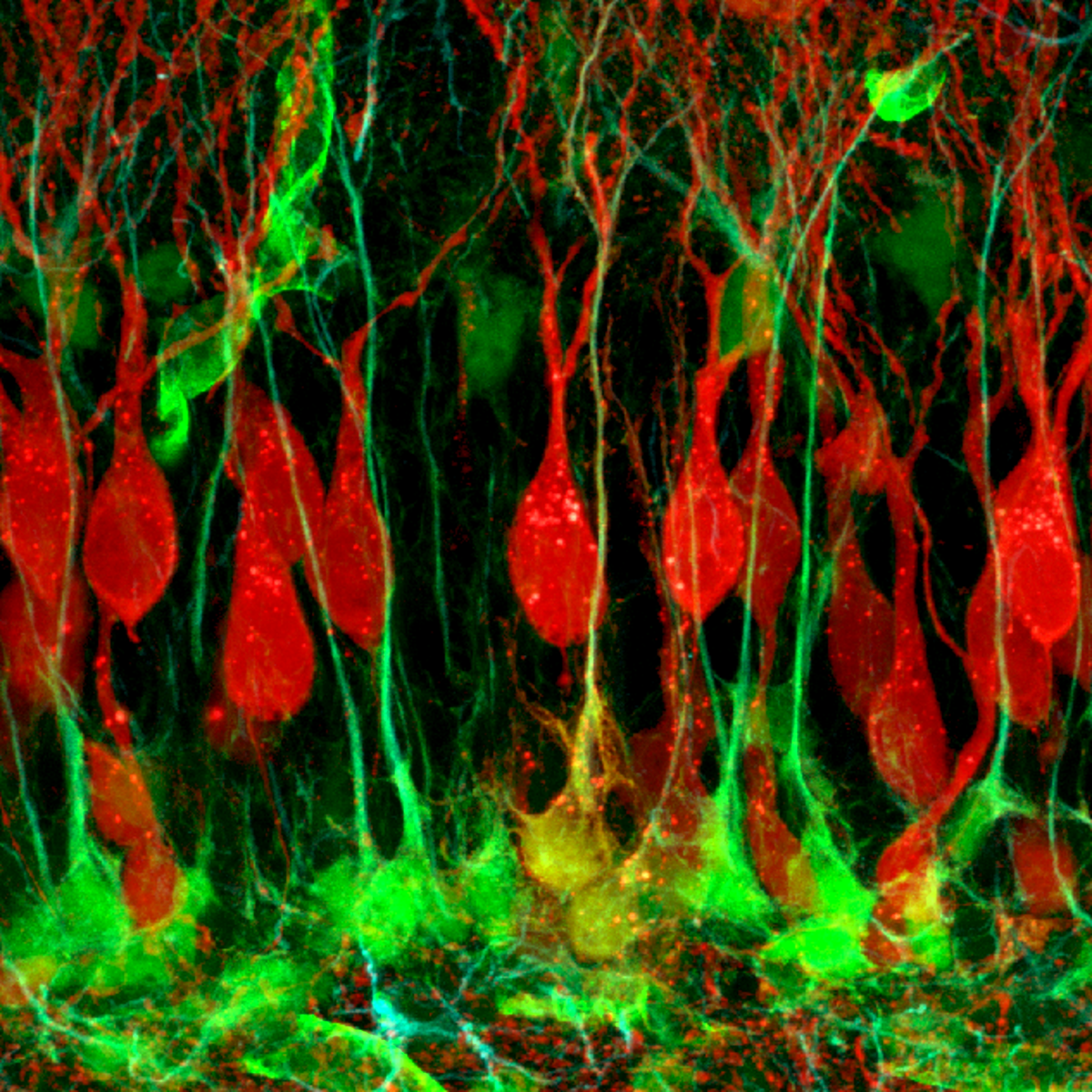

Supplement: Supplementary file 5 — Source Data Fig. 4 [file 44318_2023_11_MOESM5_ESM.zip › EMBOJ-2023-113564_SourceDataForFigure4/4A/P2_P14_DGinjection/p2_p14 DG inj_Merge.tif]

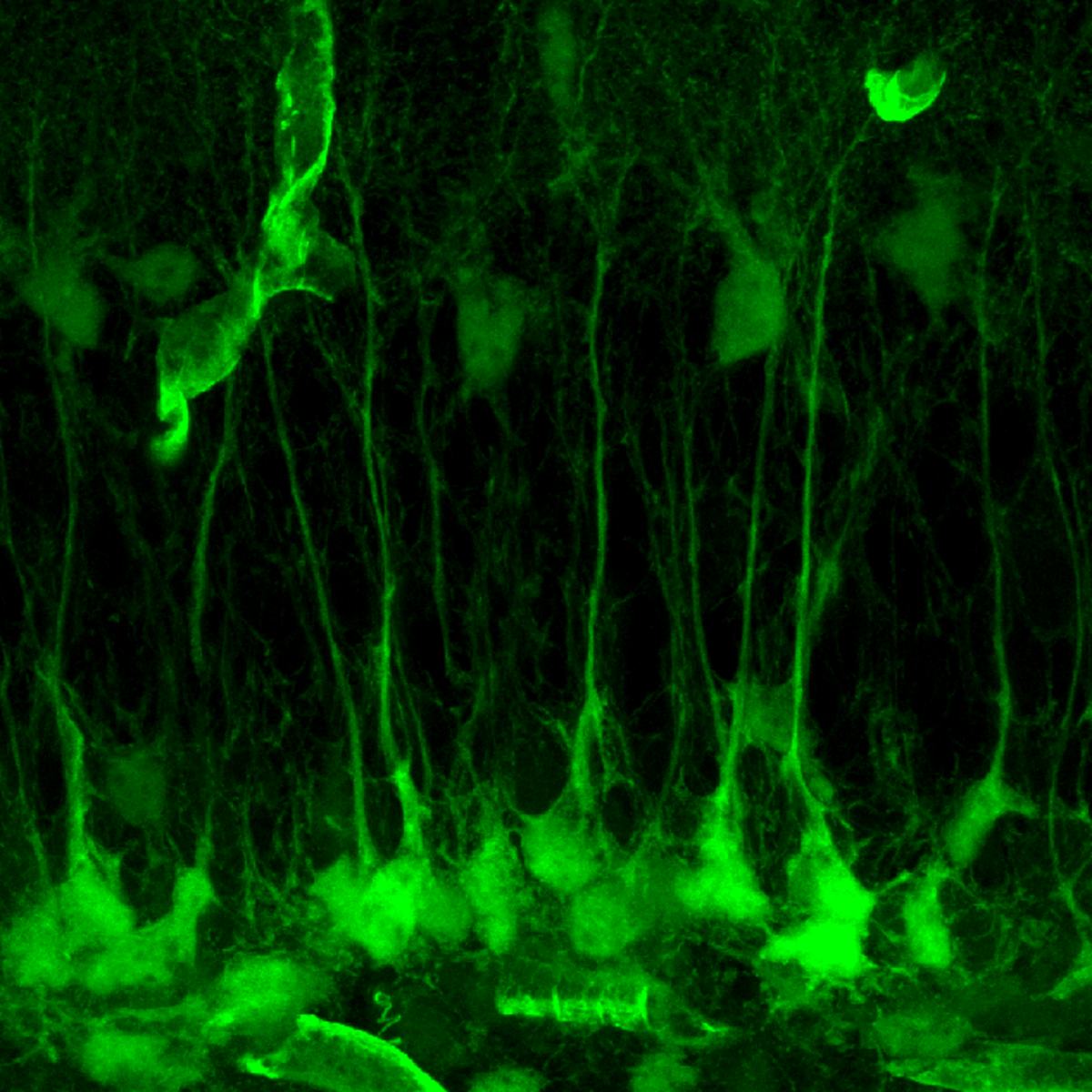

Supplement: Supplementary file 5 — Source Data Fig. 4 [file 44318_2023_11_MOESM5_ESM.zip › EMBOJ-2023-113564_SourceDataForFigure4/4A/P2_P14_DGinjection/p2_p14 DG inj_Nestin.tiff]

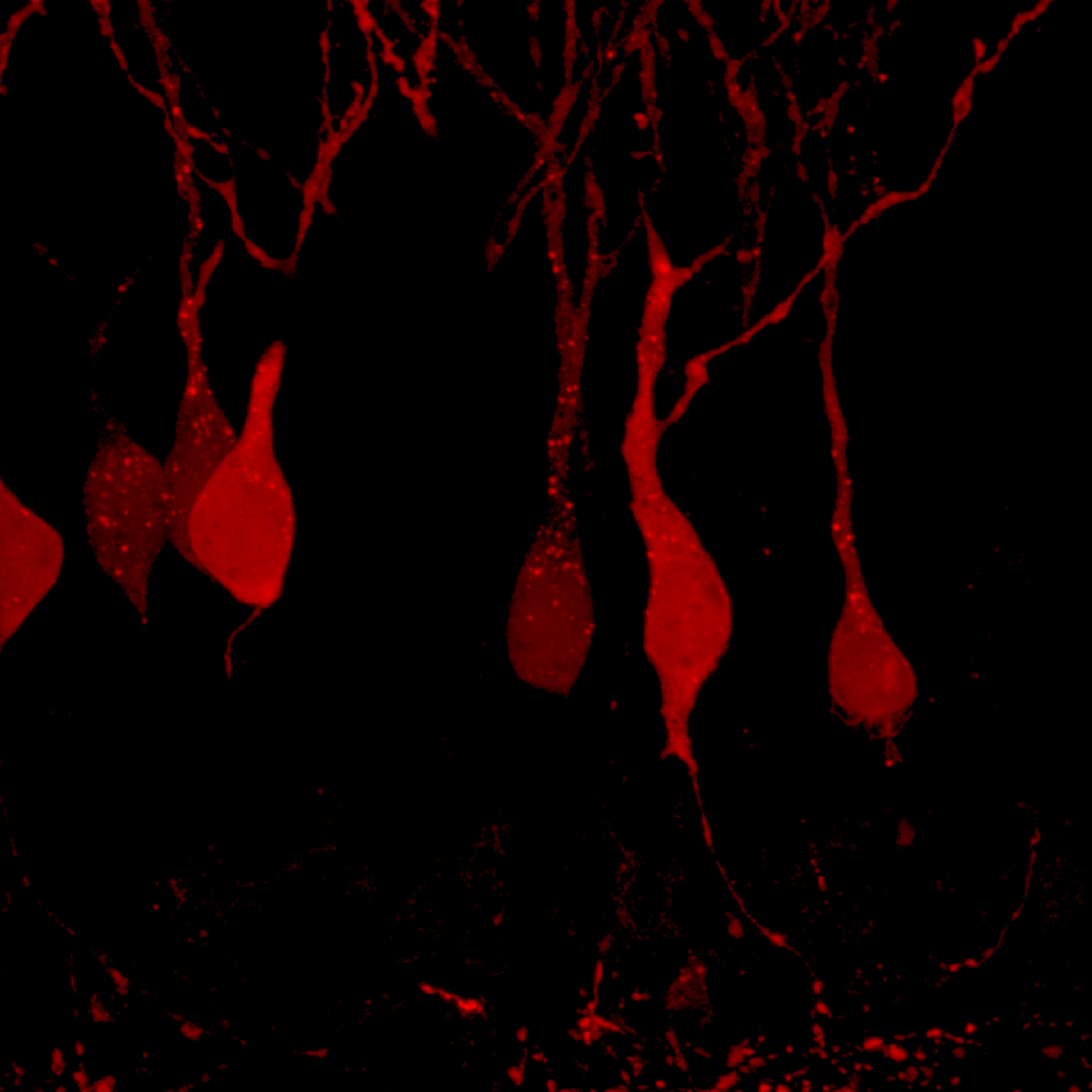

Supplement: Supplementary file 5 — Source Data Fig. 4 [file 44318_2023_11_MOESM5_ESM.zip › EMBOJ-2023-113564_SourceDataForFigure4/4A/P2_P14_DMSinjection/p2_p14 DMS inj_Cherry.tiff]

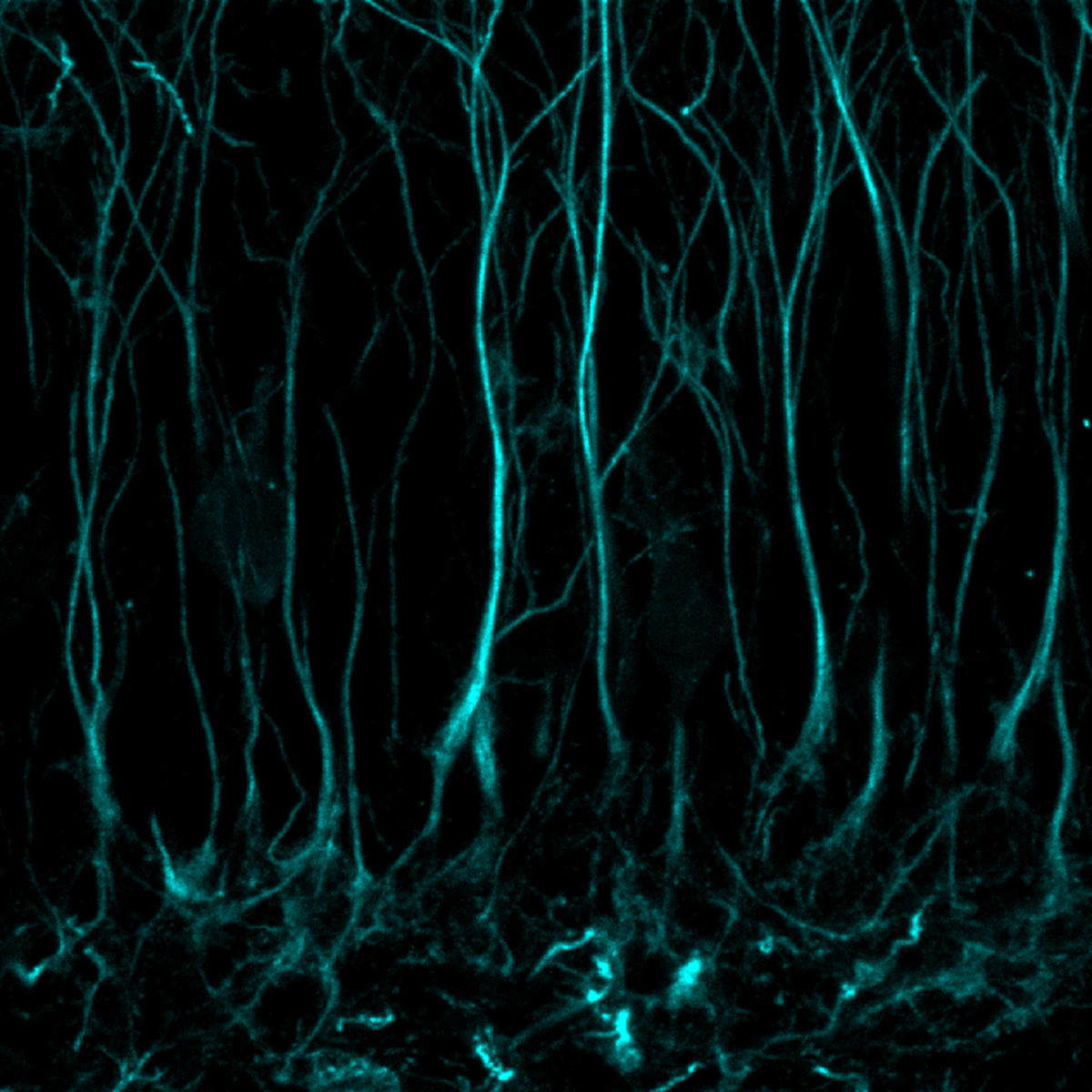

Supplement: Supplementary file 5 — Source Data Fig. 4 [file 44318_2023_11_MOESM5_ESM.zip › EMBOJ-2023-113564_SourceDataForFigure4/4A/P2_P14_DMSinjection/p2_p14 DMS inj_GFAP.tiff]

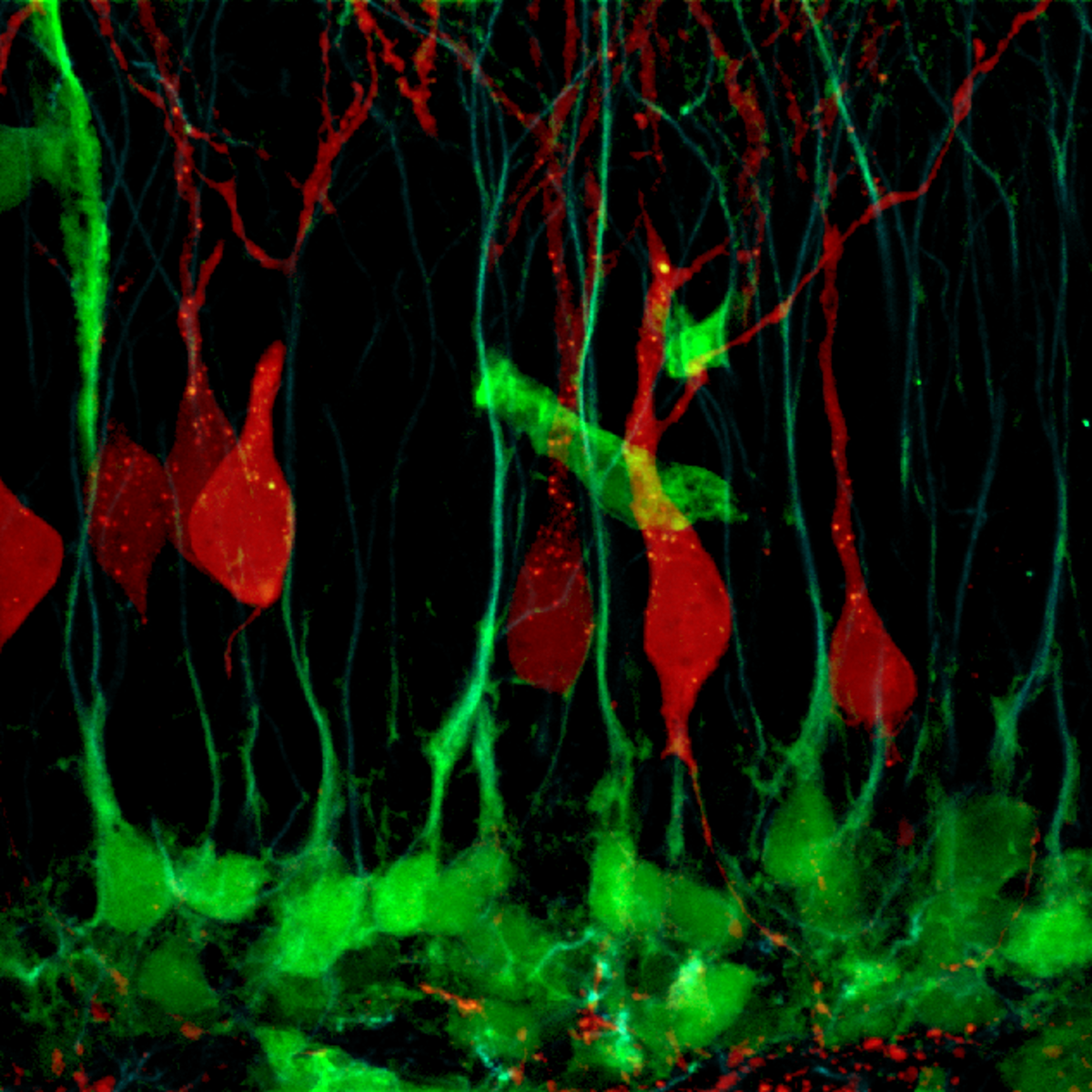

Supplement: Supplementary file 5 — Source Data Fig. 4 [file 44318_2023_11_MOESM5_ESM.zip › EMBOJ-2023-113564_SourceDataForFigure4/4A/P2_P14_DMSinjection/p2_p14 DMS inj_Merge.tiff]

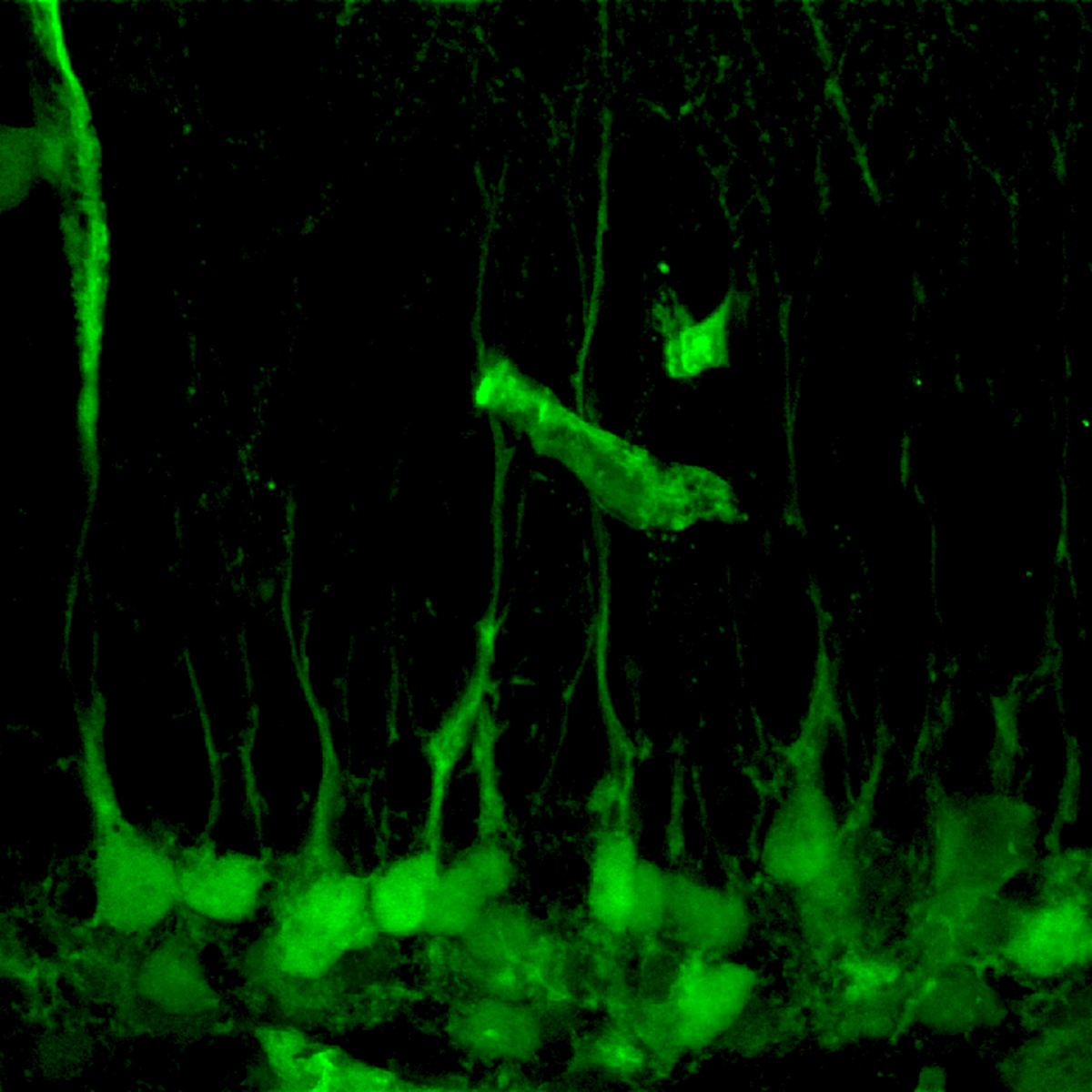

Supplement: Supplementary file 5 — Source Data Fig. 4 [file 44318_2023_11_MOESM5_ESM.zip › EMBOJ-2023-113564_SourceDataForFigure4/4A/P2_P14_DMSinjection/p2_p14 DMS inj_Nestin.tiff]

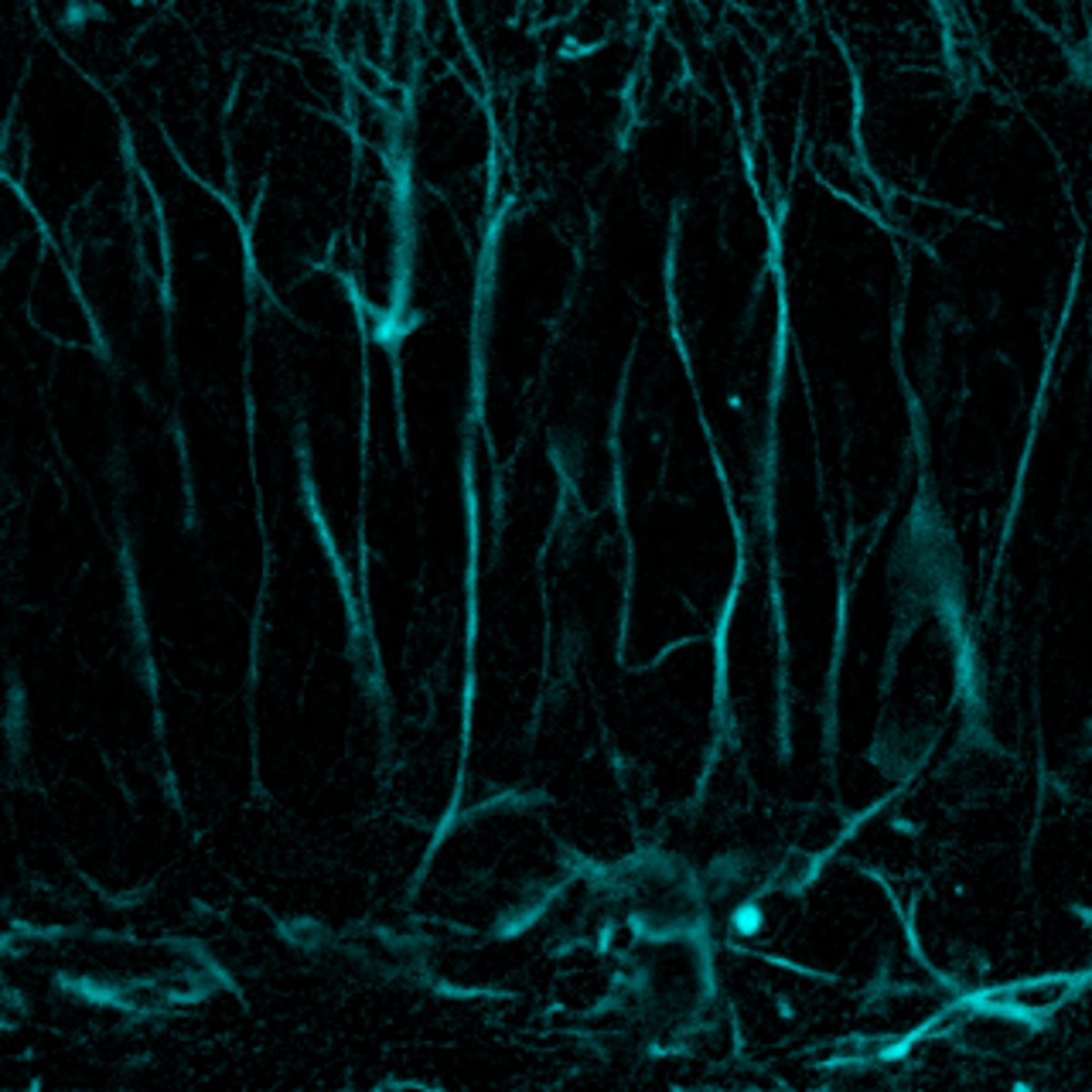

Supplement: Supplementary file 5 — Source Data Fig. 4 [file 44318_2023_11_MOESM5_ESM.zip › EMBOJ-2023-113564_SourceDataForFigure4/4B/P5_P14_DGinjection/p5_p14 DG inj_GFAP.tiff]

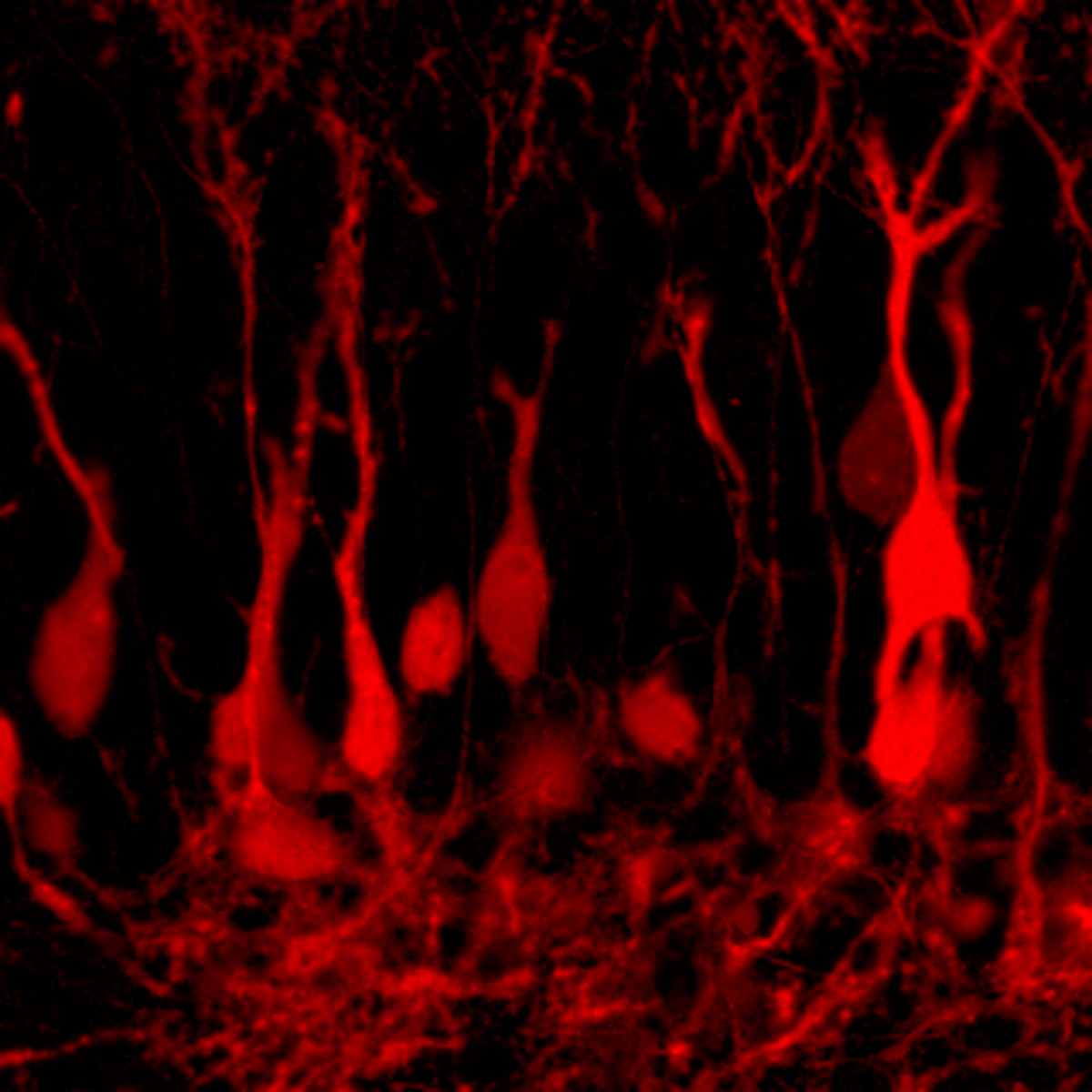

Supplement: Supplementary file 5 — Source Data Fig. 4 [file 44318_2023_11_MOESM5_ESM.zip › EMBOJ-2023-113564_SourceDataForFigure4/4B/P5_P14_DGinjection/p5_p14 DG inj_mCherry.tiff]

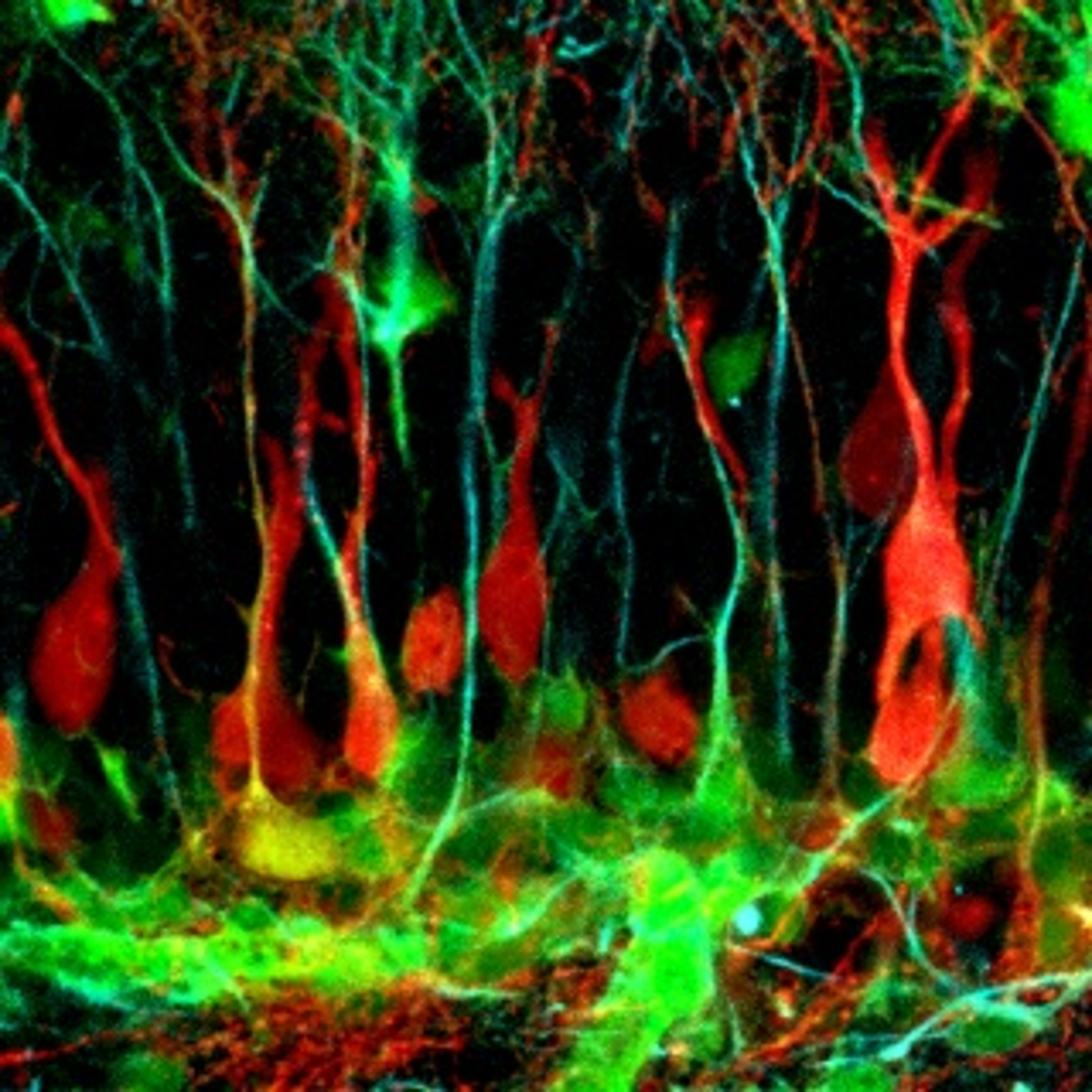

Supplement: Supplementary file 5 — Source Data Fig. 4 [file 44318_2023_11_MOESM5_ESM.zip › EMBOJ-2023-113564_SourceDataForFigure4/4B/P5_P14_DGinjection/p5_p14 DG inj_Merge.tiff]

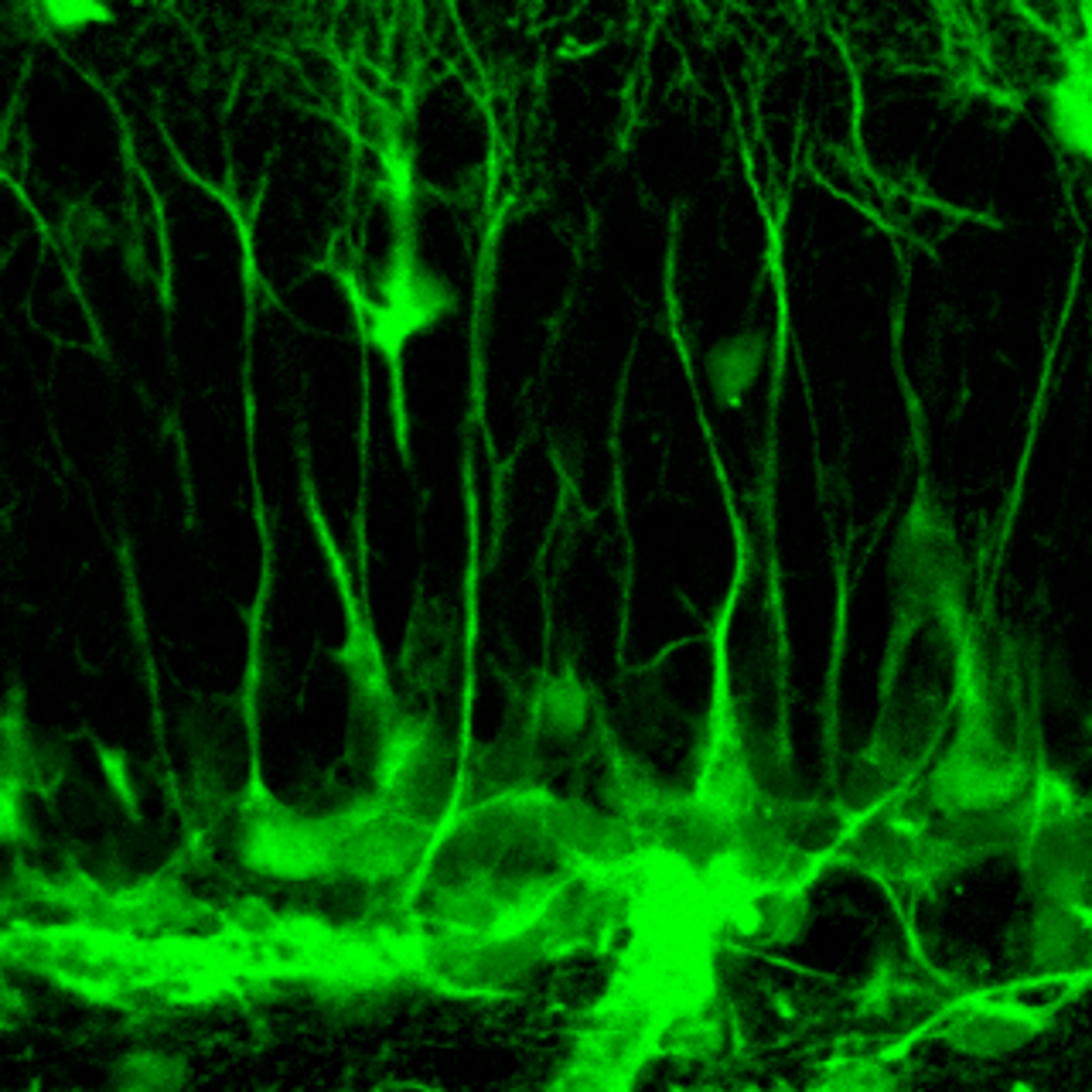

Supplement: Supplementary file 5 — Source Data Fig. 4 [file 44318_2023_11_MOESM5_ESM.zip › EMBOJ-2023-113564_SourceDataForFigure4/4B/P5_P14_DGinjection/p5_p14 DG inj_Nestin.tiff]

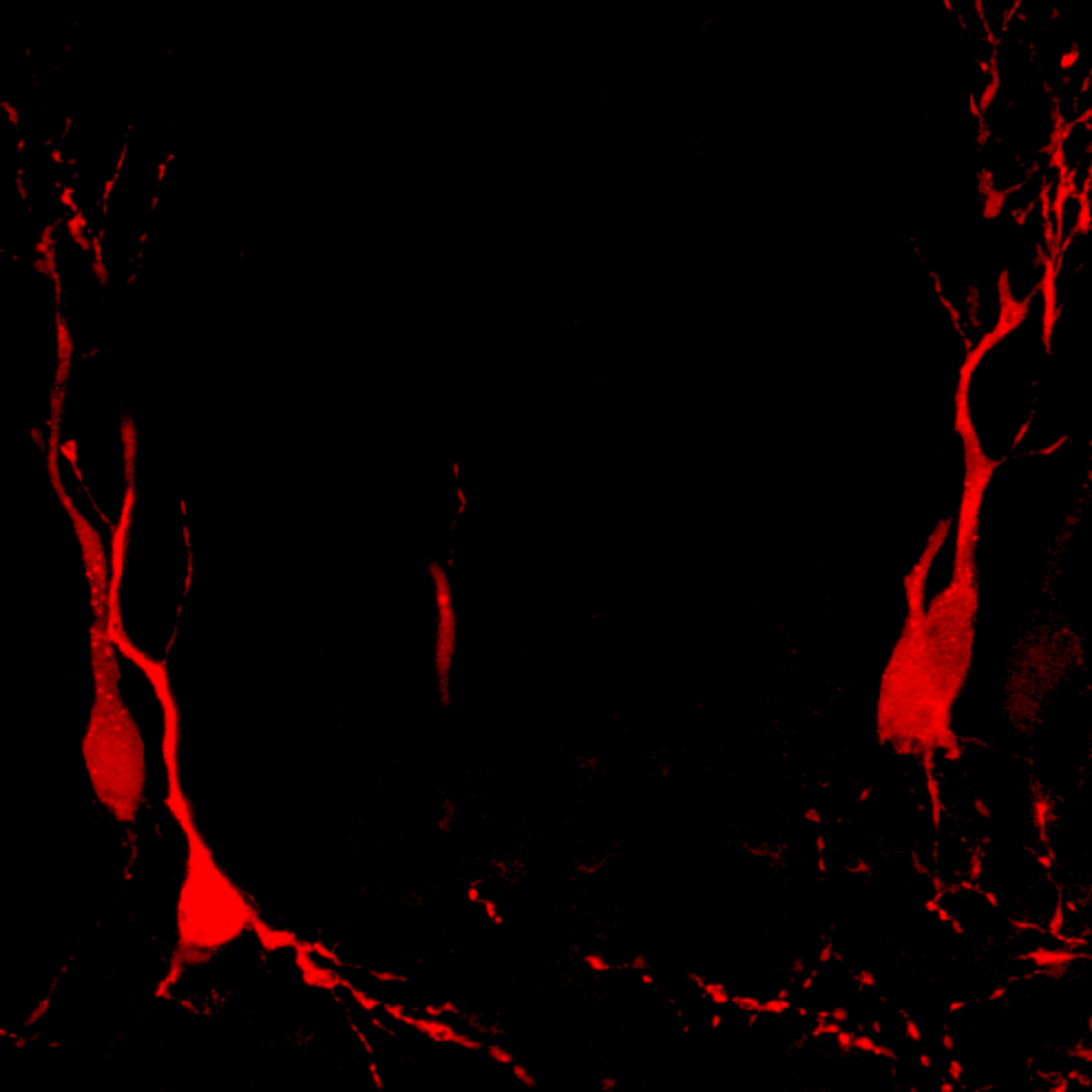

Supplement: Supplementary file 5 — Source Data Fig. 4 [file 44318_2023_11_MOESM5_ESM.zip › EMBOJ-2023-113564_SourceDataForFigure4/4B/P5_P14_DMSinjection/p5_p14 DMS inj_Cherry.tiff]

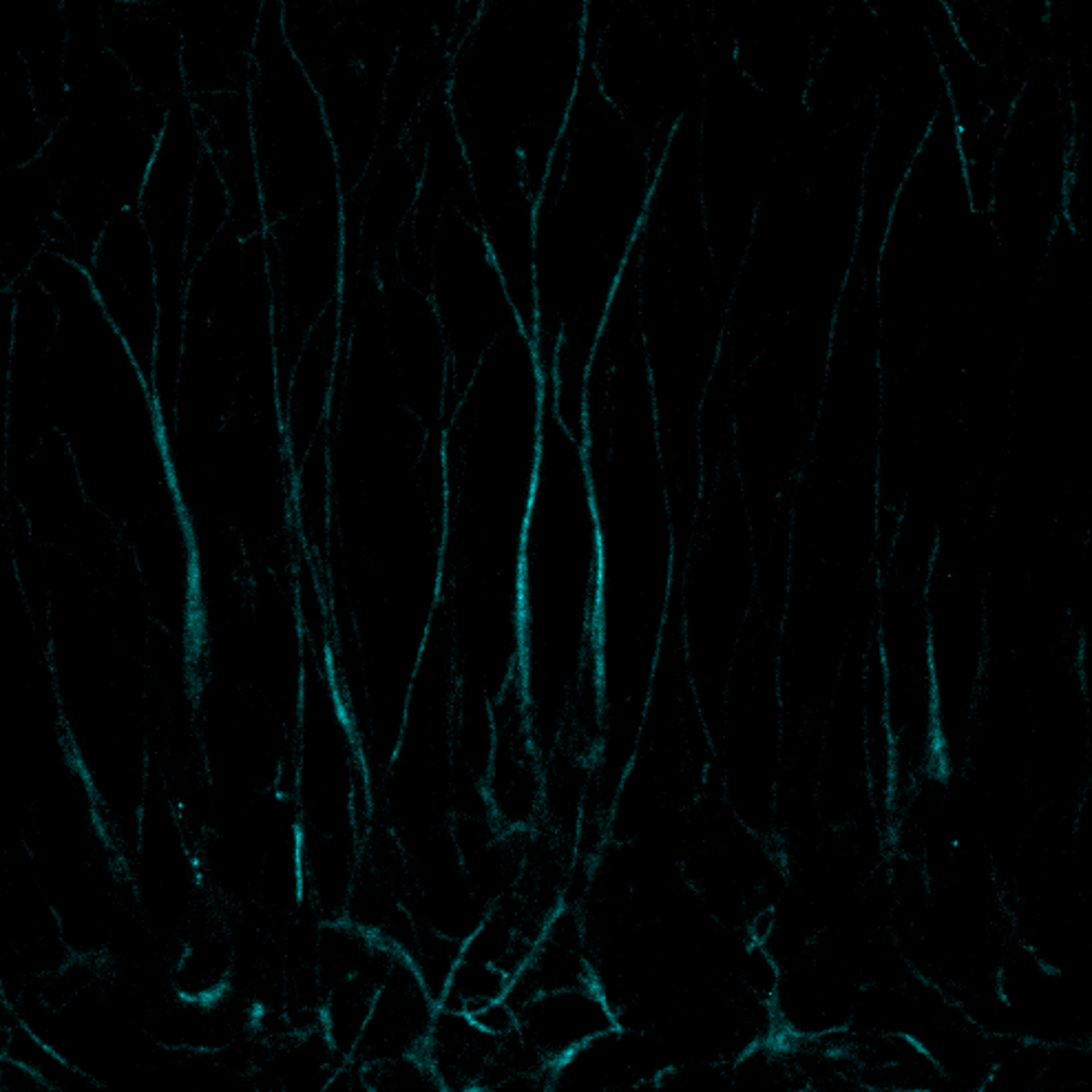

Supplement: Supplementary file 5 — Source Data Fig. 4 [file 44318_2023_11_MOESM5_ESM.zip › EMBOJ-2023-113564_SourceDataForFigure4/4B/P5_P14_DMSinjection/p5_p14 DMS inj_GFAP.tiff]

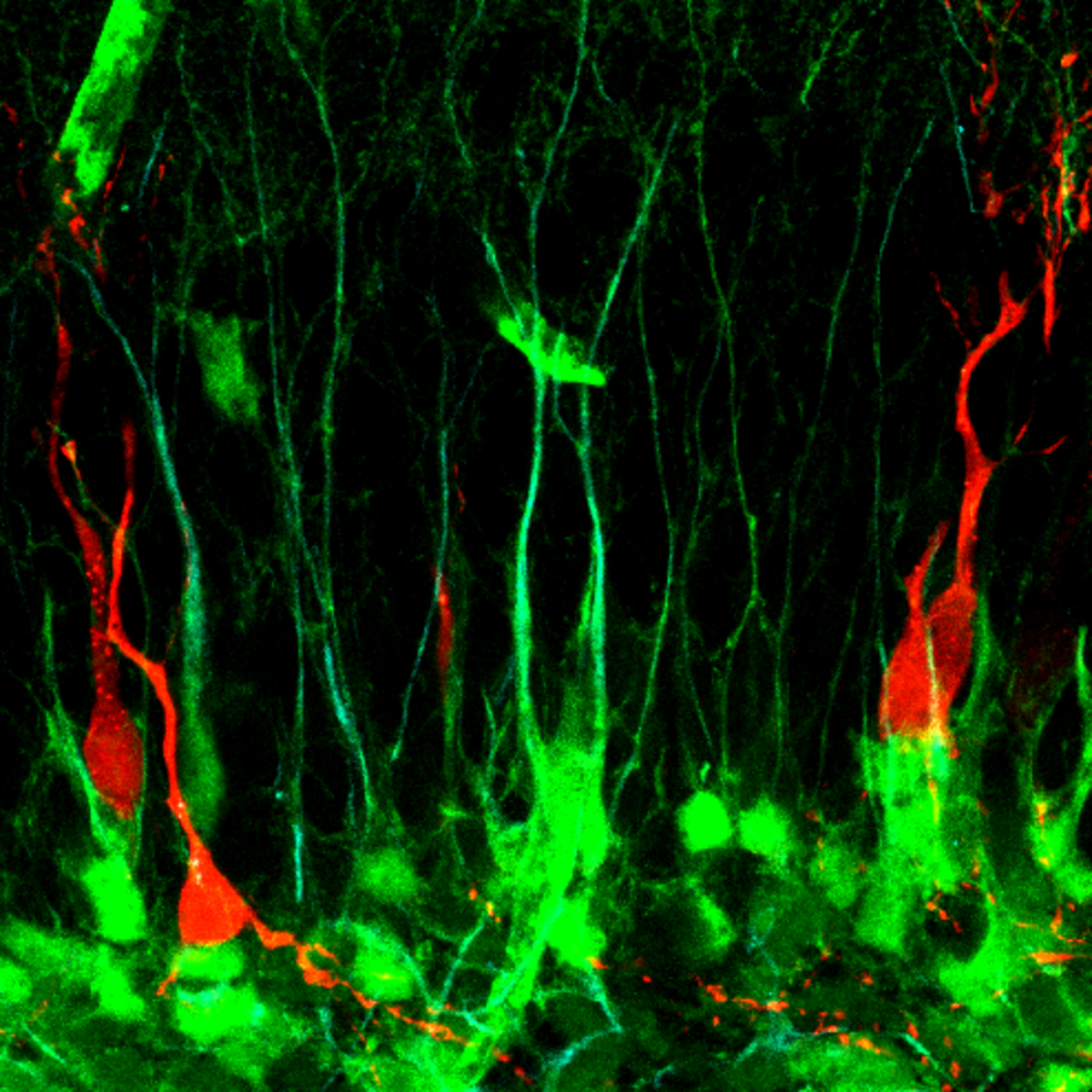

Supplement: Supplementary file 5 — Source Data Fig. 4 [file 44318_2023_11_MOESM5_ESM.zip › EMBOJ-2023-113564_SourceDataForFigure4/4B/P5_P14_DMSinjection/p5_p14 DMS inj_Merge.tiff]

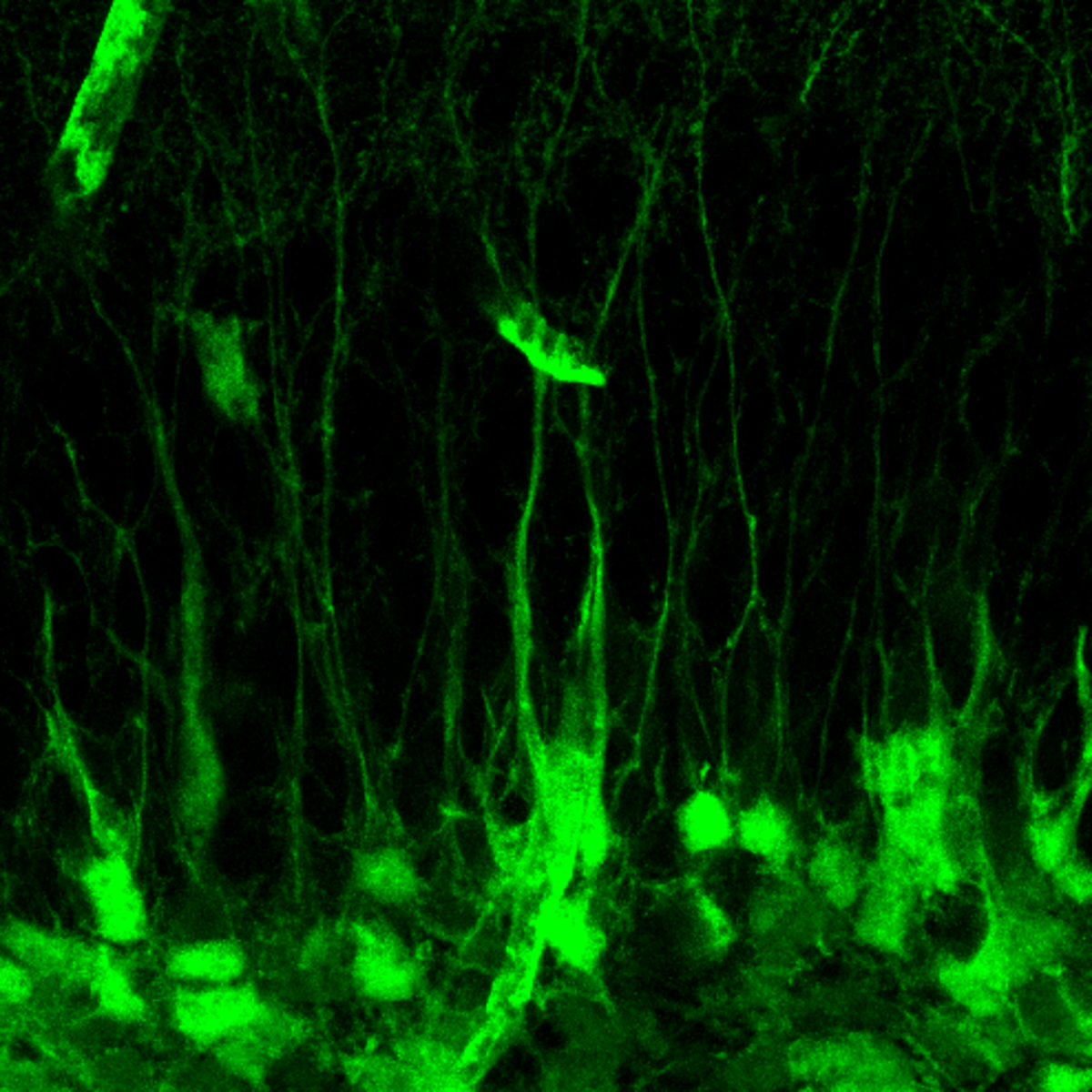

Supplement: Supplementary file 5 — Source Data Fig. 4 [file 44318_2023_11_MOESM5_ESM.zip › EMBOJ-2023-113564_SourceDataForFigure4/4B/P5_P14_DMSinjection/p5_p14 DMS inj_Nestin.tiff]

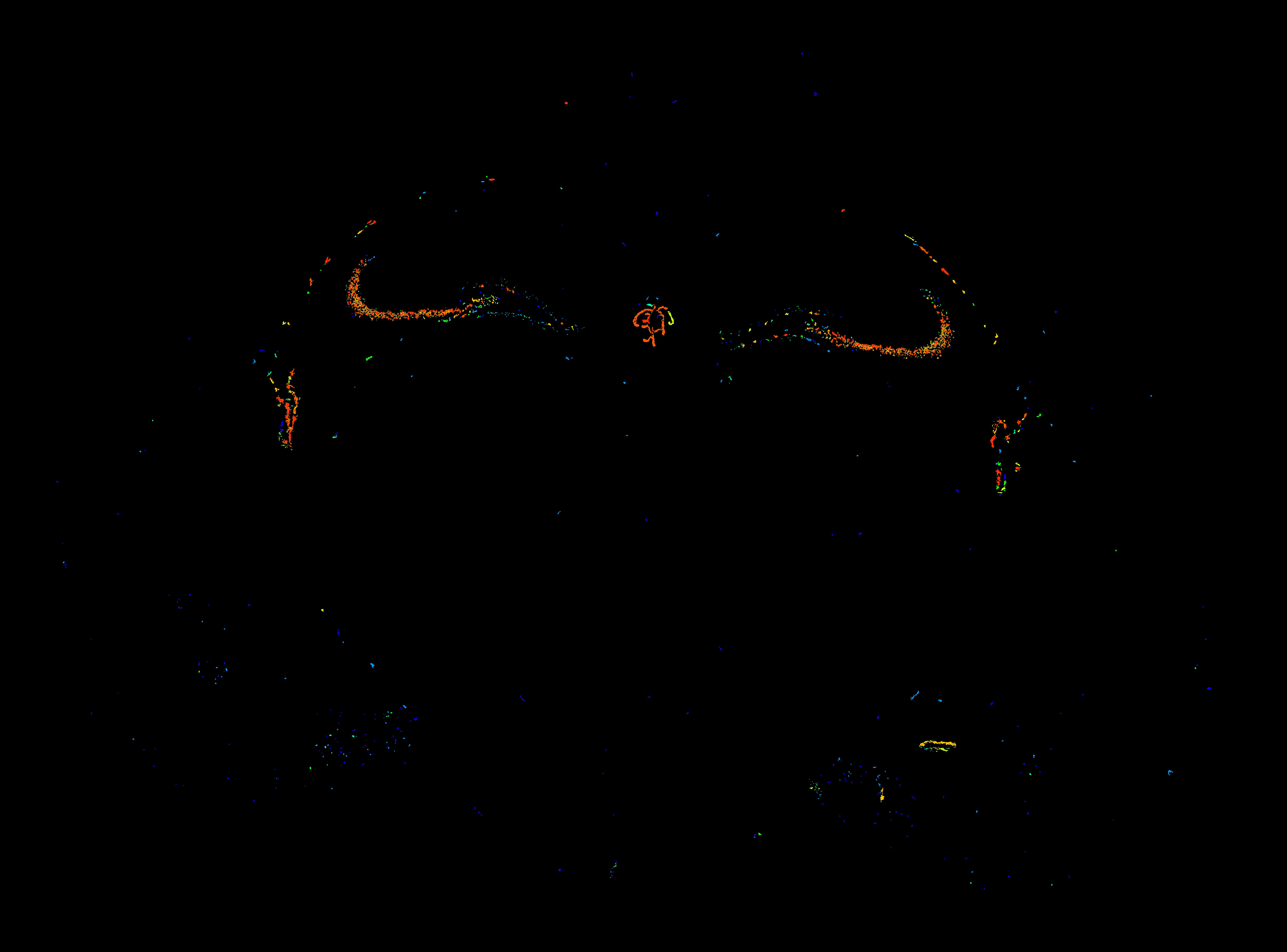

Supplement: Supplementary file 6 — Source Data Fig. EV1F [file 44318_2023_11_MOESM6_ESM.zip › EMBOJ-2023-113564R1_SourceDataForFigureEV1F/ccnd2_expression_205_222.jpg]

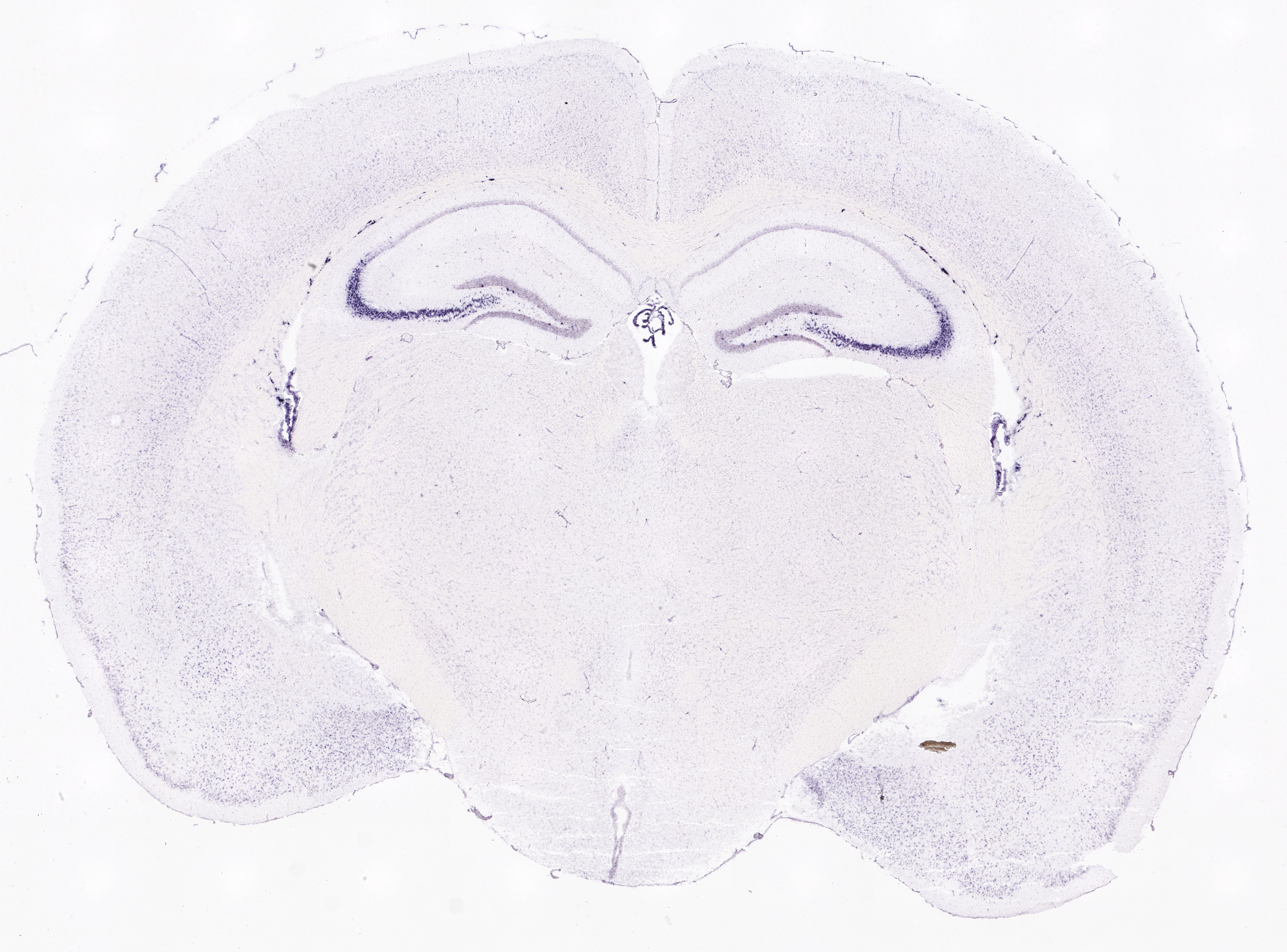

Supplement: Supplementary file 6 — Source Data Fig. EV1F [file 44318_2023_11_MOESM6_ESM.zip › EMBOJ-2023-113564R1_SourceDataForFigureEV1F/ccnd2_ISH_205_222.jpg]
