# Supplementary material for: Generation of adult hippocampal neural stem cells occurs in the early postnatal dentate gyrus and depends on cyclin D2
Source: EMBO J. 2023 Dec 20;43(3):1. doi: 10.1038/s44318-023-00011-2 (PMC10897295; doi:10.1038/s44318-023-00011-2)
Supplement: Supplementary file 7 — Expanded View Figures [file 44318_2023_11_MOESM7_ESM.pdf]

Expanded View Figures

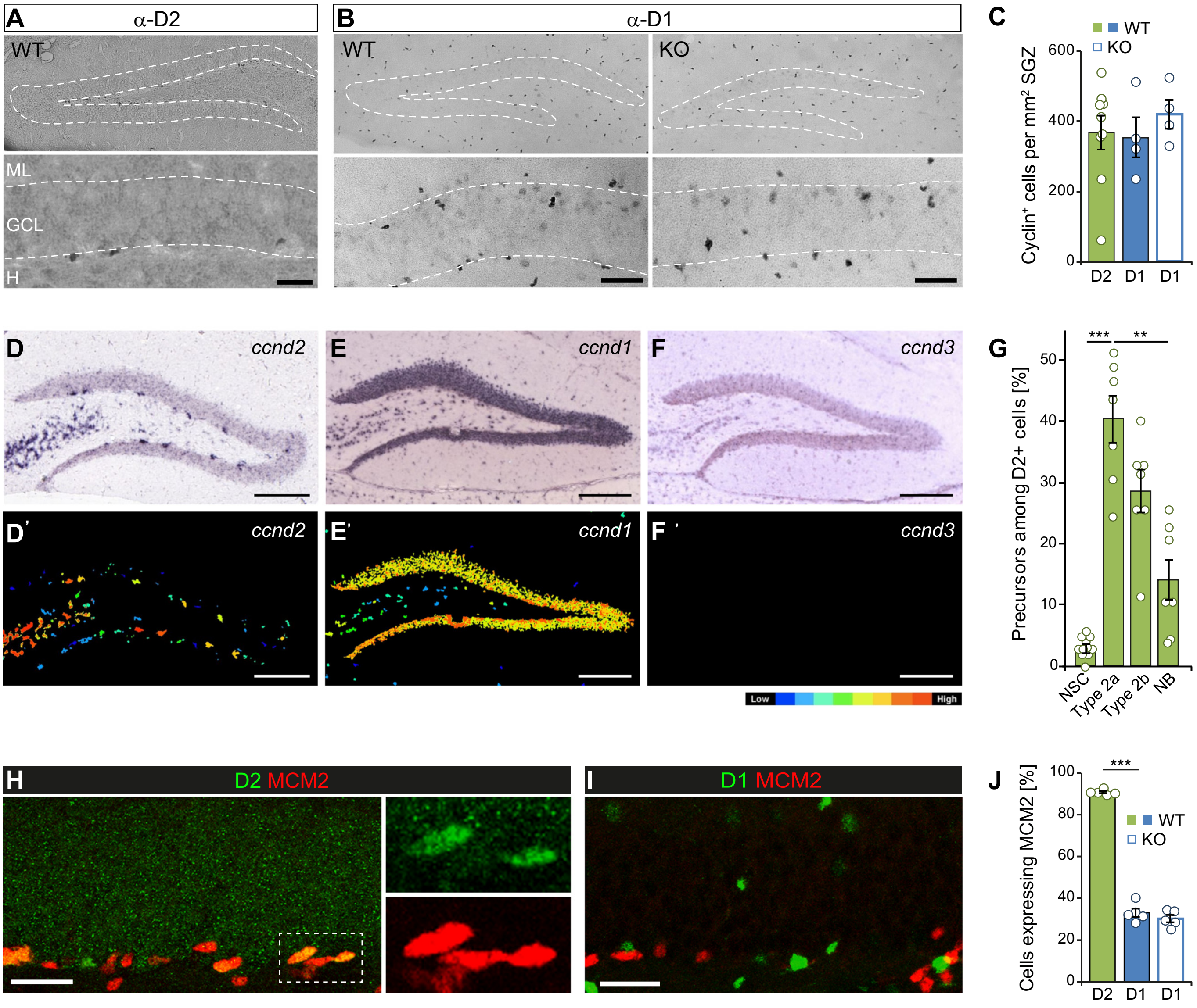

Figure EV1. Cyclin D2 is expressed by actively dividing progenitors in the adult SGZ.

(**A**, **B**) Peroxidase staining of coronal brain sections illustrating the distribution of cyclin D2 (**A**) and D1 (**B**) in the DG of WT and D2KO mice. Upper panels: overview of the DG, lower panels: magnified image of the suprapyramidal blade. (**C**) Density of cyclin D^{+} cells in the adult SGZ (D2: $n = 9$, D1: $n = 4$ mice/group). (**D–F**) Expression of *ccnd1* and *ccnd2* mRNA and lack of *ccnd3* in the adult DG. Allen Mouse Brain Atlas, https://mouse.brain-map.org/experiment/show/205, https://mouse.brain-map.org/experiment/show/69540507, https://mouse.brain-map.org/experiment/show/68191468. (**D´–F´**) Expression mask image display highlighting cells with highest probability of gene expression. (**G**) Quantification of the proportions of NSCs, type 2 cells and neuroblasts among cyclin $D2^{+}$ cells revealed a prevalence of transit-amplifying type 2 progenitors ($n = 7$ mice). (**H**, **I**) Confocal images illustrating the expression of MCM2 in cyclin $D2^{+}$ and $D1^{+}$ cell populations. Maximum intensity projections of 11 µm high *Z*-stacks. (**J**) Proportions of cyclin $D2^{+}$ and $D1^{+}$ cells expressing MCM2 ($n = 5$ mice/group). Data information: All values represent mean ± SEM. Statistics: One-way ANOVA (**C**, **J**) and One-way RM-ANOVA (**G**), $^{**}P < 0.01$, $^{***}P < 0.001$; Scale bars: 25 µm (**A**, **B**, **H**, **I**) and 200 µm (**D–F´**). ML molecular layer, GCL granule cell layer, H hilus. Source data are available online for this figure.

 © The Author(s)

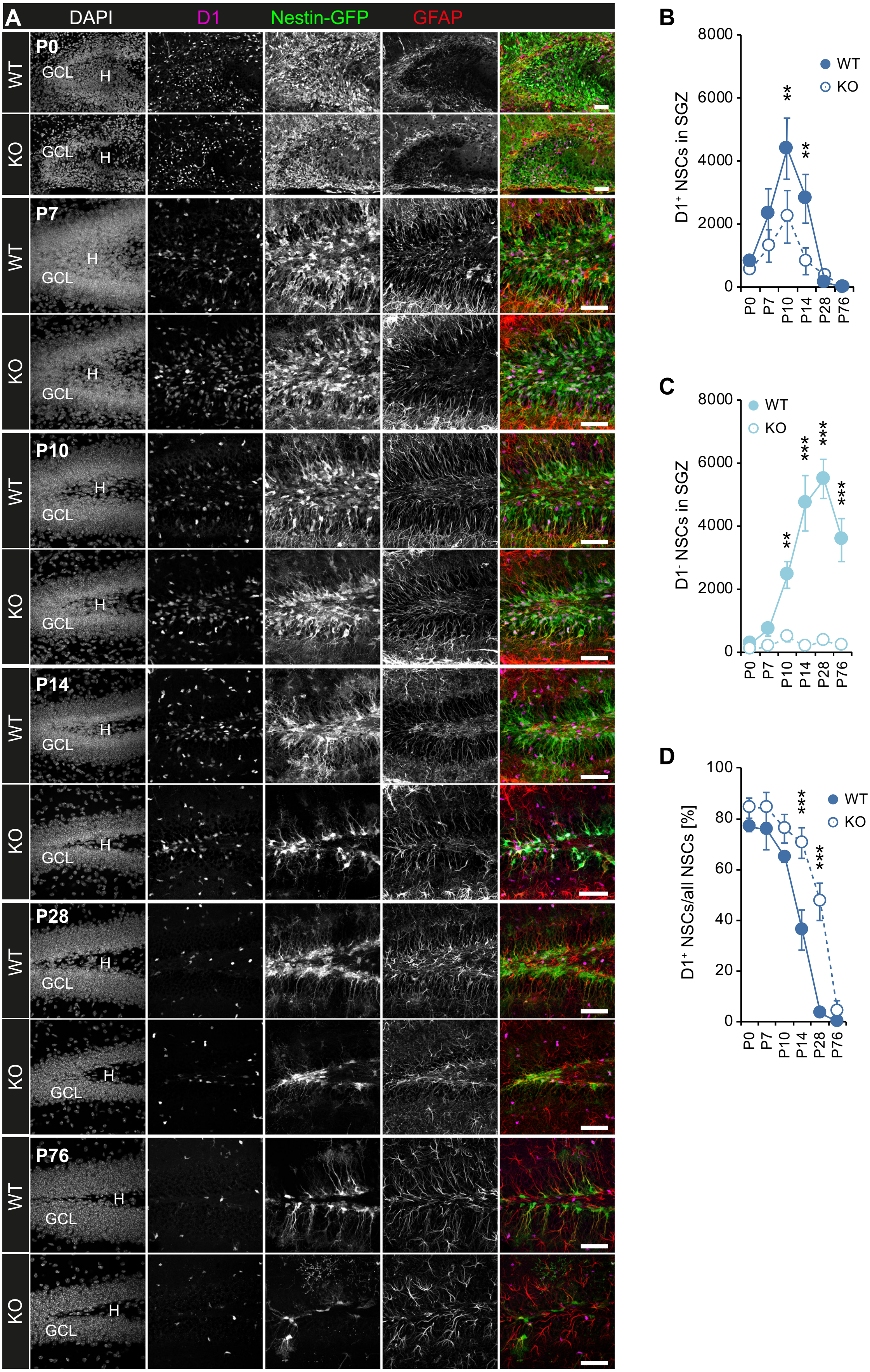
A
DAPI
D1
Nestin-GFP
GFAP
P0
P7
P10
P14
P28
P76
WT
KO
GCL
H
B
D1⁺ NSCs in SGZ
C
D1⁻ NSCs in SGZ
D
D1⁺ NSCs/all NSCs [%]
**
***

Figure EV2. The lack of cyclin D2 impairs the postnatal expansion of the cyclin D1⁻ NSC population and diminishes the transient cyclin D1⁺ NSC population.

(**A**) Confocal images of the developing DG of WT and D2KO mice immunostained against cyclin D1, nestin-GFP, GFAP and DAPI. Figures represent maximum intensity projections of 12.07 μm high *Z*-stacks. (**B**, **C**) Quantification of cyclin $D1^+$ and $D1^-$ NSC numbers in the SGZ. (**B**) Cyclin $D1^+$ NSCs are a transient population. Deletion of cyclin D2 affects but does not prevent their appearance, suggesting that cyclin D1 may either partially compensate for the lack of cyclin D2 or designate a distinct dNSC population. (**C**) Deletion of cyclin D2 prevents the expansion of the cyclin $D1^-$ NSC population. $N = 4$ mice/group except P10 KO and P14 WT with $n = 5$. (**D**) Proportions of NSCs expressing cyclin D1 are higher in mutant mice compared to WT ($n = 4$ mice/group except P10 KO and P14 WT with $n = 5$). Data information: Values represent mean ± SEM. statistics: 2-way ANOVA, $^{**}P < 0.01$, $^{***}P < 0.001$; Scale bars: 50 μm. H hilus, GCL granule cell layer.

© The Author(s)

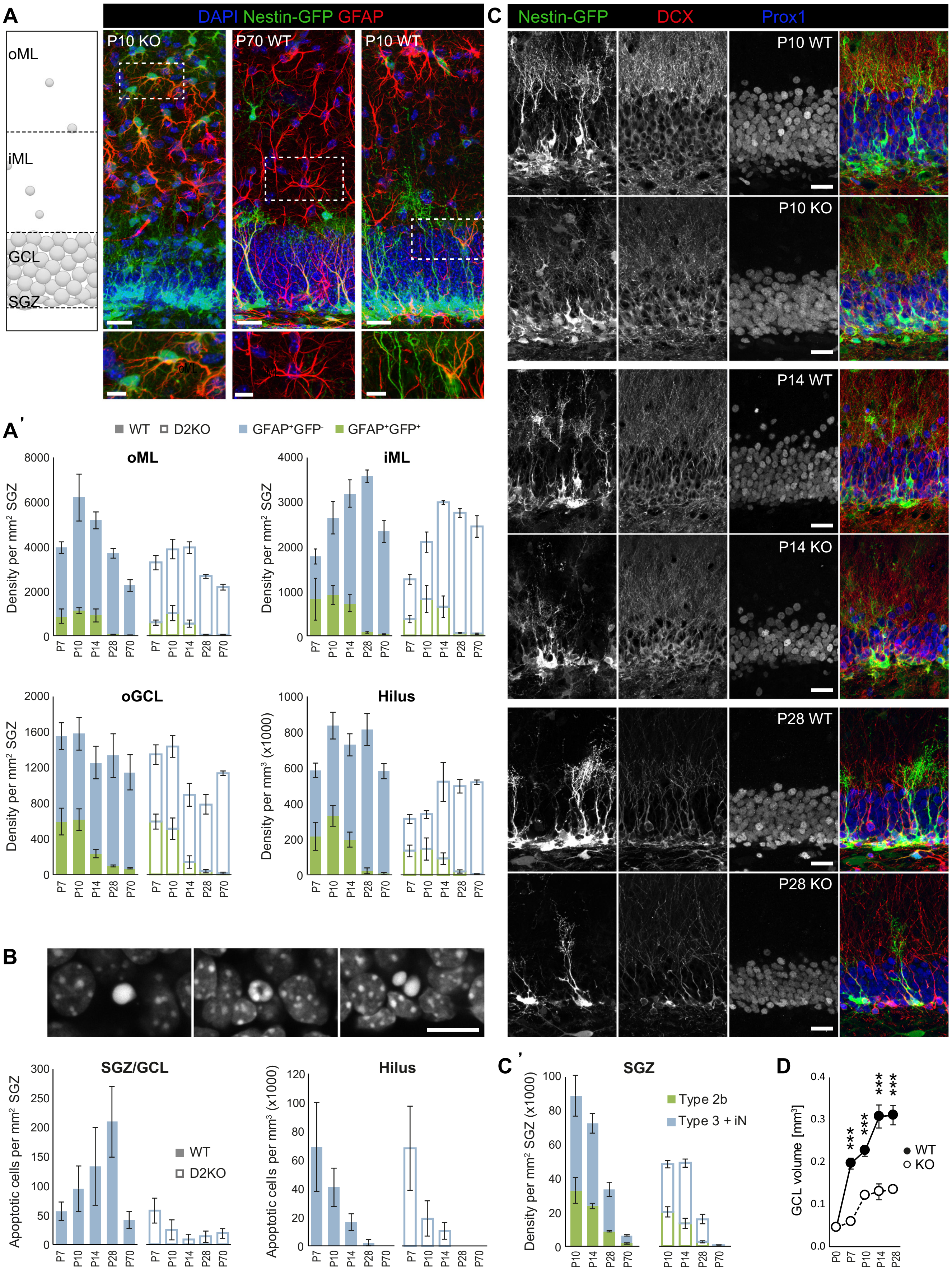
A
DAPI Nestin-GFP GFAP
oML
iML
GCL
SGZ
P10 KO
P70 WT
P10 WT
C
Nestin-GFP DCX Prox1
P10 WT
P10 KO
P14 WT
P14 KO
P28 WT
P28 KO
A'
WT D2KO GFAP+GFP- GFAP+GFP+
oML
iML
oGCL
Hilus
Density per mm² SGZ
Density per mm³ (x1000)
P7 P10 P14 P28 P70
B
SGZ/GCL
Hilus
Apoptotic cells per mm² SGZ
Apoptotic cells per mm³ (x1000)
WT
D2KO
C'
SGZ
Type 2b
Type 3 + iN
Density per mm² SGZ (x1000)
D
GCL volume [mm³]
WT
KO
***
P0 P7 P10 P14 P28

Figure EV3. The impaired formation of the aNSC pool in D2KO mice is not caused by apoptosis or differentiation of postnatal NSCs.

(**A**) Postnatal astrogliogenesis is not increased upon deletion of cyclin D2. Top panel (from left to right): Sample confocal images of immature ($GFAP^+GFP^+$) and mature ($GFAP^+GFP^-$) stellate astrocytes in the ML and of an immature polar astrocyte in the GCL. Images represent maximum intensity projections of 12.07 µm confocal *Z*-stacks; scale bars 20 µm and 10 µm in magnified images). (**A´**) Quantification of immature and mature astrocytes ($n = 4$/group). Because of the extensive migration of dNSC-derived astrocytes in the postnatal DG (Brunne et al, 2010), analysis was performed in different layers of the DG. (**B**) Quantification of apoptotic cells ($n = 4$ mice/group except $n = 5$ in P10 and P14 WT). Top panel: DAPI staining showing the morphotypes of nuclei (pyknotic, donut-shaped and karyorrhectic; single optical planes) considered as apoptotic. Scale bar 10 µm. (**C**, **C´**) Quantification of neuroblasts and immature neurons in the postnatal SGZ ($n = 4$ mice/group). Images represent maximum intensity projections of 6.9 µm confocal *Z*-stacks, scale bars represent 20 µm. (**D**) Quantification of the GCL volume ($n = 5$ mice/group except $n = 6$ in P7 KO and $n = 4$ in P28 WT; 2-way ANOVA, $^{***}P < 0.001$). Data information: Values represent mean ± SEM. SGZ subgranular zone, GCL granule cell layer, iML inner half of the molecular layer, oML outer half of the ML representing the subpial germinative niche of the developing DG.

© The Author(s)

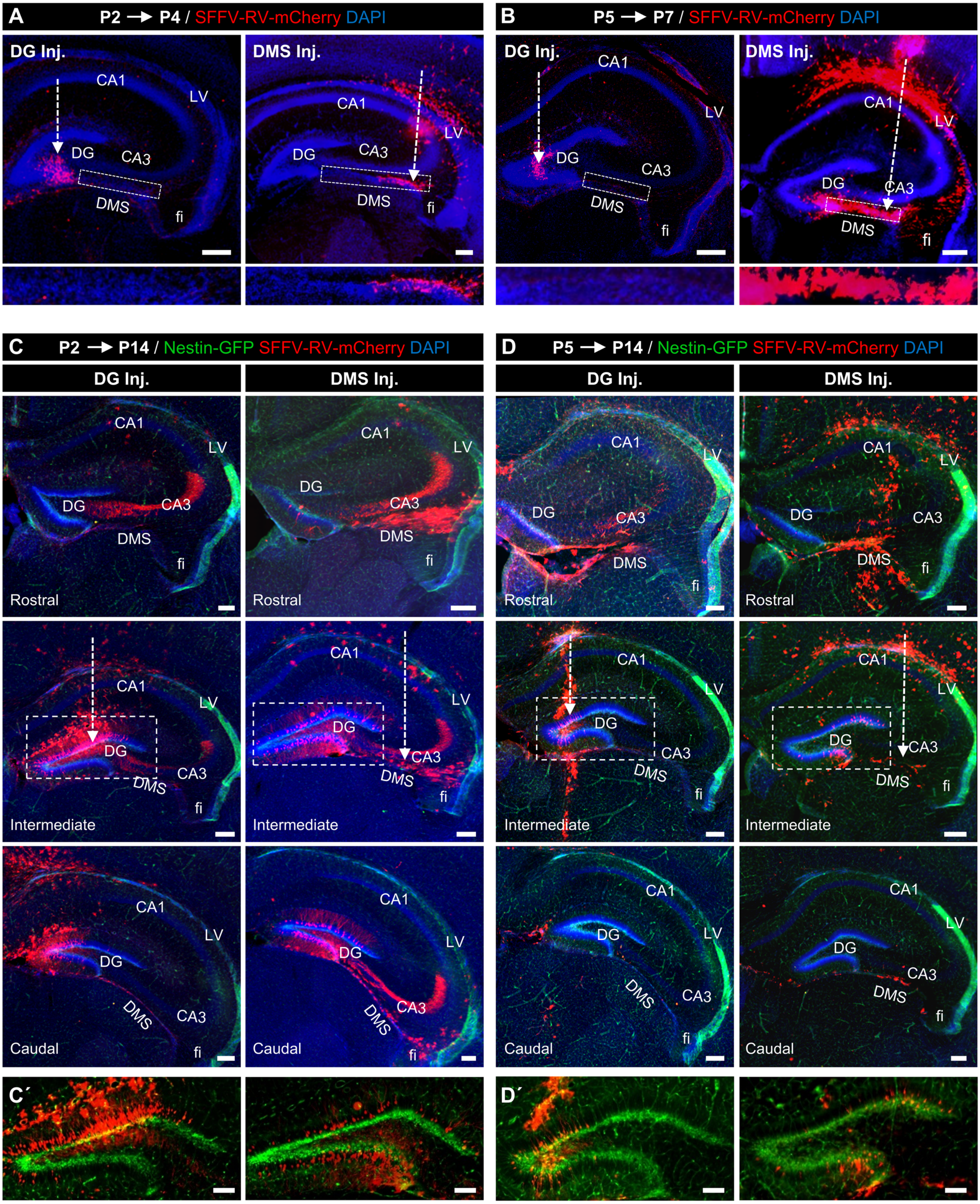
A
P2 → P4 / SFFV-RV-mCherry DAPI
DG Inj.
DMS Inj.
CA1
LV
DG
CA3
DMS
fi
B
P5 → P7 / SFFV-RV-mCherry DAPI
C
P2 → P14 / Nestin-GFP SFFV-RV-mCherry DAPI
D
P5 → P14 / Nestin-GFP SFFV-RV-mCherry DAPI
Rostral
Intermediate
Caudal
C´
D´

◀ **Figure EV4. Fluorescence slide-scanner images showing the spatial destination of SFFV-RV-mCherry-transduced cells after injection into the DG or in the DMS.**

(**A**, **B**) To evaluate the spatial precision of SFFV-RV injections into the DG or the DMS, C57Bl/6 mice were injected either at P2 (**A**) or P5 (**B**) and sacrificed 2 days later. The squares delimitate the DMS shown at higher magnification below, and arrows mark the injection path. At both ages, mCherry^{+} cells were observed exclusively at the injected site, verifying that the injection paradigm is effective for tracing the lineage of aNSCs from different postnatal niches. A wider distribution, including the DG near the fimbrodentate junction, was found in mice injected in the DMS, reflecting the migratory activity of the precursors located in that area. (**C**, **D**) Nestin-GFP expressing pups were injected at P2 (**C**) or P5 (**D**) and the spatial destination of transduced cells from rostral to caudal was assessed at P14. The arrows mark the injection path. Insets delineate the DG, which is shown at higher magnification in **C´** and **D´**. In all cases, we observed mCherry^{+} cells located in the GCL, as well as axons entering the hilus. However, mCherry^{+} cells connecting the LV with the DG were observable only when the injection was done in the DMS. Data information: Scale bars = 200 μm. CA cornu ammonis, DG dentate gyrus, DMS dentate migratory stream, fi fimbria, GCL granule cell layer, LV lateral ventricle.

 © The Author(s)
